# Family of magnetic field-boosted superconductors in rhombohedral graphene

**DOI:** 10.1038/s41586-026-10815-x

**Published:** 2026-06-29

**Authors:** Junseok Seo, Armel A. Cotten, Shenyong Ye, Mingchi Xu, Omid Sharifi Sedeh, Henok Weldeyesus, Tonghang Han, Zhengguang Lu, Zhenghan Wu, Wei Xu, Jixiang Yang, Emily Aitken, Prayoga P. Liong, Phatthanon Pattanakanvijit, Zach Hadjri, Rasul Gazizulin, Kenji Watanabe, Takashi Taniguchi, Mingda Li, Dominik M. Zumbühl, Long Ju

**Affiliations:** 1 Department of Physics, Massachusetts Institute of Technology, Cambridge, MA USA; 2 Department of Physics, University of Basel, Basel, Switzerland; 3 Department of Physics, Florida State University, Tallahassee, FL USA; 4 Department of Physics, University of Florida, Gainesville, FL USA; 5 National High Magnetic Field Laboratory High B/T Facility, University of Florida, Gainesville, FL USA; 6 Research Center for Electronic and Optical Materials, National Institute for Materials Science, Tsukuba, Japan; 7 Research Center for Materials Nanoarchitectonics, National Institute for Materials Science, Tsukuba, Japan; 8 Department of Nuclear Science and Engineering, Massachusetts Institute of Technology, Cambridge, MA USA

**Keywords:** Superconducting properties and materials, Electronic properties and materials

## Abstract

In some unconventional superconductors, time-reversal symmetry can be broken apart from the gauge symmetry^1^, resulting in superconductivity that can be enhanced or induced by magnetic fields^2^. However, field-enhanced superconductors are more vulnerable to impurities than their Bardeen–Cooper–Schrieffer counterparts^3^. Crystalline rhombohedral multilayer graphene is a promising platform to explore them because of its superior material quality and gate-tunable strong correlation effects^4,5^. Here we report transport measurements of rhombohedral tetralayer and pentalayer graphene, demonstrating a spectrum of clean-limit superconductivities. We found three different types of field-enhanced and field-induced superconductivities in the pentalayer. They are all robust against an in-plane field up to 8.5 T, exceeding the Pauli limit by tens of times. Compared with Bernal bilayer graphene showing only in-plane field-enhancement^6^, pentalayer graphene features superconductors enhanced by out-of-plane as well as in-plane fields. They also reside at much lower gate electric fields owing to the intrinsically flatter band dispersion—facilitating their study and further engineering. Moreover, we observed that proximitized spin–orbit coupling generates multiple new superconductors without introducing additional disorder effects. Our work establishes a new family of magnetic field-boosted superconductors in rhombohedral graphene. Using the high accessibility with moderate gate voltages, this will pave the way for realizing non-Abelian quasiparticles through interfacial engineering^7^ in the extreme clean limit, in that proximitized spin–orbit coupling leads to topological states^8^ and maintains the ultrahigh quality of crystalline graphene.

## Abstract (web summary)

Transport measurements show multiple magnetic-field-enhanced and field-induced superconducting states in rhombohedral graphene, providing a clean, tunable platform for studying unconventional superconductivity and topological quantum states.

## Main

In a strongly correlated system, the normal state of a superconductor is subject to diverse instabilities. This can result in unconventional superconductivity (SC) breaking a symmetry on top of global gauge symmetry^1^. Unconventional SCs play a key part in understanding correlated electron physics and can enable fault-tolerant quantum computations^9^. Despite its importance, it remains one of the biggest open questions in condensed matter physics owing to the intricate interplay between various broken symmetries and their effects on pairing mechanisms. In bulk materials^10–16^, studying doping-dependent behaviour relies on comparing different crystals, which can be complicated by varied defect levels. By contrast, two-dimensional materials allow for in situ tunable doping by electrostatic gating, which enables comparing hundreds of samples effectively in a reliable fashion. Especially, unconventional SC has been actively explored in moiré superlattices^17–23^ because of flexibility in interlayer twist angles. Nevertheless, the requirement of a precise twist angle and its spatial variation^24^ have posed challenges to reproducing phase diagrams from different devices^25^.

Rhombohedral *N*-layer graphene (RNG) has recently emerged as an ideal platform to study unconventional SC. It features a simple chemical ingredient and homogeneous crystalline structure, facilitating the search of unconventional SCs vulnerable to impurities^3^. Moreover, it hosts a flat low-energy band dispersion (*E* ∝ *k*^*N*^) that can be further fine-tuned by gate voltages^26^. These features foster enhanced electron correlation effects and various isospin-symmetry-broken states^4,5^, which can lead to SCs mediated by mechanisms other than phonons^27–29^. Although SCs in rhombohedral bilayer graphene (R2G)^6,30,31^ and trilayer graphene (R3G)^32–34^ have been studied, unconventional SCs in thicker RNG remain less explored. The latter, however, offers several advantages over its thinner counterparts. First, RNGs with *N* > 3 provide richer isospin-symmetry-broken states, such as the layer-antiferromagnet^4,5^ and valley-polarized half-metal^35^, in which exotic SCs can emerge. Second, isospin-symmetry-broken parent states are induced by lower gate electric fields for *N* > 3, reducing the risk of gate leakage. Third, RNGs with *N* > 3 have shown a rich family of quantum anomalous Hall (QAH) states^8,36–38^ that can lead to non-Abelian quasiparticles when combined with SC^7,39,40^.

Here, we report transport measurements on hole-doped rhombohedral tetralayer (R4G) and pentalayer graphene (R5G) without proximitized spin–orbit coupling (SOC) and moiré effects. We observed multiple superconducting states, highlighting several unconventional ones in R5G that are enhanced or induced by magnetic fields: (1) SC2 is strengthened by an in-plane magnetic field *B*_∥_; (2) SC4 is induced by a high *B*_∥_; (3) SC3 is enhanced by a small out-of-plane magnetic field *B*_⊥_ and is robust against *B*_∥_. These observations imply their unconventional nature. From a separate R4G/WSe_2_ device, we observed several new SCs induced by proximitized SOC effects. These states are located at relatively low displacement fields, and their parent states preserve the long mean free path of bare graphene—both facts will facilitate unconventional SC through interface engineering. The main findings in this work were reproduced in two R4G and five R5G devices and in two independent fridges from two different labs.

## SC in rhombohedral multilayer graphene

**Fig. 1:**
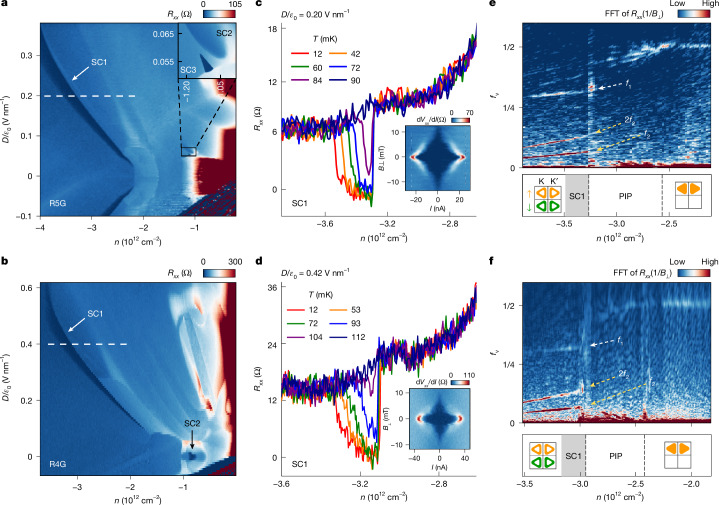
SC in hole-doped bare RNG. **a**,**b**, *R*_*xx*_ as a function of *n* and *D*/*ε*_0_ for devices R5G-1 (**a**) and R4G-1 (**b**), respectively, at zero magnetic field. The inset of **a** shows a magnified map corresponding to the black box. Several regions with vanishing *R*_*xx*_ are observed, including the SC1 akin to that observed in R3G and three new SC states labelled as SC2 and SC3. **c**,**d**, *R*_*xx*_ as a function of *n* at varied temperatures and fixed *D*/*ε*_0_ for SC1 in devices R5G-1 (**c**) and R4G-1 (**d**), respectively. The inset shows differential resistance as a function of d.c. current and out-of-plane magnetic field in R5G (*n* = −3.315 × 10^12 ^cm^−2^, *D*/*ε*_0_ = 0.20 V nm^−1^) and R4G (*n* = −2.58 × 10^12 ^cm^−2^, *D*/*ε*_0_ = 0.28 V nm^−1^). Vanishing *R*_*xx*_, nonlinear d*V*_*xx*_/d*I* and the modulation of *I*_c_ by *B*_⊥_ suggest that these are superconducting states. **e**,**f**, Fast Fourier transform of *R*_*xx*_(1/*B*_⊥_) as a function of *n* and the normalized frequency *f*_*ν*_ along the dashed lines in **a** and **b**, respectively. The relation *f*_1_ − *f*_2_ = 1/4 holds for SC1 in both R5G and R4G (grey range in the bottom panel), suggesting an annular-shaped full-metal as the parent state. The partially isospin-polarized phase (PIP) appears next to SC1. FFT, fast Fourier transform.

**Extended Data Fig. 1:**
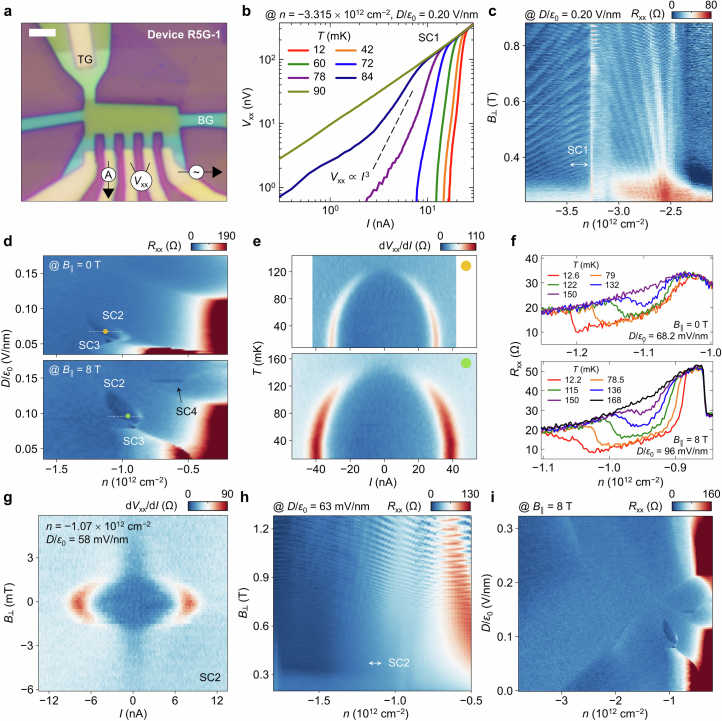
Additional data for SC1 and SC2 in R5G. **a**, Optical image of Device R5G-1 with a representative measurement configuration. Scale bar: 3 μm. **b**, Voltage *V*_xx_ versus current *I* at varied temperatures for the SC1 of R5G. **c**, *R*_xx_ as a function of *n* and *B*_⊥_, covering the density range of the SC1. The data in **c** was used for the Fourier transform in Fig. 1e. **d**, *R*_xx_ maps at *B*_║_ = 0.0 (top) and 8.0 (bottom) T from Device R5G-1. While SC3 is robust, SC2 is enlarged and SC4 is induced by *B*_║_. **e**, Differential resistance as a function of *I* and *T* at the orange and green dot positions in **d**. Both *I*_c_ and *T*_BKT_ are enhanced by *B*_║_. **f**, *R*_xx_ as a function of *n* at varied temperatures along the dashed lines in **d**. The *R*_xx_ dip corresponding to SC2 persists to higher temperatures under finite *B*_║_. **g**, Differential resistance as a function of *I* and *B*_⊥_ at SC2 (*n* = −1.07 × 10^12 ^cm^−2^, *D*/*ε*_0_ = 58 mV nm) in Device R5G-1. The disappearance of nonlinear d*V*_xx_/d*I* by *B*_⊥_ is consistent with SC phenomenology. In addition, *B*_c,⊥_ ≈ 1.5 mT and *R*_n_ ≈ 30 Ω at this (*n*, *D*) point, which puts the SC2 around the clean limit (*ξ* ≈ 470 nm and *l* ≈ 1.1 μm). **h**, Landau fan diagrams taken at *D*/*ε*_0_ = 63 mV/nm as a function of *n*. This data was used for the Fourier transform in Fig. 2e. **i**, *R*_xx_ map in a large *n*-*D* space at *B*_║_ = 8.0 T. SC1 is fully killed at this magnetic field.

**Extended Data Fig. 2:**
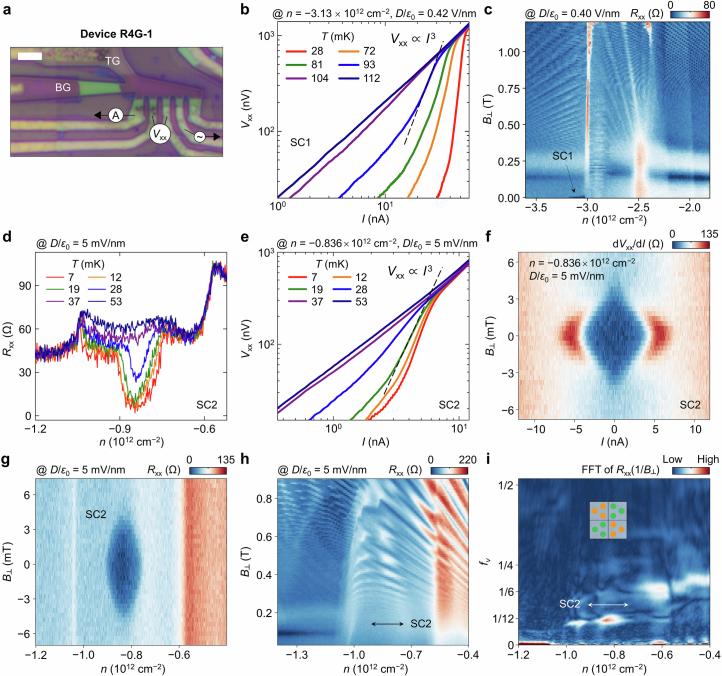
Additional data for SC1 and SC2 in R4G. **a**, Optical image of Device R4G-1 with a representative measurement configuration. Scale bar: 3 μm. **b**, Voltage *V*_xx_ versus current *I* at varied temperatures for the SC1 of R4G. **c**, *R*_xx_ as a function of *n* and *B*_⊥_, covering the density range of the SC1. The data in **c** was used for the Fourier transform in Fig. 1f. **d**, *R*_xx_ as a function of *n* at varied temperatures for the SC2 of R4G. **e**, Voltage *V*_xx_ versus current *I* at varied temperatures for the SC2. The dashed line denotes when *V*_*xx*_ is proportional to *I*^3^, corresponding to the BKT transition temperature *T*_BKT_ ≈ 19 mK. **f**, Differential resistance as a function of *I* and *B*_⊥_ at *n* = −0.836 × 10^12 ^cm^−2^ and *D*/*ε*_0_ = 5 mV/nm. Vanishing *R*_xx_, nonlinear d*V*_xx_/d*I*, and their quick suppression by *B*_⊥_ suggest SC2 in R4G is a superconductor. In addition, *B*_c,⊥_ ≈ 3 mT and *R*_n_ ≈ 60 Ω at this (*n*, *D*) point, putting the SC2 near the clean limit (*ξ* ≈ 330 nm and *l* ≈ 660 nm). **g**, *R*_xx_ as a function of *n* and *B*_⊥_ at *D*/*ε*_0_ = 5 mV/nm. **h**, Landau fan diagrams taken at *D*/*ε*_0_ = 5 mV/nm as a function of *n*. **i**, Fourier transform of *R*_xx_(1/*B*_⊥_) as a function of *n* and *f*_*ν*_. There is a peak at *f*_*ν*_ = 1/12, which suggests the normal state of SC2 in R4G is a trigonally warped full metal.

Figure 1a,b shows the *R*_*xx*_ maps of R5G and R4G at the mixing chamber temperature *T* of 7 mK, highlighting several regions with vanishing resistance. We label each state as SC1–SC3. Figure 1c,d shows *R*_*xx*_ linecuts at varied temperatures. *R*_*xx*_ drops to zero as *T* decreases in a range of charge densities. The insets show d*V*_*xx*_/d*I* as a function of source–drain current *I* and *B*_⊥_. Peaks in d*V*_*xx*_/d*I* are visible at zero *B*_⊥_ and get suppressed by nonzero *B*_⊥_. Fermiology analyses (Methods) demonstrate that SC1 is developed at the phase boundary between a full-metal with an annular Fermi surface and a partially isospin-polarized phase (Fig. 1e,f), identically to SC1 in R3G^32^. Temperature-dependent *R*_*xx*_, d*V*_*xx*_/d*I* and fermiology analyses for SC2 are shown in Extended Data Figs. 1 and 2.

The decrease in *R*_*xx*_, nonlinear d*V*_*xx*_/d*I* and response to *B*_⊥_ indicate that SC1 and SC2 in R5G and R4G are superconductors. Their phenomenology resembles SCs in moiré graphene^17–21^, twisted WSe_2_ (refs. ^22,23^) and thinner rhombohedral graphene^6,30–34^. However, the coherence length *ξ* and mean free path *l* of these SCs and their normal state (Methods) put themselves in the clean limit (*ξ*/*l* ≪ 1), in that *ξ* ≈ 200 nm, *l* ≈ 1.6 μm and *ξ* ≈ 300 nm, *l* ≈ 1 μm for SC1 in R5G and R4G, respectively. This is contrary to twisted graphene and WSe_2_ superconductors, in which the normal-state resistance is higher and *ξ*/*l* > 1. Rhombohedral graphene in the clean limit provides an ideal playground for exploring unconventional SCs, which are discussed in the following sections.

## SC2 enhanced and SC4 induced by *B*_∥_

**Fig. 2:**
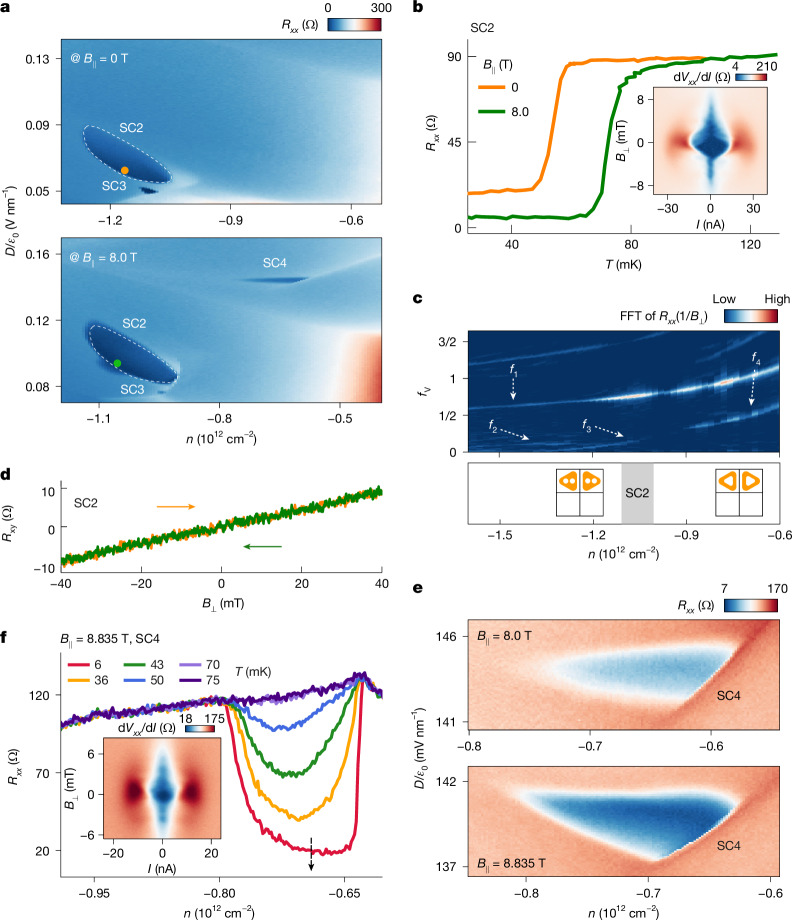
SC2 and SC4 in R5G, respectively, enhanced and induced by *B*_∥_. **a**, *R*_*xx*_ maps at *B*_∥_ = 0 T (top) and 8 T (bottom). Whereas SC3 survives, SC2 is enlarged, and SC4 is induced by *B*_∥_. For comparison, the boundary of SC2 at *B*_∥_ = 0 T is drawn as white dashed curves. **b**, *R*_*xx*_ as functions of *T* at the (*n*, *D*) point (indicated as circles in **a**) with the minimal resistance at each field. The transition temperature is increased by applying *B*_∥_. The inset shows differential resistance as a function of *I* and *B*_⊥_ for SC2 at *B*_∥_ = 6.5 T. Fraunhofer-like modulations are observed. **c**, Fast Fourier transform of *R*_*xx*_(1/*B*_⊥_) as a function of *n* and *f*_*ν*_. Around SC2, *f*_2_ and *f*_3_ have merged and the relation *f*_1_ − 2*f*_3_ ≈ 1/2 holds, suggesting its normal state is a half-metal with an annular-shaped Fermi surface. **d**, *R*_*xy*_ as a function of *B*_⊥_ in the forward and backward sweeping directions in the normal state of SC2 (*n* = −1.07 × 10^12 ^cm^−2^, *D*/*ε*_0_ = 58 mV nm^−1^, *B*_∥_ = 0 T, *T* = 160 mK). The absence of the anomalous Hall effect indicates zero valley polarization. **e**, *R*_*xx*_ maps magnifying into SC4 at *B*_∥_ = 8.0 T (top) and 8.835 T (bottom). The resistance of SC4 decreases as applying higher *B*_∥_. **f**, *R*_*xx*_ as a function of *n* at varied temperatures and *D*/*ε*_0_ = 140.1 mV nm^−1^ for SC4 at *B*_∥_ = 8.835 T. A clear decrease in *R*_*xx*_ is observed as *T* decreases. The inset shows differential resistance as a function of *I* and *B*_⊥_ for SC4 at *B*_∥_ = 8.835 T and the *n* value marked by the dashed arrow. Fraunhofer-like modulations are observed. All the data were taken from device R5G-2. FFT, fast Fourier transform.

**Fig. 3:**
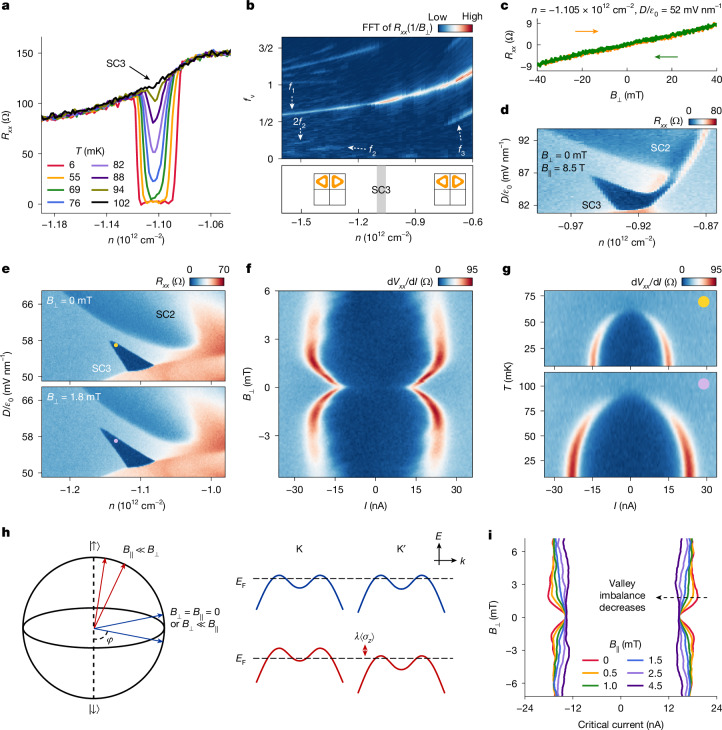
SC3 in R5G enhanced by *B*_⊥_. **a**, *R*_*xx*_ as a function of *n* at *D*/*ε*_0_ = 49.61 mV nm^−1^ and varied temperatures. The resistance vanishes as *T* decreases. **b**, Fast Fourier transform of *R*_*xx*_(1/*B*_⊥_) as a function of *n* and *f*_*ν*_, suggesting SC3 is developed from a half-metal with an annular-shaped Fermi surface. **c**, *R*_*xy*_ as a function of *B*_⊥_ in the forward and backward scanning directions in the normal state of SC3 (*T* = 160 mK). The absence of the anomalous Hall effect indicates zero valley polarization. **d**, *R*_*xx*_ map at *B*_⊥_ = 0 mT and *B*_∥_ = 8.5 mT. SC3 features ultrahigh PVR of >34. **e**, *R*_*xx*_ maps at *B*_⊥_ = 0 mT (top) and 1.8 mT (bottom). The SC3 region is enlarged by *B*_⊥_. **f**, Differential resistance as a function of *I* and *B*_⊥_ for *n* = −1.135 × 10^12 ^cm^−2^ and *D*/*ε*_0_ = 57 mV nm^−1^. *I*_c_ is increased by small *B*_⊥_. **g**, Differential resistance as a function of *I* and *T* for *n* = −1.135 × 10^12 ^cm^−2^ and *D*/*ε*_0_ = 57 mV nm^−1^ under *B*_⊥_ = 0 mT (top) and 1.8 mT (bottom). Both *I*_c_ and transition temperature increased with the small *B*_⊥_. Data in **e**–**g** show a rare enhancement of SC by *B*_⊥_. **h**, Description of a theoretical model. When *B*_⊥_ much larger than *B*_∥_ is applied, the spin vectors are steered along the *z*-axis, resulting in the valley-contrasting energy ±*λ*⟨*σ*_*z*_⟩/2. This induces the valley imbalance and enhanced valley DOS, which can strengthen intravalley pairing. **i**, Critical currents (extracted from peaks in d*V*_*xx*_/d*I* from Extended Data Fig. 9f–k) as functions of *B*_⊥_ for *B*_∥_ tuned around a few mT. The enhancement of SC3 by *B*_⊥_ gets weaker under *B*_∥_ of only a few mT. The data in **b**–**g** are from device R5G-1 and the data in **a**,**i** are from device R5G-2. FFT, fast Fourier transform.

Having SCs established in R5G and R4G, we investigate R5G under *B*_∥_. Figure 2a shows *R*_*xx*_ maps at *B*_∥_ = 0 and 8 T. We observe two differences comparing these maps: (1) SC2 is expanded in the *n–D* space at *B*_∥_ = 8 T; (2) a new low-resistance state indicated as SC4 is induced by high *B*_∥_. SC4 is well separated from SC2 and SC3 in the map, suggesting it is distinct from the superconductors existing at *B*_∥_ = 0 T. SC3 also survives at *B*_∥_ = 8 T and is eventually connected to SC2 at *B*_∥_ = 8.5 T in the other two devices (Fig. 3d and Extended Data Fig. 4g).

We now focus on the comparison of SC2 at *B*_∥_ = 0 and 8 T. First, the area of SC2 in the *n*–*D* space increased at *B*_∥_ = 8 T. This can be seen in the contour of SC2 at *B*_∥_ = 0 T, indicated as a white dashed curve, being smaller than SC2 at *B*_∥_ = 8 T. Second, *R*_*xx*_ dropped to lower values across the SC2 at *B*_∥_ = 8 T. Finally, *R*_*xx*_ starts to drop from the normal state at higher temperatures under *B*_∥_ = 8 T (Fig. 2b). Here, we compared the evolution of *R*_*xx*_ under varied temperatures at the (*n*, *D*) point featuring the minimal resistance at each field, because SC2 shifts to a different location in the *n*–*D* space by *B*_∥_. The (*n*, *D*) points where the data in Fig. 2b were taken are denoted by yellow and green circles in Fig. 2a.

The expansion of the phase in the *n*–*D* space, with the increase in the transition temperature and critical current *I*_c_ (Extended Data Fig. 1e) suggests that SC2 is enhanced by *B*_∥_. We performed fermiology analyses to understand its isospin structure. Figure 2c shows the fast Fourier transform of *R*_*xx*_(1/*B*_⊥_) as a function of *n* and *f*_*ν*_ (Methods). Two branches *f*_2_ and *f*_3_ obey the relation *f*_1_ − (*f*_2_ + *f*_3_) ≈ 1/2, and they are merged around the emergence of SC2. This suggests the normal state of SC2 is an annular half-metal. Combining this with the absence of anomalous Hall effect in its normal state (Fig. 2d), we conclude that SC2 arises from a valley-unpolarized annular half-metal parent state.

Contrary to SC2 existing at zero magnetic field, SC4 is observed only at high *B*_∥_. Figure 2e shows the *R*_*xx*_ map around SC4 at *B*_∥_ = 8.0 T and 8.835 T, featuring a stripe-shaped region with low resistance. The resistance decreases as applying higher *B*_∥_, which was limited by the magnet used. Figure 2f shows *R*_*xx*_ linecuts at varied temperatures with *V*_*xx*_–*I* characteristics. Nonlinear d*V*_*xx*_/d*I* is observed at the base temperature, and the reduction of *R*_*xx*_ disappears as temperature increases. Peaks in d*V*_*xx*_/d*I* disappear at *B*_⊥_ ≈ 6 mT.

The suppression of *R*_*xx*_, nonlinear d*V*_*xx*_/d*I* and responses to *B*_⊥_ for SC4 are consistent with SC phenomenology. Although the fragility of SC4 complicates detailed analyses, we may take 70 mK at which *R*_*xx*_ starts decreasing as a conservative estimate of the transition temperature. This gives the Pauli-limit violation ratio (PVR) of about 68 at the highest field available, when compared with a zero-field Bardeen–Cooper–Schrieffer superconductor with the same transition temperature.

## SC3 enhanced by *B*_⊥_

Figure 3a shows *R*_*xx*_ for SC3, featuring vanishing resistance at the base temperature and a sharp rise as *T* increases. Together with nonlinear d*V*_xx_/d*I* (Fig. 3f), this suggests that SC3 is a superconductor.

We perform fermiology analyses to understand its Fermi surface structure. Figure 3b shows the fast Fourier transform of *R*_*xx*_(1/*B*_⊥_) as a function of *n* and *f*_*ν*_. The branch *f*_1_ spans across the whole carrier density, and the low-frequency feature is broken into two segments, *f*_2_ and *f*_3_. Although the low-frequency branch in the density range of SC3 is unclear, *f*_2_ and *f*_3_ at its two ends follow relations *f*_1_ − *f*_2_ ≈ 1/2 and *f*_1_ − *f*_3_ ≈ 1/2, suggesting a half-metal with an annular Fermi surface. Figure 3c shows *R*_*xy*_ as *B*_⊥_ is swept back and forth in the normal state of SC3, showing no anomalous Hall effect. SC3 is still observable at *B*_∥_ = 8.5 T (Fig. 3d).

These observations suggest that SC3 is developed from a valley-unpolarized half-metal with annular Fermi surface. Especially, SC3 exceeds the Pauli limit (about 0.24 T) by a factor of >34 (see Extended Data Fig. 7j for the estimation of the Berezinskii–Kosterlitz–Thouless (BKT) transition temperature *T*_BKT_). This ultrahigh PVR supports the unconventional nature of SC3.

Figure 3e shows *R*_*xx*_ maps at *B*_⊥_ = 0 and 1.8 mT. Unlike SC2 and most other SCs, SC3 is expanded by *B*_⊥_. Figure 3f shows d*V*_*xx*_/d*I* as a function of *I* and *B*_⊥_. The critical current is doubled by *B*_⊥_ = 1.8 mT before decreasing at higher fields. *T*_BKT_ also increases from about 47 mK to 76 mK at this (*n*, *D*) point, as shown in comparison between maps at the two field values (Fig. 3g). Similar enhancements of *I*_c_ and *T*_BKT_ are observed across the SC3 region (Extended Data Fig. 7a–f) and disappear under nonzero *B*_∥_ (Extended Data Fig. 8a,b).

The enhancement of SC by small *B*_⊥_ adds more exoticness to SC3. As explained in the Methods, mechanisms based on spin or valley magnetism, or natural Josephson junctions with the phase difference of π (that is, 0–π junction) cannot explain this observation. We sketch one possible scenario that consistently explains all of our observations (see Methods for details). Under zero magnetic fields, the spin-polarized and valley-unpolarized normal state possesses the spin vectors canted with the angle *φ* ≈ 90° (ref. ^33^) (Fig. 3h), because Hund’s coupling *E*_H_ (about 2 meV) is much stronger than the intrinsic SOC *λ* of graphene^41,42^ (approximately 50 μeV). For nonzero *B*_⊥_ with *B*_∥_ = 0, the spin vectors can be steered along the *z*-axis even when the spin Zeeman energy (about 0.2 μeV for 1.8 mT) is much smaller than *λ*, because of the interplay with Hund’s coupling. The nonzero spin component ⟨*σ*_*z*_⟩ leads to the valley-contrasting energy ±*λ*⟨*σ*_*z*_⟩/2 and the valley imbalance in the hole occupation, as described in Fig. 3h. The detailed model calculations are presented in the Methods and Extended Data Fig. 9. This will result in an increase in the density of states (DOS) for one valley and a decrease for the other valley. Assuming intravalley pairing dominates SC3, the increased DOS for one valley can strengthen the superconductivity.

Transport measurements under carefully adjusted *B*_⊥_ and *B*_∥_ provide further evidence for the proposed scenario, although more experiments are required to fully test it. The spin vectors aligned to the *z*-axis by *B*_⊥_ will tend to be steered back to their easy plane (*φ* = 90°) when *B*_∥_ comparable to *B*_⊥_ is applied. As *B*_⊥_ of approximately 1.8 mT maximizes the strength of SC3, *B*_∥_ around the same value will weaken the enhancement. This hypothesis is confirmed in Fig. 3i, in which the maximum of *I*_c_ at *B*_⊥_ ≈ 1.8 mT is suppressed by *B*_∥_ of merely a few mT. Moreover, when stronger SOC is imposed, the spin vectors will be pinned more robustly and accordingly the same *B*_⊥_ will induce a smaller spin polarization and valley imbalance. This suggests the enhancement of SC3 is suppressed when strong SOC is proximitized to R5G. This prediction is consistent with the absence of the increase in *I*_c_ by *B*_⊥_ for SC3 in R5G/WS_2_ (Extended Data Fig. 9o).

## New SCs induced by SOC

**Fig. 4:**
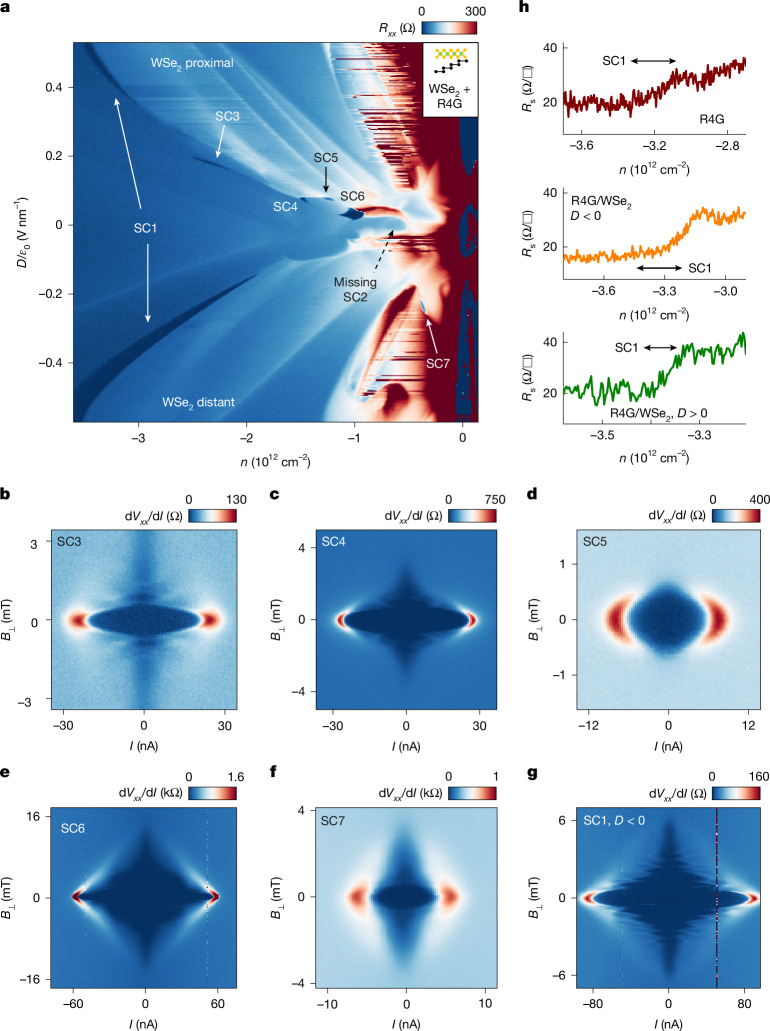
Proliferation of SC in R4G by proximitized SOC effects. **a**, *R*_*xx*_ map of a R4G/WSe_2_ device. Five new states (SC3–7) emerge while SC2 is suppressed. The names of SCs follow the convention of Fig. 1b. **b**–**g**, Differential resistance as a function of *I* and *B*_⊥_ for SC3 (*n* = −2.356 × 10^12 ^cm^−2^, *D*/*ε*_0_ = 0.18 V nm^−1^), SC4 (*n* = −1.482 × 10^12 ^cm^−2^, *D*/*ε*_0_ = 0.072 V nm^−1^), SC5 (*n* = −1.242 × 10^12 ^cm^−2^, *D*/*ε*_0_ = 0.072 V nm^−1^), SC6 (*n* = −1.00 × 10^12 ^cm^−2^, *D*/*ε*_0_ = 0.026 V nm^−1^), SC7 (*n* = −0.38 × 10^12 ^cm^−2^, *D*/*ε*_0_ = − 0.24 V nm^−1^) and SC1 (*n* = −3.25 × 10^12 ^cm^−2^, *D*/*ε*_0_ = − 0.42 V nm^−1^). Vanishing *R*_*xx*_, nonlinear d*V*_*xx*_/d*I* (Extended Data Fig. 10) and their suppression by *B*_⊥_ suggest SC3–7 are superconductors. **h**, Square resistance as a function of *n* in the normal state of the SC1 of bare R4G (top, *D*/*ε*_0_ = 0.42 V nm^−1^), R4G/WSe_2_ at negative *D* (middle, *D*/*ε*_0_ = − 0.42 V nm^−1^) and R4G/WSe_2_ at positive *D* (bottom, *D*/*ε*_0_ = 0.469 V nm^−1^). Little change in *R*_s_ implies the preserved high quality of graphene with WSe_2_ at proximity.

Finally, we examine the impact of proximitized SOC on SC in rhombohedral graphene. Figure 4a shows the *R*_*xx*_ map of device R4G/WSe_2_. Compared with bare R4G (Fig. 1b), we observe additional states with vanishing *R*_*xx*_ labelled as SC3–7. Figure 4b–g shows d*V*_*xx*_/d*I* as a function of *I* and *B*_⊥_ for these states. For each SC, d*V*_*xx*_/d*I* vanishes at small *I* and exhibits peaks at zero *B*_⊥_. These features are suppressed as *B*_⊥_ increases. Extended Data Fig. 10 shows temperature-dependent *R*_*xx*_ and d*V*_*xx*_/d*I*, which are aligned with SC phenomenology.

These observations indicate SC3–7 are superconductors. Phenomenologically, the introduction of SOC proliferates regions with different isospin symmetries and renders more SCs at their phase boundaries. Simultaneously, SC1 is weakened when the holes are polarized to the WSe_2_-proximal side of R4G, evidenced by decreases in *T*_c_, *I*_c_ and *B*_c,⊥_ (Extended Data Fig. 10). Together with SC2 missing in R4G/WSe_2_, the diverse impacts of SOC on SC in R4G are similar to observations in R3G^34^.

We note that engineering SC states by WSe_2_ proximity keeps the high quality of bare graphene. Figure 4h compares normal-state resistance for SC1 in bare R4G, the WSe_2_-distant and WSe_2_-proximal side of R4G/WSe_2_ at comparable *D* fields. Although the strength of SC1 varies, all the normal-state square resistance is about 25 Ω/□ for the hole density where SC1 emerges. This suggests that the mean free path and the cleanliness of graphene are mostly unaffected by the WSe_2_ layer.

## Discussions and outlook

In summary, we observed lots of superconductors in R4G and R5G, featuring three unconventional ones (SC2–4 in R5G) with unusual responses to magnetic fields. Especially, their remarkably high PVRs put them among superconductors with the highest PVR value reported^2,43^. These new SCs show several distinct characteristics compared with SCs in the literature. First, in-plane critical field exceeding the Pauli limit has been reported in transition-metal dichalcogenides (TMDs)^43–46^ and SOC-proximitized graphene^31,37^. There, strong Ising SOC pins the electron spins to the out-of-plane direction and makes the SC robust against *B*_∥_. However, these Ising SCs are killed by high *B*_∥_ before being fully spin-polarized. By contrast, SC2–4 are robust against *B*_∥_ with ultrahigh PVR and arise from half-metals. As the intrinsic SOC of graphene is much weaker than Hund’s coupling^33,41,42^ favouring the spin polarization of half-metals, this suggests SC2–4 are spin-polarized differently from Ising SC. Second, notable PVR has been reported in twisted trilayer graphene^21^ and R3G^32^ without proximitized SOC. However, their demonstrated PVRs are smaller than the lower bound of SC3 in R5G that is not even enhanced by *B*_∥_. Third, field-enhanced and field-induced SCs have been observed in uranium-based compounds^47,48^, organic superconductors^49,50^ and Eu_*x*_Sn_1−*x*_Mo_6_S_8_ (ref. ^51^). Spin fluctuations or the Jaccarino–Peter effect^52^ has been proposed to explain these phenomena. However, bare graphene lacks magnetic elements and the aforementioned mechanisms cannot be directly applied. Considering the alteration of adjacent states under a high in-plane field, we propose that change in the normal states by *B*_∥_ may enhance SC2 and induce SC4 (Methods). This might also be the case for SC in R2G^6^ appearing only under nonzero *B*_∥_. Further efforts are necessary to fully understand these field-enhanced and field-induced SCs.

Rhombohedral multilayer graphene also provides a platform for further engineering SCs. A promising direction is proximations with QAH states to engineer non-Abelian quasiparticles^7,39,40^ in a lateral junction. QAH states with a wide range of Chern numbers and fractional QAH states have been discovered in rhombohedral graphene neighboured by TMDs^8^ or with moiré effects^36–38^. The coexistence of SCs and topological states alleviates complexities in combining them from different materials. Moreover, many superconductors in our work can be reached by low gate electric fields, compared with other graphene SCs^6,30,31,53,54^. This reduces the risk of gate leakage when accessing these SCs, which will facilitate realizing non-Abelian anyons and topological quantum computations.

## Methods

### Device fabrications

Rhombohedral graphene and hexagonal boron nitride (hBN) flakes were prepared onto SiO_2_/Si substrates by mechanical exfoliation. The rhombohedral domains of graphene were identified and confirmed with an infrared camera^55^, near-field infrared nanoscopy^56^ and Raman spectroscopy^57^, and subsequently isolated by cutting with a femtosecond laser. Van der Waals heterostructures were made following a dry transfer procedure. We picked up the top hBN, graphite, middle hBN and graphene using poly(bisphenol A carbonate) film on polydimethylsiloxane, and landed it on a prepared bottom stack consisting of an hBN and graphite bottom gate. The device was then etched into a multi-terminal structure using electron-beam lithography and reactive ion etching. Cr/Au was thermally evaporated for electrical connections to the source, drain and gate electrodes.

### Electrical transport measurements

The devices were measured in either a Bluefors LD250 dilution refrigerator at MIT or a Leiden MNK126-700 dilution refrigerator at the University of Basel. In the setup at MIT, the d.c. and a.c. currents were generated by Keysight 33210A function generator with an a.c. frequency of 17.77 Hz. Stanford Research Systems SR830 lock-in amplifiers and Basel Precision Instruments (BASPI) SP1004 voltage preamplifiers were used to measure the longitudinal and Hall resistance *R*_*xx*_ and *R*_*xy*_. Keithley 2400 source meters were used to apply top- and bottom-gate voltages, *V*_TG_ and *V*_BG_. Twisted phosphorus bronze wires or thermocoax cables thermalized at each stage of the fridge went through two thermal meanders (one on the mixing chamber plate and the other on the cold finger), ceramic radiofrequency filters (DigiKey), four-stage RC filters, and silver epoxy filters (MFT25 from BASPI and a custom-made one) to cool down electrons. In the setup at the University of Basel, MFLI Zurich Instrument lock-in amplifiers were used. The same lock-in amplifier generated the a.c. current, whereas the d.c. current was provided by a BASPI DAC SP927. Moreover, the drain–source current was pre-amplified to a voltage signal by an SP983c *I* to *V* converter. Thermocoax cables thermalized at each stage of the fridge were used with two sets of silver epoxy filter^58^ (one on the mixing chamber plate and the other right next to the sample holder). Keithley 2400 or BASPI DAC SP927 was used to apply the gate voltages. The applied *V*_TG_ and *V*_BG_ were swept to adjust carrier density *n* = (*C*_T_*V*_TG_ + *C*_B_*V*_BG_)/*e* and displacement field *D* = (*C*_T_*V*_TG_ − *C*_B_*V*_BG_)/2, where *C*_T_ and *C*_B_ are top-gate and bottom-gate capacitance per unit area obtained from the Landau fan diagram. The longitudinal resistance *R*_*xx*_ is defined as four-terminal differential resistance d*V*_*xx*_/d*I* at zero d.c. current.

### Shubnikov–de Haas oscillations and fermiology analyses

Shubnikov–de Haas oscillations were obtained by measuring *R*_*xx*_ as a function of *B*_⊥_ at the base temperature. For fermiology analyses, *R*_*xx*_ was Fourier-transformed as a function of 1/*B*_⊥_. The Fourier-transformed data are a function of the frequency *f*_1/*B*_. Then, *f*_1/*B*_ is normalized by the frequency that corresponds to the full carrier density as *f*_ν_ = *f*_1/*B*_/(|*n*|*h*/*e*).

### Extraction of coherence length and mean free path

The coherence length *ξ* of a superconductor and the mean free path *l* of its normal state are extracted as *ξ* $=\sqrt{\frac{h/(2e)}{2\pi {B}_{c,\perp }}}$ and $l\cong \frac{h}{{e}^{2}}\frac{1}{4{k}_{F}{R}_{s}}$, respectively. Here, *B*_c,⊥_ is the out-of-plane critical field, ${R}_{s}={R}_{n}W/L$ is the square resistance of normal-state resistance *R*_*n*_, ${k}_{{\rm{F}}}\approx \sqrt{\pi n}$ is the Fermi wavevector, *L* and *W* are the length and width of the sample, *h* is Planck’s constant and *e* is the elementary charge^32^.

### Measurements under high *B*_∥_

Devices R5G-1, R5G-2 and R5G-3 were measured in the fridges at MIT and the University of Basel, and SC1-3 appearing at zero magnetic fields were observed in both of the fridges. The fridge at the University of Basel is equipped with a Cryomagnetics two-axis magnet providing the magnetic fields both in-plane and perpendicular to the sample. Here, the quench boundaries limited the highest available fields. As the perpendicular component of the in-plane field due to a sample misalignment could be compensated with the perpendicular magnet using the sample as a magnetometer, we obtained the data for R5G under *B*_∥_ from the fridge at the University of Basel. The data for R4G/WSe_2_ under *B*_∥_ (Supplementary Fig. 1) were obtained from the fridge at MIT using a three-axis superconducting magnet (American Magnetics).

### Fraunhofer-like interference patterns and nonzero residual resistance

**Extended Data Fig. 3:**
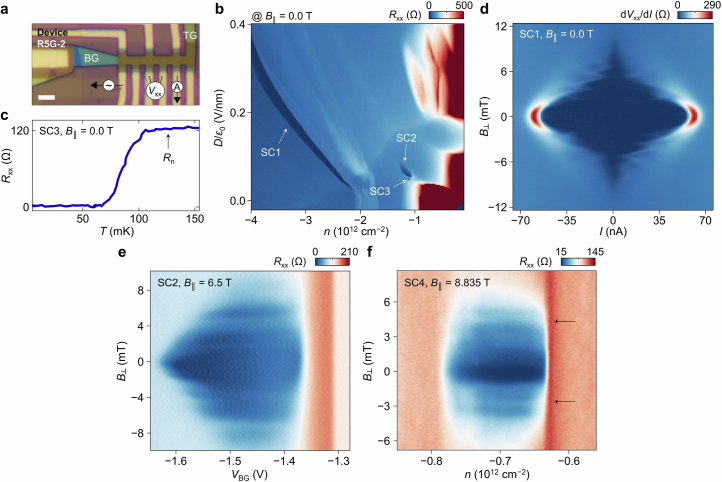
Reproducibility of SC in R5G from Device R5G-2. **a**, Optical image of Device R5G-2 with a representative measurement configuration. Scale bar: 3 μm. **b**, *R*_xx_ as a function of *n* and *D*/*ε*_0_. All three SCs in R5G at zero magnetic fields are reproduced. **c**, Temperature-dependent *R*_xx_ of SC3 (*n* = −1.100 × 10^12 ^cm^−2^, *D*/*ε*_0_ = 49.61 mV/nm). SC3 approaches 0 Ω below the transition temperature. **d**, Differential resistance as a function of *I* and *B*_⊥_ for SC1 (*n* = −3.38 × 10^12 ^cm^−2^, *D*/*ε*_0_ = 0.214 V/nm). Clear interference patterns are observed. **e,f**, *R*_xx_ under varied *B*_⊥_ for SC2 (*V*_TG_ = − 0.2 V, **e**) and SC4 (*D*/*ε*_0_ = 140.1 mV/nm, **f**). Modulations of SC2 and SC4 by *B*_⊥_ were observed, demonstrating Fraunhofer-like interferences.

**Extended Data Fig. 4:**
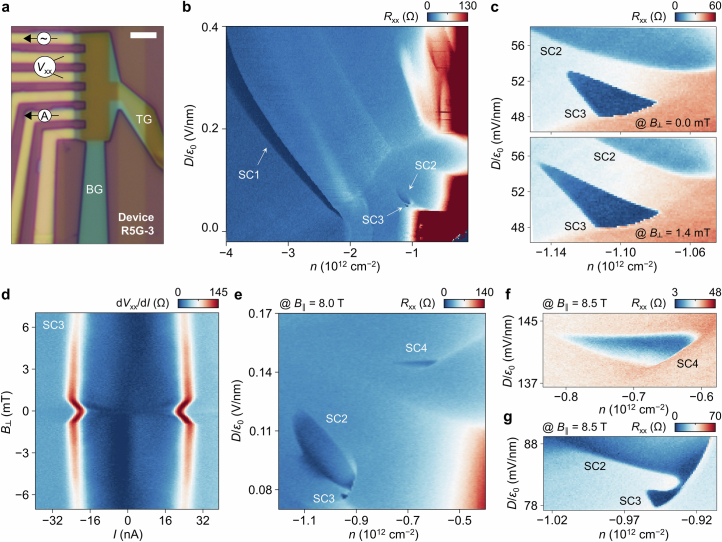
Reproducibility of SC in R5G from Device R5G-3. **a**, Optical image of Device R5G-3 with a representative measurement configuration. Scale bar: 3 μm. **b**, *R*_xx_ as a function of *n* and *D*/*ε*_0_. All three SCs in R5G at zero magnetic fields are reproduced. **c**, *R*_xx_ as a function of *n* and *D*/*ε*_0_ taken at *B*_⊥_ = 0.0 (top) and 1.4 mT (bottom). Area in the *n*-*D* space occupied by SC3 is expanded by a nonzero *B*_⊥_, which is the opposite from the case of SC2. **d**, Differential resistance as a function of *I* and *B*_⊥_ for SC3 (*n* = −1.11 × 10^12 ^cm^−2^, *D*/*ε*_0_ = 50.6 mV/nm), showing the increase in the critical current by small *B*_⊥_. **e**, *R*_xx_ as a function of *n* and *D*/*ε*_0_ taken at *B*_║_ = 8.0 T. SC4 that did not appear at zero field is observed. **f**, Zoomed-in view of *R*_xx_ around SC4 at *B*_║_ = 8.5 T. **g**, Zoomed-in view of *R*_xx_ around SC3 at *B*_║_ = 8.5 T. SC3 is connected to SC2 in the *n*-*D* space.

**Extended Data Fig. 5:**
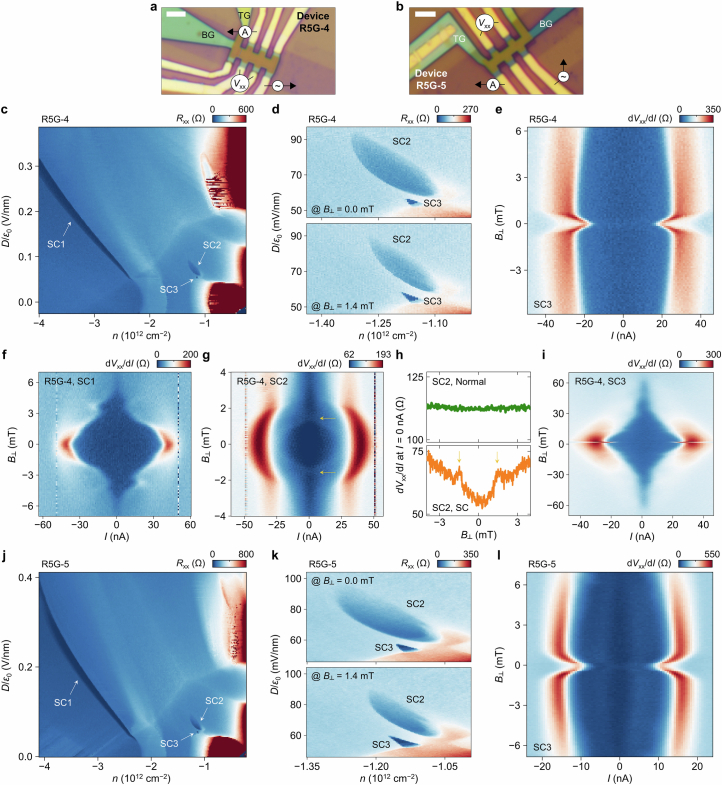
Reproducibility of SC in R5G from Device R5G-4 and R5G-5. **a,b**, Optical image of Device R5G-4 (**a**) and R5G-5 (**b**) with representative measurement configurations. Scale bar: 3 μm. **c**, *R*_xx_ as a function of *n* and *D*/*ε*_0_ for Device R5G-4. All three SCs in Fig. 1a are reproduced. **d**, *R*_xx_ as a function of *n* and *D*/*ε*_0_ focusing on SC2 and SC3 at *B*_⊥_ = 0.0 (top) and 1.4 mT (bottom). Area in the *n*-*D* space occupied by SC3 increased by a nonzero *B*_⊥_. **e**, Differential resistance as a function of *I* and *B*_⊥_ for SC3 (*n* = −1.16 × 10^12 ^cm^−2^, *D*/*ε*_0_ = 55 mV/nm), showing the increase of critical current by small *B*_⊥_. **f-i**, Differential resistance as a function of *I* and *B*_⊥_ for SC1 (*n* = −3.57 × 10^12 ^cm^−2^, *D*/*ε*_0_ = 0.225 V/nm) (**f**), SC2 (*n* = −1.17 × 10^12 ^cm^−2^, *D*/*ε*_0_ = 67 mV/nm) (**g**) and SC3 (*n* = −1.16 × 10^12 ^cm^−2^, *D*/*ε*_0_ = 55 mV/nm) (**i**) from Device R5G-4. The differential resistance at zero DC current for SC2 in its normal state (top) and superconducting state (bottom) is plotted in **h**. The modulation of d*V*_xx_/d*I* by *B*_⊥_ are observed in SC1-SC3. This modulation in SC2 is highlighted with yellow arrows in **g** and **h**. **j**, *R*_xx_ as a function of *n* and *D*/*ε*_0_ for Device R5G-5. All three SCs in Fig. 1a are reproduced. **k**, *R*_xx_ as a function of *n* and *D*/*ε*_0_ focusing on SC2 and SC3 at *B*_⊥_ = 0.0 (top) and 1.4 mT (bottom). Area in the *n*-*D* space occupied by SC3 increased by a nonzero *B*_⊥_. **l**, Differential resistance as a function of *I* and *B*_⊥_ for SC3 (*n* = −1.143 × 10^12 ^cm^−2^, *D*/*ε*_0_ = 55.9 mV/nm), showing the increase of critical current by small *B*_⊥_.

**Extended Data Fig. 6:**
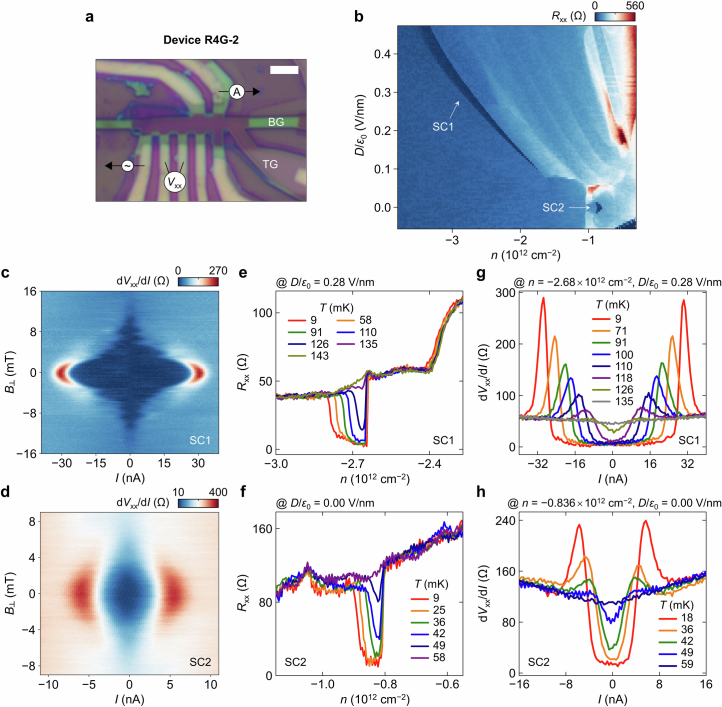
Reproducibility of SC in R4G from Device R4G-2. **a**, Optical image of Device R4G-2 with a representative measurement configuration. Scale bar: 3 μm. **b**, *R*_xx_ as a function of *n* and *D*/*ε*_0_. Two SCs in Fig. 1b in the main text are reproduced. **c,d**, Differential resistance as a function of *I* and *B*_⊥_ for SC1 (*n* = −2.68 × 10^12 ^cm^−2^, *D*/*ε*_0_ = 0.28 V/nm) (**c**) and SC2 (*n* = −0.836 × 10^12 ^cm^−2^, *D*/*ε*_0_ = 0.00 V/nm) (**d**). SC1 shows a clear Fraunhofer-like pattern. **e,f**, *R*_xx_ as a function of *n* at varied temperatures and fixed *D*/*ε*_0_ for SC1 (**e**) and SC2 (**f**). **g,h**, Differential resistance d*V*_xx_/d*I* as a function of *I* at varied temperatures for SC1 (*n* = −2.68 × 10^12 ^cm^−2^, *D*/*ε*_0_ = 0.28 V/nm, **g**) and SC2 (*n* = −0.836 × 10^12 ^cm^−2^, *D*/*ε*_0_ = 0.00 V/nm, **h**). Vanishing *R*_xx_, nonlinear d*V*_xx_/d*I*, and the modulation of critical current by *B*_⊥_ suggest these are superconducting states.

**Extended Data Fig. 7:**
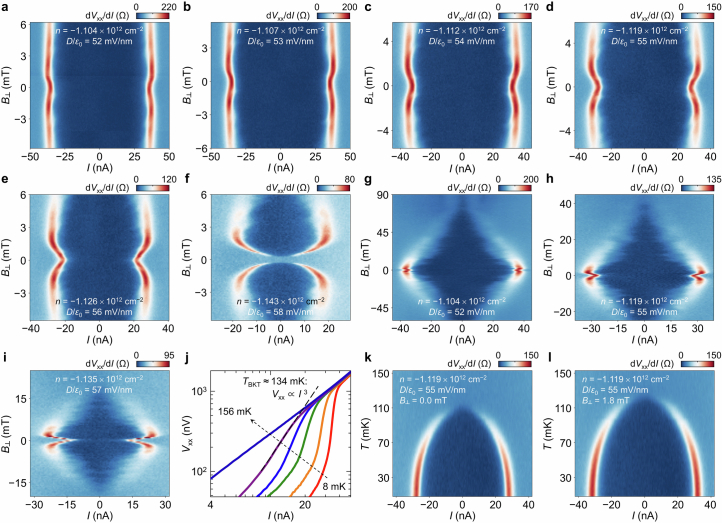
*T*- and *B*_⊥_-dependent measurements on SC3 in Device R5G-1. **a**-**f**, Differential resistance as a function of *I* and *B*_⊥_ at different (*n*, *D*) points. The enhancement of SC3 by *B*_⊥_ is observed globally across its phase space. **g**-**i**, Differential resistance as a function of *I* and *B*_⊥_ at different (*n*, *D*) points for a wider range of the magnetic field. Fraunhofer-like patterns arising from quantum interference are observed in **i** which is adjacent to the phase boundary of SC3, and they become fainter as *D*/*ε*_0_ decreases. **j**, *V*_xx_ versus current *I* at *T* = 8, 88, 115, 126, 134 and 156 mK for *n* = −1.104 × 10^12 ^cm^−2^ and *D*/*ε*_0_ = 52 mV/nm, where SC3 was the strongest in this device. The dashed line is the prediction from the BKT theory, giving *T*_BKT_ ≈ 134 mK. **k,l**, Differential resistance as a function of *I* and *T* at *n* = −1.119 × 10^12 ^cm^−2^ and *D*/*ε*_0_ = 55 mV/nm under *B*_⊥_ = 0 and 1.8 mT. *T*_BKT_ increases from 99 to 110 mK by applying *B*_⊥_ of 1.8 mT, which demonstrates the increase in *T*_BKT_ happens globally.

The residual resistance relative to the normal-state resistance in some of the superconducting states, such as SC2 and SC4 in R5G, is relatively large compared with the others. The ratios of residual resistance to normal-state resistance for superconductors reported in this work are presented in Supplementary Table 1. This nonzero residual resistance has been observed in several graphene SCs^30–32,59^. Fraunhofer-like interference patterns that arise from phase-coherent transport can serve as additional evidence for SC. There can be non-SC islands in a μm-size device because of sample inhomogeneity, even when (*n*, *D*) are tuned to a superconducting phase. Phase-coherent charge transport is affected by the Aharonov–Bohm phase, and thus the interference pattern appears. We found these interference patterns and periodic modulations of SC by *B*_⊥_ in most of the superconducting states reported here: SC1 (Extended Data Fig. 6c) in R4G; SC1 (Extended Data Figs. 3d and 5f), SC2 (Fig. 2b, Extended Data Figs. 3e and 5g,h), SC3 (Extended Data Figs. 5i and 7i) and SC4 (Fig. 2f and Extended Data Fig. 3f) in R5G; SC1 (Fig. 4g), SC3 (Fig. 4b), SC4 (Fig. 4c) and SC6 (Fig. 4e) in R4G/WSe_2_. We note that a limited number of crystalline rhombohedral graphene devices^32,33^ have demonstrated Fraunhofer-like patterns in their superconducting states. This is in contrast to moiré graphene devices^17–20,60,61^ in which Fraunhofer-like patterns could be observed more universally and were more pronounced. Possible reasons are (1) less inhomogeneity in a crystalline graphene system and (2) a lower out-of-plane critical field that kills SC even before the interference pattern is developed. For example, SC1 at negative *D* in R4G/WSe_2_ shows the first side-peak at *B*_⊥_ ≈ 1.1 mT (Fig. 4g). However, SC5 (Fig. 4d) and SC1 at positive *D* (Extended Data Fig. 10s) are already killed at this value of *B*_⊥_ and the interference patterns do not appear.

### Ruling out possible mechanisms for the enhancement of SC3 by *B*_⊥_

**Extended Data Fig. 8:**
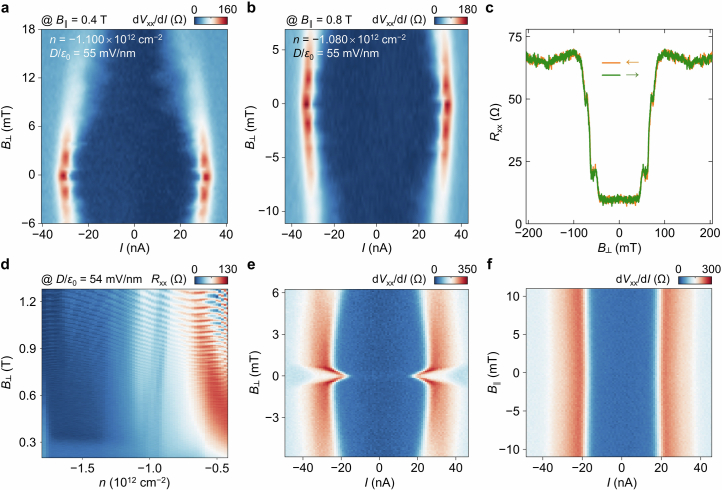
Additional data for SC3 in R5G. **a,b**, Differential resistance as a function of *I* and *B*_⊥_ for *B*_║_ = 0.4 T (**a**) and *B*_║_ = 0.8 T (**b**). *B*_⊥_-induced enhancement for SC3 disappeared under a high enough in-plane field. **c**, *R*_xx_ as a function of *B*_⊥_ in the forward and backward scanning directions for SC3 (*n* = −1.132 × 10^12 ^cm^−2^, *D*/*ε*_0_ = 54 mV/nm) at the base temperature. There is no hysteretic behavior observed, implying the absence of orbital magnetism in the superconducting state of SC3. **d**, Landau fan diagrams taken at *D*/*ε*_0_ = 54 mV/nm as a function of *n* covering SC3. This data was used for the Fourier transform in Fig. 3b. **e,f**, Dependence of the critical current of SC3 on small *B*_⊥_ and *B*_║_. The critical current *I*_c_ increases with showing maxima only when the out-of-plane field is applied. These data were measured for *n* = −1.16 × 10^12 ^cm^−2^ and *D*/*ε*_0_ = 55 mV/nm.

In summary, SC3 is enhanced by a small out-of-plane magnetic field when *B*_∥_ is zero. Similar values of *B*_∥_ do not enhance the SC3. The enhancement by *B*_⊥_ also disappears in the presence of nonzero *B*_∥_. Here, we discuss several scenarios that cannot fully explain these observations. First, *B*_⊥_ can couple to orbital ferromagnetic moments and lower the energy of the SC state compared with its competing states. This is the case for chiral SC developed at high displacement fields^53^. However, unlike chiral SC, the absence of magnetic hysteresis in the normal (Fig. 3c) and superconducting (Extended Data Fig. 8c) states of SC3 suggests the vanishing orbital ferromagnetism. Second, even without a spontaneous valley polarization, the valley-contrasted orbital magnetization can be generated by finite *B*_⊥_ through the valley Zeeman effect and induce the valley polarization. The DOS in one of the valleys may increase, and thus SC3 is enhanced. This is, however, contradictory to the disappearance of the enhancement under nonzero *B*_∥_, because it is unlikely that the in-plane field affects the valleys. Third, spin Zeeman energy can shift the Fermi level to a van Hove singularity whose enhanced DOS can develop stronger SC. However, the absence of the increase in *I*_c_ under *B*_∥_ (Extended Data Fig. 8f) rules out this scenario. Finally, for a percolative superconductor, natural junctions formed by superconducting and normal-state islands in a sample can lead to an increase in *I*_c_ under tiny *B*_⊥_. This occurs when a π phase difference across the junction, which is called a 0–π junction, is compensated by the *B*_⊥_-induced Aharonov–Bohm phase. This phenomenon has been observed in engineered Josephson junctions^62–65^. The anomalous SC phenomenology under *B*_⊥_ observed in some twisted bilayer graphene devices^18,60^ and twisted MoTe_2_ (tMoTe_2_) nanojunctions^66^ has also been understood as coming from a 0–π junction. For the tMoTe_2_ nanojunctions, it was proposed that both spin-singlet and spin-triplet condensates exist across the Pd_7_MoTe_2_–tMoTe_2_–Pd_7_MoTe_2_ junction (where Pd_7_MoTe_2_ is a superconductor without anomalous behaviour), because tMoTe_2_ breaks the inversion symmetry. Provided these two condensates have a phase difference of π, a 0–π junction is formed across the nanojunction and the SC transport phenomena may be enhanced by applying *B*_⊥_. Regarding SC3 in R5G, if there are 0–π junctions in the devices due to sample inhomogeneity, the optimal *B*_⊥_ value resulting in the strongest enhancement will notably depend on (*n*, *D*). This is because the normal-state area connecting two superconducting regions in the junction will expand as the SC gets weaker. However, the value of *B*_⊥_ showing the strongest SC3 is independent of (*n*, *D*) in our data (Extended Data Fig. 7a–f).

### Proposed mechanism for the enhancement of SC3 by *B*_⊥_ based on SOC

Here, we provide a physical picture compatible with all our observations on SC3 in R5G. The normal state of SC3 is a spin-polarized and valley-unpolarized half-metal. Owing to the intrinsic SOC strength *λ* much smaller than the Hund’s coupling *E*_H_ in bare R5G, the spins from the two valleys are largely polarized in-plane with a small canting to the out-of-plane direction^33,67^. When a large-enough *B*_⊥_ is applied and the spins are polarized out of plane, the SOC projected to one layer of R5G (suggested by the fermiology analysis), ${H}_{\mathrm{SOC}}=-\frac{\lambda }{2}{\tau }_{z}{\sigma }_{z}$, where *τ*_*z*_ is the valley, and *σ*_*z*_ is the *z*-component of the spin, induces a valley-contrasting shift in the Fermi energy. Then, the DOS of one valley increases, whereas that of the other valley decreases. Assuming the dominant pairing mechanism is intravalley and the two valleys are mostly decoupled^68^ owing to the large momentum difference between them, one of the intravalley pairing channels can be enhanced by the increased DOS^69^. As the global transport of a superconductor is dominated by the stronger pairing channel, SC can be enhanced by *B*_⊥_. The SOC-induced shift in the Fermi energy is weaker when the spin is mostly aligned in-plane by *B*_∥_, and thus the enhancement by *B*_⊥_ is not observed.

We provide calculations supporting the aforementioned scenario. We consider the free energy density *F* (refs. ^33,67,70^):

$$\begin{array}{c}F=\frac{\kappa }{2}({n}_{+}^{2}+{n}_{-}^{2})-{J}_{{\rm{H}}}{{\bf{n}}}_{+}{\boldsymbol{\cdot }}{{\bf{n}}}_{{\boldsymbol{-}}}-\frac{\lambda }{2}({n}_{+}^{z}-{n}_{-}^{z})\\ \,-\,{h}_{\perp }({n}_{+}^{z}+{n}_{-}^{z})-{h}_{\Vert }({n}_{+}^{x}+{n}_{-}^{x})-{h}_{V}({n}_{+}-{n}_{-}).\end{array}$$

Here, **n**_+_ and **n**_−_ describe the total spin polarization vectors whose magnitude is the hole density and direction corresponds to the spin direction for each valley (+ for K, − for K′). The first term features the largest energy scale from the band dispersion and long-range Coulomb interaction. The second term describes Hund’s coupling, where *J*_H_ ≈ 2 meV/(10^12 ^cm^−2^) (ref. ^33^). The third term accounts for the SOC of the system. For bare R5G, this term corresponds to the intrinsic Kane–Mele SOC projected to one layer of the graphene. We take *λ* = 50 μeV for bare R5G^33,41,42,71^. When the SOC is proximitized to graphene by TMD, *λ* is of the order of 1 meV depending on the relative angle between graphene and TMD^31^. The fourth and fifth terms are the spin Zeeman energy for *B*_⊥_ and *B*_∥_, respectively. Here, *h*_⊥_ = *g*_S_*μ*_B_*B*_⊥_ and *h*_∥_ = *g*_S_*μ*_B_*B*_∥_ with the spin *g*-factor *g*_S_ = 2 and Bohr magneton *μ*_B_. The final term accounts for the valley Zeeman effect, where *h*_*V*_ = *g*_*V*_*μ*_B_*B*_⊥_. The two remaining parameters, *κ* and *g*_*V*_, are affected by the band structure. We used a 10-band Slonczewski–Weiss–McClure-type tight-binding model and calculated the average energy per hole ⟨*E*_h_⟩ and *g*_*V*_. We assumed a half-metal state with *n* = −1.1 × 10^12 ^cm^−2^, *D*/*ε*_0_ = 50 mV nm^−1^, which corresponds to where the SC3 was observed. Two sets of tight-binding parameters taken from refs. ^53^^,^^72^ gave consistent results of ⟨*E*_h_⟩ ≈ 1.4 meV and *g*_*V*_ ≈ 6. The value of ⟨*E*_h_⟩ is used to estimate *κ* as $\frac{\kappa }{2}({n}_{0}^{2}+{n}_{0}^{2})=\frac{\kappa {n}^{2}}{4}=\langle {E}_{{\rm{h}}}\rangle |n|$ and thus $\kappa =4\langle {E}_{{\rm{h}}}\rangle /|n|$ ≈ 5 meV/(10^12 ^cm^−2^).

We can calculate the spin canting angle (Fig. 3h) and the valley imbalance of hole occupations by minimizing *F* as a function of **n**_±_. We write the spin polarization vectors as

$${{\bf{n}}}_{+}=({n}_{0}+\delta )(\sin {\theta }_{+},0,\cos {\theta }_{+}),\,{{\bf{n}}}_{-}=({n}_{0}-\delta )(\sin {\theta }_{-},0,\cos {\theta }_{-}).$$

Here, *θ*_+_ and *θ*_−_ are the spin canting angles and *δ* is the valley imbalance. The total hole density is |*n*| = 2*n*_0_. For zero magnetic fields, the solution is ${\theta }_{+}=\pi -{\theta }_{-}={\cos }^{-1}\left(\frac{\lambda }{2{J}_{{\rm{H}}}|n|}\right)$ and *δ* = 0 (ref. ^33^). When external magnetic fields are applied, we cannot assume *θ*_+_ = π − *θ*_−_ and *δ* = 0, but can numerically find the solution.

**Extended Data Fig. 9:**
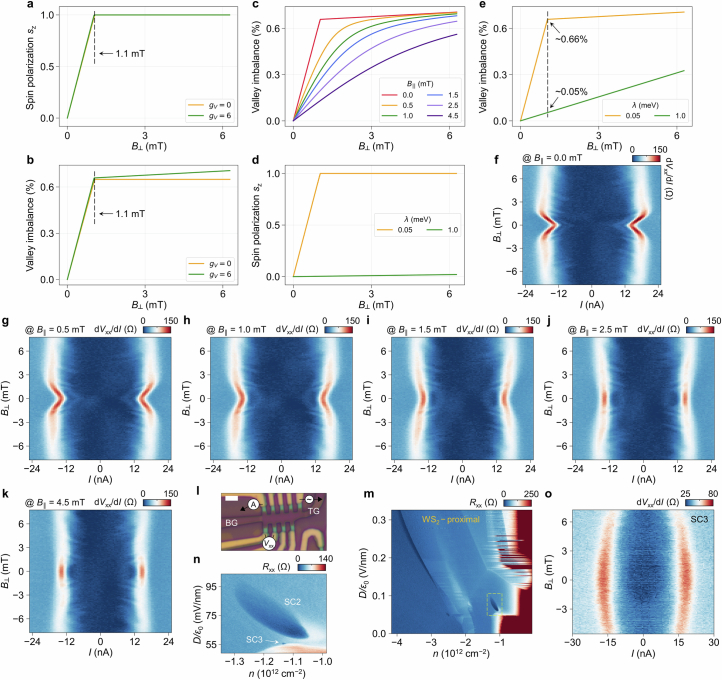
Calculations for the enhancement of SC3 and supporting experimental results. **a,b**, Spin polarization *s*_z_ (**a**) and valley imbalance normalized by *n*_0_ (**b**) as functions of a perpendicular magnetic field for bare R5G with *g*_*V*_ = 0 and 6. The optimal value of *B*_⊥_, calculated to be 1.1 mT, is mostly independent of *g*_*V*_. **c**, Valley imbalance normalized by *n*_0_ under varied *B*_║_ as functions of *B*_⊥_. The increase in the valley imbalance by *B*_⊥_ gets slower by applying *B*_║_, which is consistent with the experimental observations. **d,e**, Effect of the proximitized SOC on the spin polarization (**d**) and valley imbalance (**e**). When the SOC is strong, the valley imbalance is reduced by an order of magnitude and the cusp in the valley imbalance at optimal *B*_⊥_ disappears. **f-k**, Differential resistance as a function of *I* and *B*_⊥_ in SC3 from Device R5G-3 at *B*_║_ = 0.0 (**f**), 0.5 (**g**), 1.0 (**h**), 1.5 (**i**), 2.5 (**j**) and 4.5 (**k**) mT. **l**, Optical image of the Device R5G/WS_2_ and the representative measurement configuration. **m**, *R*_xx_ map of the device at the WS_2_-proximal side. **n**, *R*_xx_ map in the yellow dashed box in **m**. A weak SC3 is observed. **o**, Differential resistance as a function of *I* and *B*_⊥_ for SC3 (*n* = −1.133 × 10^12 ^cm^−2^, *D*/*ε*_0_ = 55.7 mV/nm). No enhancement by *B*_⊥_ was observed from SC3 in R5G/WS_2_.

**Extended Data Fig. 10:**
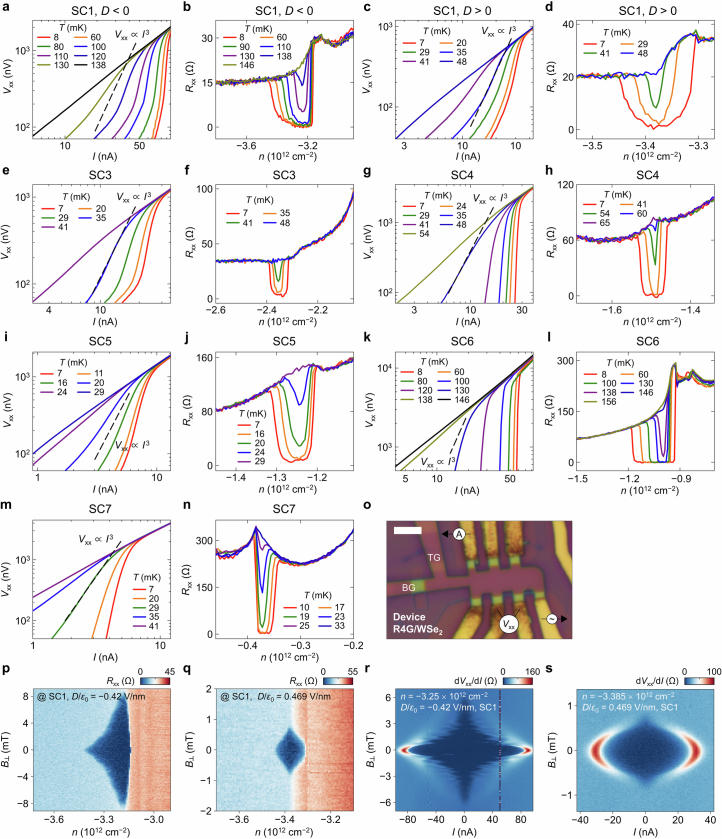
Additional data for SC in R4G/WSe_2_. Differential resistance as a function of *I* at varied temperatures for **a**, SC1 at negative *D* (*n* = −3.25 × 10^12 ^cm^−2^, *D*/*ε*_0_ = − 0.42 V/nm), **c**, SC1 at positive *D* (*n* = −3.385 × 10^12 ^cm^−2^, *D*/*ε*_0_ = 0.469 V/nm), **e**, SC3 (*n* = −2.356 × 10^12 ^cm^−2^, *D*/*ε*_0_ = 0.18 V/nm), **g**, SC4 (*n* = −1.482 × 10^12 ^cm^−2^, *D*/*ε*_0_ = 0.072 V/nm), **i**, SC5 (*n* = −1.242 × 10^12 ^cm^−2^, *D*/*ε*_0_ = 0.072 V/nm), **k**, SC6 (*n* = −1.00 × 10^12 ^cm^−2^, *D*/*ε*_0_ = 0.026 V/nm) and **m**, SC7 (*n* = −0.38 × 10^12 ^cm^−2^, *D*/*ε*_0_ = − 0.24 V/nm). The dashed lines indicate when *V*_xx_ is proportional to *I*^3^, corresponding to the BKT transition. *R*_xx_ as a function of *n* at varied temperatures for **b**, SC1 at negative *D* (*D*/*ε*_0_ = − 0.42 V/nm), **d**, SC1 at positive *D* (*D*/*ε*_0_ = 0.469 V/nm), **f**, SC3 (*D*/*ε*_0_ = 0.18 V/nm), **h**, SC4 (*D*/*ε*_0_ = 0.072 V/nm), **j**, SC5 (*D*/*ε*_0_ = 0.072 V/nm), **l**, SC6 (*D*/*ε*_0_ = 0.026 V/nm) and **n**, SC7 (*D*/*ε*_0_ = − 0.24 V/nm). **o**, Optical image of Device R4G/WSe_2_ and the representative measurement configuration. Scale bar: 3 μm. **p,q**, *R*_xx_ as a function of *n* and *B*_⊥_ for the SC1 on the WSe_2_-distant side (**p**) and the WSe_2_-proximal side (**q**). **r,s**, Differential resistance as a function of *I* and *B*_⊥_ for the SC1 on the WSe_2_-distant side (**r**) and the WSe_2_-proximal side (**s**). These data show the range of SC1 along the *n* axis, *I*_c_, and *B*_c,⊥_ all decrease when SC1 is placed to the WSe_2_-proximal side.

We first focus on the case when *B*_∥_ = 0 but *B*_⊥_ is nonzero. The calculated spin polarization along the *z*-axis, ${s}_{z}=({n}_{+}^{z}+{n}_{-}^{z})/|n|$, and the valley imbalance *δ* normalized by *n*_0_ are shown in Extended Data Fig. 9a,b. The spin polarization is saturated to unity at around *B*_⊥_ = 1.1 mT, regardless of the valley Zeeman effect. This field value is reasonably close to the optimal *B*_⊥_ (1.4–1.8 mT) found in experiments. The valley imbalance also rapidly increases up to the same field value and shows the cusp regardless of *g*_*V*_. This increase leads to the substantial change in the DOS of each valley and thus the enhancement of SC3 as explained previously. When the valley Zeeman term is turned on, the valley imbalance keeps increasing slowly above the optimal field, although *s*_*z*_ is saturated to unity. Nevertheless, this increase in *δ* is much slower than that driven by the spin canting, and the orbital depairing effect will probably become comparable to or more important than the valley Zeeman effect in experiments.

We can expect that the spins are steered ineffectively when *B*_∥_ comparable to *B*_⊥_ is applied. This can be directly shown in the calculation, including the spin Zeeman effect from *B*_∥_, as shown in Extended Data Fig. 9c. The increase in the valley imbalance by *B*_⊥_ becomes much slower under *B*_∥_ of a few mT. This is manifested in the SC3 phenomenology as the *B*_⊥_-induced enhancement disappearing by a tiny *B*_∥_ of a few mT, which is observed in Fig. 3i and Extended Data Fig. 9f–k. We note that there has been no example showing that graphene SCs are affected by this tiny *B*_∥_, and thus this peculiar observation well supports the validity of our model.

The theory can also be checked by increasing the SOC strength through the proximation to a TMD. This is because the spin vectors are pinned more robustly when stronger SOC is imposed, as the Ising SOC serves as a valley-contrasting effective magnetic field. This is explicitly shown in Extended Data Fig. 9d, where *s*_*z*_ was computed with *λ* = 0.05 and 1.0 meV. The spin polarization decreases substantially in the strong-SOC case. Accordingly, the valley imbalance arising from the valley-contrasting energy ±*λs*_*z*_/2 is suppressed. This is demonstrated in Extended Data Fig. 9e, where the valley imbalance at the optimal *B*_⊥_ is reduced by an order of magnitude (from 0.66% to 0.05%).

To test the scenario with varied SOC strength, we characterized device R5G/WS_2_ shown in Extended Data Fig. 9l. SC3 was weak (Extended Data Fig. 9n), but nonlinear *V*_*xx*_–*I* relations and peaks in d*V*_*xx*_/d*I* were observed. Extended Data Fig. 9o shows the evolution of d*V*_*xx*_/d*I* under *B*_⊥_ for SC3, featuring no enhancement behaviour. This is consistent with the prediction from our theory as discussed above.

### Effects of *B*_∥_ on a superconducting state

The in-plane magnetic field affects a superconducting state in two ways. First, it affects the free-energy difference between the SC and normal state, Δ*E*, depending on the spin configuration of a Cooper pair. For a spin-singlet Cooper pair, Δ*E* decreases as *B*_∥_ is applied due to spin Zeeman energy. For a spin-triplet Cooper pair, Δ*E* is mostly unaffected by *B*_∥_. Second, *B*_∥_ modifies the details of a parent state. For instance, both superconducting and normal states move in the *n*–*D* space by high *B*_∥_ (Fig. 2a). The modified parent state can give rise to a larger Δ*E* and thus a more stable superconductivity. As noted, when a Cooper pair is spin-triplet, the superconducting state does not gain energy from the normal state by *B*_∥_. In a naïve picture, therefore, *B*_∥_ will not enhance SC by the first effect. This leads to the hypothesis that the second effect (that is, modification of normal state) may result in the enhancement of SC2 and induction of SC4 by *B*_∥_. Further efforts are needed to separate the two coexisting effects here and understand the mechanism of the unusual responses of SC2 and SC4 under *B*_∥_.

### Ruling out other mechanisms for nonlinear d*V*_*xx*_/d*I*

Nonlinear *V*_*xx*_–*I* characteristics, one of the manifestations of superconductivity, can result from other mechanisms. We discuss how to exclude such possible mechanisms other than SC. First, nonlinear d*V*_*xx*_/d*I* can occur by the Joule heating effect from the drain–source current^73^. In this case, d*V*_*xx*_/d*I* monotonically increases by increasing the d.c. current, because the Joule heating power increases with the d.c. current. Our d*V*_*xx*_/d*I* data show peaks at a certain threshold current, which is inconsistent with this scenario. Second, nonlinear d*V*_*xx*_/d*I* can arise from a Schwinger-like effect^74–77^. The analogy of the Schwinger effect in quantum electrodynamics can come from the displacement of the Fermi surface by a large drain–source current. However, this current-induced displacement does not disappear by a small *B*_⊥_, which is inconsistent with our observations where nonlinear d*V*_*xx*_/d*I* is killed by a small *B*_⊥_. The same logic has been applied for distinguishing two different origins of nonlinear d*V*_*xx*_/d*I* in moiré graphene superconductors^76,77^.

### Recent reports on unusual SCs in rhombohedral graphene

During the preparation and after the submission of this work, there have been several recent reports on unusual SCs in rhombohedral graphene^70,78–83^.

## Online content

Any methods, additional references, Nature Portfolio reporting summaries, source data, extended data, supplementary information, acknowledgements, peer review information; details of author contributions and competing interests; and statements of data and code availability are available at 10.1038/s41586-026-10815-x.

## Supplementary information

Supplementary Information This file contains Supplementary Table 1 and Supplementary Fig. 1.

Peer Review File

## Data Availability

The data shown in the figures are available from 10.7910/DVN/H3LOEA (ref. ^84^). Other data that support the findings of this study are available from the corresponding authors on request.
