# Supplementary material for: Family of magnetic field-boosted superconductors in rhombohedral graphene
Source: Nature. 2026 Jun 29;656(8126):60–6. doi: 10.1038/s41586-026-10815-x (PMC13441972; doi:10.1038/s41586-026-10815-x)
Supplement: Supplementary file 1 [file 41586_2026_10815_MOESM1_ESM.pdf]

nature portfolio

https://doi.org/10.1038/s41586-026-10815-x

Supplementary information

Family of magnetic field-boosted superconductors in rhombohedral graphene

In the format provided by the authors and unedited

Supplementary Information for

Family of magnetic field-boosted superconductors in rhombohedral graphene

Junseok Seo[1,†], Armel A. Cotten[2,†], Shenyong Ye[1,†], Mingchi Xu[2], Omid Sharifi Sedeh[2], Henok Weldeyesus[2], Tonghang Han[1], Zhengguang Lu[1,3], Zhenghan Wu[1], Wei Xu[1], Jixiang Yang[1], Emily Aitken[1], Prayoga P. Liong[1], Phatthanon Pattanakanvijit[1], Zach Hadjri[1], Rasul Gazizulin[4,5], Kenji Watanabe[6], Takashi Taniguchi[7], Mingda Li[8], Dominik M. Zumbühl[2,*], Long Ju[1,*]

[1]Department of Physics, Massachusetts Institute of Technology, Cambridge, MA 02139, USA
[2]Department of Physics, University of Basel, Basel 4056, Switzerland
[3]Department of Physics, Florida State University, Tallahassee, FL 32306, USA
[4]Department of Physics, University of Florida, Gainesville, FL 32611, USA
[5]National High Magnetic Field Laboratory MagLab High B/T Facility, University of Florida, Gainesville, FL 32611, USA
[6]Research Center for Electronic and Optical Materials, National Institute for Materials Science, 1-1 Namiki, Tsukuba 305-0544, Japan
[7]Research Center for Materials Nanoarchitectonics, National Institute for Materials Science, 1-1 Namiki, Tsukuba 305-0544, Japan
[8]Department of Nuclear Science and Engineering, Massachusetts Institute of Technology, Cambridge, MA 02139, USA

[†]These authors contributed equally to this work.
*Corresponding authors. longju@mit.edu (L.J.), dominik.zumbuhl@unibas.ch (D.M.Z.)

Device	Observed SCs	$R_{SC,residual}$ / R_n		Anomalous magnetic field responses
R4G−1	SC1−2	SC1	≈0	N.A.
		SC2	0.09	
R4G−2	SC1−2	SC1	0.07	N.A.
		SC2	0.13	
R5G−1	SC1−3<br>SC4 at high $B_\parallel$	SC1	≈0	SC2 enhanced by $B_\parallel$<br>SC3 enhanced by $B_\perp$<br>SC4 induced by $B_\parallel$
		SC2 (8.0 T)	0.33	
		SC3	0.09	
		SC4 (8.0 T)	0.55	
R5G−2	SC1−3<br>SC4 at high $B_\parallel$	SC1	≈0	SC2 enhanced by $B_\parallel$<br>SC3 enhanced by $B_\perp$<br>SC4 induced by $B_\parallel$
		SC2 (8.0 T)	0.07	
		SC3	≈0	
		SC4 (8.835 T)	0.17	
R5G−3	SC1−3<br>SC4 at high $B_\parallel$	Not tested above the base temperature		SC2 enhanced by $B_\parallel$<br>SC3 enhanced by $B_\perp$<br>SC4 induced by $B_\parallel$
R5G−4	SC1−3<br>Not tested at high $B_\parallel$	Not tested above the base temperature		SC3 enhanced by $B_\perp$
R5G−5	SC1−3<br>Not tested at high $B_\parallel$	Not tested above the base temperature		SC3 enhanced by $B_\perp$
R4G/WSe_2	SC1 & SC3−7	SC1	≈0	N.A.
		SC3	0.10	
		SC4	≈0	
		SC5	0.04	
		SC6	≈0	
		SC7	≈0	
R5G/WS_2	SC1−3<br>Not tested at high $B_\parallel$	Not tested above the base temperature		N.A.

Table S1. Summary of the studied devices. $R_{SC,residual}$ is the measured R_{xx} at the base temperature (i.e., below the SC transition temperature) of 7 mK. R_n is the normal-state resistance, defined as largely temperature-independent R_{xx} above the transition temperature (see Extended Data Fig. 3c for example). The values of $R_{SC,residual}/R_n$ are measured at zero magnetic fields, unless the in-plane magnetic field is specified. Since these values require temperature-dependent measurements, we did not put them for devices that were characterized at the base temperature only. For the devices measured at the base temperature only, the enhancement of SC3 by $B_\perp$ was concluded from the increase in I_c by applying small $B_\perp$. Here, the critical current could be extracted from the position of the DC current for peaks in dV_{xx}/dI.

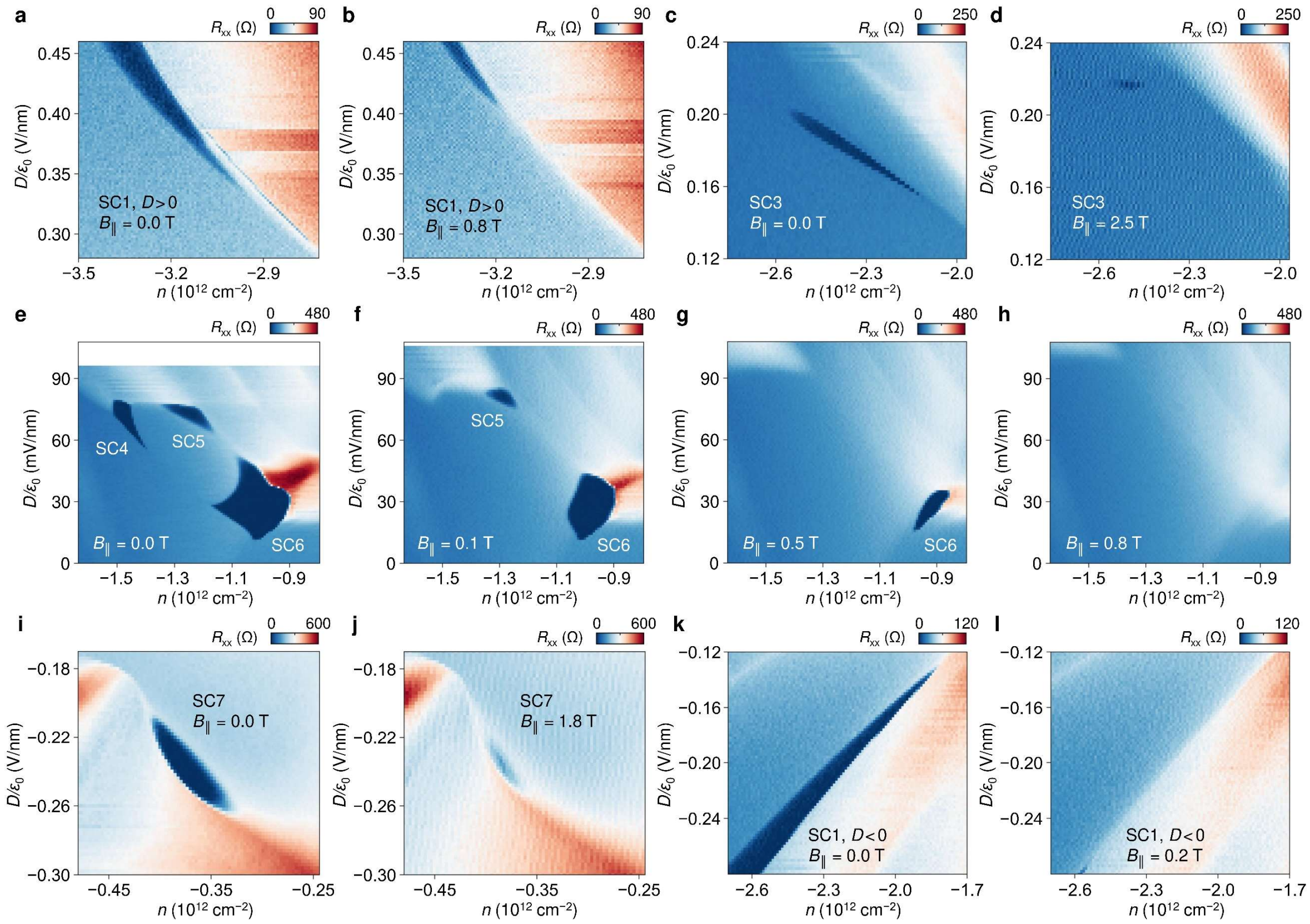

Figure S1. $B_\parallel$ responses of SCs in R4G/WSe₂. a,b, SC1 at the WSe_2-proximal side for $B_\parallel$ = 0.0 (**a**) and 0.8 (**b**) T. **c,d,** SC3 for $B_\parallel$ = 0.0 (**c**) and 2.5 (**d**) T. **e-h,** SC4-6 for $B_\parallel$ = 0.0 (**e**), 0.1 (**f**), 0.5 (**g**) and 0.8 (**h**) T. **i,j,** SC7 for $B_\parallel$ = 0.0 (**i**) and 1.8 (**j**) T. **k,l,** SC1 at the WSe_2-distant side for $B_\parallel$ = 0.0 (**k**) and 0.2 (**l**) T. All the observed SCs are monotonically suppressed by applying $B_\parallel$. SC3 and SC7 also feature high PVR values, referring to T_{BKT} extracted from Extended Data Fig. 10e and 10m.
