# Supplementary material for: Family of magnetic field-boosted superconductors in rhombohedral graphene
Source: Nature. 2026 Jun 29;656(8126):60–6. doi: 10.1038/s41586-026-10815-x (PMC13441972; doi:10.1038/s41586-026-10815-x)
Supplement: Supplementary file 2 [file 41586_2026_10815_MOESM2_ESM.pdf]

nature portfolio

Peer Review File

Family of magnetic field-boosted superconductors in rhombohedral graphene

Corresponding Author: Dr Long Ju

Any redactions in this file are there to maintain patient confidentiality, the confidentiality of unpublished data, or to remove third-party material.

This file contains all reviewer reports in order by version, followed by all author rebuttals in order by version.

Version 0:

Reviewer comments:

Referee #1

(Remarks to the Author)
I have reviewed the manuscript entitled "Family of unconventional superconductivities in crystalline graphene". In this work, the authors perform transport measurements on hole-doped rhombohedral 4- and 5-layer graphene devices (R4G and R5G) encapsulated by hBN, as well as an R4G device proximitized on one side by WSe2. (Data focusing on the electron-doped side, which shows signatures of chiral superconductivity, were reported separately in a Nature publication by the same group.) The authors report a variety of superconducting phases with unconventional transport phenomenology, in particular with regards to behaviors under applied in-plane and out-of-plane magnetic fields: (1) large Pauli-limit violations (i.e. stability under in-plane fields B_parallel), (2) induced or enhanced superconductivity with applied B_parallel, and even (3) enhancement of pairing with out-of-plane magnetic fields B_perp. While the first two properties have been observed before in different rhombohedral graphene systems, the last (and most unusual) property hasn't been convincingly demonstrated before to my knowledge. The data shown is of high quality, and I have found the paper to be well written, clearly presented and the analysis sound (see however below for comments and questions).

By now essentially every member of the rhombohedral graphene family studied in transport was shown to superconduct in at least one part of its (density, displacement field, magnetic field and spin-orbit-coupling tuned) phase diagram. So, while the results presented here for the hole-doped region of R4G and R5G don't necessarily come as a surprise, they are still very exciting and important in trying to sort out the expanding zoo of rhombohedral graphene superconductors, and ultimately figuring out their pairing symmetry and mechanism. For this reason I am inclined to recommend publication in Nature.

Detailed comments and questions:

(1) Figure 1: Why is the acronym PIP (partially isospin polarized) used here? It seems like a fully polarized half-metal (f_nu = ½) according to data in panels e and f, with only two Fermi surfaces.

(2) Figure 2: According to their interpretation of quantum oscillation data (with f_2 different from f_3), I think the normal-state drawing (yellow Fermi surfaces) should have two small pockets with different Luttinger volumes? Is the idea that these two pockets become of the same size as carrier density is varied towards SC2?

(3) Similarly: the caption of Fig 2 says "the relation (f_1 - f_2 - f_3) = ½ holds for SC2 (gray range in the bottom panel)", but in this gray range one of the frequencies has already disappeared. I suggest rephrasing slightly.

(4) Figure 2e: at what temperature is the Hall measurement of the normal state done?

(5) Lines 165-166: "We note, however, that the latter is quickly suppressed"... I don't think that's a fair characterization of the data in the referenced work. The PVR reported for the Bernal bilayer superconductor is also very large, at >15 or so (Note that this superconductor has a critical temperature closer to 25mK, so the Pauli limiting critical field is correspondingly much lower than here). So R2G is still a non-BCS superconductor, with other independent evidence suggesting it's also spin-polarized.

(6) Line 208: why mention here that SC3 is among the superconductors with the highest value of PVR reported? While true, it reads weird because SC4 was just discussed beforehand, in which the PVR is even higher.

(7) General comment about lines 215-216: I feel like the statement that B_perp enhances I_c and T_BKT for SC3, but that the enhancement disappears under B_parallel, provides strong constraints as to the underlying physics, which is not really fleshed out in the work in terms of possible interpretations.

(8) Line 222: "excludes orbital magnetism as a possible origin". I agree that orbital magnetism arising from the normal state seems to be excluded. However, we could imagine a chiral superconductor that spontaneously arises from a TRS-preserving metallic state (such as a valley-unpolarized state). In this case, wouldn't the resulting superconducting state couple linearly to a magnetic-field due to its spontaneous orbital magnetic moment (i.e. Cooper pairs carrying non-zero angular momentum)?

(9) Zeeman discussion, lines 222-223: there could be spin anisotropies to contend with at those energy scales. The Zeeman splitting is estimated at < 1 micro-eV, whereas Kane-Mele SOC in bare graphene is ~tens of micro-eV). So it's not obvious that we can simply equate the effect of Zeeman splitting on the band structure for out-of-plane and in-plane fields.

(10) Fig 4: Broken symbols (at least on my PDF) for the units of resistance \Omega/[broken]

(11) Naive question: why use the square resistance R_s rather than the normal-state resistance in Fig 4? The quantity appearing in the mean-free path l (to estimate a measure of disorder) should be R_n?

(12) Fig 4: No detailed data is presented here (or in the SM Figure 9) for the phase labeled SC5 in the phase diagram: is the superconductivity in that region too weak to characterize fully?

(13) Line 294: "suggesting spin-polarized instead of Ising SC". I don't think that's obvious. The scale of intrinsic Kane-Mele SOC in graphene (which acts as an Ising-type SOC when projected to a single layer by the displacement field) has been estimated using different techniques to be ~50 micro-eV or so. Assuming BCS scaling for simplicity, the gap size Delta~1.76 k_B T_c for the superconductors reported in this work are of the order (using T_c ~ 100 mK) ~15 micro-eV. So, in the absence of applied magnetic fields or other spin polarization, the system would be in a Ising-dominated regime from the perspective of pairing. Of course, that story changes if an in-plane field is applied or if the system develops ferromagnetic instabilities. In that case, the normal state is likely spin-canted (as discussed in Refs. 38, 53) with a large in-plane moment.

(14) SM Fig 6: the perpendicular field-induced enhancement is much weaker in this second device. Do the authors have more data on B_perp enhancement in Device 2 to put the findings into context (comparing e.g. to the much more extensive SM Fig 5)? It would be good to ascertain reproducibility more thoroughly, given that this effect is the most unusual reported in this work.

(15) SM Fig 8: what density and displacement was used for the B_parallel and B_perp evolution?

Finally, I have an optional suggestion for the authors, which I am aware may go beyond the scope of their work:

(16) Do the authors have similar data on the B_parallel and B_perp field evolution of the superconductors in the R4G/WSe2 proximitized device? It would be a very interesting comparison to the (in-plane and out-of-plane) B-field enhancements reported in Figs 1,2,3 — and perhaps tie the story together. Coming back to point (13) above: as far as the spin structure of the normal state is concerned, the distinction between hBN and WSe2-encapsulated devices may be primarily the magnitude of the anisotropy induced by Ising SOC. In the hBN case the system is more isotropic and the spin polarization can follow the direction of applied magnetic fields more easily than with WSe2, where there is a ~1 meV-scale easy-axis anisotropy to fight against. Such measurements could also test whether the spin-canted phenomenology reported in the normal state of R3G/WSe2 in Ref. 38 is also relevant here in R4G/WSe2, possibly supporting or ruling out spin-fluctuation mechanisms as in https://arxiv.org/abs/2406.17036 and follow-up theory works.

Referee #2

(Remarks to the Author)
The manuscript reports on the observation of superconductivity in rhombohedral tetralayer and pentalayer graphene. While the exploration of superconductivity in graphene-based systems remains a topic of significant interest, I have major reservations regarding the novelty, the strength of the evidence, and the depth of insight provided by this study. This work appears preliminary and does not meet the high standard of significance required for publication in Nature. The claims are not sufficiently substantiated, and the analysis lacks the rigor needed to advance the field.

Major Concerns:
1. The report of superconductivity in rhombohedral (R) graphene is not new, as it has already been established in R3G, R4G, and R5G systems by prior studies, including work from this same group. Therefore, the central finding of this manuscript—the existence of superconductivity in R4G and R5G— is rather not a discovery of new superconducting systems. The authors have not articulated what new physical principle or conceptual understanding this work provides beyond the existing literature. It remains primarily a report on specific devices without delving into new underlying mechanisms that would justify its publication in a high-impact journal. The study does not move the field forward

meaningfully.
2. The central claim of superconductivity is not fully supported by the data, which lacks several critical components expected for a conclusive demonstration.
For states other than SC1, the resistivity does not reach zero, which is a fundamental hallmark of a superconducting transition. This raises serious doubts about the nature of these phases.
The critical current-like behavior observed in dI/dV-I measurements could potentially be explained by non-superconducting out-of-equilibrium phenomena. The authors do not adequately rule out such alternative interpretations.
A cornerstone experiment for confirming superconductivity in a 2D device is the characterization of the critical current or Josephson junction behavior under a perpendicular magnetic field, leading to Fraunhofer-like interference patterns. The absence of this standard characterization is a critical omission that severely weakens the superconducting claim.
Even if one were to accept the superconducting claims, the reported critical parameters (current, field, temperature) are inferior to those already documented in other graphene systems, further questioning the significance of the finding.
3. The interpretation of the data is predominantly phenomenological and descriptive, lacking the deep physical analysis required for a high-impact publication.
The authors identify a "spin-polarized" superconducting state but provide no physical understanding or theoretical framework for its origin.
The SC3 induced by a perpendicular magnetic field is unconventional and counter-intuitive for a superconductor. This remarkable observation is merely reported without any substantive attempt at a physical explanation, which is a major missed opportunity.
While the authors call for theoretical efforts, such a call is not a substitute for providing their own initial hypotheses or a deeper discussion based on existing theories. The manuscript does not move beyond data presentation to offer new physical insights.

Minor points:
4. Optical images of the fabricated devices and the full van der Waals heterostructure are essential for assessing sample quality and geometry. Their absence makes it difficult to evaluate the experimental setup and the reproducibility of the device fabrication.
5. The claim of superconductivity in each system is seem to be supported by data from only a single device. Given the known variability in van der Waals devices and the strength of the claims, this is inadequate. Multiple devices are necessary to establish the reproducibility and reliability of the observed phenomena.

Referee #3

(Remarks to the Author)
The manuscript by Seo and co-authors reports a study of superconductivity in tetra- and penta-layer rhombohedral graphene (R4G and R5G). This study focuses on the hole-doped regime, where the authors report the discovery of several new superconducting states. First of all, in both R5G and R4G samples, they observe a superconducting state SC1 that is similar to the one reported in R3G earlier. In addition, they also report several other superconducting states in R5G with unusual magnetic field dependences. These states violate the Pauli limit, suggesting unconventional pairing, and also show enhancement with in-plane field (SC2, SC4) or out-of-plane field (SC3). The authors also report how adding the spin-orbit coupling affects the superconducting states in a R4G/WSe_2 device.

Rhombohedral few-layer graphene has been shown to have an extremely rich phase diagram of strongly correlated electronic phases, including superconductors, integer and fractional quantum anomalous Hall states, and candidates for chiral superconductivity. The reported discovery of several new superconducting states with unusual responses to magnetic field is exciting and will be of high interest to a broad community of condensed matter physicists. However, as I elaborate below, my primary concern is that the presented experimental evidence might not be sufficient to establish the presence of new superconducting states, at least for some of those states. In particular, it appears that some of the superconducting states are only observed in individual devices. Moreover, the authors do not provide an explicit discussion of the number of measured devices and/or the measured contact configuration. The authors also do not provide the information about the devices geometry. However, this information is crucial for establishing the credibility of their exciting finding. The authors need to address these issues and some other comments before I can recommend the manuscript for publication.

The main findings of this work build upon the discovery of several new superconducting states. Hence, it is crucial that the existence of those superconducting states is established with high credibility. Unfortunately, the resistance measurements often do not provide unambiguous evidence of superconductivity in graphene systems where both ballistic transport and numerous phase transitions between symmetry-broken metals are possible. For example, temperature-driven phase transitions to various symmetry-broken metallic states, which are so ubiquitous in rhombohedral and moiré systems, can look very similar to superconductivity (for example, as was pointed out in He. et al. Nature Physics 17 (2021)). Moreover, there are numerous examples when the initial reports of superconducting states observed in transport in graphene systems were not confirmed in the subsequent studies of those systems. To the best of my knowledge, this is the case with superconducting states in rhombohedral trilayer aligned to hexagonal boron nitride (arXiv:1901.04621), some superconducting states at low superlattice fillings in twisted bilayer graphene (arXiv:1903.06513), twisted double-bilayer (arXiv:1903.08130), and in twisted monolayer-bilayer graphene (arXiv:2004.12414). In the last two examples, the claim of superconductivity was removed or softened in the published versions. It is worth pointing out that both the temperature dependence of the resistivity and even the I-V curves can look similar to those associated with superconductivity.

It is even more critical to ensure the state is a superconductor when these candidate states manifest some features that are

typically not shown by superconductors, such as enhancement by in-plane or out-of-plane magnetic field reported by the author in this manuscript, or when the candidate states do not show zero resistance, which is the case with SC2 (Fig. 2). The authors observe some signs of possible interference (Ext. Fig. 7) which could provide some additional evidence for superconductivity, but they are relatively weak and only reported for SC3.

While observing some other key experimental signatures of superconductivity, such as the Meissner effect, is nearly impossible in graphene, one of the most effective ways to confirm new superconducting states has been to observe the same phenomenology in multiple devices, as demonstrated in the examples I mentioned above. That's why observing a superconducting state in resistance measurements of only a single device is generally not sufficient for claiming superconductivity with enough confidence. Unfortunately, in their manuscript, the authors do not explicitly discuss the number of measured devices (except mentioning R5G Device 2 in Extended Data Fig.6). The authors also do not discuss measuring different contact configurations, nor do they provide information about device geometry. This crucial information is required to evaluate the credibility of the claim.

Specific questions and comments:

In how many devices do the authors observe each of the reported superconducting states?

Information about the device geometry is crucial in the context of this study and also for the attempts to reproduce the reported findings. In the current version of the manuscript, the authors do not provide such information. Please include the images of the devices in the manuscript.

If devices had multiple electrical contacts, were the reported superconducting states observed for different contact combinations?

Measuring weak superconducting states in graphene devices is technically challenging, with electronic noise and high effective electron temperature sometimes playing a major role in obscuring the superconducting states. In the current version of the manuscript, the authors do not provide details on the measures taken to reduce noise and thermalize electrons. This information is crucial for attempts to reproduce these results. Please include the details of the electrical filtering and thermalization stages used on the measurement lines.

The authors mention two distinct dilution refrigerator systems used in the experiments. Were the reported superconducting states observed in both of them?

The authors provide an extensive discussion of the evolution of SC2 with the in-plane magnetic field (Fig. 2a), arguing that the superconductivity is enhanced. However, by comparing 0T and 8T panels, one can see that the normal state itself is strongly modified by the high in-plane magnetic field. At 8T, the entire SC2 region and the adjacent resistive metallic state are strongly shifted to higher displacement fields. In fact, one can strategically pick a (n,D) point for which a small in-plane magnetic field suppresses rather than enhances the superconductivity. Since the underlying band structure is strongly modified by the field, it is difficult to separate the direct effect of the field on the SC state from the indirect effect via the altered normal state. I think this should be directly acknowledged in the discussion.

The data for SC2 shows that the resistance of this state does not go to zero, but in some cases only decreases by about half (e.g., from ~30 to ~15 Ohm in Fig. 2 c and h). Moreover, if one compares SC2 to SC1 and SC3, all three states have comparable critical currents and transition temperatures, but the resistance approaches zero only for SC1 and SC3, but not for SC2. Fig 2h also shows that the resistance in SC2 has some strong carrier-density dependence. All these anomalies suggest a possibility that SC2 might not be a superconducting state, but some distinct symmetry-broken metal, as was discussed above.

Fig.3b shows the Fourier transform of the resistance as a function of 1/B. However, in contrast to other similar plots in the paper, the authors do not provide the corresponding original data R(B,n). It is also not specified at what displacement field this data is taken.

In the second-to-last paragraph of the main text, the authors discuss violation of the Pauli limit and field-enhanced superconductivity, citing some references on these observations in non-graphene systems. From reading this paragraph, one might get the impression that similar observations have never been made in graphene systems before. However, this is not the case. The Pauli limit violation has been reported in twisted trilayer graphene (Cao et al. Nature 595 (2021)) and in rhombohedral trilayer graphene (Zhou et al., Nature 598 (2022)). The superconductivity induced by in-plane field was previously reported in Bernal bilayer graphene (Zhou et al., Science 375 (2022)), and the authors cite this earlier in the manuscript.

Version 1:

Reviewer comments:

Referee #1

(Remarks to the Author)
The authors have answered my questions satisfactorily. They also present data on different R4G and R5G devices, with the

conclusions broadly consistent with the original claims, assuaging concerns of reproducibility. The authors also present data at a lower electronic temperature for R4G/WSe2, which allowed them to study more fragile superconducting states (such as SC5) that were not convincingly demonstrated in the first version of the manuscript. Finally, the addition of magnetic field response of the various R4G/WSe2 superconductors reveals very different phenomenology (no field enhancement) from the hBN devices, an important piece of information to constrain theoretical models.

To recap the main criticisms (from all the referees) in the first round of review: (1) The presence of various superconducting states in this system is not really unexpected, given the vast family of known rhombohedral graphene superconductors. (2) The manuscript leaves central questions of interpretation outstanding (for example, the reason behind the reported out-of-plane enhancement of superconductivity). The authors provide more context for these observations in the revised manuscript by ruling out some natural explanations, but they do not flesh out a reasonable candidate scenario – leaving it to future theory work – which feels unsatisfying.

With these caveats in mind, I believe the work uncovers sufficiently new and unconventional superconducting phenomenology in hole-doped R4G and R5G to capture the attention of the community. The experimental results are very extensive and will drive the field forward in search of a unifying theoretical explanation for the mysterious observed phenomena. For this reason I am inclined to recommend publication of the work in Nature.

I have two detailed comments below:

(1) When the authors discuss the effect of Hund's coupling in the discussion, I would emphasize (to contrast with WSe2 devices): "since the SOC of graphene is much weaker than Hund's coupling" -> "since the intrinsic SOC of graphene is much weaker than Hund's coupling"

(2) In response to my previous comments, the authors carried out additional measurements and emphasize that "No enhancement of SC by $B_\perp$ was observed in any of the SCs in R4G/WSe2." This seems like a key piece of information to constrain future theoretical explanations of the $B_\perp$ enhancement reported in the hBN devices. It should be pointed out more clearly in the main text (apologies if I missed it) and referred to the appropriate data in the SM Figures. Similarly, the in-plane field response of R4G/WSe2 could make it into an additional SM Figure (combining some representative data from the reply figures R8-12)

Referee #2

(Remarks to the Author)
I appreciate the authors' efforts in revising the manuscript and responding to the referees' comments. However, after careful evaluation of the revised version and comparison with existing literatures, I remain unconvinced that the findings presented here constitute a sufficiently significant advance over prior work. Therefore, I cannot recommend publication in Nature, which requires a particularly high standard of novelty and conceptual impact. My assessment is based on the following points:
1. The enhancement of superconductivity under in-plane magnetic fields (SC2 and SC4) has already been extensively reported in multiple graphene-based systems, including bilayer graphene (Science 375, 774–778 (2022)), rhombohedral graphene (Nature 598, 434–438 (2021); Nature 643, 654–661 (2025), from the same group), and twisted trilayer graphene (Nature 595, 526–531 (2021)). While the authors may argue that their devices exhibit higher PVRs, this represents only a marginal quantitative improvement rather than a qualitatively new phenomenon. This does not, in my view, meet the threshold of significance required for publication in Nature.
2. The SC3 state, emphasized by the authors and observed under perpendicular magnetic field in R5G, also lacks clear novelty. Similar behavior has been reported in other systems, such as $MoTe_2$ junctions (see Fig. 4 in Science Advances, eadq5712 (2025)), which the authors have not adequately acknowledged or discussed.
3. A major concern from the first round was the absence of a convincing physical interpretation. This issue persists. Neither their experimental data nor existing theoretical frameworks sufficiently clarify the origin of SC3. As noted previously, the manuscript does not articulate a new physical principle or conceptual advance; rather, it remains largely a phenomenological report.
4. Schwinger-like nonlinear I–V characteristics have been reported in multiple prior works (Science 375, 430–433 (2022); Nature 614, 440–444 (2023); Nature Physics 19, 830–835 (2023); arXiv:2501.06460 (2025)). Such effects are expected in flat-band systems due to their extremely low Fermi velocity. The revised manuscript still does not convincingly exclude these non-superconducting mechanisms, leaving ambiguity in the interpretation of the observed states.
5. Finally, the identification of superconductivity based on nonzero resistance still raises concerns. This issue is especially critical for a journal like Nature. The claim should be made more cautiously, given that the normal-state resistance is already very low (on the order of tens of ohms), comparable to the residual resistance in the purported superconducting state. As noted by another referee, there are precedents where initial claims of superconductivity were not substantiated by subsequent studies.

Referee #3

(Remarks to the Author)
I would like to thank the authors for providing detailed and thoughtful responses to my questions and for addressing the concerns I raised.

The new data, for both previously studied and newly fabricated devices of rhombohedral graphene multilayers, significantly

strengthen the authors' claim of unconventional superconducting states in these systems and enhance the value of the results for the community. The additional measurement details further improve the credibility of the reported findings.

I recommend the revised version of the manuscript for publication.

Version 2:

Reviewer comments:

Referee #1

(Remarks to the Author)
The authors have answered my questions satisfactorily. First, the addition of data from a new spin-orbit proximitized R5G device, and the confirmation that no anomalous enhancement of superconductivity occurs under a perpendicular magnetic field, completes the experimental picture.

On the theory side, I am glad to see that (as expected on general symmetry grounds) the modeling holds up when relaxing the unphysical assumption that the two valleys must host equal spin polarization, as pointed out in my previous note. The addition of valley Zeeman coupling, and the direct computation of valley imbalance (as opposed to indirectly through spin imbalance) paints a clear story, with immediate observable consequences that could be tested in future experiments (e.g. using magnetometry). The additional discussion of the role of enhanced Ising SOC, and the corresponding suppression of induced valley imbalance by B_perp, nicely complements the new data on the spin-orbit proximitized R5G device mentioned above. Overall, I think the revised theory modeling is a valuable addition to the manuscript. I would simply ask that the authors cite the manuscript Kumar et al. in the context of their theory modeling, since the fully unconstrained (n_+ != n_-) free energy minimization in a field-biased spin-canted state originates from there (hence the inspiration behind my question in the previous report).

Overall I find the work to be very timely and interesting. It uncovers new and intriguing phenomenology within the large family of rhombohedral graphene superconductors, along with a reasonable theoretical proposal for the unconventional magnetic field response. The dataset is extensive, and now contains multiple R4G and R5G devices in both hBN and TMD-proximitized configurations, which allows for an interesting discussion of the role of induced SOC on superconducting phenomenology in rhombohedral systems. I am convinced the results will inspire many follow-up experiments and theory efforts aimed at resolving the remaining puzzles, and I therefore recommend its publication in Nature.

Referee #2

(Remarks to the Author)
I appreciate the authors' substantial efforts in addressing my previous concerns. The additional data have improved the manuscript and have made the experimental phenomenology clearer and more convincing.
My remaining comment concerns the presentation of the experimental criteria. The definitions of residual resistance, normal-state resistance, and critical current should be stated more explicitly. I encourage the authors to include a concise device summary table, listing the measured devices, the observed superconducting states, base temperatures, residual-resistance ratios, magnetic-field responses, and the criteria used to extract Ic. Such a summary would make the reproducibility and robustness of the superconductivity claims easier to assess.
Provided that this point is appropriately addressed in the revised manuscript, I would be pleased to support publication and would not require further review of the manuscript.

Open Access This Peer Review File is licensed under a Creative Commons Attribution 4.0 International License, which permits use, sharing, adaptation, distribution and reproduction in any medium or format, as long as you give appropriate credit to the original author(s) and the source, provide a link to the Creative Commons license, and indicate if changes were made.
In cases where reviewers are anonymous, credit should be given to 'Anonymous Referee' and the source.
The images or other third party material in this Peer Review File are included in the article's Creative Commons license, unless indicated otherwise in a credit line to the material. If material is not included in the article's Creative Commons license and your intended use is not permitted by statutory regulation or exceeds the permitted use, you will need to obtain permission directly from the copyright holder.
To view a copy of this license, visit https://creativecommons.org/licenses/by/4.0/

Responses to the referees

We sincerely thank the referees for taking the time to assess our manuscript and raising suggestions to improve it. We believe that this letter and the revised manuscript properly addressed their questions and comments. In short, we have measured five more devices and are able to repeat all the observations we reported in the first draft, making our report to be based on **a total of eight devices** that are measured independently in the labs at MIT and the University of Basel. In the following, we respond in detail to the questions and comments one by one. We here used blue color for the questions by the referees and black color for our responses. Changes made in the revised manuscript are highlighted in yellow.

Referee #1 (Remarks to the Authors):

I have reviewed the manuscript entitled "Family of unconventional superconductivities in crystalline graphene". In this work, the authors perform transport measurements on hole-doped rhombohedral 4- and 5-layer graphene devices (R4G and R5G) encapsulated by hBN, as well as an R4G device proximitized on one side by WSe2. (Data focusing on the electron-doped side, which shows signatures of chiral superconductivity, were reported separately in a Nature publication by the same group.) The authors report a variety of superconducting phases with unconventional transport phenomenology, in particular with regards to behaviors under applied in-plane and out-of-plane magnetic fields: (1) large Pauli-limit violations (i.e. stability under in-plane fields B_parallel), (2) induced or enhanced superconductivity with applied B_parallel, and even (3) enhancement of pairing with out-of-plane magnetic fields B_perp. While the first two properties have been observed before in different rhombohedral graphene systems, the last (and most unusual) property hasn't been convincingly demonstrated before to my knowledge. The data shown is of high quality, and I have found the paper to be well written, clearly presented and the analysis sound (see however below for comments and questions).

By now essentially every member of the rhombohedral graphene family studied in transport was shown to superconduct in at least one part of its (density, displacement field, magnetic field and spin-orbit-coupling tuned) phase diagram. So, while the results presented here for the hole-doped region of R4G and R5G don't necessarily come as a surprise, they are still very exciting and important in trying to sort out the expanding zoo of rhombohedral graphene superconductors, and ultimately figuring out their pairing symmetry and mechanism. For this reason I am inclined to recommend publication in Nature.

Response 1

We thank the referee for appreciating the quality and merits of our manuscript by summarizing the main points and comparing it with existing literatures. We tried our best to address the comments given by the referee.

Detailed comments and questions:

(1) Figure 1: Why is the acronym PIP (partially isospin polarized) used here? It seems like a fully polarized half-metal (f_nu = ½) according to data in panels e and f, with only two Fermi surfaces.

Response 2

Figure 1e and 1f in the manuscript show the evolution of fermiology as a function of charge density *n*

around SC1. There are three density ranges that correspond to different fermiology: the rightmost range (small negative n) corresponds to a fully polarized half metal, as the referee pointed out; the leftmost range (large negative n) corresponds to an annular full metal; the middle range, where f_ν shows one peak between 1/4 and 1/2 and another peak with a very low frequency, is assigned to be a PIP phase without a complete isospin polarization [H. Zhou et al. *Nature* **598**, 434-438 (2021)].

We understand that the labels in Fig. 1e&f could be misleading, so we addressed this by adding vertical dashed lines to separate the three ranges in Fig. 1e and 1f in the revised manuscript.

(2) Figure 2: According to their interpretation of quantum oscillation data (with f_2 different from f_3), I think the normal-state drawing (yellow Fermi surfaces) should have two small pockets with different Luttinger volumes? Is the idea that these two pockets become of the same size as carrier density is varied towards SC2?

Response 3

Yes, this is our interpretation. We believe the two pockets become to occupy the same size as the carrier density approaches SC2, as f_2 becomes identical to f_3.

(3) Similarly: the caption of Fig 2 says "the relation (f_1 - f_2 - f_3) = ½ holds for SC2 (gray range in the bottom panel)", but in this gray range one of the frequencies has already disappeared. I suggest rephrasing slightly.

Response 4

Thanks for pointing this out. In response to the referee's comment, we changed the caption of Fig. 2d to "Around SC2, f_2 and f_3 have merged and the relation $f_1 - 2f_3 \approx 1/2$ holds."

(4) Figure 2e: at what temperature is the Hall measurement of the normal state done?

Response 5

The Hall measurements in Figure 2e were performed at $T = 160$ mK, which is sufficiently above the T_c of SC2. In the revised manuscript, we specified in figure captions the mixing chamber temperature at which the Hall measurements for the normal state of SC2 (Fig. 2e) and SC3 (Fig. 3c) were carried out. Please note that 160 mK is also above the critical temperature of SC3.

(5) Lines 165-166: "We note, however, that the latter is quickly suppressed"... I don't think that's a fair characterization of the data in the referenced work. The PVR reported for the Bernal bilayer superconductor is also very large, at >15 or so (Note that this superconductor has a critical temperature closer to 25mK, so the Pauli limiting critical field is correspondingly much lower than here). So R2G is still a non-BCS superconductor, with other independent evidence suggesting it's also spin-polarized.

Response 6

We appreciate this concern raised by the referee and agree that SC found in bilayer graphene (R2G) also exhibits a remarkably high Pauli-limit violation ratio and is likely an unconventional superconductor. In

the revised manuscript, we deleted the sentence comparing SC4 in R5G with SC in R2G in the section "SC2 enhanced and SC4 induced by $B_{\parallel}$" as follows: "SC4 shows no sign of suppression at up to $B_{\parallel}$ = 8.5 T (Extended Data Fig. 7m). This exceeds the Pauli limit[6] of a BCS superconductor with the same transition temperature by a factor of ~65, taking 70 mK as a conservative estimate for T_{BKT}." Instead, we discussed the similar observation of field-induced SC in R2G in the Discussions and outlook as follows: "… This might also be the case for SC in R2G[32] that appears only under finite $B_{\parallel}$." This was to match the logic flow of the main text and not to mislead the readers that a similar field-induced SC has not been studied in a graphene system (**Response 42**).

(6) Line 208: why mention here that SC3 is among the superconductors with the highest value of PVR reported? While true, it reads weird because SC4 was just discussed beforehand, in which the PVR is even higher.

Response 7

We agree with the referee that mentioning the ultrahigh value of PVR in SC3 at this point might cause confusion. In the revised manuscript, the sentence that compares the PVR of unconventional SCs in this work is moved to the Discussions and outlook: "Especially, their remarkably high PVRs put them among superconductors with the highest PVR values reported[2,46]."

(7) General comment about lines 215-216: I feel like the statement that B_perp enhances I_c and T_BKT for SC3, but that the enhancement disappears under B_parallel, provides strong constraints as to the underlying physics, which is not really fleshed out in the work in terms of possible interpretations.

Response 8

We agree with the referee that the disappearance of SC enhancement under $B_{\parallel}$ should be discussed more, in terms of its implications on the underlying physics. At the first glance, this observation seems to suggest that the SC enhancement under $B_{\perp}$ is a spin effect: spin magnetization can couple to $B_{\perp}$ and result in a spin-polarized Fermi surface, while such spin-polarization disappears when a large $B_{\parallel}$ completely aligns spins to the in-plane direction. However, this picture is against the missing of SC enhancement by $B_{\parallel}$: spin effect should be isotropic in the half-metal state (see **Response 10** below), meaning the enhancement of SC by $B_{\perp}$ should also be observed when a comparable $B_{\parallel}$ is applied. Therefore, a pure spin effect cannot explain the enhancement of SC by $B_{\perp}$.

Next, we discuss the scenario of orbital effect enhancing SC. If there exists a spontaneous valley-polarization at the Fermi surface, the resulted net orbital magnetization could couple to $B_{\perp}$ and further enhance the density of states in one of the valleys to enhance SC [Z. Dong, A. V. Chubukov & L. Levitov, *Phys. Rev. B* **107**, 174512 (2023)]. However, we did not observe any signatures of orbital magnetization, due to valley polarization of the SC parent state (see Fig. 3c in the main text), or due to a spontaneous TRSB SC order parameter (see **Response 9** below). Of course, a valley polarization can be induced by $B_{\perp}$, even if the spontaneous valley-symmetry-breaking is absent at zero magnetic field. However, the in-plane field should not prevent this field-induced valley-polarization from enhancing the SC, since the application of $B_{\parallel}$ will not directly affect the valleys in a simple picture. This is against the observation of disappearance of SC enhancement by $B_{\perp}$ under $B_{\parallel}$.

In this regard, neither pure spin nor orbital effect can explain all of our experimental observations. However, we still followed the referee's suggestion by expanding our discussion on the implications of

experiment and possible interpretations. In the revised manuscript, we added sentences "Secondly, even without a spontaneous valley polarization, the valley-contrasted orbital magnetization can be generated by finite $B_\perp$ and induce the valley polarization. The density of states in one of the valleys may increase and thus enhance of SC3. This is however contradictory to the disappearance of the enhancement under nonzero $B_\parallel$, since the in-plane field is unlikely to affect the valleys directly." that flesh out the disappearance of the enhancement by $B_\perp$ under $B_\parallel$.

(8) Line 222: "excludes orbital magnetism as a possible origin". I agree that orbital magnetism arising from the normal state seems to be excluded. However, we could imagine a chiral superconductor that spontaneously arises from a TRS-preserving metallic state (such as a valley-unpolarized state). In this case, wouldn't the resulting superconducting state couple linearly to a magnetic-field due to its spontaneous orbital magnetic moment (i.e. Cooper pairs carrying non-zero angular momentum)?

Response 9

This is a very interesting and possible scenario. Naively, there could be two such chiral superconductors corresponding to the opposite chirality. In transport experiments that measure resistance, one can imagine magnetic switching with hysteresis when sweeping the out-of-plane magnetic field, similar to that observed in our previous work [T. Han et al. *Nature* **643**, 645-661 (2025)]. We have tested this possibility for SC3 by checking the response of resistance at the base temperature as $B_\perp$ is swept back and forth. Figure R1 shows the data taken from Device R5G-5. No obvious hysteretic behavior was observed. We therefore conclude that there is no experimental evidence of spontaneous orbital magnetism in SC3.

In the revised manuscript, we added the data in Figure R1 as Extended Data Fig. 9g, and changed the sentence in the main text as "the absence of magnetic hysteresis in the normal (Fig. 3c) and superconducting (Extended Data Fig. 9g) states of SC3 suggests the vanishing orbital ferromagnetism, …".

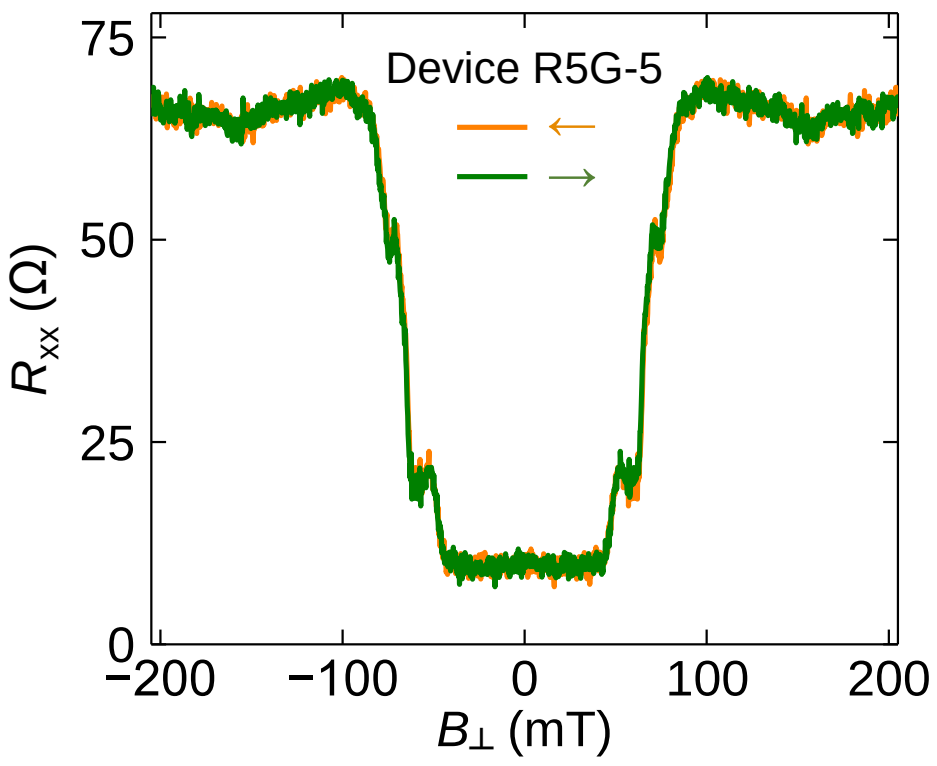

Figure R1. Absence of $B_\perp$ hysteresis of SC3 below its transition temperature. *R_{xx} as a function of $B_\perp$ in the forward and backward scanning directions for SC3 ($n = -1.132 \times 10^{12}$ cm^{-2}, $D/\varepsilon_0 = 54$ mV/nm) in Device R5G-5 at the base temperature. There is no hysteretic behavior observed, implying the absence of orbital magnetism in the superconducting state of SC3.*

(9) Zeeman discussion, lines 222-223: there could be spin anisotropies to contend with at those energy scales. The Zeeman splitting is estimated at < 1 micro-eV, whereas Kane-Mele SOC in bare graphene is ~tens of micro-eV). So it's not obvious that we can simply equate the effect of Zeeman splitting on the

band structure for out-of-plane and in-plane fields.

Response 10

We appreciate the referee for pointing out this question. It is true that Kane-Mele SOC can be much larger than the Zeeman splitting. However, there is Hund's coupling that dwarfs both SOC and Zeeman, and the Hund's coupling aligns spins in the half-metal state, instead of the valley-contrasted spin directions as determined solely by Kane-Mele SOC. Given the aligned spin configuration by the dominant Hund's coupling, the additional Zeeman energy applied by an external magnetic field will orient all the spins to the field direction, regardless the direction of magnetic field. This results in negligible spin anisotropy, even with the spin Zeeman energy of ~1 μeV [Z. Dong, E. Lantagne-Hurtubise & J. Alicea, arXiv:2406.17036 (2024)]. Experimentally, this picture is supported by the magnetometry measurements in rhombohedral trilayer graphene (R3G) [C. L. Patterson et al. *Nature* **641**, 632-638 (2025)]: in the half-metal phase in R3G, no observable spin anisotropy was found in the absence of proximitized SOC effect. Therefore, we think the effect of spin Zeeman splitting is similar when the field is pointed out-of-plane and in-plane for a half-metal, which is the parent state of SC3.

(10) Fig 4: Broken symbols (at least on my PDF) for the units of resistance \Omega/[broken]

Response 11

This might be a display issue of PDF: the units of resistance in Fig. 4h were denoted as Ω/□, as double-checked in Figure R2. This unit indicates that the resistance is normalized into *square* resistance by taking the sample geometry into account.

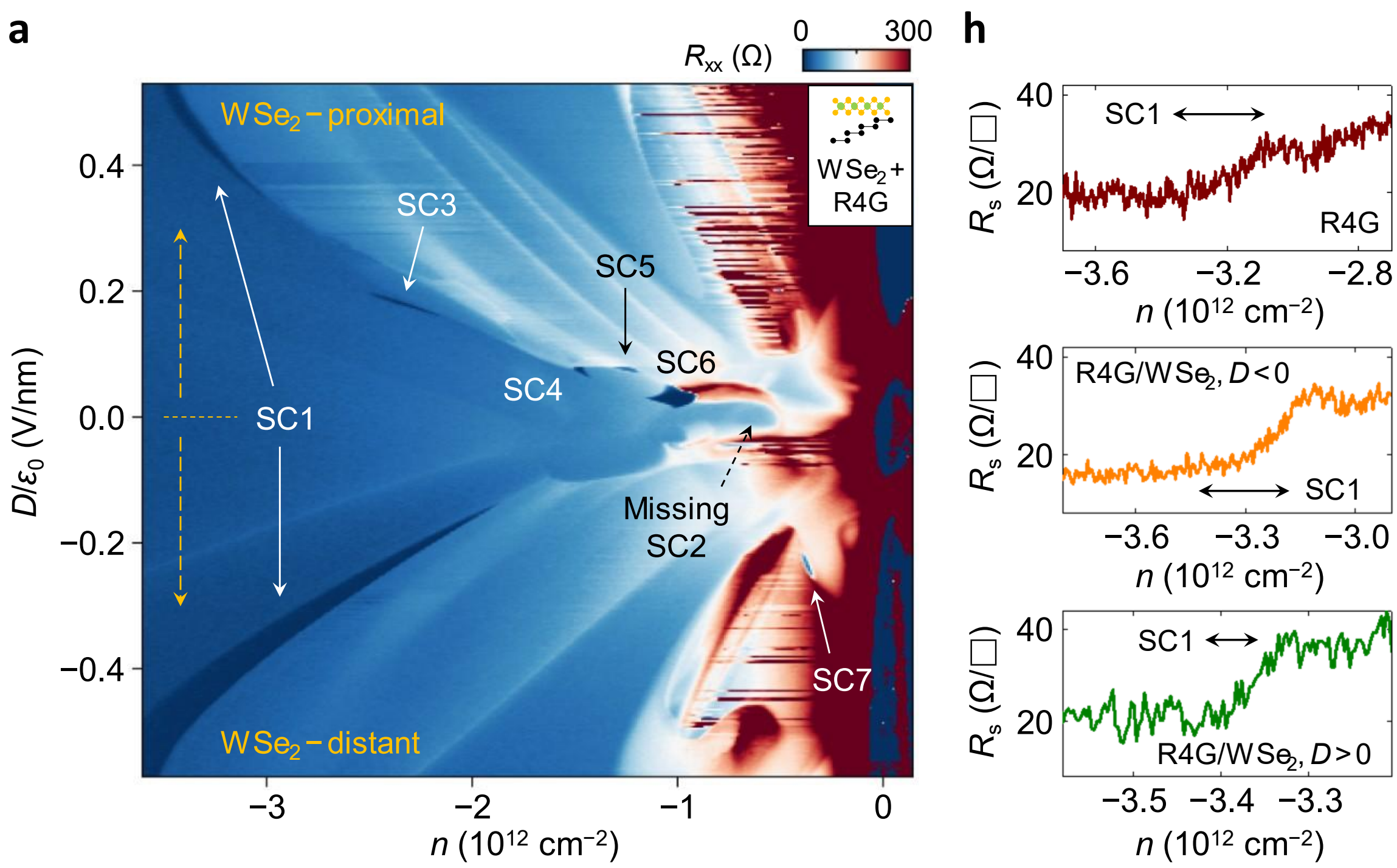

Figure R2. Part of Fig. 4 in the manuscript, showing the unit of R_s in the panel h.

(11) Naive question: why use the square resistance R_s rather than the normal-state resistance in Fig 4?

The quantity appearing in the mean-free path l (to estimate a measure of disorder) should be R_n?

Response 12

Yes, the mean-free-path depends on R_n, but the latter is also affected by the sample geometry, such as channel length and width. We chose to compare the square resistance R_s in Figure 4f for the fair comparison among different devices. When estimating the mean-free path l in line 103 of the previous manuscript, the square resistance was effectively used as the equation, $l \cong \frac{h}{e^2}\frac{L}{W}\frac{1}{4k_F R_n} = \frac{h}{e^2}\frac{1}{4k_F R_s}$, in that $R_s = R_n W/L$.

To avoid confusion by readers, we changed the equation to estimate the mean-free path and added the definition of the square resistance in the Methods Section of the revised manuscript as below.

Extraction of coherence length and mean-free-path: The coherence length ξ of a superconductor and the mean-free-path l of its normal state are extracted as $\xi = \sqrt{\frac{h/(2e)}{2\pi B_{c,\perp}}}$ and $l \cong \frac{h}{e^2}\frac{1}{4k_F R_s}$, respectively. Here, $B_{c,\perp}$ is the out-of-plane critical field, $R_s = R_n W/L$ is the square resistance of normal-state resistance R_n, $k_F \approx \sqrt{\pi n}$ is the Fermi wavevector, L and W are the length and width of the sample, h is Planck's constant and e is the elementary charge[36].

(12) Fig 4: No detailed data is presented here (or in the SM Figure 9) for the phase labeled SC5 in the phase diagram: is the superconductivity in that region too weak to characterize fully?

Response 13

We thank the referee for raising this question. We loaded the device back to fridge and measured the data for SC5 as shown in Figure R3. At the same time, we found all the superconductivities in R4G/WSe_2 were considerably enhanced, due to our efforts on electronic filtering and the resulted lower electron temperature. The new data taken in this measurement run are demonstrated in Figure R4 and R5.

In the revised manuscript, we added the SC5 data from detailed characterizations of Device R4G/WSe_2 in Figure 4 and Extended Data Figure 10. We also updated the data for the other superconductivities found in Device R4G/WSe_2 in Figure 4 and Extended Data Figure 10.

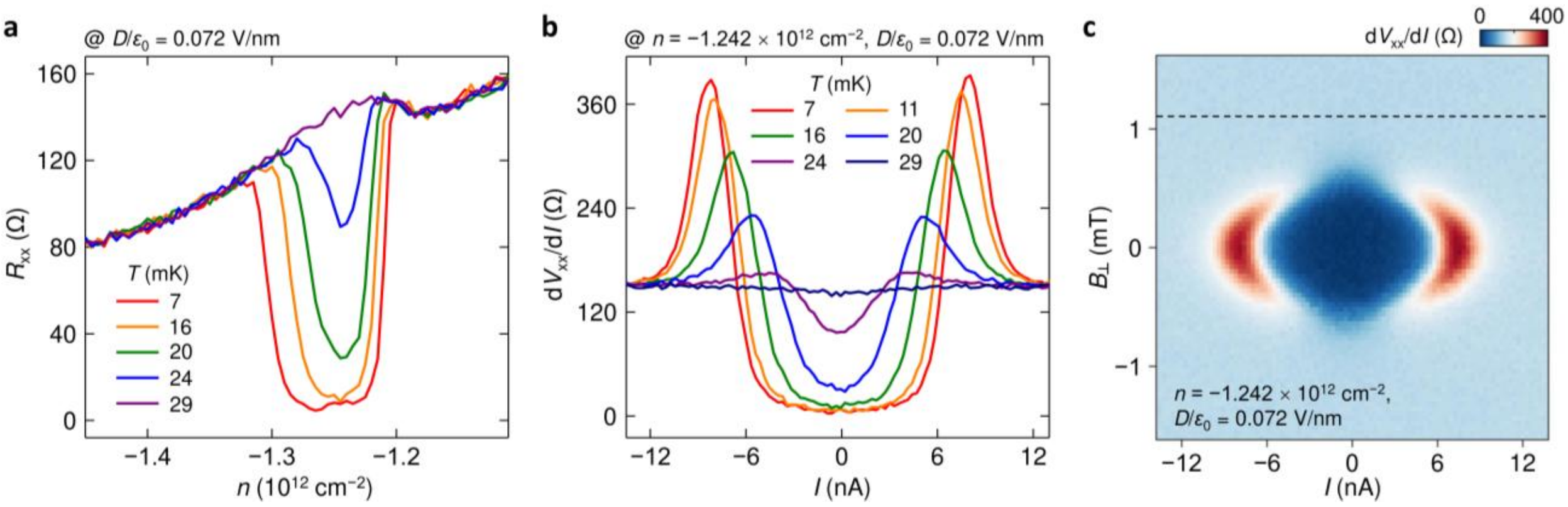

***Figure R3. Detailed characterizations of SC5 in R4G/WSe_2. a**, Resistance R_{xx} as a function of temperature T. **b**, Differential resistance dV_{xx}/dI as a function of temperature. **c**, Differential resistance dV_{xx}/dI as a function of DC current I and out-of-plane magnetic field $B_\perp$. SC5 is fully suppressed by the $B_\perp$ value (dashed line) in which SC1 at negative D shows the first side-peak of the interference pattern (Fig. R4r).*

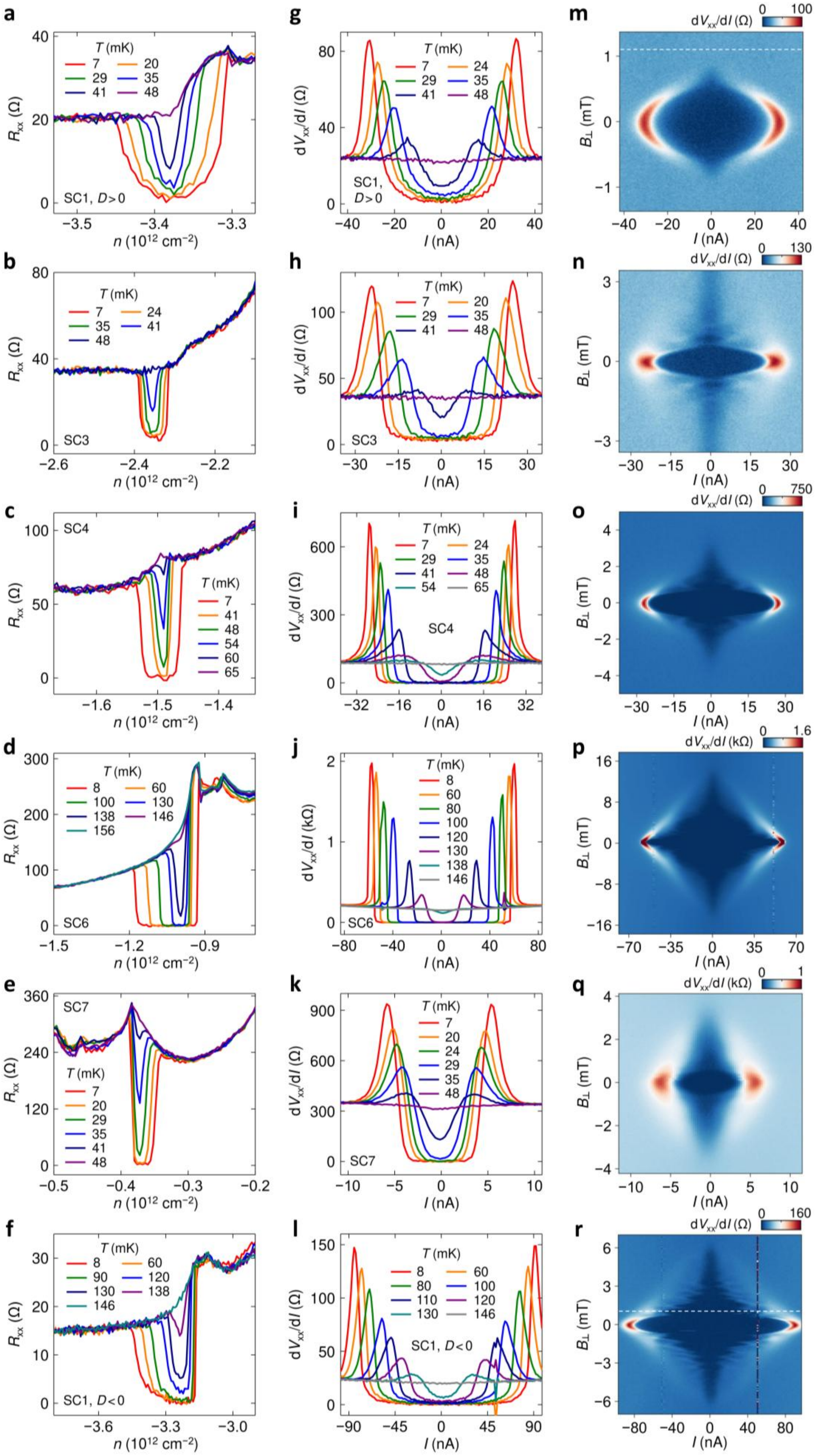
a
b
c
d
e
f
g
h
i
j
k
l
m
n
o
p
q
r
T (mK)
SC1, D > 0
SC3
SC4
SC6
SC7
SC1, D < 0
R_{xx} (Ω)
n (10^{12} cm^{-2})
dV_{xx}/dI (Ω)
dV_{xx}/dI (kΩ)
I (nA)
$B_\perp$ (mT)

***Figure R4. Detailed characterizations of SCs in R4G/WSe$_2$. a-f**, Resistance R_{xx} as a function of temperature T for SC1 at positive D (D/ε_0 = 0.469 V/nm, **a**), SC3 (D/ε_0 = 0.18 V/nm, **b**), SC4 (D/ε_0 = 0.072 V/nm, **c**), SC6 (D/ε_0 = 0.026 V/nm, **d**), SC7 (D/ε_0 = −0.22 V/nm, **e**), and SC1 at negative D (D/ε_0 = −0.42 V/nm, **f**). **g-l**, Differential resistance dV_{xx}/dI as a function of temperature SC1 at positive D ($n = -3.385 \times 10^{12}$ cm^{-2}, D/ε_0 = 0.469 V/nm, **g**), SC3 ($n = -2.356 \times 10^{12}$ cm^{-2}, D/ε_0 = 0.18 V/nm, **h**), SC4 ($n = -1.482 \times 10^{12}$ cm^{-2}, D/ε_0 = 0.072 V/nm, **i**), SC6 ($n = -1.00 \times 10^{12}$ cm^{-2}, D/ε_0 = 0.026 V/nm, **j**), SC7 ($n = -0.38 \times 10^{12}$ cm^{-2}, D/ε_0 = −0.22 V/nm, **k**), and SC1 at negative D ($n = -3.25 \times 10^{12}$ cm^{-2}, D/ε_0 = −0.42 V/nm, **l**). **m-r**, Differential resistance dV_{xx}/dI as a function of DC current I and out-of-plane magnetic field $B_\perp$ for SC1 at positive D (**m**), SC3 (**n**), SC4 (**o**), SC6 (**p**), SC7 (**q**), and SC1 at negative D (**r**). The (n, D/ε_0) points used in **m-r** are the same to those in **g-l**. SC1 at positive D (Fig. R4m) is fully suppressed by the $B_\perp$ value (dashed line) in which SC1 at negative D shows the first side-peak of the interference pattern (Fig. R4r).*

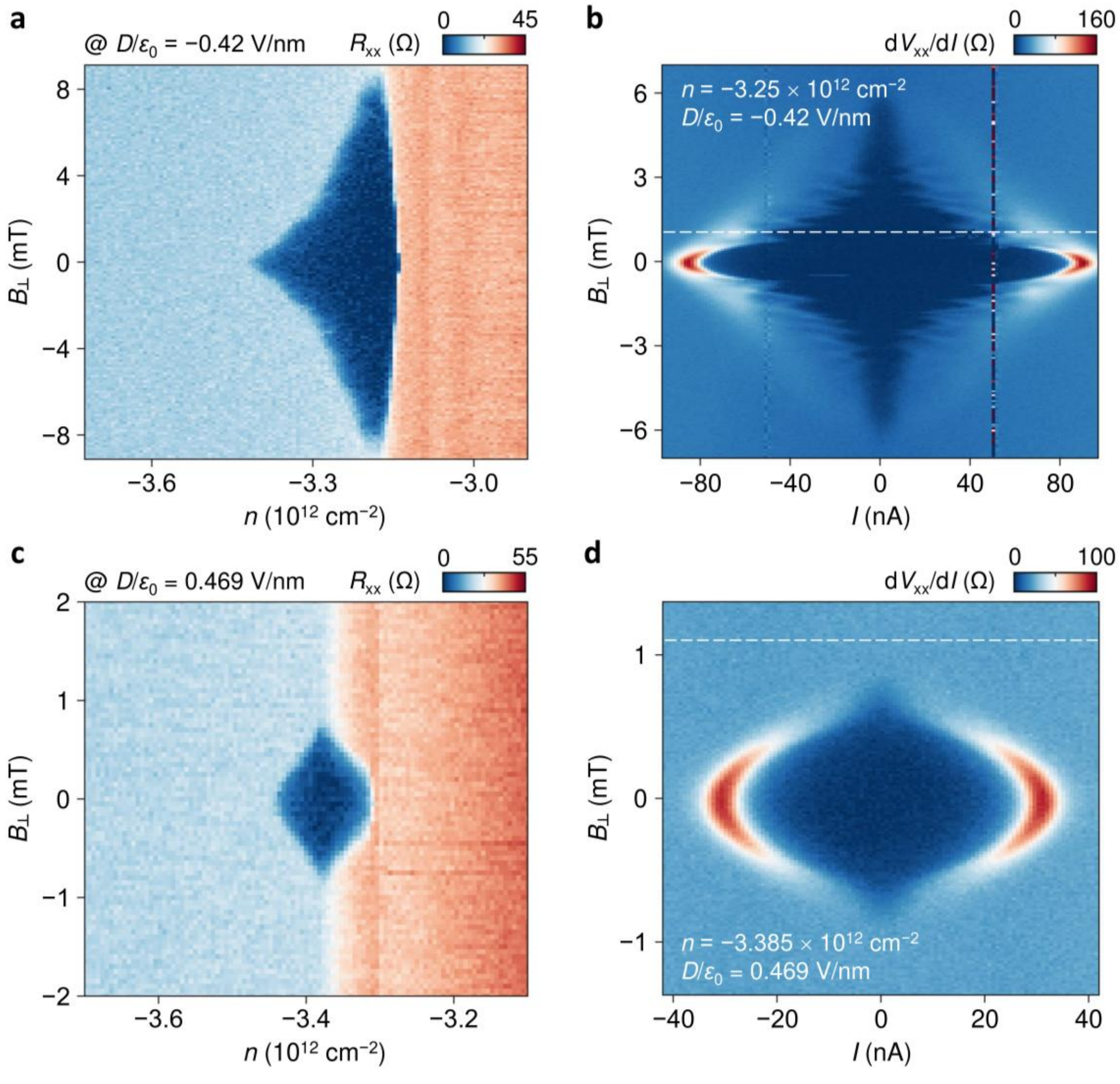

***Figure R5. Comparison of SC1 in R4G/WSe$_2$ on the WSe$_2$-distant and WSe$_2$-proximal side. a,** R_{xx} as a function of n and $B_\perp$ for the SC1 on the WSe$_2$-distant side. **b**, Differential resistance as a function of I and $B_\perp$ for the SC1 on the WSe$_2$-distant side. **c**, R_{xx} as a function of n and $B_\perp$ for the SC1 on the WSe$_2$-proximal side. **d**, Differential resistance as a function of I and $B_\perp$ for the SC1 on the WSe$_2$-proximal side. These data show the range of SC1 along the n axis, I_c, and $B_{c,\perp}$ all decrease when SC1 is placed to the WSe$_2$-proximal side. SC1 at positive D is fully suppressed by the $B_\perp$ value (dashed line) in which SC1 at negative D shows the first side-peak of the interference pattern.*

(13) Line 294: "suggesting spin-polarized instead of Ising SC". I don't think that's obvious. The scale of intrinsic Kane-Mele SOC in graphene (which acts as an Ising-type SOC when projected to a single layer by the displacement field) has been estimated using different techniques to be ~50 micro-eV or so. Assuming BCS scaling for simplicity, the gap size Delta~1.76 k_B T_c for the superconductors reported in this work are of the order (using T_c ~ 100 mK) ~15 micro-eV. So, in the absence of applied magnetic fields or other spin polarization, the system would be in a Ising-dominated regime from the perspective of pairing. Of course, that story changes if an in-plane field is applied or if the system develops ferromagnetic instabilities. In that case, the normal state is likely spin-canted (as discussed in Refs. 38, 53) with a large in-plane moment.

Response 14

We appreciate the referee for this point. The referee is correct about the SOC effect in the absence of applied magnetic fields or other spin polarization. However, the rhombohedral graphene system has a ferromagnetic instability by strong correlation effects, and this changes the story, as the referee also pointed out. The ferromagnetic instability is due to the Hund's coupling (few meV), which dominates the Kane-Mele SOC (tens of μeV) for a half-metal [C. L. Patterson et al. *Nature* **641**, 632-638 (2025)]. As discussed in **Response 10**, the two spins from each valley are therefore aligned to each other in the normal state already. In this regard, the system is not in an Ising-dominated regime but is consistent with the spin-polarized scenario.

We think the Hund's coupling is important in this discussion. Hence, we changed the line 294 in the previous manuscript as follows: "Firstly, in-plane critical field exceeding the Pauli limit has been reported in transition-metal dichalcogenides (TMDs)[46-49] and SOC-proximitized graphene[35,41]. There, strong Ising SOC pins the electron spins to the out-of-plane direction and makes the SC robust against $B_\parallel$. However, such Ising SCs are killed by high $B_\parallel$ before being fully spin-polarized. By contrast, SC2-4 are robust against $B_\parallel$ with ultrahigh PVR and arise from half-metals. Since the SOC of graphene is much weaker than Hund's coupling[37,50-52] favoring the spin polarization of half-metals, this suggests SC2-4 are spin-polarized differently from Ising SC."

(14) SM Fig 6: the perpendicular field-induced enhancement is much weaker in this second device. Do the authors have more data on B_perp enhancement in Device 2 to put the findings into context (comparing e.g. to the much more extensive SM Fig 5)? It would be good to ascertain reproducibility more thoroughly, given that this effect is the most unusual reported in this work.

Response 15

Yes, we agree that reproducibility is important. To ascertain the reproducibility of this observation, we showed this $B_\perp$-induced enhancement from additional R5G devices in Figure R6. The increase in the critical current I_c and the area in the *n*-*D* phase space could be reproduced. In response to the referee's comment, we added this additional data in Extended Data Figure 4 and 5.

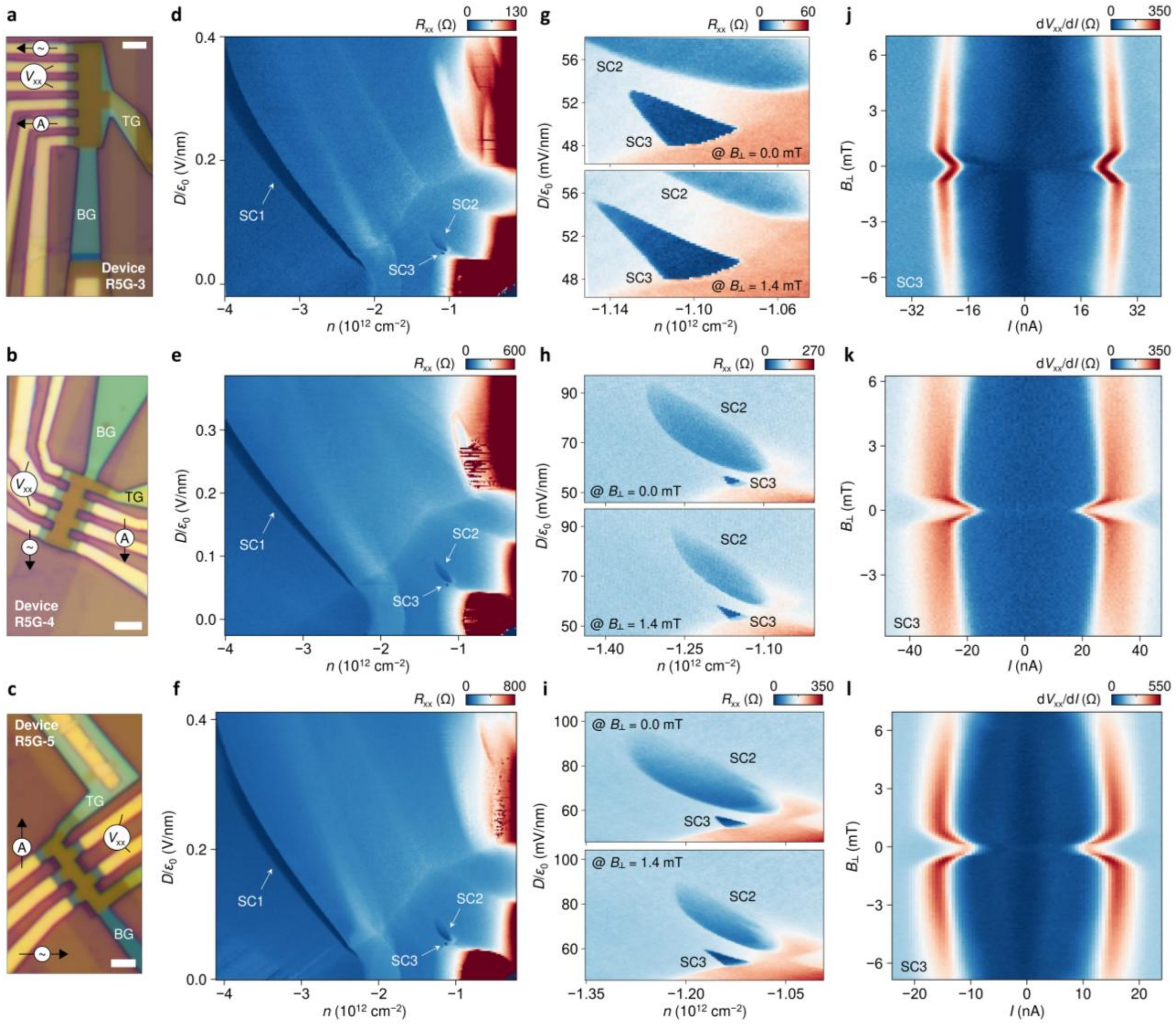

***Figure R6. Enhancement of SC by B$_\perp$ in additional R5G devices. a-c,** Optical image of the devices with the used measurement configurations. Scale bar: 3 μm.* ***d-f,** Longitudinal resistance R_{xx} as a function of n and D/ε_0 for Device R5G-3 (**d**), R5G-4 (**e**) and R5G-5 (**f**). All the three superconducting states at zero magnetic fields are reproduced.* ***g-i,** R_{xx} map at $B_\perp$ = 0.0 (top) and 1.4 mT (bottom) for Device R5G-3 (**g**), R5G-4 (**h**) and R5G-5 (**i**). The area of SC3 in the n-D space is increased by applying finite $B_\perp$.* ***j-l,** Differential resistance as a function of I and $B_\perp$ for SC3 Device R5G-3 ($n = -1.11 \times 10^{12}$ cm^{-2}, D/ε_0 = 50.6 mV/nm, **j**), R5G-4 ($n = -1.16 \times 10^{12}$ cm^{-2}, D/ε_0 = 55 mV/nm, **h**) and R5G-5 ($n = -1.143 \times 10^{12}$ cm^{-2}, D/ε_0 = 55.9 mV/nm, **i**). The critical current is increased by $B_\perp$.*

(15) SM Fig 8: what density and displacement was used for the B_parallel and B_perp evolution?

Response 16

The data in Extended Data Figure 8 in the previous manuscript was taken at (n, D/ε_0) = (-1.104×10^{12} cm^{-2}, 52 mV/nm) for the device R5G-1. We note that this original device was damaged during the shipping from the University of Basel to MIT. During the revision process, we took the equivalent set of data from another Device R5G-4, taken at (n, D/ε_0) = (-1.16×10^{12} cm^{-2}, 55 mV/nm), as shown in Figure R7. The sharp enhancement of SC3 happens only by $B_\perp$. In response to the referee's comment, data for Extended Data Figure 8 in the original manuscript is replaced with Figure R7 (as Extended Data Figure 9), and the

values of n and D/ε_0 are added.

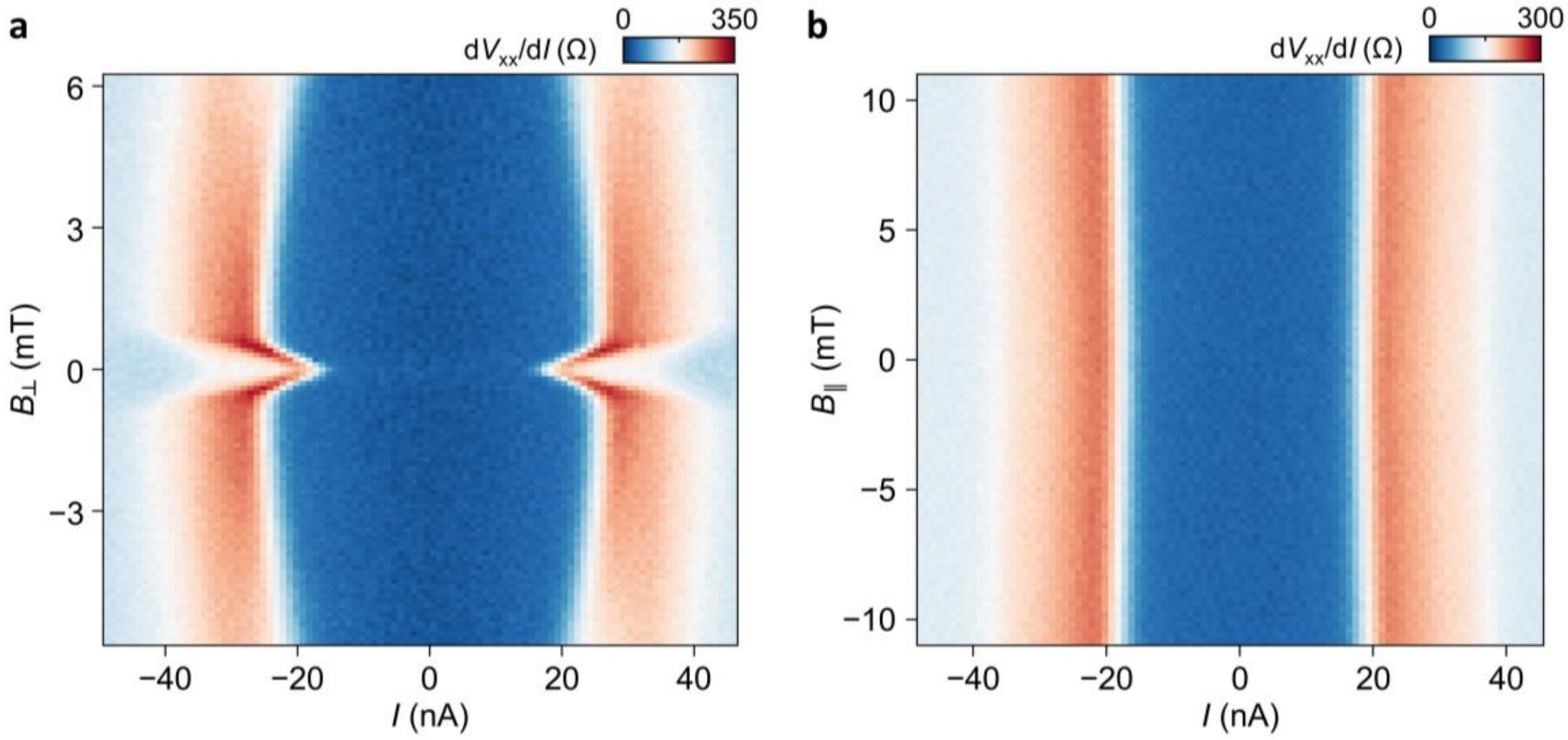

Figure R7. Differential resistance as functions of the drain-source current I and magnetic fields. a-b, *Differential resistance under an out-of-plane magnetic field* $B_\perp$ *(**a**) and an in-plane magnetic field* $B_\parallel$ *(**b**). The enhancement of SC3 appears only under* $B_\perp$*. The data was taken at* $(n, D/\varepsilon_0) = (-1.16 \times 10^{12}\ cm^{-2}, 55\ mV/nm)$.

Finally, I have an optional suggestion for the authors, which I am aware may go beyond the scope of their work:

(16) Do the authors have similar data on the B_parallel and B_perp field evolution of the superconductors in the R4G/WSe2 proximitized device? It would be a very interesting comparison to the (in-plane and out-of-plane) B-field enhancements reported in Figs 1,2,3 — and perhaps tie the story together. Coming back to point (13) above: as far as the spin structure of the normal state is concerned, the distinction between hBN and WSe2-encapsulated devices may be primarily the magnitude of the anisotropy induced by Ising SOC. In the hBN case the system is more isotropic and the spin polarization can follow the direction of applied magnetic fields more easily than with WSe2, where there is a ~1 meV-scale easy-axis anisotropy to fight against. Such measurements could also test whether the spin-canted phenomenology reported in the normal state of R3G/WSe2 in Ref. 38 is also relevant here in R4G/WSe2, possibly supporting or ruling out spin-fluctuation mechanisms as in https://arxiv.org/abs/2406.17036 and follow-up theory works.

Response 17

We appreciate this suggestion and have followed it by measuring the in-plane field response of SCs in the R4G/WSe_2 device during the revision. The data for SC1 to SC7 are shown in Figure R8-R12, from the positive to negative values of D. We found all of these SCs are monotonically suppressed by applying $B_\parallel$. As for the response to $B_\perp$, the data for SC5 and the rest of the SCs in R4G/WSe_2 are shown in Figure R3c and R4m-r, respectively, in **Response 13**. No enhancement of SC by $B_\perp$ was observed in any of the SCs in R4G/WSe_2.

We do not have the capability to perform a magnetometry experiment such as nano-SQUID (superconducting quantum interference device). Therefore, we could not determine the spin structure of phases of interest in Device R4G/WSe_2 and test theories such as the spin-fluctuation mechanism. We think this is beyond the scope of the current manuscript, as the referee suggested, and such measurement will be an interesting future direction.

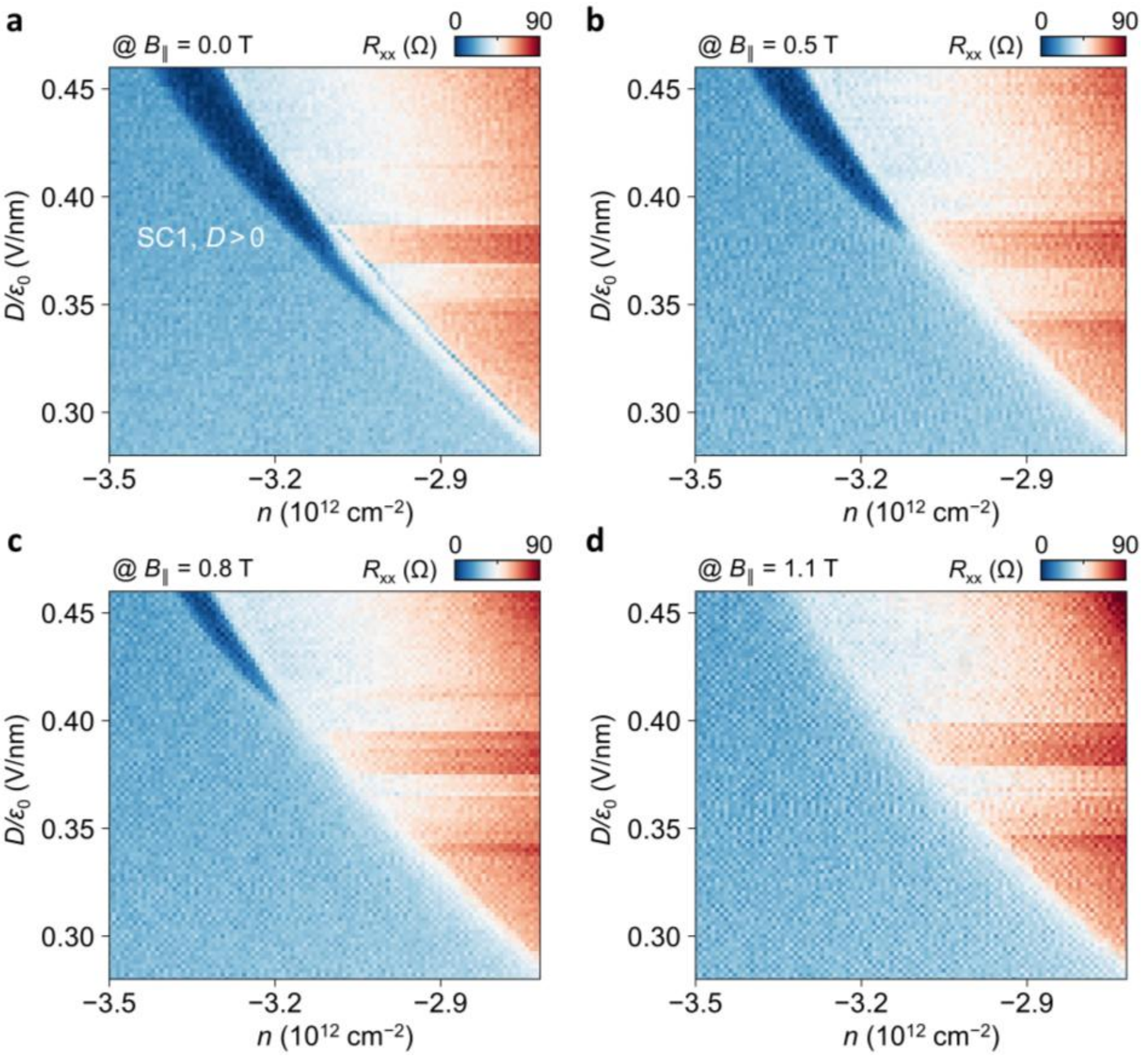

***Figure R8. In-plane magnetic field response of SC1 at positive D in Device R4G/WSe₂. a-d**, The data were taken at $B_\parallel$ = 0.0 (**a**), 0.5 (**b**), 0.8 (**c**), and 1.1 T (**d**). SC1 at positive D monotonically suppressed by $B_\parallel$.*

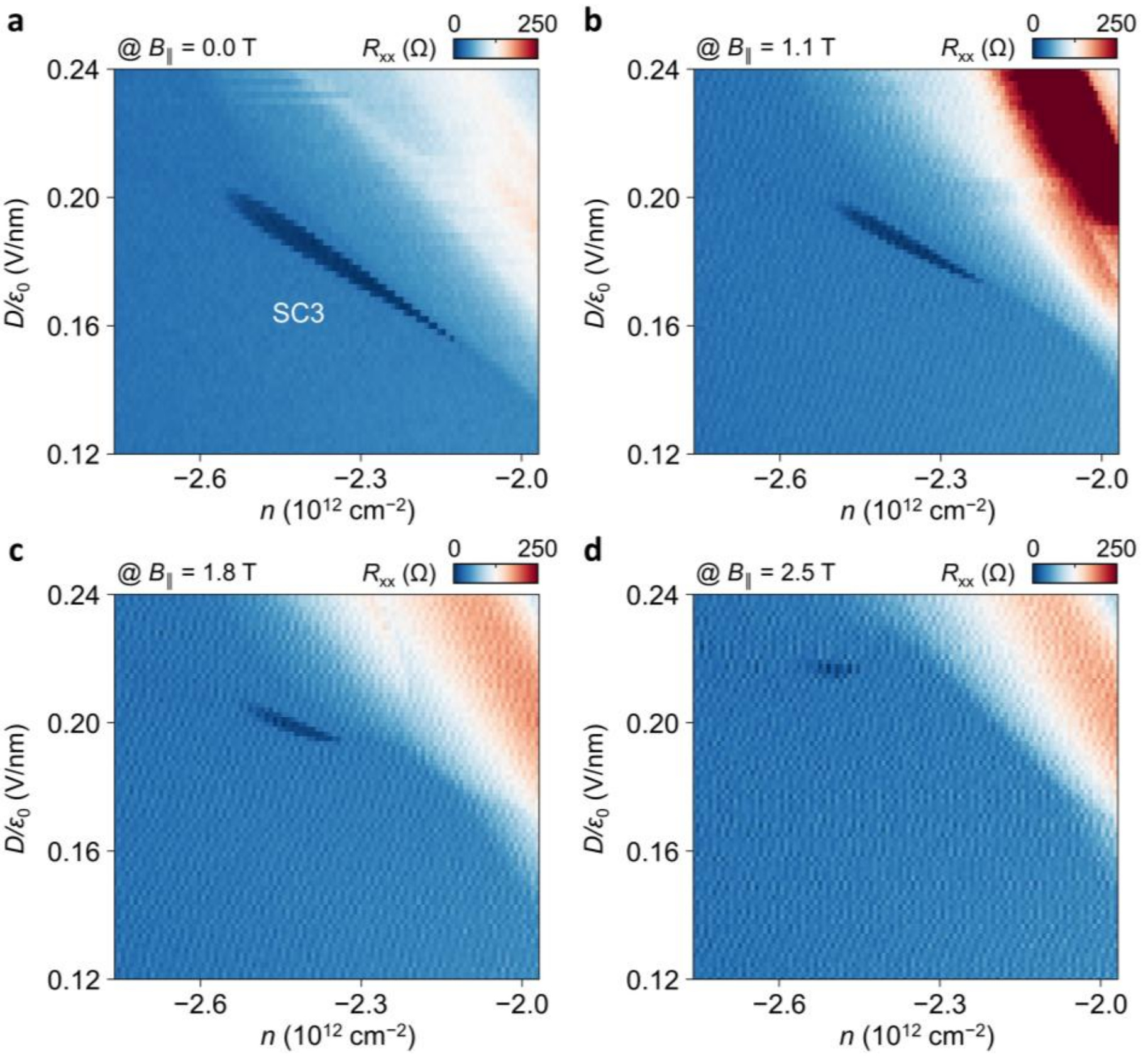

***Figure R9. In-plane magnetic field response of SC3 in Device R4G/WSe₂. a-d**, The data were taken at $B_\parallel$ = 0.0 (**a**), 1.1 (**b**), 1.8 (**c**), and 2.5 T (**d**). SC3 is monotonically suppressed by $B_\parallel$.*

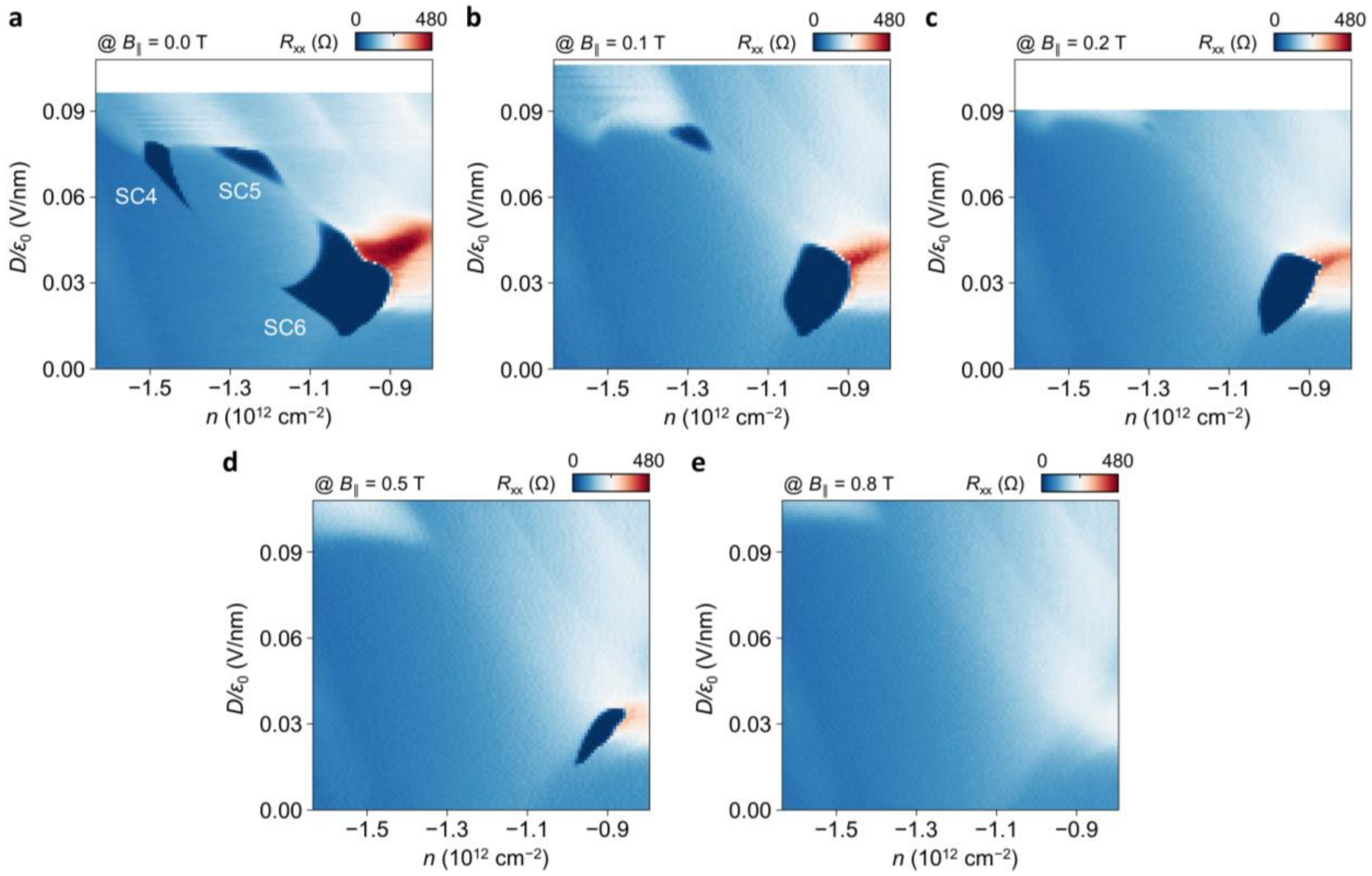

***Figure R10. In-plane magnetic field response of SC4, SC5 and SC6 in Device R4G/WSe₂. a-e**, The data were taken at $B_\parallel$ = 0.0 (**a**), 0.1 (**b**), 0.2 (**c**), 0.5 (**d**) and 0.8 T (**e**). SC4, SC5 and SC6 are suppressed by $B_\parallel$.*

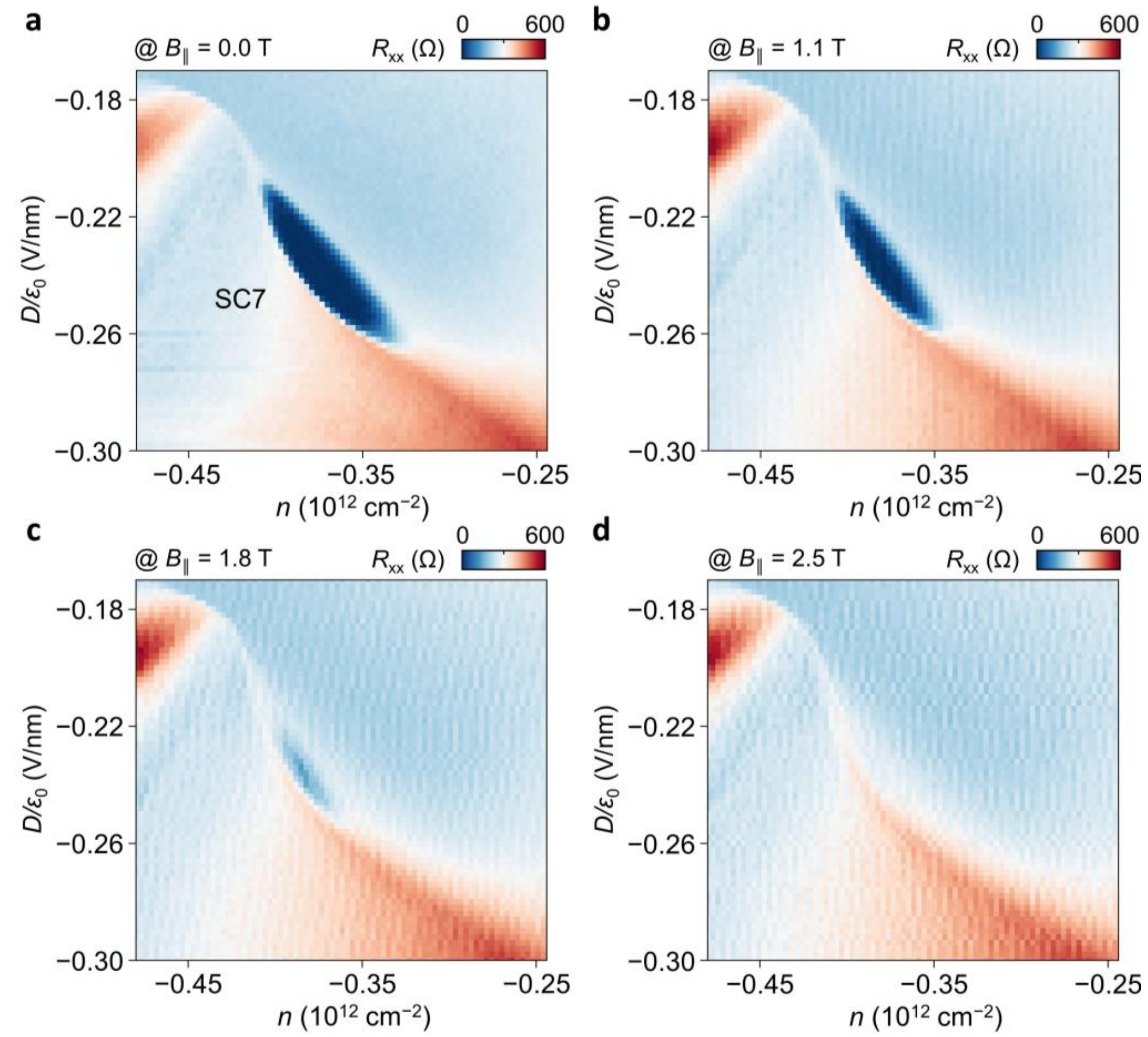

***Figure R11. In-plane magnetic field response of SC7 in Device R4G/WSe₂. a-d**, The data were taken at $B_\parallel$ = 0.0 (**a**), 1.1 (**b**), 1.8 (**c**), and 2.5 T (**d**). SC7 is monotonically suppressed by $B_\parallel$.*

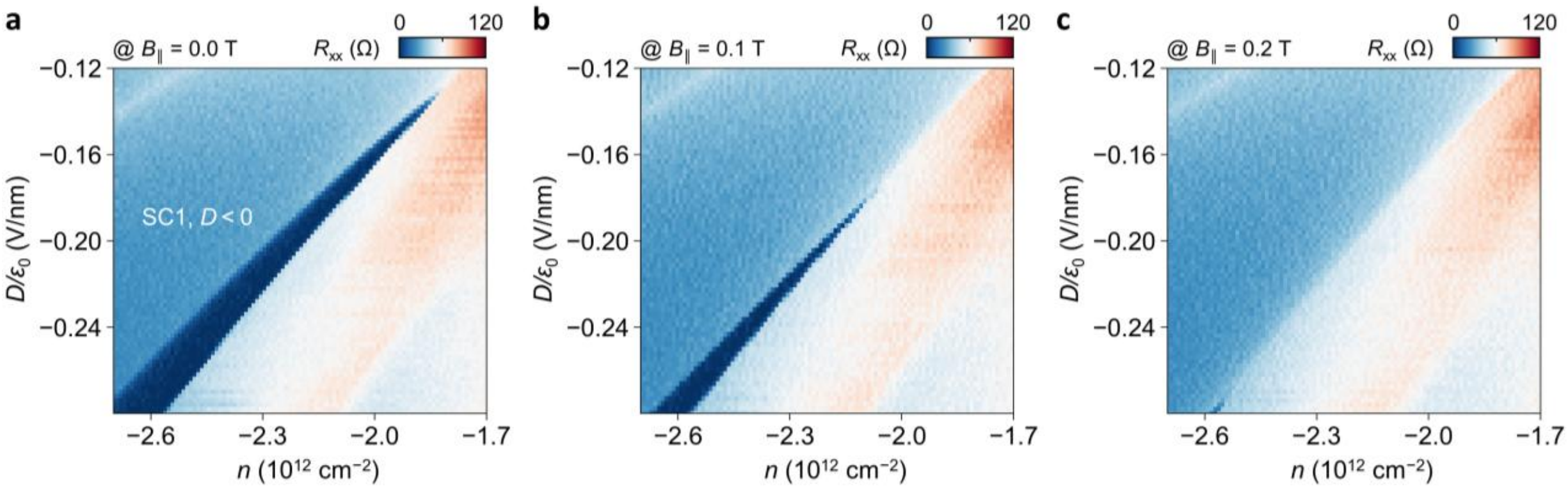

***Figure R12. In-plane magnetic field response of SC1 at negative D in Device R4G/WSe$_2$. a-c**, The data were taken at $B_\parallel$ = 0.0 (**a**), 0.1 (**b**), and 0.2 T (**c**). SC1 at negative D is monotonically suppressed by $B_\parallel$ at a lower value compared to SC1 at positive D (Fig. R8). This may be attributed to the weaker Ising SOC effect.*

Referee #2 (Remarks to the Authors):

The manuscript reports on the observation of superconductivity in rhombohedral tetralayer and pentalayer graphene. While the exploration of superconductivity in graphene-based systems remains a topic of significant interest, I have major reservations regarding the novelty, the strength of the evidence, and the depth of insight provided by this study. This work appears preliminary and does not meet the high standard of significance required for publication in Nature. The claims are not sufficiently substantiated, and the analysis lacks the rigor needed to advance the field.

Response 18

We sincerely appreciate the referee's time to evaluate the manuscript, and pointing out '*the exploration of superconductivity in graphene-based systems remains a topic of significant interest*.' We tried our best below to address the comments and questions made by the referee.

Major Concerns:

1. The report of superconductivity in rhombohedral (R) graphene is not new, as it has already been established in R3G, R4G, and R5G systems by prior studies, including work from this same group. Therefore, the central finding of this manuscript—the existence of superconductivity in R4G and R5G—is rather not a discovery of new superconducting systems.

Response 19

We respectfully disagree with the referee's comments 'The report of superconductivity in rhombohedral (R) graphene is not new' and 'is rather not a discovery of new superconducting systems.' In conventional superconductivity research, a particular material usually hosts one or two superconducting states. In two-dimensional materials such as rhombohedral graphene, although it is the same chemical composition and structure, each combination of n and D gives effectively a new material to explore in the conventional sense. This is an exciting platform for superconductivity, since many different superconducting states can be explored, studied, and compared in the same chemical material and switched conveniently by electrostatic gating. Previous literatures indeed report certain superconducting states in rhombohedral multilayer graphene, but the superconducting states we reported in this work have clear new ingredients.

They should not be simply categorized as 'not new' due to the same rhombohedral graphene platform, and should instead be interpreted as new superconductors−contrary to what the referee said.

Specifically, we would like to recap on our main findings and how they are different from previously reported superconductivities in graphene and other superconductors. Firstly, we observed new superconducting states SC2 and SC4 in R5G, which are enhanced and induced by $B_{\parallel}$, respectively. Only a few examples such as uranium-based compounds, organic superconductors and $Eu_xSn_{1-x}Mo_6S_8$ have demonstrated similar phenomena, and they all host localized magnetic moments that can explain the enhancements. In contrast, rhombohedral graphene does not contain magnetic elements, suggesting that SC2 and SC4 in R5G have fundamental difference from such materials. Among two-dimensional materials, bilayer graphene (R2G) has shown field-induced SC [H. Zhou et al. *Science* **375**, 774-778 (2022)], but the demonstrated PVR is lower than SC2 and SC4 in R5G. Secondly, we observed a new superconducting state SC3 in R5G, which is enhanced by $B_{\perp}$. This is even rarer and further from conventional understanding, **as pointed out by this referee later and by Referee 1**. Except for impurity effects, the only SC that has shown a similar enhancement is the chiral SC in electron-doped rhombohedral graphene [T. Han et al. *Nature* **643**, 654-661 (2025)]. In that case, the magnetic enhancement could be relatively easily understood, since the parent state has orbital magnetism that can directly be coupled to an out-of-plane field. In stark contrast, SC3 is developed from a half-metal state without the spontaneous valley polarization. This implies the different origin of $B_{\perp}$-induced enhancement of SC3. We could not find an existing theory to explain this phenomenon−signifying the novelty.

The authors have not articulated what new physical principle or conceptual understanding this work provides beyond the existing literature. It remains primarily a report on specific devices without delving into new underlying mechanisms that would justify its publication in a high-impact journal. The study does not move the field forward meaningfully.

Response 20

First of all, we do have new observations in this work compared to the previous studies. Especially, as Referee 1 pointed out 'enhancement of pairing with out-of-plane magnetic fields B_perp... hasn't been convincingly demonstrated before to my knowledge,' the enhancement of SC by $B_{\perp}$ is physics definitely beyond the existing literature.

Secondly, our work is not just a report on *specific* devices. The revised manuscript is based on eight devices in total and thus provides the great reproducibility of the reported phenomena. This point is emphasized in **Response 27** in more detail.

Thirdly, we disagree that reporting new superconducting states 'does not move the field forward meaningfully' or only 'new underlying mechanisms that would justify its publication in a high-impact journal'. It is needless to say how much the discovery of a high-T_c superconductor has revolutionized modern condensed matter physics. The recent example of magic-angle twisted bilayer graphene reported by the Jarillo-Herrero group also had huge impact in the field. Both works reported phenomenologically new superconductors, without providing 'new physical principle or conceptual understanding.' Even today, the mechanisms of superconductivity in these two systems are still not clear. Our work falls into the same category, by reporting SCs that show unconventional characteristics that have not been observed or investigated in previous SC literatures.

Nevertheless, to be constructive, we took the suggestion on expanding our discussion on possible underlying mechanism, as can be seen in detail in **Response 25**.

2. The central claim of superconductivity is not fully supported by the data, which lacks several critical components expected for a conclusive demonstration.

For states other than SC1, the resistivity does not reach zero, which is a fundamental hallmark of a superconducting transition. This raises serious doubts about the nature of these phases.

The critical current-like behavior observed in dI/dV-I measurements could potentially be explained by non-superconducting out-of-equilibrium phenomena. The authors do not adequately rule out such alternative interpretations.

Response 21

We will respond accordingly if the referee can specify what kind of 'non-superconducting out-of-equilibrium phenomena' are referred to. We are guessing that one trivial scenario giving rise to nonlinear dV_{xx}/dI is the Joule heating from the DC current. If this were the case, however, dV_{xx}/dI would be a monotonic function of I instead of showing well developed peaks. This case is well explained in the reference [M. He et al. *Nat. Phys.* **17**, 26-30 (2021)].

As for the nonzero resistance, the R_{xx} of SC3 at zero magnetic fields and SC2 under high $B_{\parallel}$ approach 0 Ω and close to 0 Ω in the new device measured during the revision, as shown in Figure R13a&b. We would like to note that electrical transport measurements for small graphene devices at ultralow temperatures suffer from technical issues such as insufficient noise filtering and contact resistance, as pointed out by Referee 3 (**Response 36**). These cause the disequilibrium of the electron system with the lattice and can lead to nonzero resistance [L. Holleis et al. Nat. Phys. 21, 444-450 (2025)]. This problem of small residual resistance is fairly common for a mesoscopic 2D superconductor, (eg. Fig. 4c-e of [H. Zhou et al. Nature 598, 434-438 (2021)], Fig. 1e of [L. Holleis et al. Nat. Phys. 21, 444-450 (2025)], Fig. 4b of [Y. Zhang et al. Nature 641, 625-631 (2025)], Fig. 3b&4b of [Y. Guo et al. arXiv:2511.17423v1 (2025)]). Even in the most robust superconductivity, SC1, in rhombohedral graphene, sometimes the resistance does not reach zero. Figure R13c shows such an example in which the resistance of SC1 from Device R5G-4 does not strictly touch 0 Ω, in contrast to that from Device R5G-1, R5G-2 (Figure R13d) and R5G-3. Please note that the SC1 from Device R5G-4 nevertheless shows Fraunhofer-like interference patterns (Figure R14c), supporting the existence of the phase-coherent transport of a superconductor.

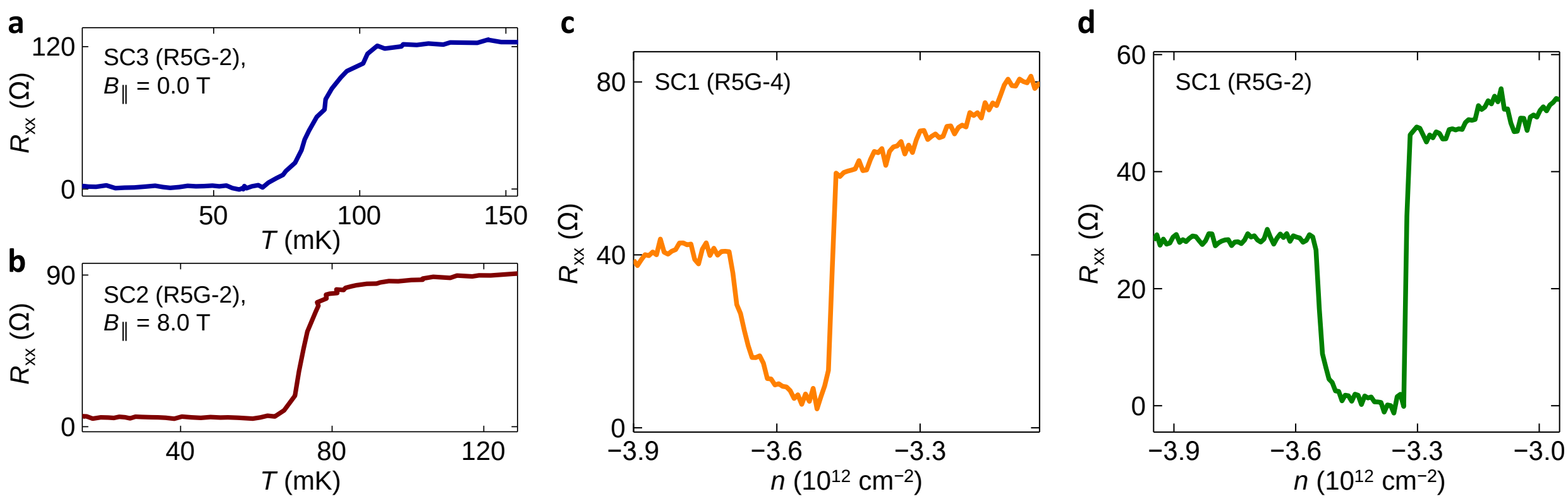

Figure R13. Tackling the issue of nonzero residual resistance in SCs in R5G. a,b, *Temperature-dependent R_{xx} of SC3 at $B_{\parallel} = 0.0$ T ($n = -1.100 \times 10^{12}$ cm^{-2}, $D/\varepsilon_0 = 49.61$ mV/nm,* ***a****) and SC2 at $B_{\parallel} = 8.0$ T ($n = -1.055 \times 10^{12}$ cm^{-2}, $D/\varepsilon_0 = 93.17$ mV/nm,* ***b****). The resistance of SC3 and SC3 approaches 0 Ω and around 0 Ω below the transition temperature, respectively.* ***c,d,*** *Comparison of SC1 from Device R5G-4 ($D/\varepsilon_0 = 0.225$ V/nm,* ***c****) and R5G-2 ($D/\varepsilon_0 = 0.214$ V/nm,* ***d****). Device R5G-2 shows 0 Ω while R5G-4 does not.*

A cornerstone experiment for confirming superconductivity in a 2D device is the characterization of the critical current or Josephson junction behavior under a perpendicular magnetic field, leading to Fraunhofer-like interference patterns. The absence of this standard characterization is a critical omission that severely weakens the superconducting claim.

Response 22

We completely agree with the referee on the importance of observing Fraunhofer-like interference patterns for claiming SC, and we truly appreciate this great suggestion to improve our manuscript. We have followed the suggestion to perform such measurements for all the SC states reported in our original manuscript. In short, we could observe interference patterns for most of the states reported in this work: SC1 (Fig. R14a) in R4G; SC1 (Fig. R14b&c), SC2 (Fig. R14d-f) and SC3 (Fig. R14g&h) in R5G; SC1 (Fig. R14i), SC3 (Fig. R14j), SC4 (Fig. R14k) and SC6 (Fig. R14l) in R4G/WSe_2. The observation of these interference patterns further supports the superconducting nature of the claimed states.

For the other SC states, we could find weak or no interference patterns as shown in Figure R15. We suspect two reasons for the lack of or weak Fraunhofer patterns. Firstly, crystalline graphene is a clean material system and thus exhibits small spatial inhomogeneity. There can be non-SC islands in a micron-size device due to sample inhomogeneity, even when (n, D) are tuned to a superconducting phase. The phase-coherent transport is affected by the Aharonov-Bohm phase defined by this non-SC area and thus the interference pattern appears. In this regard, the pattern becomes less obvious when the sample is more homogeneous. This is also the case for twisted graphene superconductors. Compared to magic-angle twisted bilayer graphene (Figure R16a), twisted multilayer graphene takes advantage of angle-locking effect [S. Turkel et al. *Science* **376**, 193-199 (2022)] and shows less clear interference patterns (see Figure R16b-d for examples) [Z. Zhou et al. *Nat. Phys.* **21**, 1773-1779 (2025); Y. Zhang et al. *Science* **377**, 1538-1543 (2022)]. Secondly, a SC state with a low out-of-plane critical field can be killed even before the interference pattern is developed. Figure R15 shows the comparison between the SC states in the same sample with a clear (R15a, R15c & R15e) and weak/no (R15b, R15d & R15f-h) interference pattern. When the first side-peak of the interference pattern appears (indicated as the dashed line), the resistance of the SC with a weak pattern already recovers the normal-state R_{xx} mostly. This was also the case for twisted bilayer graphene with a low out-of-plane critical field (Figure R16e). To visualize a clearer interference pattern for these SCs, devices with a much more delicate design such as gate-defined Josephson junctions are necessary. Such gate-defined Josephson junctions in dual-gated graphene devices require significant experimental efforts and are beyond the scope of the current work.

In response to the referee's comment, we added the discussion about the observation of interference patterns in the Methods section as "Fraunhofer-like interference patterns and nonzero residual resistance": "Fraunhofer-like interference patterns that arise from phase-coherent transport can serve as additional evidence for SC. There can be non-SC islands in a micron-size device due to sample inhomogeneity, even when (n, D) are tuned to a superconducting phase. The phase-coherent transport is affected by the Aharonov-Bohm phase and thus the interference pattern appears. We found Fraunhofer-like interference patterns and periodic modulations of SC by $B_\perp$ in most of superconducting states reported here: SC1 (Extended Data Fig. 6c) in R4G; SC1 (Extended Data Fig. 3e and 5f), SC2 (Extended Data Fig. 3f, 5g and 5h), SC3 (Extended Data Fig. 5i and 8i) in R5G; SC1 (Fig. 4g), SC3 (Fig. 4b), SC4 (Fig. 4c), SC6 (Fig. 4e) in R4G/WSe_2. We note that a very limited number of crystalline rhombohedral graphene devices[36,37] have demonstrated Fraunhofer-like patterns in their superconducting states. This is in contrast to moiré graphene devices[18-20,22,65,66] in which Fraunhofer-like patterns could be observed more universally and were more pronounced. Possible reasons are (1) less inhomogeneity in a crystalline graphene system and (2) a lower out-of-plane critical field that kills SC even before the interference pattern is developed. For

example, SC1 at negative D in R4G/WSe2 shows the first side-peak at $B_\perp$ ≈ 1.1 mT (Fig. 4g). However, SC5 (Fig. 4d) and SC1 at positive *D* (Extended Data Fig. 10s) are already killed at this value of $B_\perp$ and the interference patterns do not appear."

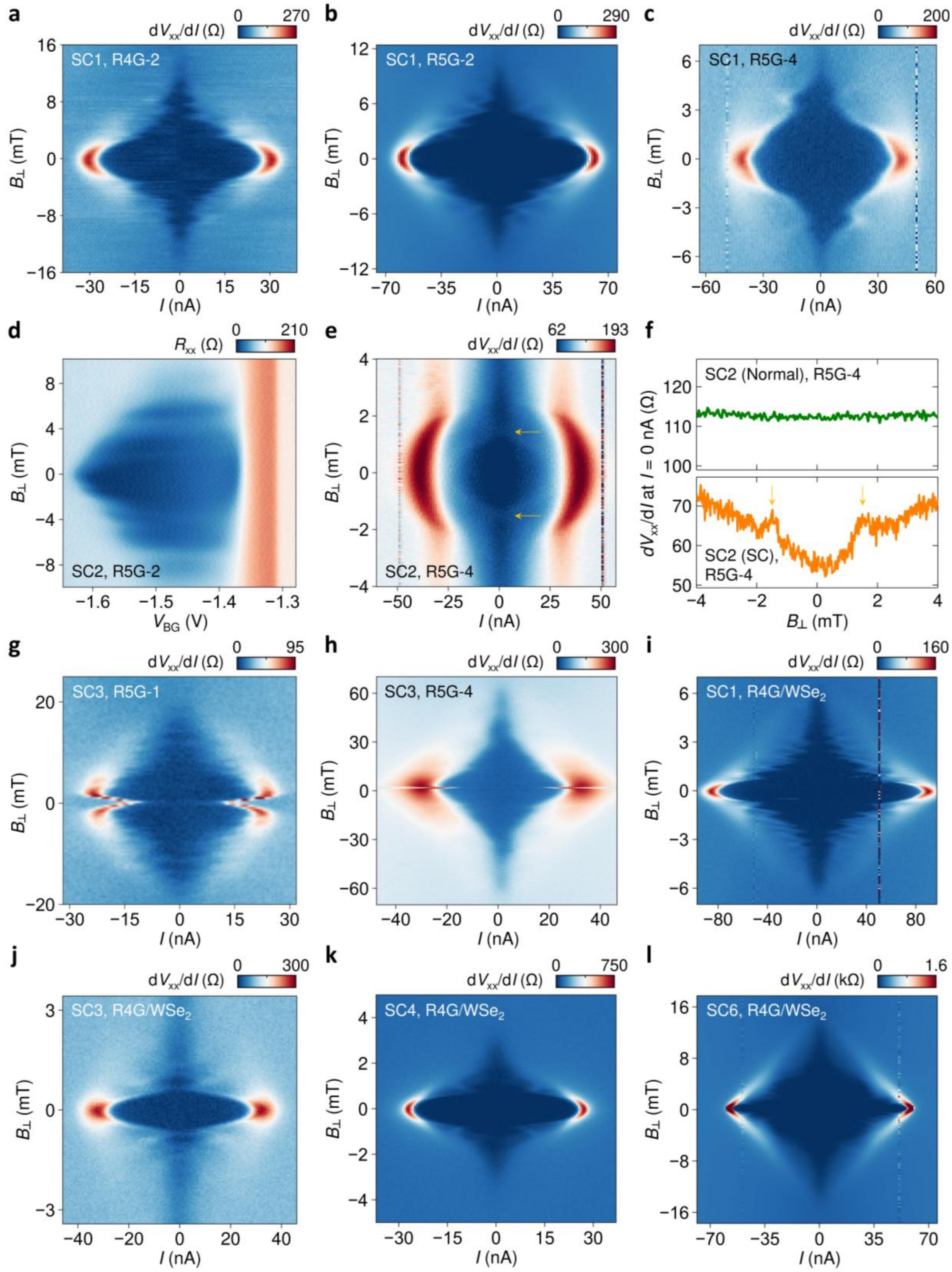

Figure R14. Fraunhofer-like interference patterns. ***a**, Differential resistance as a function of I and $B_\perp$ for SC1 in Device R4G-2 ($n = -2.68 \times 10^{12}$ cm^{-2}, $D/\varepsilon_0 = 0.28$ V/nm).* ***b,c,** Differential resistance as a function of I and $B_\perp$ for SC1 in Device R5G-3 ($n = -3.38 \times 10^{12}$ cm^{-2}, $D/\varepsilon_0 = 0.214$ V/nm,* ***b**) and Device R5G-4 (n = −3.57*

$\times 10^{12}$ cm^{-2}, D/ε_0 = 0.225 V/nm, **c**). ***d,*** R_{xx} as a function of V_{BG} and $B_\perp$ for SC2 in Device R5G-2 (V_{TG} = −0.2 V). The oscillation of R_{xx} by $B_\perp$ is observed. ***e,*** Differential resistance as a function of I and $B_\perp$ for SC2 in Device R5G-3 (n = −1.17 × 10^{12} cm^{-2}, D/ε_0 = 67 mV/nm). Small increase in the threshold current from $B_\perp$ of ~2 to ~4 mT is observed. ***f,*** Differential resistance at zero DC current for SC2 in Device R5G-3 (n = −1.17 × 10^{12} cm^{-2}, D/ε_0 = 67 mV/nm) in its normal state (top panel) and superconducting state (bottom panel). The modulation by $B_\perp$, highlighted with yellow arrows corresponding to those in ***e***, only exists below the SC transition temperature. This implies the modulation of dV_{xx}/dI is inherent in the superconducting state. ***g,h,*** Differential resistance as a function of I and $B_\perp$ for SC3 in Device R5G-1 (n = −1.135 × 10^{12} cm^{-2}, D/ε_0 = 57 mV/nm, ***g***) and Device R5G-4 (n = −1.16 × 10^{12} cm^{-2}, D/ε_0 = 55 mV/nm, ***h***). ***i-l,*** Differential resistance as a function of I and $B_\perp$ for SC1 (n = −3.25 × 10^{12} cm^{-2}, D/ε_0 = −0.42 V/nm, ***i***), SC3 (n = −2.356 × 10^{12} cm^{-2}, D/ε_0 = 0.18 V/nm, ***j***), SC4 (n = −1.482 × 10^{12} cm^{-2}, D/ε_0 = 72 mV/nm, ***k***) and SC6 (n = −1.00 × 10^{12} cm^{-2}, D/ε_0 = 26 mV/nm, ***l***) in Device R4G/WSe_2. All these data further corroborate the formation of superconducting states.

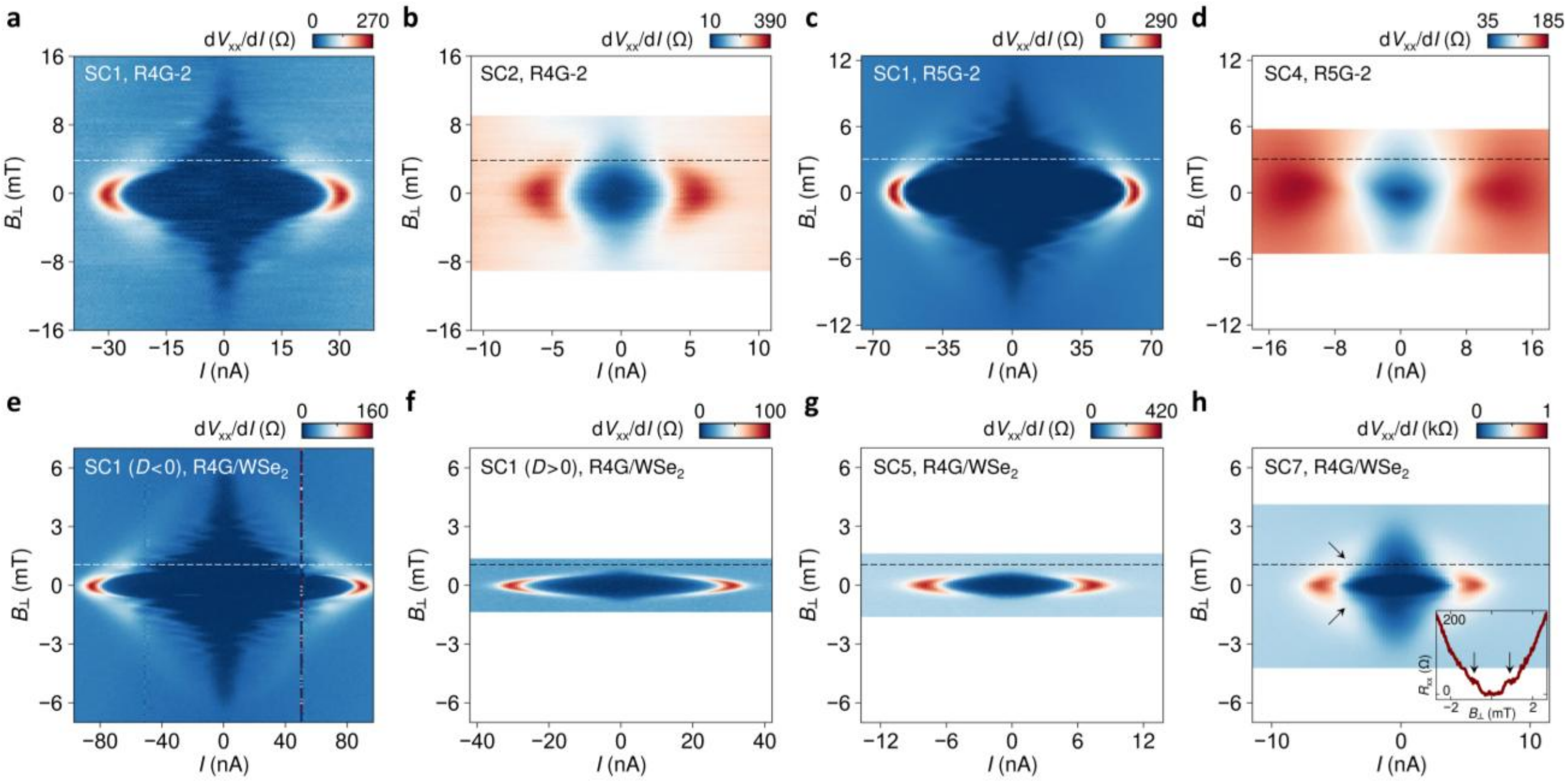

Figure R15. Comparison to cases with weak/no interference patterns. a,b, *Differential resistance as a function I and* $B_\perp$ *for SC1 (n = −2.68 ×* 10^{12} cm^{-2}*,* D/ε_0 *= 0.28 V/nm,* ***a****) and SC2 (n = −0.842 ×* 10^{12} cm^{-2}*,* D/ε_0 *= 6.2 mV/nm,* ***b****) in Device R4G-2. The dashed lines denote* $B_\perp$ *at which the first side-peak of interference patterns in* ***a*** *appears. SC2 mostly recovers the normal-state resistance at this line, indicating SC2 is killed before the interference pattern might be developed.* ***c,d,*** *Differential resistance as a function of for I and* $B_\perp$ *for SC1 (n = −3.38 ×* 10^{12} cm^{-2}*,* D/ε_0 *= 0.214 V/nm,* ***c****) and SC4 (n = −0.606 ×* 10^{12} cm^{-2}*,* D/ε_0 *= 0.144 V/nm,* ***d****) in Device R5G-3. The dashed lines denote* $B_\perp$ *at which the first side-peak of interference patterns in* ***c*** *appears. SC4 mostly recovers the normal-state resistance at this line, indicating SC2 is killed before the interference pattern might be developed.* ***e-j,*** *Differential resistance as a function I and* $B_\perp$ *for SC1 at negative D (n = −3.25 ×* 10^{12} cm^{-2}*,* D/ε_0 *= −0.42 V/nm,* ***e****), SC1 at positive D (n = −3.385 ×* 10^{12} cm^{-2}*,* D/ε_0 *= 0.469 V/nm,* ***f****), SC5 (n = −1.242 ×* 10^{12} cm^{-2}*,* D/ε_0 *= 72 mV/nm,* ***g****) and SC7 (n = −0.38 ×* 10^{12} cm^{-2}*,* D/ε_0 *= −0.24 V/nm,* ***h****). The dashed lines denote* $B_\perp$ *at which the first side-peak of interference patterns in* ***e*** *appears. SC1 at positive D and SC5 recover the normal-state resistance at this line, indicating these SCs are killed before the interference pattern might be developed.* ***Inset****: SC7 develops weak kinks near the current-induced transition and plateaus at* dV_{xx}/dI *at I = 0 nA (i.e.,* R_{xx}*) around the* $B_\perp$ *value of the dashed line, showing the signatures of weak interference patterns.*

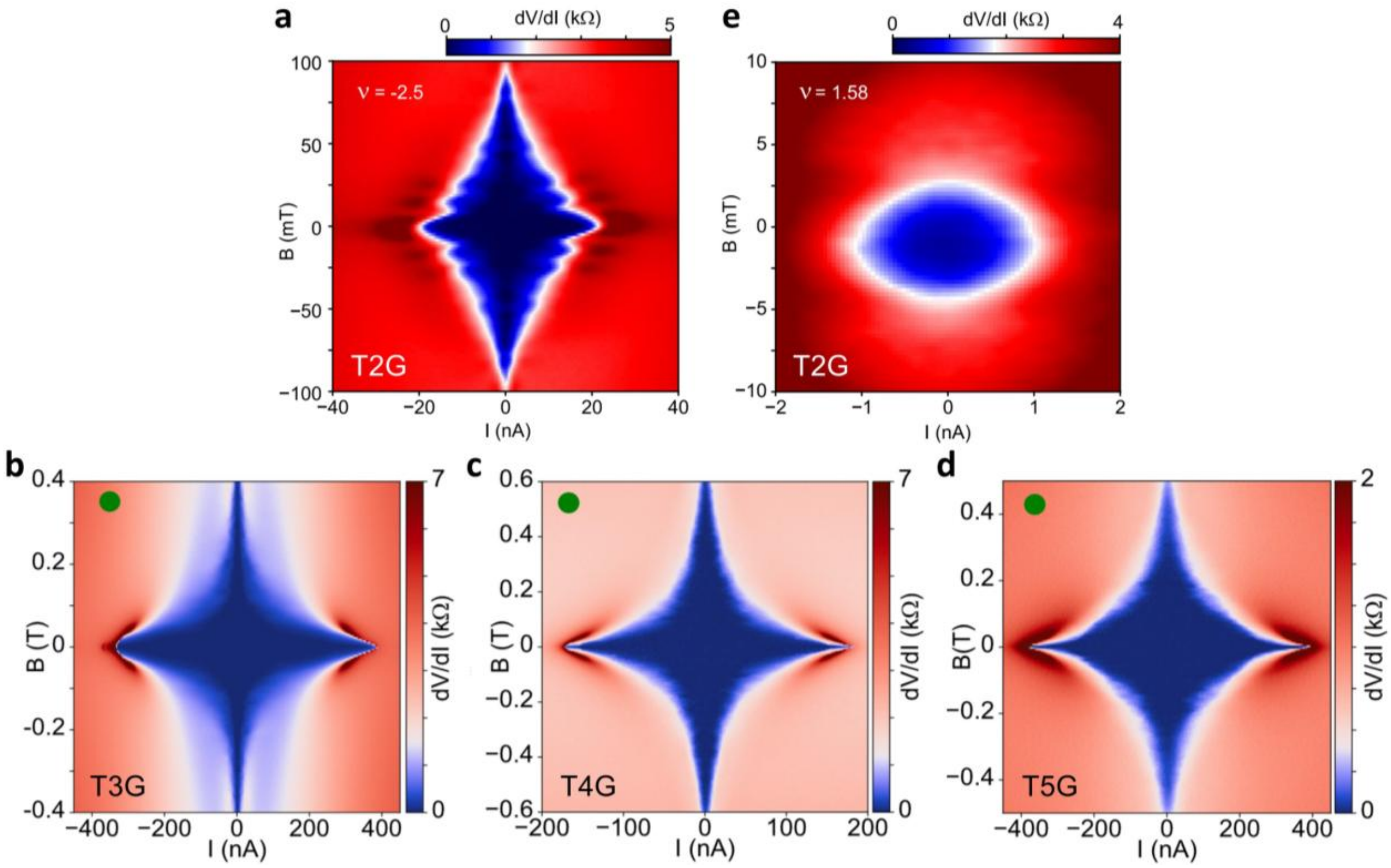

Figure R16. Examples of interference patterns in twisted N-layer graphene (TNG). a-d, *Differential resistance as a function of the DC current I and out-of-plane magnetic field B for twisted bilayer (**a**), trilayer (**b**), tetralayer (**c**) and pentalayer (**d**) graphene with WSe_2 at proximity. When the out-of-plane critical field is high enough, T2G shows a pronounced interference pattern, whereas the thicker twisted graphene does not.* ***e,*** *Differential resistance as a function of I and B for the same T2G sample with a lower out-of-plane critical field. The interference pattern is not observed. The data in **a**&**e** are adapted from [H. S. Arora et al. Nature **583**, 379-384 (2020)], and the data in **b**-**d** are adapted from [Y. Zhang et al. Science **377**, 1538-1543 (2022)].*

Even if one were to accept the superconducting claims, the reported critical parameters (current, field, temperature) are inferior to those already documented in other graphene systems, further questioning the significance of the finding.

Response 23

We respectfully disagree with this comment. The Pauli-limit violation ratio (PVR), which is an extremely important parameter that assesses the robustness of a SC under the in-plane field, is much higher for SC2-4 in R5G than what has been experimentally demonstrated in other graphene systems. As explained in the abstract of the manuscript, they exceed the Pauli limit of a BCS superconductor by tens of times. The obtained PVR is among the highest value reported as already explained in the main text of the manuscript. This point is one of the significances from our finding and implies the unconventional nature of these SCs.

In addition, we do not think the significance of finding new superconducting states can be all reflected by the values of critical parameters. For example, the discovery of superconductivity in magic-angle twisted bilayer graphene has clearly triggered tremendous interest in condensed matter physics, but the T_c there is lower than that in cuprates by tens of times. This lower T_c does not diminish in any sense the similarity of phase diagram between MATBG and cuprate superconductors, among other intriguing directions to explore. In our case, the main new findings are the unconventional responses to magnetic fields of these new superconducting states, regardless of the absolute T_c or critical current.

3. The interpretation of the data is predominantly phenomenological and descriptive, lacking the deep physical analysis required for a high-impact publication.
The authors identify a "spin-polarized" superconducting state but provide no physical understanding or theoretical framework for its origin.

Response 24

We believe suggesting SC2 and SC4 to be spin-polarized is a very important physical understanding about these two superconductors. We have further performed the fermiology analysis for the parent state of SC2 and determined it to be spin-polarized and valley-unpolarized half-metal. This is fully consistent with our conclusion that SC2 is spin-polarized. Establishing the isospin structure of the parent and SC states provides very important information about the possible symmetry of the superconducting order parameter. This is the same methodology and rationale used by previous graphene superconductivity literatures, for examples, [H. Zhou et al. *Science* **375**, 774-778 (2022)], [Y. Zhang et al. *Nature* **613**, 268-273 (2023)], [T. Han et al. Nature 643, 654-661 (2025)].

We are not sure what the referee meant by saying 'no physical understanding or theoretical framework for its origin'. If the referee is talking about the pairing mechanism, it is unrealistic for anyone to use transport experiments to nail down the underlying mechanism. This is well known in the field of superconductivity.

The SC3 induced by a perpendicular magnetic field is unconventional and counter-intuitive for a superconductor. This remarkable observation is merely reported without any substantive attempt at a physical explanation, which is a major missed opportunity.
While the authors call for theoretical efforts, such a call is not a substitute for providing their own initial hypotheses or a deeper discussion based on existing theories. The manuscript does not move beyond data presentation to offer new physical insights.

Response 25

We sincerely appreciate the referee's agreement that our observations in SC3 are remarkable. In our manuscript, we have already considered several scenarios for $B_\perp$-induced enhancements in SC3. In brief, we have discussed (1) coupling between $B_\perp$ and spontaneous orbital magnetism, (2) the effect of valley polarization induced by nonzero $B_\perp$, (3) Zeeman coupling between $B_\perp$ and electron spins, and (4) a natural Josephson junction in percolative SC with π phase shift. **This is clearly against the referee's comment 'without any substantive attempt at a physical explanation'.** Please also note that the scenario (2) was added during the revision process.

Unfortunately, none of these scenarios could explain our observations. We have talked to several theorists, including leading ones who work on graphene superconductivity directly, about the possible mechanism. They agreed with our analyses of (1-4) listed above but still could not give a picture that matches the experimental observations perfectly. As experimentalists, we believe that we tried our best in discussing this phenomenon based on existing theories and provided a background for readers to speculate around it. In the revised manuscript, we expanded the discussion of possible origins of SC3 being enhanced by a small $B_\perp$ field based on data taken additionally during the revision as follows: "Firstly, $B_\perp$ can couple to orbital ferromagnetic moments and lower the energy of the SC state versus its competing states. However, unlike chiral SC[45], the absence of magnetic hysteresis in the normal (Fig. 3c) and superconducting (Extended Data Fig. 9g) states of SC3 suggests the vanishing orbital ferromagnetism.

Secondly, even without a spontaneous valley polarization, the valley-contrasted orbital magnetization can be generated by finite $B_\perp$ and induce the valley polarization. The density of states in one of the valleys may increase and thus SC3 is enhanced. This is however contradictory to the disappearance of the enhancement under nonzero $B_\parallel$, since the in-plane field is unlikely to affect the valleys."

Minor points:

4. Optical images of the fabricated devices and the full van der Waals heterostructure are essential for assessing sample quality and geometry. Their absence makes it difficult to evaluate the experimental setup and the reproducibility of the device fabrication.

Response 26

We followed the suggestion and provided the optical images of all the devices, as shown in Figure R17. We have also included the optical images of each device in Extended Data Figures.

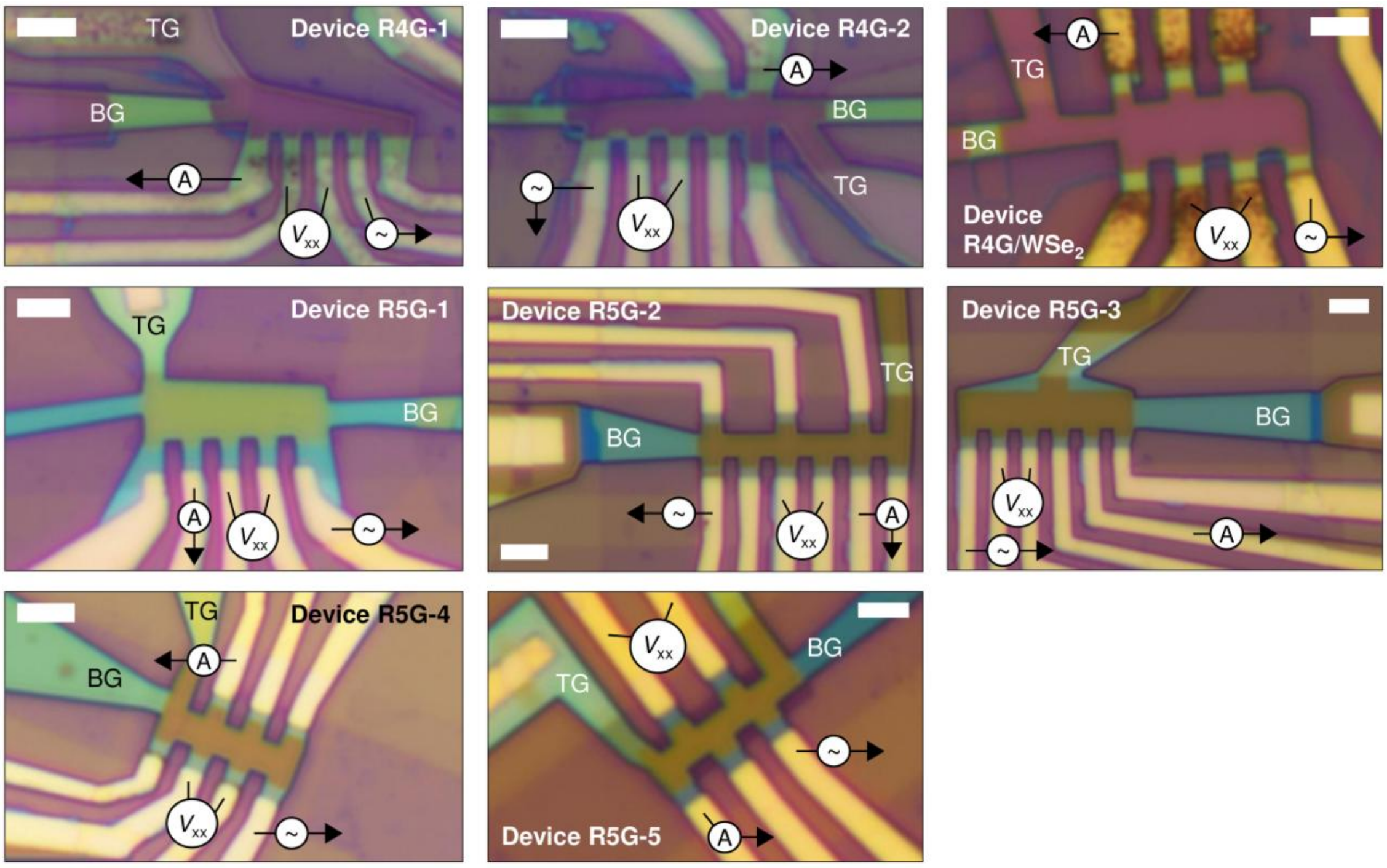

__Figure R17. Optical images of the measured devices.__ In total, two R4G, one R4G/WSe$_2$, and five R5G devices have been studied in this work. BG and TG denote back gate and top gate, respectively. The representative measurement configuration for each device is also described. Scale bars: 3 μm.

5. The claim of superconductivity in each system is seem to be supported by data from only a single device. Given the known variability in van der Waals devices and the strength of the claims, this is inadequate. Multiple devices are necessary to establish the reproducibility and reliability of the observed phenomena.

Response 27

We agree with the referee's comment that multiple devices ought to be tested to firmly establish the phenomena we observed. For bare rhombohedral graphene, we have measured **two R4G devices** and **five R5G devices** in total. In addition to Device R5G-1 and R4G-1 from which data in the main text were obtained, Figure R18-R22 show the data taken from Device R5G-2, R5G-3, R5G-4, R5G-5 and R4G-2.

All the superconducting phases reported in the original manuscript could be observed. Device R5G-2 and R5G-3 were sent to the University of Basel where a high in-plane magnetic field is available. Both of them reproduced SC4 developed at high $B_{\parallel}$ only, as shown in Figure R18 and R19. ***We believe the number of devices studied here and the self-consistency of observations far exceed the standard of the graphene and TMD SC community.***

We guess that the referee might not have appreciated the difference between rhombohedral graphene and moiré-superlattice materials based on 2D materials by saying 'Given the known variability in van der Waals devices.' We would like to take this chance to clarify the critical difference between them. The moiré-superlattice materials, such as twisted graphene and twisted TMD, indeed tends to host pronounced sample-to-sample variations, due to the difficulty in ensuring exactly the same twist angle. Rhombohedral graphene, as a naturally existing crystalline structure, avoids such variability and provides a much more reproducible material system to work on. This is a great advantage when we study and understand the underlying physics. Of course, additional impurities could still be introduced during the stacking process. However, Figure R18-22 clearly demonstrate the reproducibility of our observations across different devices. This suggests, at least in our experiment, the stacking-process-induced variability is a minor issue.

In response to the referee's comment, we added data from Device R5G-2, R5G-3, R5G-4, R5G-5 and R4G-2 in the revised manuscript as Extended Data Figure 3-6.

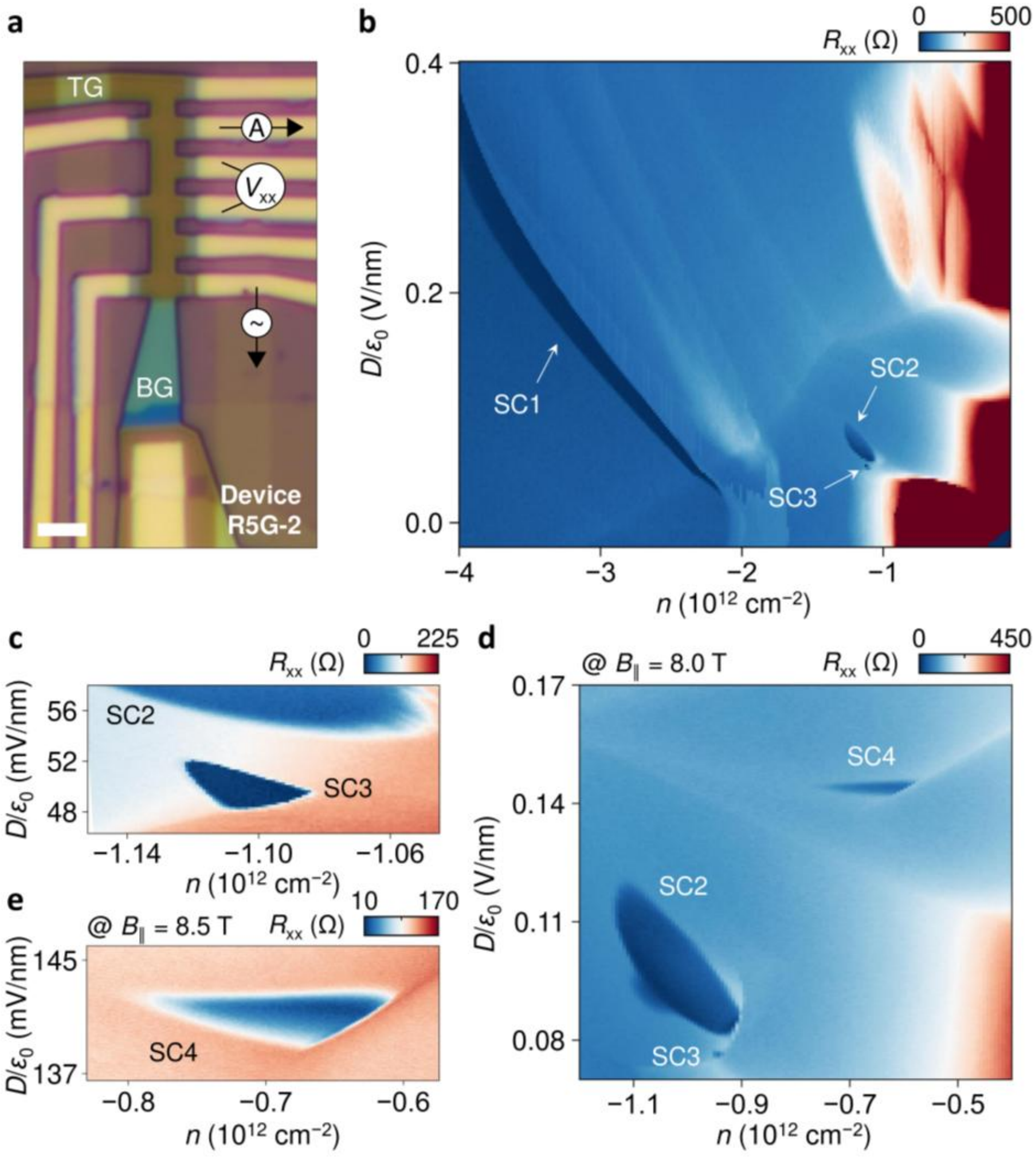

Figure R18. Reproducibility of SC in R5G from Device R5G-2. a**, Optical image of Device R5G-2 with a representative measurement configuration. Scale bar: 3 μm.* ***b, *R_{xx} as a function of n and D/ε_0. All three SCs in R5G at zero magnetic fields are reproduced.* ***c,*** *Zoomed-in view of R_{xx} around SC3.* ***d,*** *R_{xx} as a function of n and*

D/ε_0 at $B_\parallel = 8.0$ T. SC4 that did not appear at zero field is observed. ***e,*** *Zoomed-in view of* R_{xx} *around SC4 at* $B_\parallel = 8.5$ T.

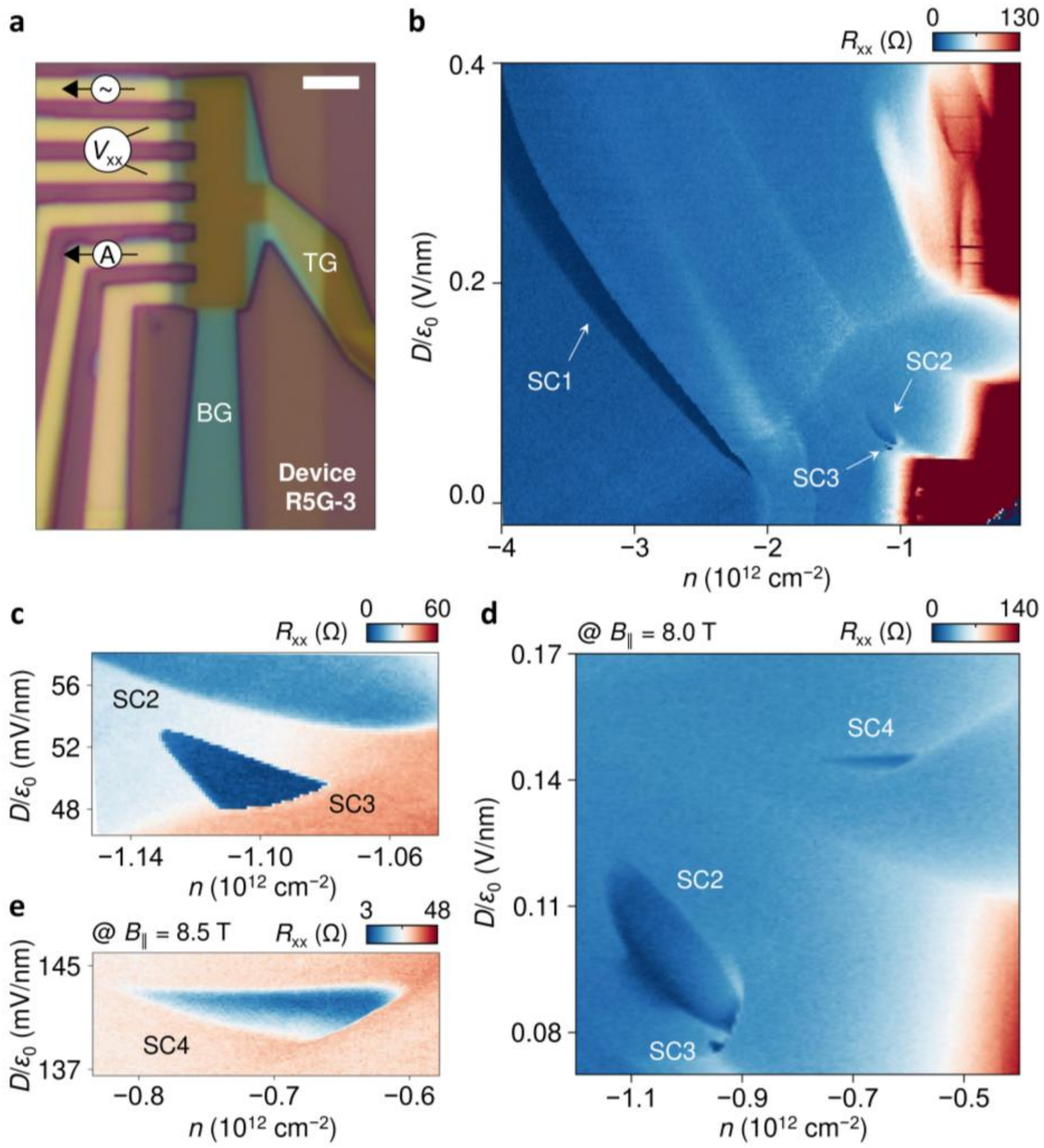

Figure R19. Reproducibility of SC in R5G from Device R5G-3. a*, Optical image of Device R5G-3 with a representative measurement configuration. Scale bar: 3 μm.* ***b,*** R_{xx} *as a function of n and* D/ε_0*. All three SCs in R5G at zero magnetic fields are reproduced.* ***c,*** *Zoomed-in view of* R_{xx} *around SC3.* ***d,*** R_{xx} *as a function of n and* D/ε_0 *at* $B_\parallel = 8.0$ T*. SC4 that did not appear at zero field is observed.* ***e,*** *Zoomed-in view of* R_{xx} *around SC4 at* $B_\parallel = 8.5$ T*.*

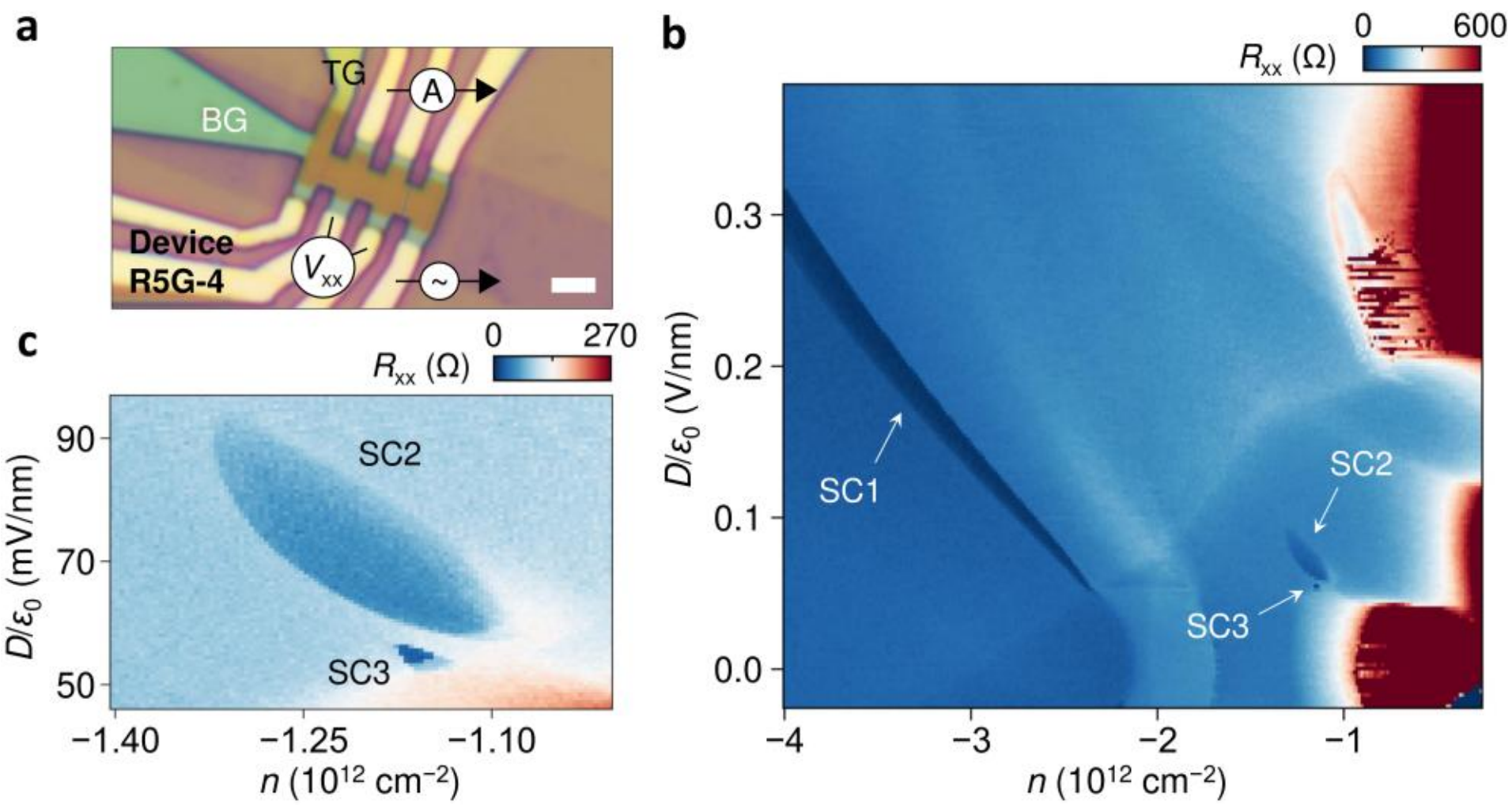

Figure R20. Reproducibility of SC in R5G from Device R5G-4. a*, Optical image of Device R5G-4 with a*

representative measurement configuration. Scale bar: 3 μm. ***b,*** *R_{xx} as a function of n and D/ε_0. All three SCs in R5G at zero magnetic fields are reproduced.* ***c,*** *R_{xx} as a function of n and D/ε_0 focusing on SC2 and SC3.*

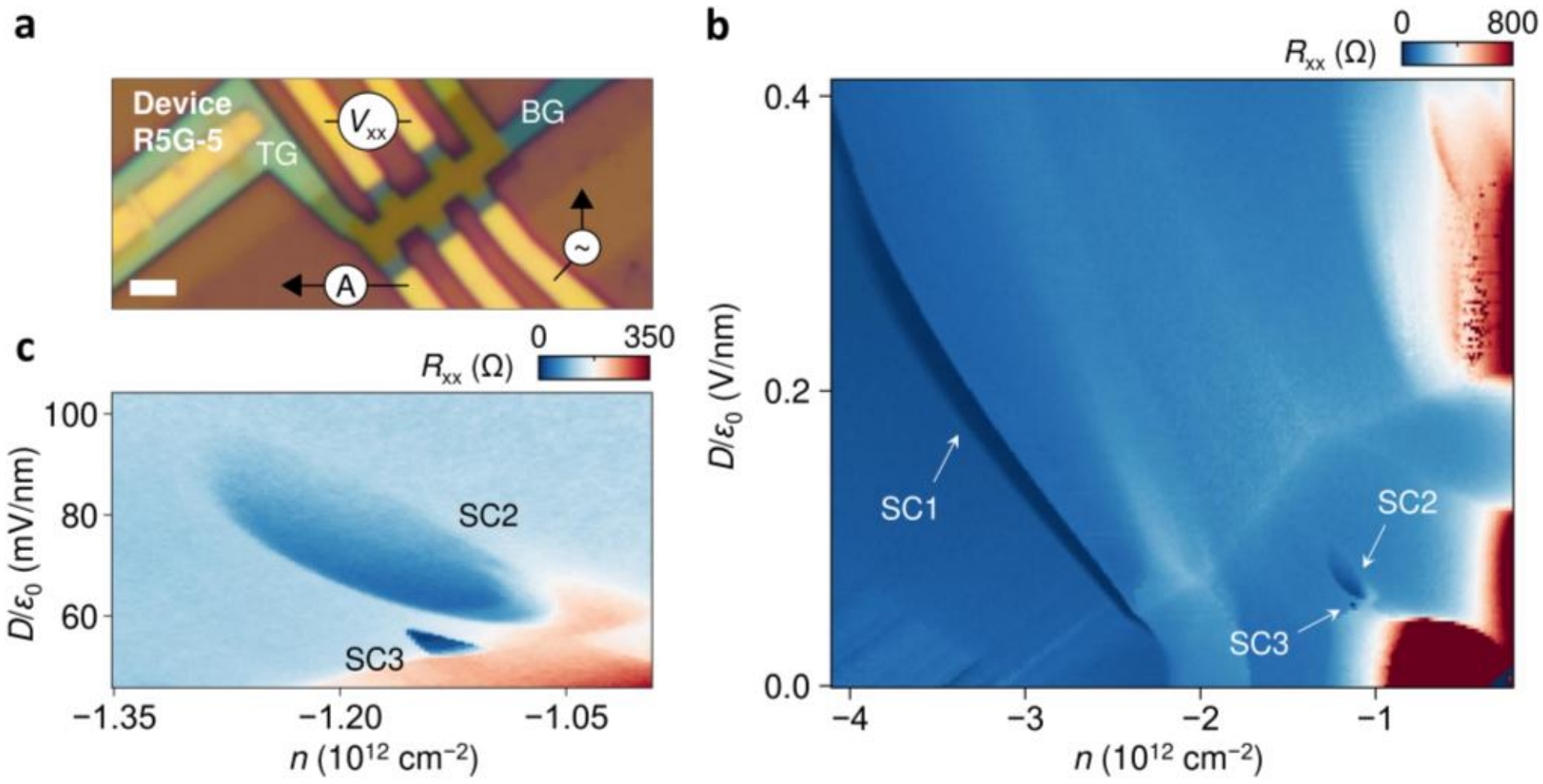

Figure R21. Reproducibility of SC in R5G from Device R5G-5. a*, Optical image of Device R5G-5 with a representative measurement configuration. Scale bar: 2 μm.* ***b,*** *R_{xx} as a function of n and D/ε_0. All three SCs in R5G at zero magnetic fields are reproduced.* ***c,*** *R_{xx} as a function of n and D/ε_0 focusing on SC2 and SC3.*

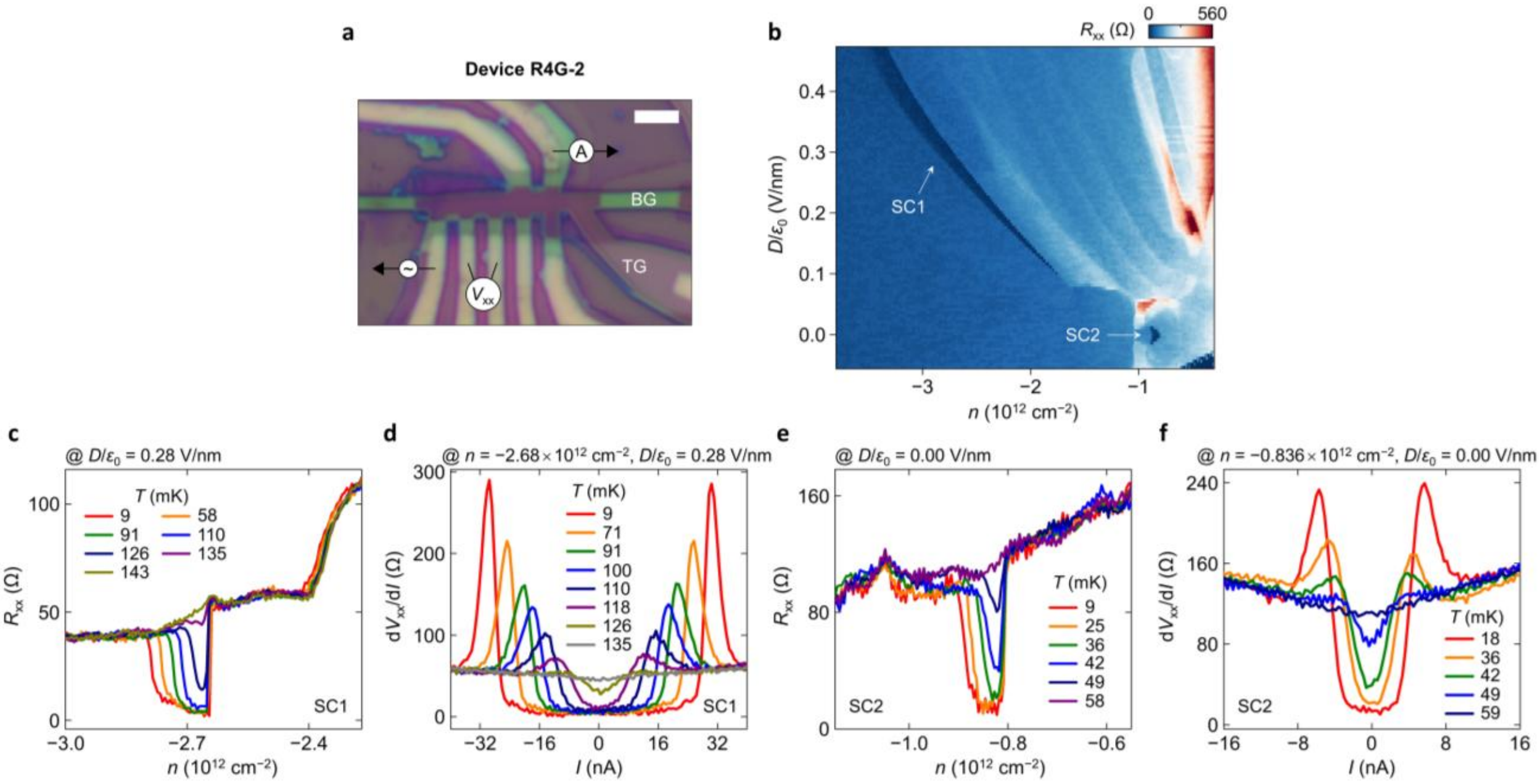

Figure R22. Reproducibility of SC in R4G from Device R4G-2. a*, Optical image of Device R4G-2 with a representative measurement configuration. Scale bar: 3 μm.* ***b,*** *R_{xx} as a function of n and D/ε_0. All the two SCs in R4G are reproduced.* ***c,d,*** *R_{xx} as a function of n at varied temperatures and fixed D/ε_0 for SC1 (**c**) and SC2 (**d**).* ***e,f,*** *Differential resistance dV_{xx}/dI as a function of I at varied temperatures for SC1 (**e**) and SC2 (**f**).*

Referee #3 (Remarks to the Authors):

The manuscript by Seo and co-authors reports a study of superconductivity in tetra- and penta-layer rhombohedral graphene (R4G and R5G). This study focuses on the hole-doped regime, where the authors report the discovery of several new superconducting states. First of all, in both R5G and R4G samples,

they observe a superconducting state SC1 that is similar to the one reported in R3G earlier. In addition, they also report several other superconducting states in R5G with unusual magnetic field dependences. These states violate the Pauli limit, suggesting unconventional pairing, and also show enhancement with in-plane field (SC2, SC4) or out-of-plane field (SC3). The authors also report how adding the spin-orbit coupling affects the superconducting states in a R4G/WSe2 device.

Rhombohedral few-layer graphene has been shown to have an extremely rich phase diagram of strongly correlated electronic phases, including superconductors, integer and fractional quantum anomalous Hall states, and candidates for chiral superconductivity. The reported discovery of several new superconducting states with unusual responses to magnetic field is exciting and will be of high interest to a broad community of condensed matter physicists.

Response 28

We thank the referee for appreciating the merits of our work and summarizing the main points.

However, as I elaborate below, my primary concern is that the presented experimental evidence might not be sufficient to establish the presence of new superconducting states, at least for some of those states. In particular, it appears that some of the superconducting states are only observed in individual devices. Moreover, the authors do not provide an explicit discussion of the number of measured devices and/or the measured contact configuration. The authors also do not provide the information about the devices geometry. However, this information is crucial for establishing the credibility of their exciting finding. The authors need to address these issues and some other comments before I can recommend the manuscript for publication.

Response 29

We understand and fully agree that reproducibility, measurement contact configuration, and device geometry are important information to establish the credibility of our findings. We are glad to provide all this information as detailed below (as well as in the responses to Referee #1&2). In short, all our observations can be repeated **in total eight devices**. This is at least rare among all groups that work and publish on 2D materials. We hope this new information could address the remaining concerns of the referee.

The main findings of this work build upon the discovery of several new superconducting states. Hence, it is crucial that the existence of those superconducting states is established with high credibility. Unfortunately, the resistance measurements often do not provide unambiguous evidence of superconductivity in graphene systems where both ballistic transport and numerous phase transitions between symmetry-broken metals are possible. For example, temperature-driven phase transitions to various symmetry-broken metallic states, which are so ubiquitous in rhombohedral and moiré systems, can look very similar to superconductivity (for example, as was pointed out in He. et al. Nature Physics 17 (2021)). Moreover, there are numerous examples when the initial reports of superconducting states observed in transport in graphene systems were not confirmed in the subsequent studies of those systems. To the best of my knowledge, this is the case with superconducting states in rhombohedral trilayer aligned to hexagonal boron nitride (arXiv:1901.04621), some superconducting states at low superlattice fillings in twisted bilayer graphene (arXiv:1903.06513), twisted double-bilayer (arXiv:1903.08130), and in twisted monolayer-bilayer graphene (arXiv:2004.12414). In the last two examples, the claim of

superconductivity was removed or softened in the published versions. It is worth pointing out that both the temperature dependence of the resistivity and even the I-V curves can look similar to those associated with superconductivity.

Response 30

We appreciate the comment and agree that transport measurements (such as the temperature dependence of R_{xx} and V_{xx}-I relations) could mix the behaviors of superconductors with metals, if not treated properly. The example references cited by the referee indeed raise two important questions: 1. what are and how to rule out trivial explanations of superconductor-like temperature dependence of R_{xx} and V_{xx}-I relations? 2. why some initially reported superconducting states observed in transport in graphene systems were not confirmed in the subsequent studies? In the following, we directly compare our work with the references cited by the referee and explain the differences.

Firstly, [M. He et al. *Nat. Phys.* **17**, 26 (2021)] reported superconductor-like temperature-dependent resistance and nonlinear V_{xx}-I relations at low temperatures in a twisted double bilayer graphene sample. The authors concluded that the observed state is not a superconductor, and explained the nonlinear V_{xx}-I as due to Joule-heating effect. The key evidence is that $\mathrm{d}V_{xx}/\mathrm{d}I$ shows monotonic increase as a function of $P = V_{xx}I$ and equivalently I (because $\mathrm{d}V_{xx}/\mathrm{d}I$ is not negative). This is shown in Fig. 2d and 2e in [M. He et al. *Nat. Phys.* **17**, 26 (2021)], and might also be the case of Fig. 2c in [G. Chen et al. *Nature* **572**, 215-219 (2019)]. In stark contrast, $\mathrm{d}V_{xx}/\mathrm{d}I$ in our data shows clear peaks at the threshold current instead of monotonic increase. Hence, we conclude nonlinear V_{xx}-I relations for the SCs reported in this work do not originate from Joule heating.

Secondly, all the references cited by the referee studied graphene-based moiré superlattice systems, between two graphene flakes or between graphene and hBN. It is well known that the twist angle plays an important role in determining the ground state in such systems (hence 'magic' angle). Experimentally, it is hard to ensure precisely the same twist angle from sample to sample, or even uniformity of twist-angle within the same sample as evidenced by [A. Uri et al. *Nature* **581**, 47 (2020)] and [H. Yoo et al. *Nat. Mater.* **18**, 448 (2019)]. Therefore, it is not surprising that some of previously reported superconductivity could not be reproduced. Rhombohedral graphene studied in our work, however, is free of such problems due to its highly uniform crystalline structure and not relying on moiré superlattices. All the observations we reported in the first draft can now be reproduced in multiple devices, including the initial devices and additional ones measured during the revision process, we have measured a total of eight: **two R4G devices, five R5G devices, and one R4G/WSe_2 device**. Further details about the measured devices are shown in **Response 33** and **34**. We believe the repeatability of SC phenomenology in R4G and R5G is above the standard of published papers in the graphene superconductivity community. Hence, lots of concerns on graphene-based moiré systems are not applied to rhombohedral graphene for good reasons.

Based on the differences in both transport phenomena and repeatability, we conclude that our claims of superconductivity in the current manuscript are much more robust than in previous literatures, even if our work is based on pure transport measurements.

It is even more critical to ensure the state is a superconductor when these candidate states manifest some features that are typically not shown by superconductors, such as enhancement by in-plane or out-of-plane magnetic field reported by the author in this manuscript, or when the candidate states do not show zero resistance, which is the case with SC2 (Fig. 2). The authors observe some signs of possible interference (Ext. Fig. 7) which could provide some additional evidence for superconductivity, but they are relatively weak and only reported for SC3.

Response 31

We appreciate the referee's comment. First of all, SC2 in a new R5G device fabricated during the revision shows nearly zero resistance with a very sharp drop across the transition temperature. Figure R23 shows the temperature dependence of SC2 at a couple of (n, D) points taken at $B_\parallel$ = 8.0 T from Device R5G-2. The resistance R_{xx} drops from ~90 Ω to the value close to zero ohm across the transition temperature.

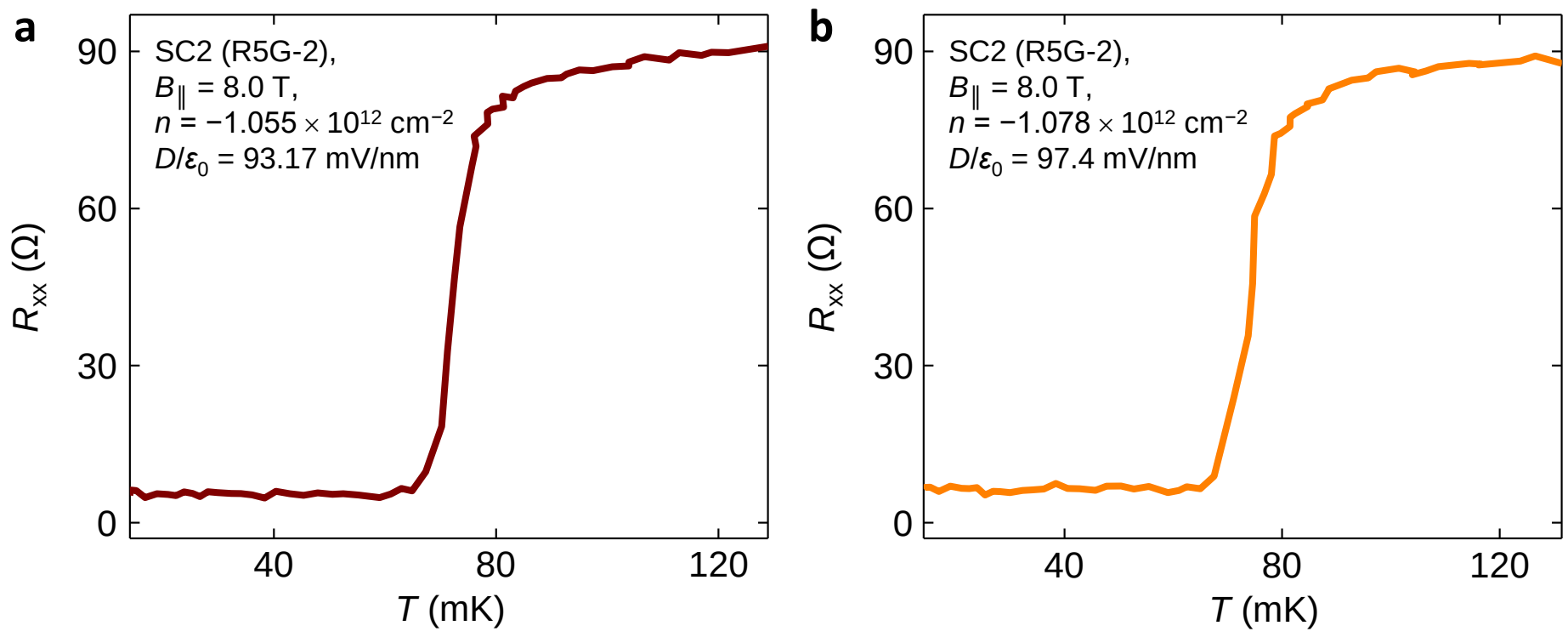

Figure R23. Temperature dependence of SC2 across the SC transition. *In the new Device R5G-2, the SC transitions are very sharp and SC2 approaches 0 Ω much better than the device in the previous manuscript. The data were taken at high* $B_\parallel$ *to strengthen SC2.*

In addition, we followed the referee's suggestion to measure interference patterns as additional evidence of superconductivity. Figure R24 shows such data for: SC1 (Fig. R24a) in R4G; SC1 (Fig. R24b&c), SC2 (Fig. R24d-f) and SC3 (Fig. R24g&h) in R5G; SC1 (Fig. R24i), SC3 (Fig. R24j), SC4 (Fig. R24k) and SC6 (Fig. R24l) in R4G/WSe_2. Clear interference patterns can be observed in most of the reported states. Some of these states did not strictly touch R_{xx} = 0 Ω, but still showed interference patterns. We believe the reason for nonzero residual R_{xx} below the transition temperature could be due to imperfect thermalizations of electrons [L. Holleis et al. *Nat. Phys.* **21**, 444-450 (2025)] that can arise from insufficient noise filtering and non-ideal electrical contacts. These problems are common in micron-size graphene devices at ultralow temperatures. Since electrical transport measurements cannot be cure-all for characterizing SCs in every aspect owing to these experimental factors, we measured multiple devices (in total two R4G and five R5G devices) to minimize their impact. We could reproduce the same phenomenology in all of these devices.

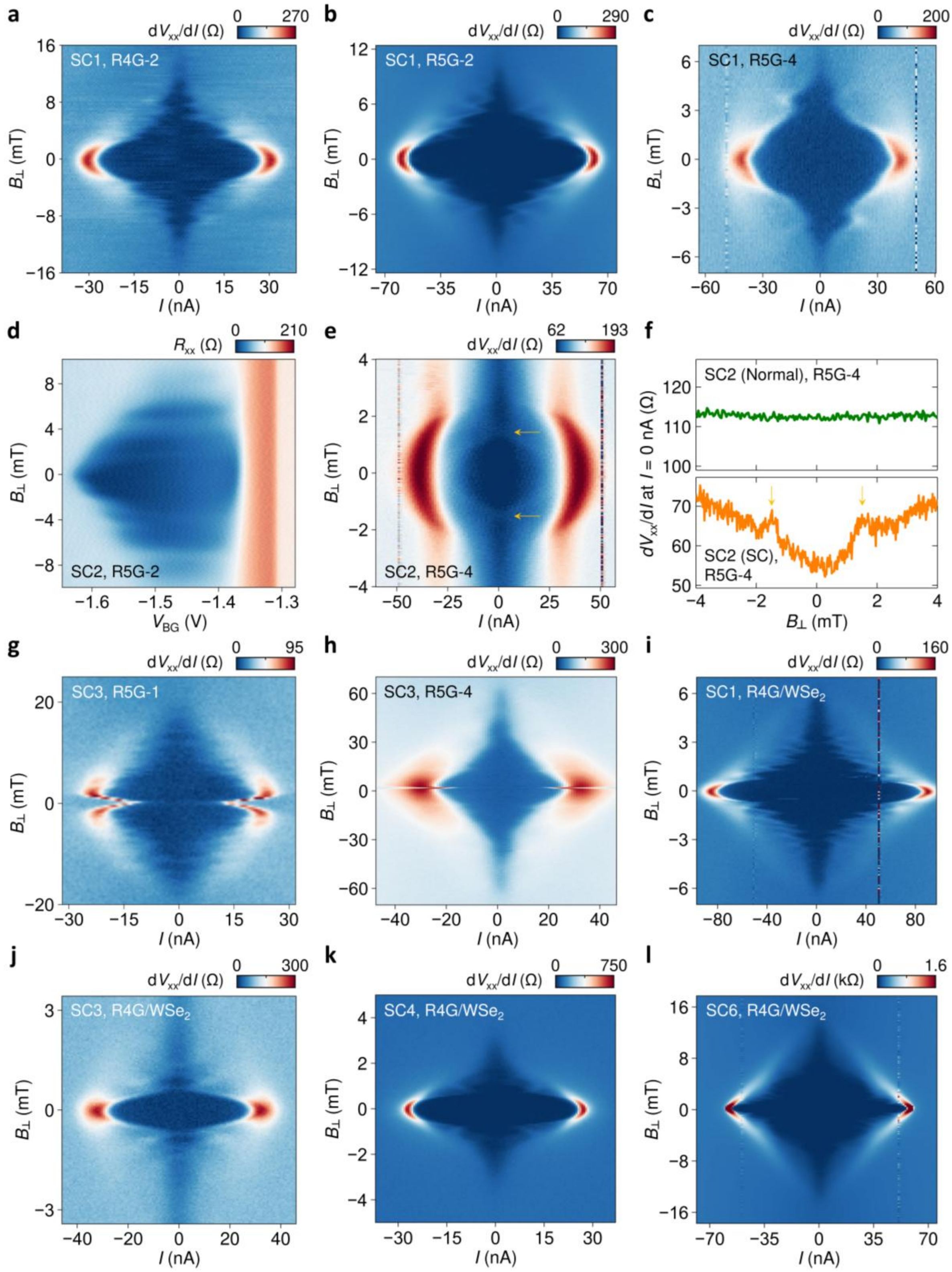

Figure R24. Fraunhofer-like interference patterns. ***a****, Differential resistance as a function of I and $B_\perp$ for SC1 in Device R4G-2 ($n = -2.68 \times 10^{12}$ cm^{-2}, $D/\varepsilon_0 = 0.28$ V/nm).* ***b,c,*** *Differential resistance as a function of I and $B_\perp$ for SC1 in Device R5G-3 ($n = -3.38 \times 10^{12}$ cm^{-2}, $D/\varepsilon_0 = 0.214$ V/nm,* ***b****) and Device R5G-4 ($n = -3.57 \times 10^{12}$ cm^{-2}, $D/\varepsilon_0 = 0.225$ V/nm,* ***c****).* ***d,*** *R_{xx} as a function of V_{BG} and $B_\perp$ for SC2 in Device R5G-2 ($V_{TG} = -0.2$ V). The oscillation of R_{xx} by $B_\perp$ is observed.* ***e,*** *Differential resistance as a function of I and $B_\perp$ for SC2 in Device R5G-3 ($n = -1.17 \times 10^{12}$ cm^{-2}, $D/\varepsilon_0 = 67$ mV/nm). Small increase in the threshold current from $B_\perp$ of ~2 to ~4 mT is observed.* ***f,*** *Differential resistance at zero DC current for SC2 in Device R5G-3 ($n = -1.17 \times$*

*10^{12} cm^{-2}, D/ε_0 = 67 mV/nm) in its normal state (top panel) and superconducting state (bottom panel). The modulation by $B_\perp$, highlighted with yellow arrows corresponding to those in **e**, only exists below the SC transition temperature. This implies the modulation of dV_{xx}/dI is inherent in the superconducting state. **g,h,** Differential resistance as a function of I and $B_\perp$ for SC3 in Device R5G-1 ($n = -1.135 \times 10^{12}$ cm^{-2}, D/ε_0 = 57 mV/nm, **g**) and Device R5G-4 ($n = -1.16 \times 10^{12}$ cm^{-2}, D/ε_0 = 55 mV/nm, **h**). **i-l,** Differential resistance as a function of I and $B_\perp$ for SC1 ($n = -3.25 \times 10^{12}$ cm^{-2}, D/ε_0 = −0.42 V/nm, **i**), SC3 ($n = -2.356 \times 10^{12}$ cm^{-2}, D/ε_0 = 0.18 V/nm, **j**), SC4 ($n = -1.482 \times 10^{12}$ cm^{-2}, D/ε_0 = 72 mV/nm, **k**) and SC6 ($n = -1.00 \times 10^{12}$ cm^{-2}, D/ε_0 = 26 mV/nm, **l**) in Device R4G/WSe_2. All these data further corroborate the formation of superconducting states.*

In general, interference patterns are much weaker in crystalline graphene than in twisted (especially bilayer) graphene superconductors. This was also the case in some of our superconducting states, as shown in Figure R25. We believe possible reasons for weaker interference patterns are (1) less inhomogeneity in a crystalline graphene system and (2) a lower out-of-plane critical field that kills SC even before the interference pattern is developed. Firstly, there can be non-SC islands in a micron-size device due to sample inhomogeneity, even when (*n*, *D*) are tuned to a superconducting phase. If the sample is cleaner, there is less chance that the non-SC islands are formed and thus the interference pattern becomes less pronounced. This is also the case for twisted graphene superconductors. Compared to magic-angle twisted bilayer graphene (Figure R26a), twisted multilayer graphene takes advantage of angle-locking effect [S. Turkel et al. *Science* **376**, 193-199 (2022)] and shows less clear interference patterns (see Figure R26b-d for examples) [Z. Zhou et al. *Nat. Phys.* **21**, 1773-1779 (2025); Y. Zhang et al. *Science* **377**, 1538-1543 (2022)]. Secondly, the out-of-plane critical field of SCs here is so low that they can be killed before showing the interference patterns. Figure R25 shows comparison between the SC states in the same sample with a clear (R25a, R25c & R25e) and weak/no (R25b, R25d & R25f-h) interference pattern. When the first side-peak of the interference pattern appears (indicated as the dashed line), the resistance of the SC with a weak pattern already recovers the normal-state R_{xx} mostly. This was also the case for twisted bilayer graphene with a low out-of-plane critical field (Figure R26e). To visualize a clearer interference pattern for these SCs, devices with a much more delicate design such as gate-defined Josephson junctions are necessary. This is a part of ongoing efforts and is beyond the scope of the current work.

In response to the referee's comment, we added a section to discuss the observation of interference patterns in the Methods section as "Fraunhofer-like interference patterns and nonzero residual resistance."

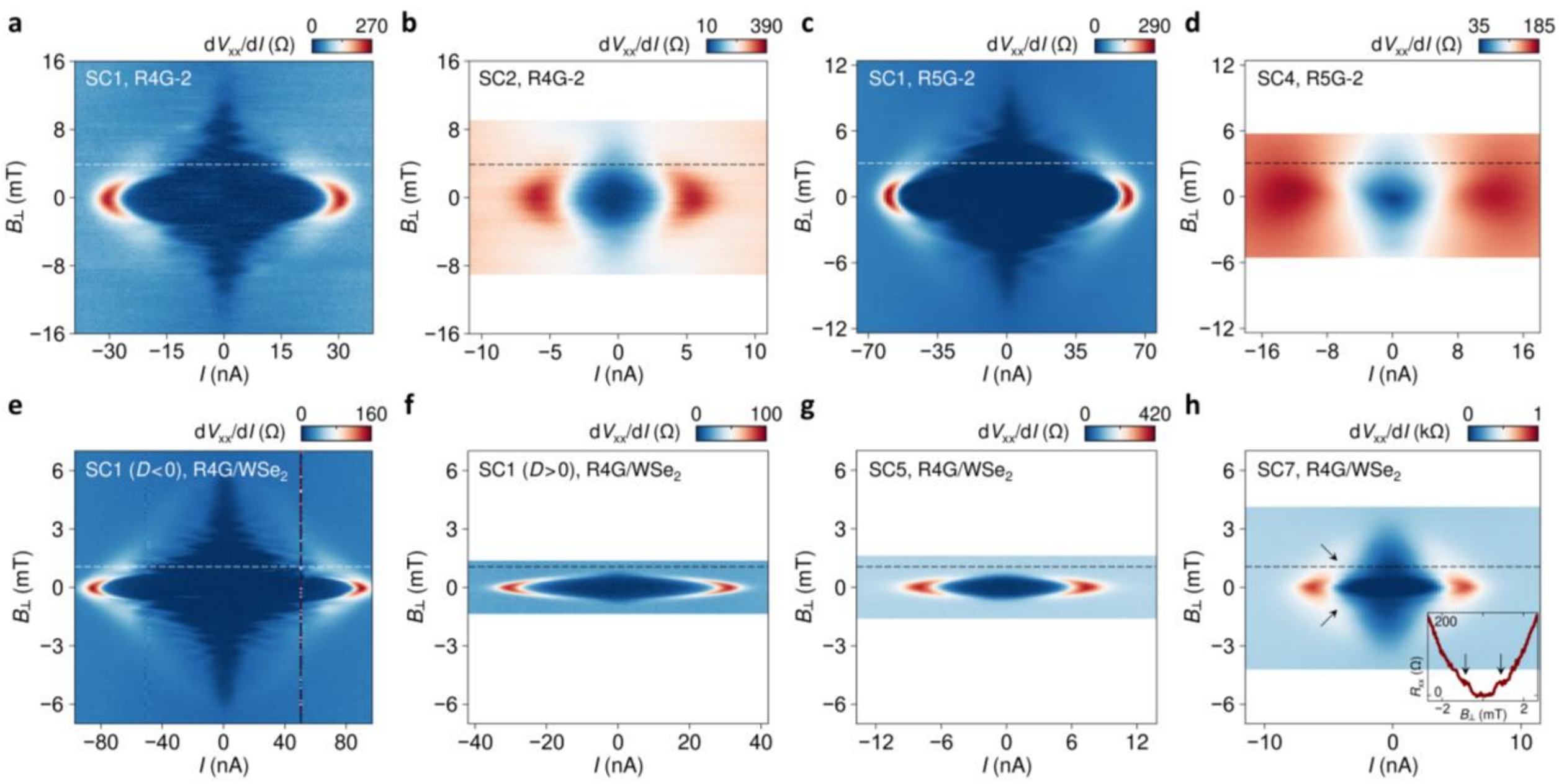

Figure R25. Comparison to cases with weak/no interference patterns. a,b, *Differential resistance as a function I and $B_\perp$ for SC1 ($n = -2.68 \times 10^{12}$ cm^{-2}, $D/\varepsilon_0 = 0.28$ V/nm,* ***a****) and SC2 ($n = -0.842 \times 10^{12}$ cm^{-2}, $D/\varepsilon_0 = 6.2$ mV/nm,* ***b****) in Device R4G-2. The dashed lines denote $B_\perp$ at which the first side-peak of interference patterns in* ***a*** *appears. SC2 mostly recovers the normal-state resistance at this line, indicating SC2 is killed before the interference pattern might be developed.* ***c,d,*** *Differential resistance as a function of for I and $B_\perp$ for SC1 ($n = -3.38 \times 10^{12}$ cm^{-2}, $D/\varepsilon_0 = 0.214$ V/nm,* ***c****) and SC4 ($n = -0.606 \times 10^{12}$ cm^{-2}, $D/\varepsilon_0 = 0.144$ V/nm,* ***d****) in Device R5G-3. The dashed lines denote $B_\perp$ at which the first side-peak of interference patterns in* ***c*** *appears. SC4 mostly recovers the normal-state resistance at this line, indicating SC2 is killed before the interference pattern might be developed.* ***e-j,*** *Differential resistance as a function I and $B_\perp$ for SC1 at negative D ($n = -3.25 \times 10^{12}$ cm^{-2}, $D/\varepsilon_0 = -0.42$ V/nm,* ***e****), SC1 at positive D ($n = -3.385 \times 10^{12}$ cm^{-2}, $D/\varepsilon_0 = 0.469$ V/nm,* ***f****), SC5 ($n = -1.242 \times 10^{12}$ cm^{-2}, $D/\varepsilon_0 = 72$ mV/nm,* ***g****) and SC7 ($n = -0.38 \times 10^{12}$ cm^{-2}, $D/\varepsilon_0 = -0.24$ V/nm,* ***h****). The dashed lines denote $B_\perp$ at which the first side-peak of interference patterns in* ***e*** *appears. SC1 at positive D and SC5 recover the normal-state resistance at this line, indicating these SCs are killed before the interference pattern might be developed.* ***Inset****: SC7 develops weak kinks near the current-induced transition and plateaus at dV_{xx}/dI at $I = 0$ nA (i.e., R_{xx}) around the $B_\perp$ value of the dashed line, showing the signatures of weak interference patterns.*

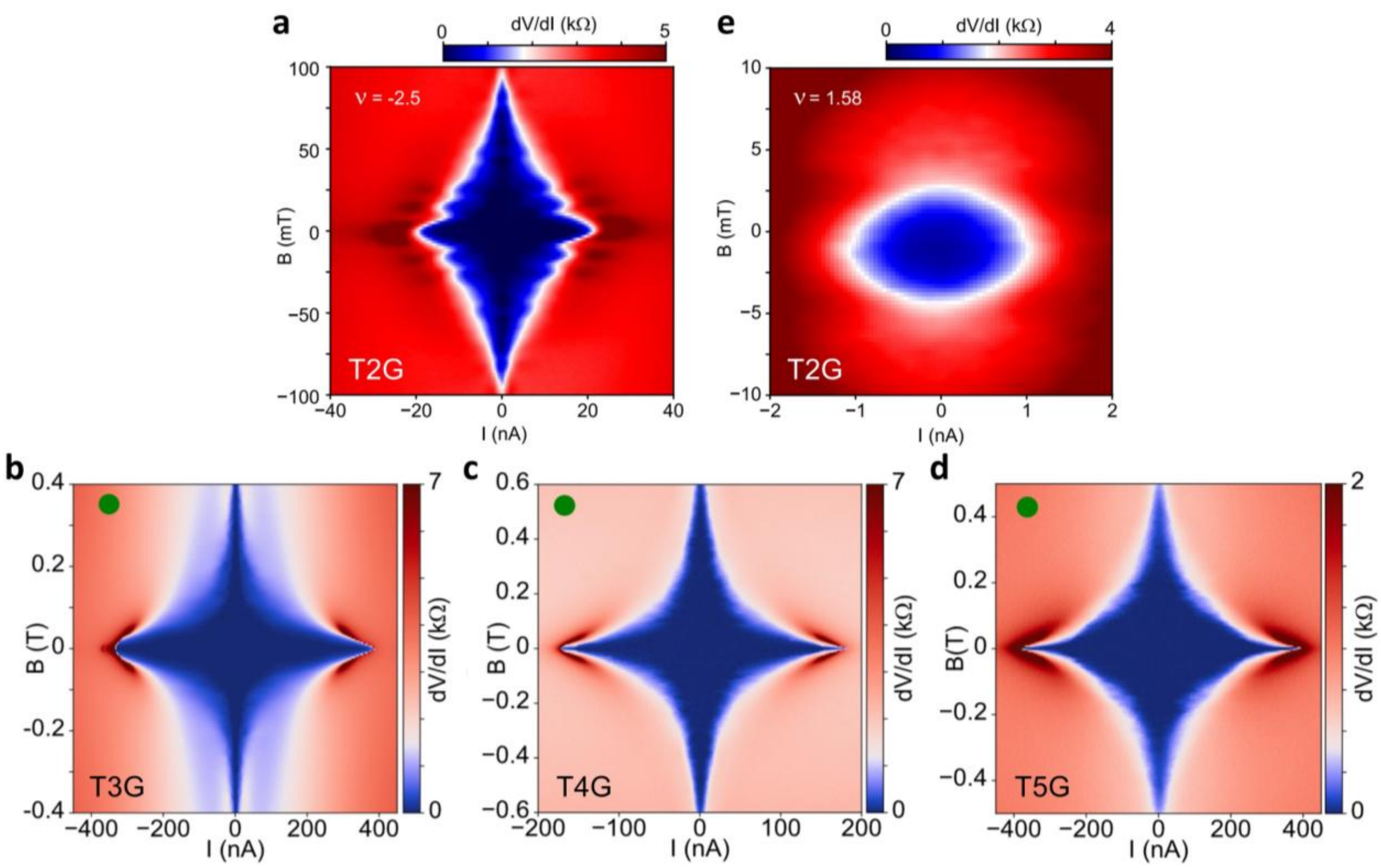

Figure R26. Examples of interference patterns in twisted N-layer graphene (TNG). a-d, *Differential resistance as a function of the DC current I and out-of-plane magnetic field B for twisted bilayer (****a****), trilayer (****b****), tetralayer (****c****) and pentalayer (****d****) graphene with WSe_2 at proximity. When the out-of-plane critical field is high enough, T2G shows a pronounced interference pattern, whereas the thicker twisted graphene does not.* ***e,*** *Differential resistance as a function of I and B for the same T2G sample with a lower out-of-plane critical field. The interference pattern is not observed. The data in* ***a&e*** *are adapted from [H. S. Arora et al. Nature* ***583****, 379-384 (2020)], and the data in* ***b-d*** *are adapted from [Y. Zhang et al. Science* ***377****, 1538-1543 (2022)].*

While observing some other key experimental signatures of superconductivity, such as the Meissner effect, is nearly impossible in graphene, one of the most effective ways to confirm new superconducting states

has been to observe the same phenomenology in multiple devices, as demonstrated in the examples I mentioned above. That's why observing a superconducting state in resistance measurements of only a single device is generally not sufficient for claiming superconductivity with enough confidence. Unfortunately, in their manuscript, the authors do not explicitly discuss the number of measured devices (except mentioning R5G Device 2 in Extended Data Fig.6). The authors also do not discuss measuring different contact configurations, nor do they provide information about device geometry. This crucial information is required to evaluate the credibility of the claim.

Response 32

We totally agree with the referee's comment that reproducing the same phenomenology in multiple devices is very crucial. We have measured **in total five R5G devices and two R4G devices** in this work and reproduced the same SC phenomenology in those samples (Figure R18-R22). The number of devices measured, information about device geometry, and measurements with different contact configurations are elaborated in **Response 33, 34 and 35**, respectively.

In how many devices do the authors observe each of the reported superconducting states?

Response 33

We have measured two R4G devices, five R5G devices, and one R4G/WSe_2 device, whose images are shown in **Figure R27**. Both SC1 and SC2 in R4G were observed in the two R4G devices, and SC1-SC3 in R5G were observed in all five R5G devices. As for SC4 in R5G, we must ship devices from MIT (where they are fabricated) to Basel (where we perform measurements under a high in-plane field as needed to reveal SC4). Unfortunately, some devices were killed during the shipping possibly by static discharge. Therefore, we measured Device R5G-1, R5G-2 and R5G-3 only under high $B_\parallel$ and could reproduce SC4.

In response to the referee's comment, we added data from Device R5G-2, R5G-3, R5G-4, R5G-5 and R4G-2 in Extended Data Figure 3-6 in the revised manuscript, while the main text discusses results from Device R5G-1, R4G-1 and R4G/WSe_2. This is noted in the main text of the revised manuscript as "The main text discusses Device R4G-1, R5G-1 and R4G/WSe_2, while the main findings were reproduced in one other R4G and four other R5G devices in two independent fridges from two different labs (Extended Data Figures)."

Information about the device geometry is crucial in the context of this study and also for the attempts to reproduce the reported findings. In the current version of the manuscript, the authors do not provide such information. Please include the images of the devices in the manuscript.

Response 34

Thanks for pointing out this concern. Figure R27 shows the optical images of the measured devices. In response to the referee's comment, we included the optical images of each device in Extended Data Figures.

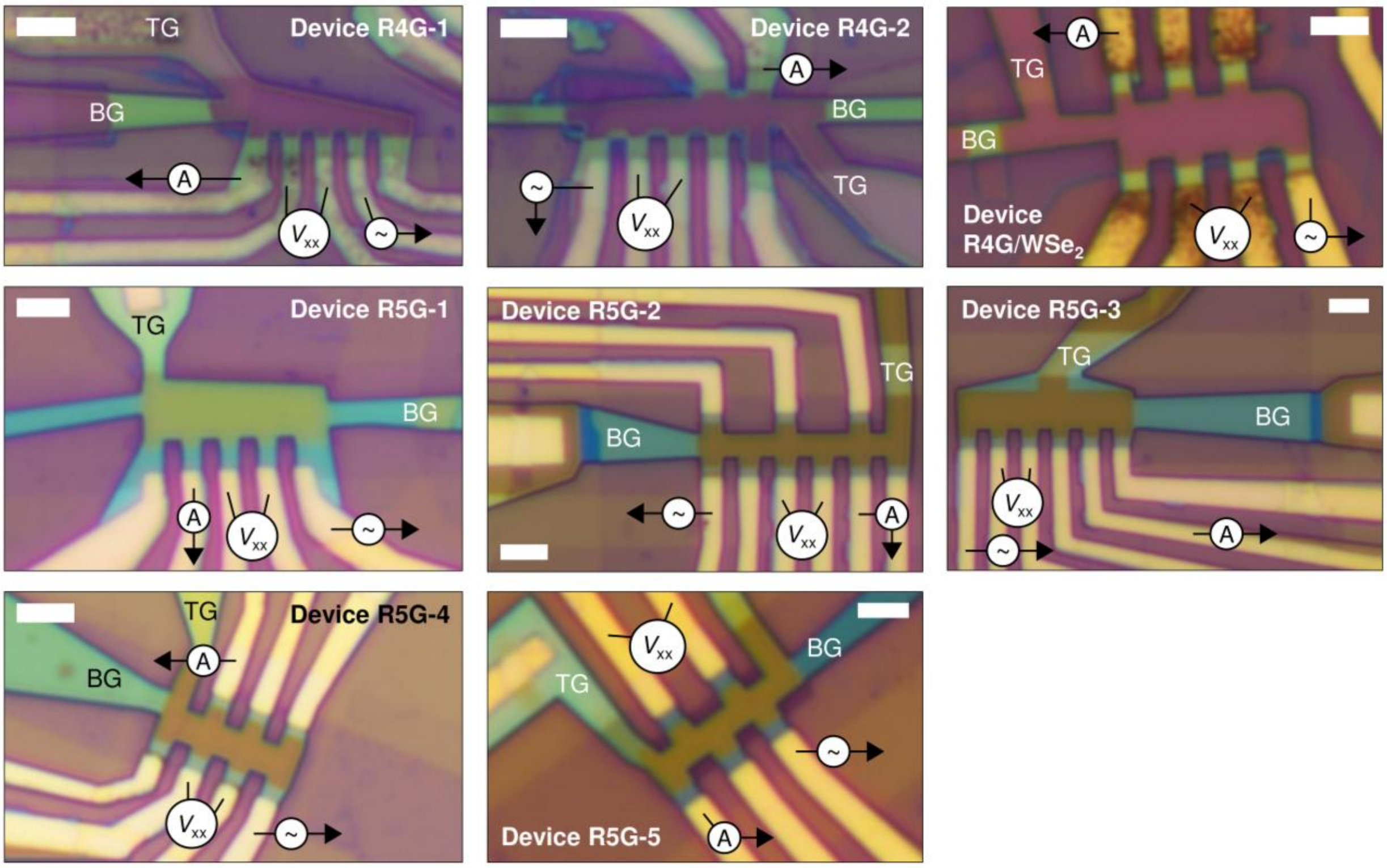

Figure R27. Optical images of the measured devices. *In total, two R4G, one R4G/WSe$_2$, and five R5G devices have been studied in this work. BG and TG denote back gate and top gate, respectively. The representative measurement configuration for each device is also described. Scale bars: 3 μm.*

If devices had multiple electrical contacts, were the reported superconducting states observed for different contact combinations?

Response 35

We have tested different combinations of contacts for several samples. Figure R28, R29 and R30 show the results for the Device R4G-2, R5G-2 and R4G/WSe$_2$, respectively. Clear sudden drops in R_{xx} versus charge density and peaks in dV_{xx}/dI are observed in the tested contacts. We could not test all measurement configurations in all the samples due to the ground loop that could be created when using multiple lock-in amplifiers at the same time.

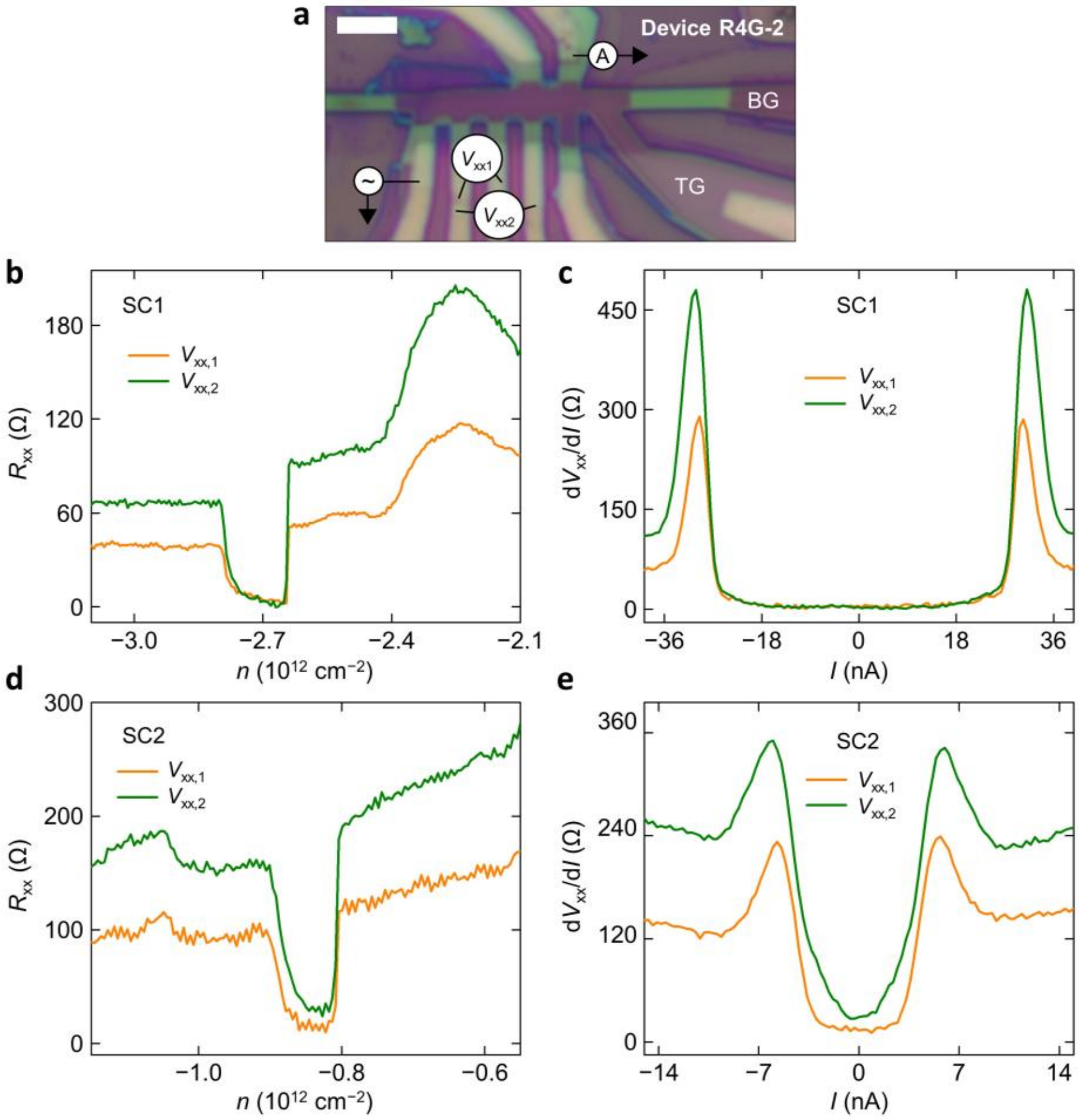

***Figure R28. SCs in Device R4G-2 from different measurement configurations. a**, Two configurations used in the measurements. Scale bar: 3 μm. **b,d,** R_{xx} measured from the two voltage pairs $V_{xx,1}$ and $V_{xx,2}$ for SC1 (D/ε_0 = 0.28 V/nm) (**b**) and SC2 (D/ε_0 = 0.00 V/nm) (**d**). **c,e,** Differential resistance dV_{xx}/dI from the two voltage pairs $V_{xx,1}$ and $V_{xx,2}$ for SC1 ($n = -2.68 \times 10^{12}$ cm^{-2}, D/ε_0 = 0.28 V/nm) (**b**) and SC2 ($n = -0.836 \times 10^{12}$ cm^{-2}, D/ε_0 = 0.00 V/nm) (**e**). Both the decrease in R_{xx} and peaks in dV_{xx}/dI are clear from the two different voltage pairs.*

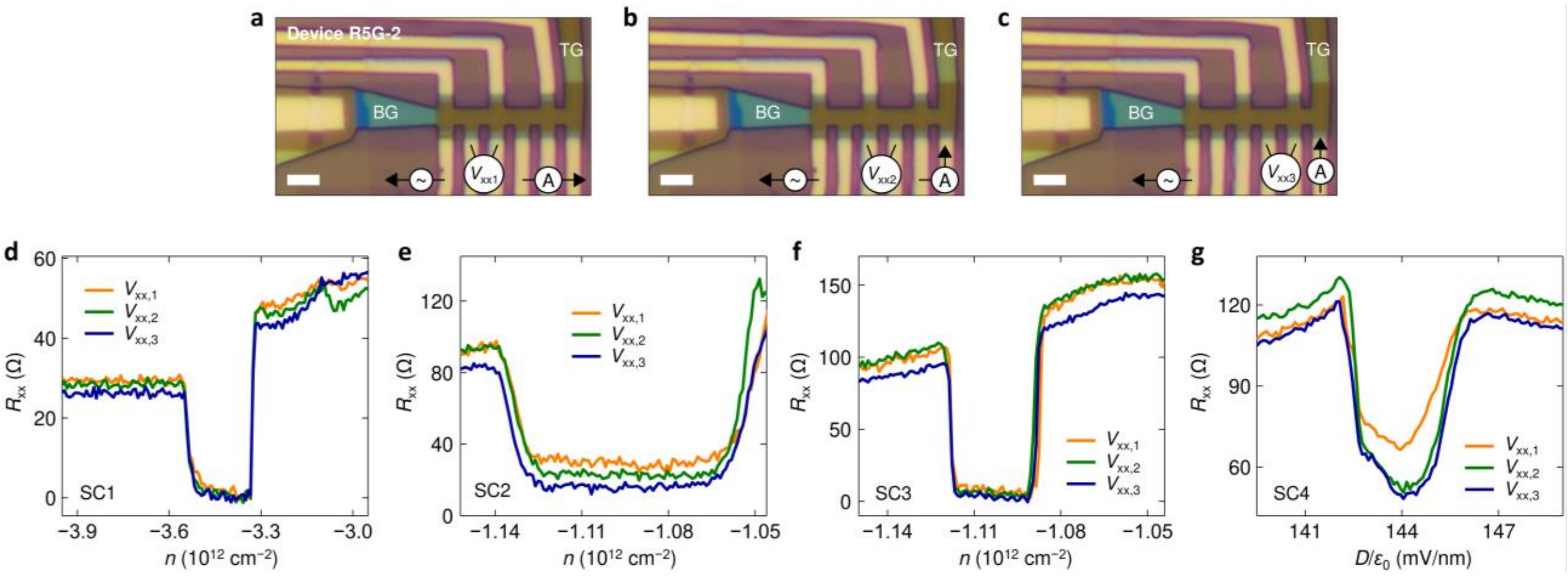

***Figure R29. SCs in Device R5G-2 from different measurement configurations. a-c**, Three configurations used in the measurements. Scale bar: 3 μm. **d-g,** R_{xx} measured from the three voltage pairs $V_{xx,1}$ to $V_{xx,3}$ for SC1 (D/ε_0 = 0.214 V/nm, $B_\parallel$ = 0.0 T) (**d**), SC2 (D/ε_0 = 57 mV/nm, $B_\parallel$ = 0.0 T) (**e**), SC3 (D/ε_0 = 50 mV/nm, $B_\parallel$ = 0.0 T) (**f**) and SC4 ($n = -0.616 \times 10^{12}$ cm^{-2}, $B_\parallel$ = 8.0 T) (**g**). The decrease in R_{xx} is observed from the three different voltage pairs.*

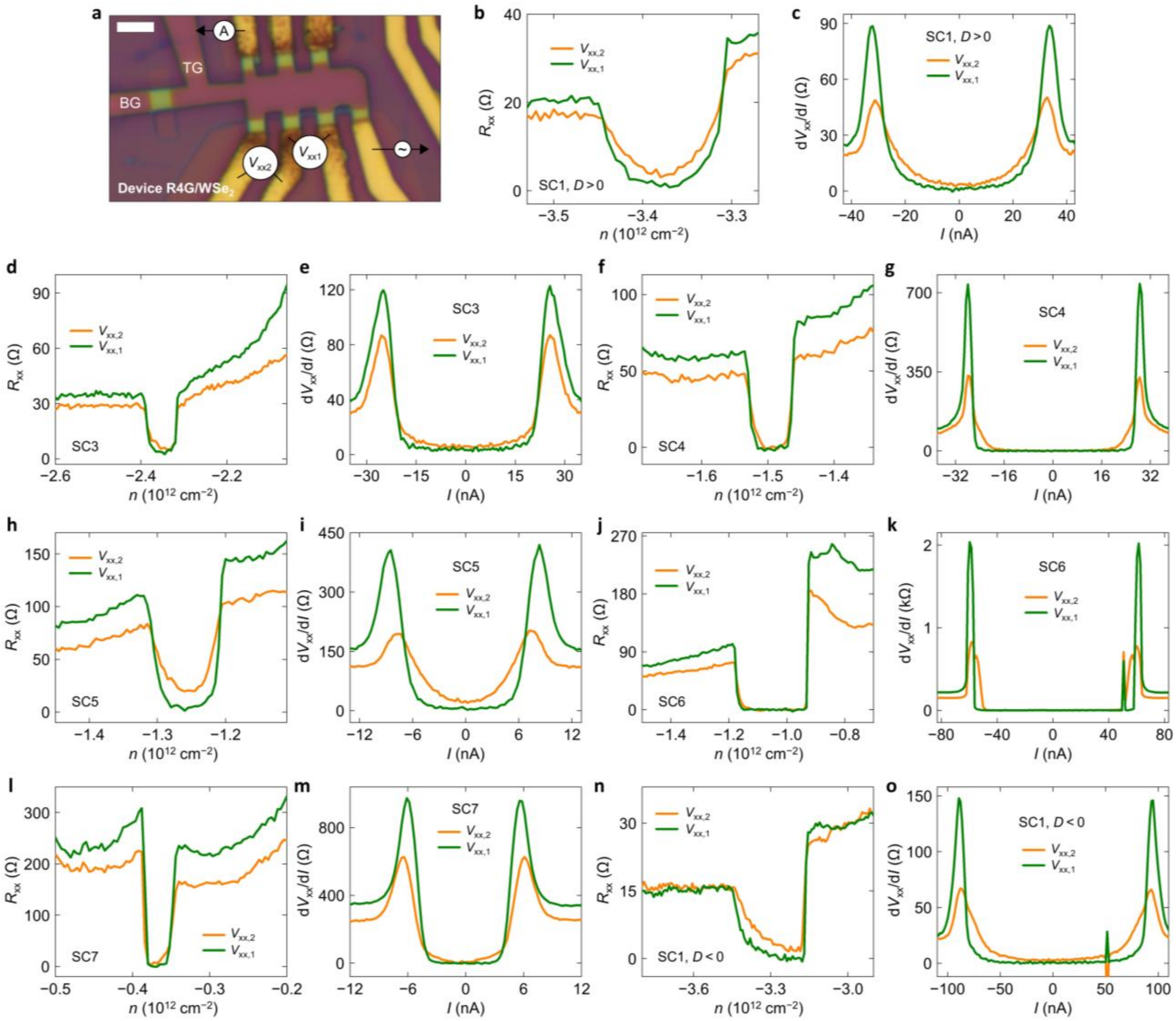

Figure R30. SCs in Device R4G/WSe$_2$ from different measurement configurations. ***a***, *Two configurations used in the measurements. Scale bar: 3 μm.* ***b,d,f,h,j,l,n,*** *R_{xx} measured from the two voltage pairs $V_{xx,1}$ and $V_{xx,3}$ for SC1 at positive D (D/ε_0 = 0.469 V/nm) (**b**), SC3 (D/ε_0 = 0.18 V/nm) (**d**), SC4 (D/ε_0 = 0.072 V/nm) (**f**), SC5 (D/ε_0 = 0.072 V/nm) (**h**), SC6 (D/ε_0 = 0.026 V/nm) (**j**), SC7 (D/ε_0 = −0.24 V/nm) (**l**), and SC1 at negative D (D/ε_0 = −0.42 V/nm) (**n**).* ***c,e,g,i,k,m,o,*** *Differential resistance dV_{xx}/dI measured from the two voltage pairs $V_{xx,1}$ and $V_{xx,3}$ for SC1 at positive D ($n = -3.385 \times 10^{12}$ cm^{-2}, D/ε_0 = 0.469 V/nm) (**c**), SC3 ($n = -2.356 \times 10^{12}$ cm^{-2}, D/ε_0 = 0.18 V/nm) (**e**), SC4 ($n = -1.482 \times 10^{12}$ cm^{-2}, D/ε_0 = 0.072 V/nm) (**g**), SC5 ($n = -1.242 \times 10^{12}$ cm^{-2}, D/ε_0 = 0.072 V/nm) (**i**), SC6 ($n = -1.00 \times 10^{12}$ cm^{-2}, D/ε_0 = 0.026 V/nm) (**k**), SC7 ($n = -0.38 \times 10^{12}$ cm^{-2}, D/ε_0 = −0.24 V/nm) (**m**), and SC1 at negative D ($n = -3.25 \times 10^{12}$ cm^{-2}, D/ε_0 = −0.42 V/nm) (**o**). The decrease in R_{xx} and peaks in dV_{xx}/dI are observed from the two different voltage pairs.*

Measuring weak superconducting states in graphene devices is technically challenging, with electronic noise and high effective electron temperature sometimes playing a major role in obscuring the superconducting states. In the current version of the manuscript, the authors do not provide details on the measures taken to reduce noise and thermalize electrons. This information is crucial for attempts to reproduce these results. Please include the details of the electrical filtering and thermalization stages used on the measurement lines.

Response 36

Thanks for a valuable question and appreciating the technical challenges of measuring a fragile superconducting state. Following the suggestion by this referee, we provide details on the measures taken to reduce noise and thermalize electrons.

In the case of the Bluefors LD250 dilution fridge at MIT, additional electronic filters are installed on the mixing chamber plate. Twisted phosphorus bronze wires or thermocoax cables are first thermalized at each stage of the fridge. Subsequently, the wires go through a home-made thermal meander, ceramic radiofrequency filters (DigiKey), and four-stage RC filters. The wires are additionally thermalized by silver epoxy filters (MFT25 from Basel Precision Instruments, and a home-made one) and a final thermal meander made on a copper core PCB board.

In the case of the Leiden MNK126-700 dilution fridge at the University of Basel, thermocoax cables are thermalized at each stage of the fridge. Next, one home-made silver epoxy filter [C. P. Scheller et al. *Appl. Phys. Lett.* **104**, 211106 (2014)] is installed on the mixing chamber plate, while the same silver epoxy filter is right at the back of the sample holder.

In the revised manuscript, we added the details of the electrical filters and thermalization stages in the Methods Section.

The authors mention two distinct dilution refrigerator systems used in the experiments. Were the reported superconducting states observed in both of them?

Response 37

Yes. Figure R31, R32 and R33 show R_{xx} maps in *n-D* space showing the SC1-SC3 of Device R5G-1, R5G-2 and R5G-3 at zero magnetic fields. They clearly demonstrate the existence of SC1-SC3 in both of the fridges. We note that the most notable difference between the two fridges is whether a high in-plane magnetic field can be applied. For the setup at MIT, there was neither a vector magnet nor sample rotator. Thus, we could not apply a pure in-plane field by *in situ* compensating the out-of-plane field due to sample misalignment from the magnet axis.

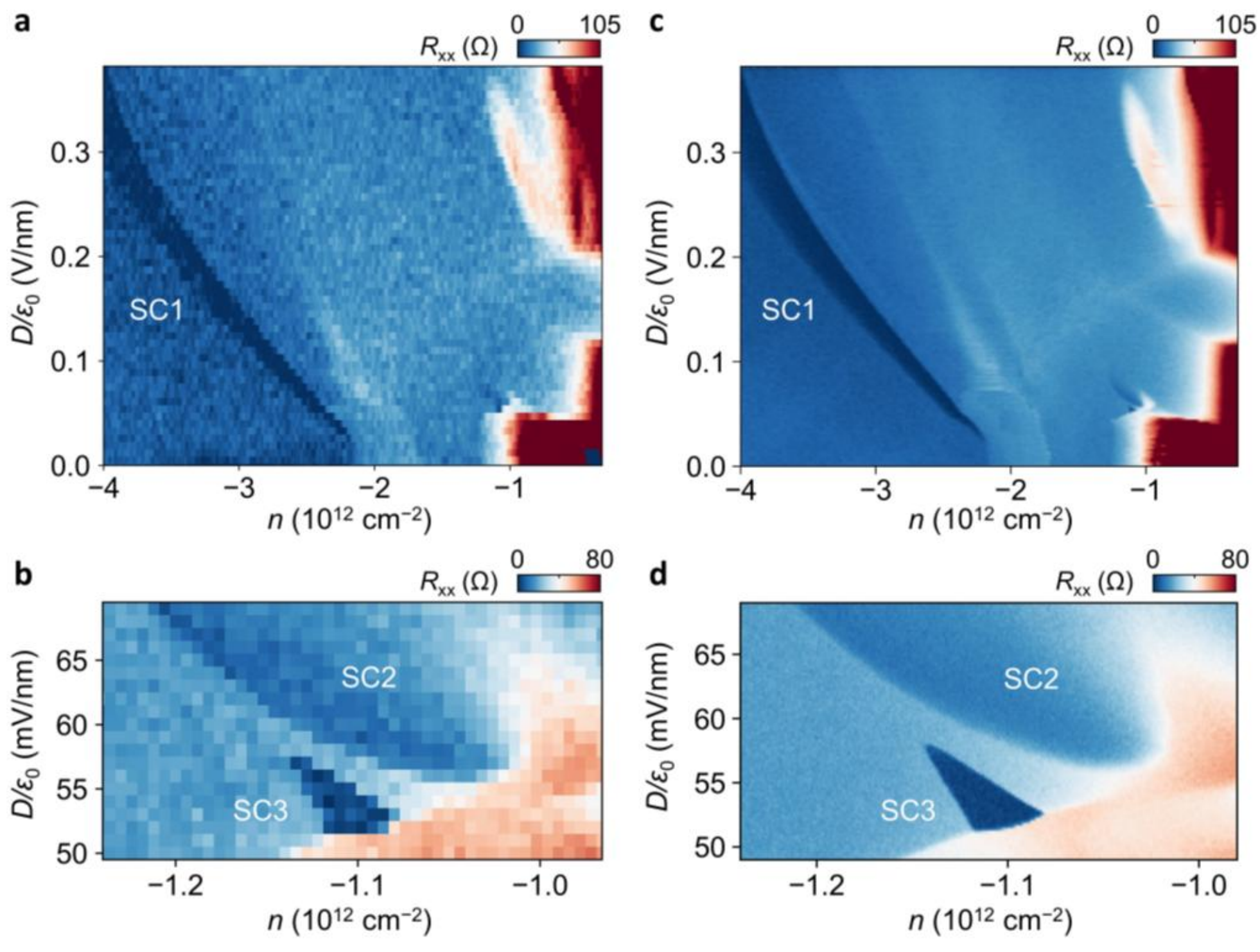

Figure R31. Measurements of Device R5G-1 in the setup at MIT (a,b) and the University of Basel (c,d). *The*

*superconducting states SC1-SC3 are clear in both of the maps. Data points in (**a,b**) are more coarse due to the lack of measurement time.*

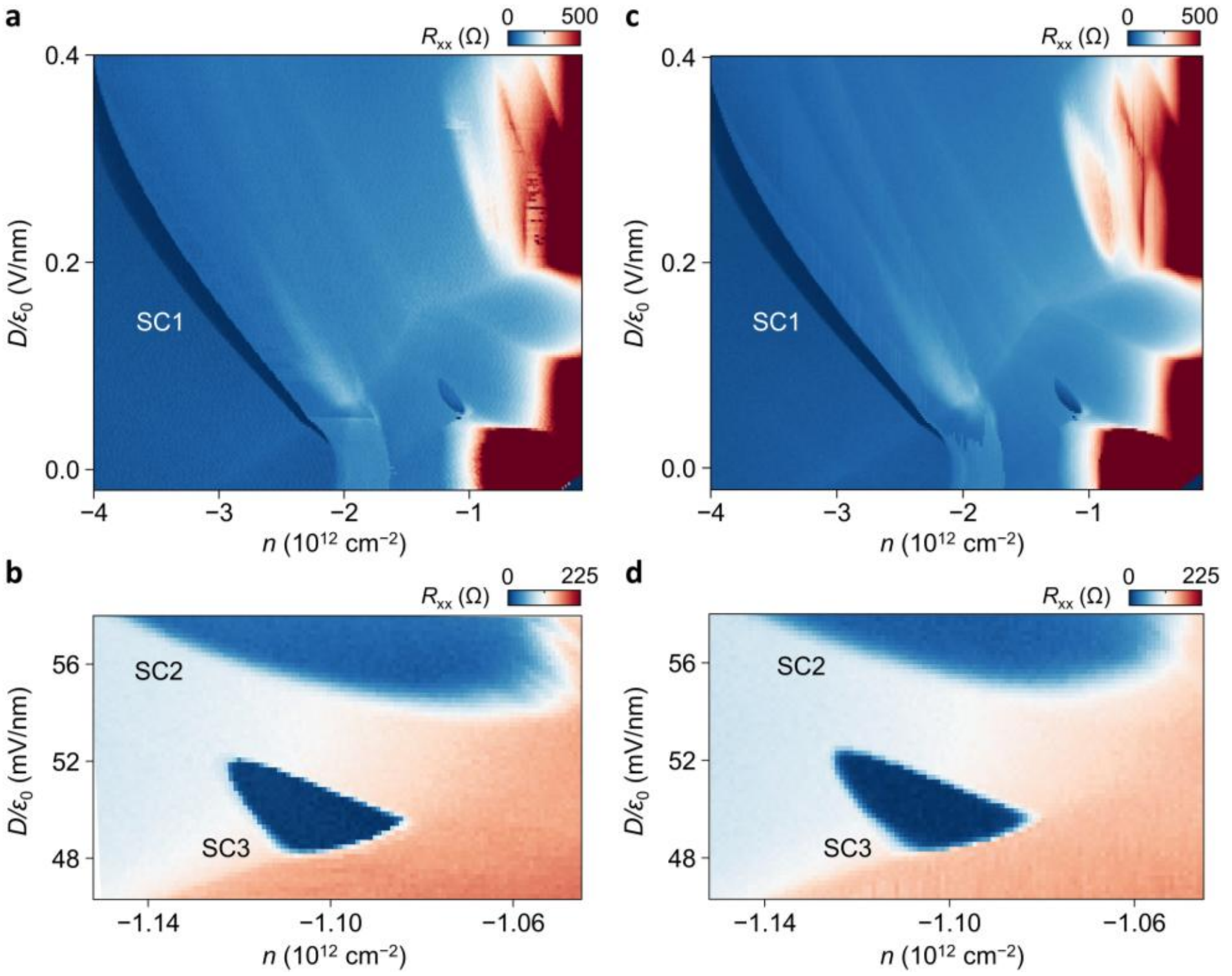

***Figure R32. Measurements of Device R5G-2 in the setup at MIT (a,b) and the University of Basel (c,d).** The superconducting states SC1-SC3 are clear in both of the maps.*

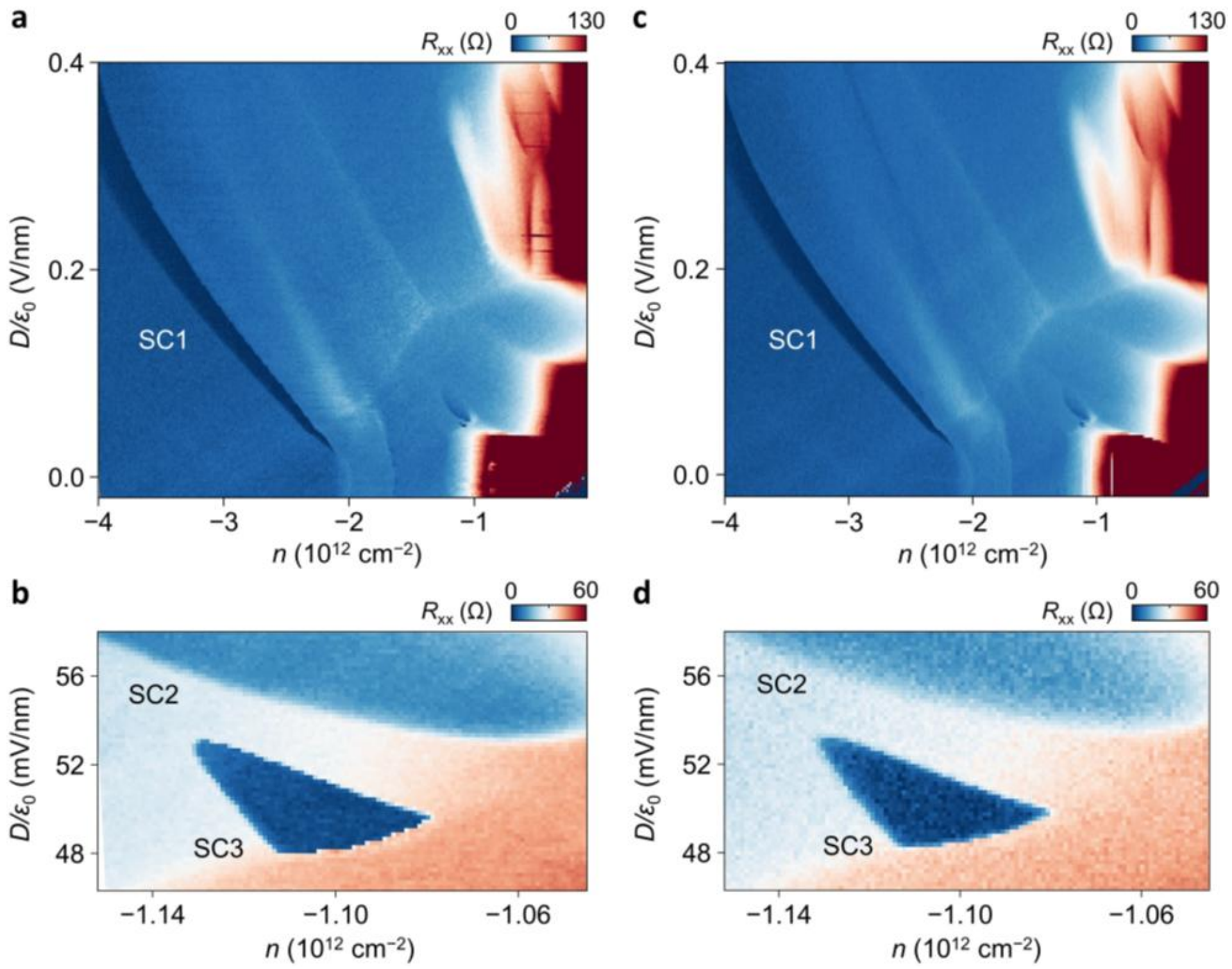

***Figure R33. Measurements of Device R5G-3 in the setup at MIT (a,b) and the University of Basel (c,d).** The superconducting states SC1-SC3 are clear in both of the maps.*

The authors provide an extensive discussion of the evolution of SC2 with the in-plane magnetic field (Fig. 2a), arguing that the superconductivity is enhanced. However, by comparing 0T and 8T panels, one can see that the normal state itself is strongly modified by the high in-plane magnetic field. At 8T, the entire SC2 region and the adjacent resistive metallic state are strongly shifted to higher displacement fields. In fact, one can strategically pick a (n,D) point for which a small in-plane magnetic field suppresses rather than enhances the superconductivity.

Response 38

We appreciate the referee for this valuable comment. It is true that the global shift of the phase in the *n-D* space by $B_\parallel$ can lead to the misinterpretation. Although we presented some logics for the fair comparison of SC2 at different values of $B_\parallel$ in the previous manuscript, we here demonstrate further evidence to clarify this issue. Firstly, SC2 at $B_\parallel$ = 8.0 T is expanded in the *n-D* space and reaches lower R_{xx}, compared to the data taken at $B_\parallel$ = 0.0 T. Figure R34a and R34b compare the R_{xx} map focusing on SC2 at $B_\parallel$ = 0.0 and 8.0 T. The area of SC2 at nonzero $B_\parallel$ is larger and its resistance clearly decreased globally across the whole phase. Secondly, the transition temperature of the (*n*, *D*) point featuring the strongest SC2 increases as $B_\parallel$ is applied. For this comparison, we took the (*n*, *D*) points exhibiting the lowest R_{xx} across the SC2 phase at each $B_\parallel$ and compared their transition temperature, as shown in Figure R34c. The resistance starts to drop at higher *T* at $B_\parallel$ = 8.0 T, suggesting the transition temperature increased by finite $B_\parallel$.

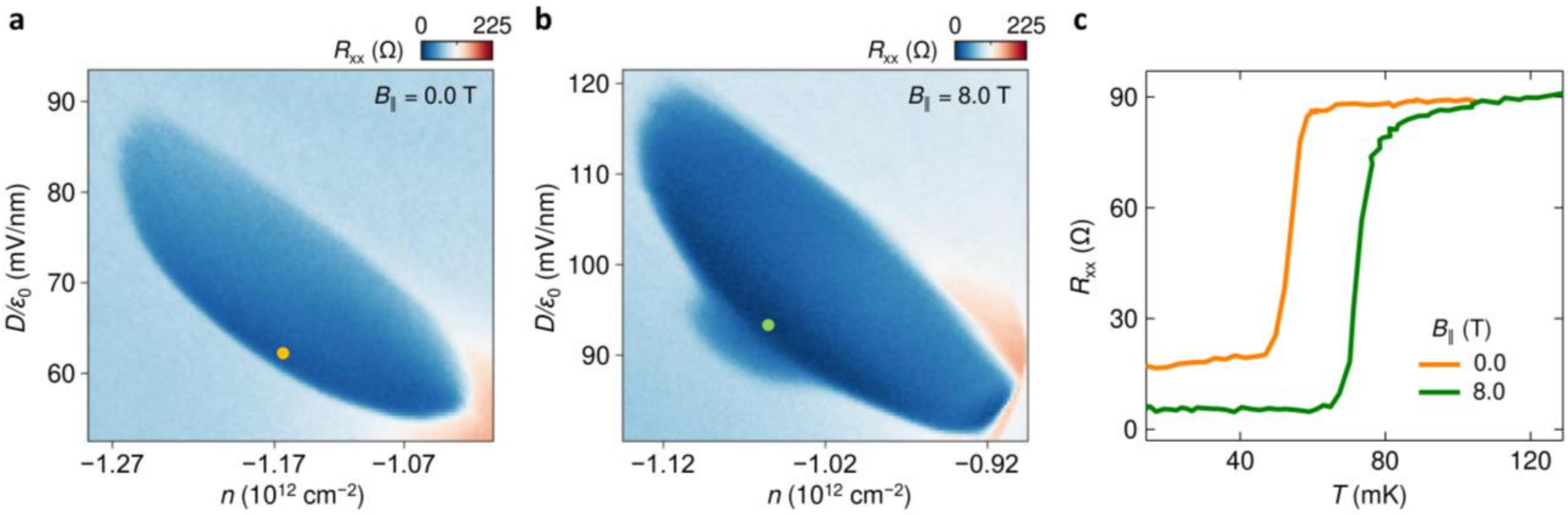

Figure R34. Further effort for the fair comparison of SC2 at B$_\parallel$ = 0 and 8 T. a,b, *R_{xx} map of SC2 in Device R5G-2 taken at $B_\parallel$ = 0.0 (**a**) T and 8.0 (**b**) T. The area of SC2 in the n-D space is larger and its R_{xx} is globally lower at $B_\parallel$ = 8.0 than 0.0 T.* ***c,*** *Temperature-dependent R_{xx} at the (n, D) point with the lowest R_{xx} of SC2 at $B_\parallel$ = 0.0 and 8.0 T in Device R5G-2. The drop of R_{xx} is found at higher T for $B_\parallel$ = 8.0 T. Each (n, D) point is shown in **a&b** with the corresponding color.*

Since the underlying band structure is strongly modified by the field, it is difficult to separate the direct effect of the field on the SC state from the indirect effect via the altered normal state. I think this should be directly acknowledged in the discussion.

Response 39

We appreciate the referee for pointing out this concern. As the referee suggested, an in-plane magnetic field impacts a superconducting state in two ways. Firstly, it affects the superconductor gap Δ depending on the spin configuration of a Cooper pair. For a spin-singlet Cooper pair, Δ decreases as $B_\parallel$ is applied due to spin Zeeman energy. For a spin-triplet case, Δ is mostly unaffected by $B_\parallel$. We believe this is the "direct

effect of the field on the SC state" mentioned by the referee. Secondly, $B_{\parallel}$ modifies the details of a parent state. The modified parent state can give rise to a larger superconductor gap. We suppose this is the "indirect effect via the altered normal state" put by the referee.

As discussed above, when a Cooper pair is spin-triplet, the superconducting state does not gain energy difference from the normal state by $B_{\parallel}$. In a naïve picture, therefore, $B_{\parallel}$ will not enhance SC by the first effect. This leads to our hypothesis that the indirect effect via the altered normal state may result in the enhancement of SC2 by the in-plane field, in that the normal state of SC2 is likely to be spin-polarized. However, it is challenging for only transport measurements to fully distinguish the direct and indirect effect by $B_{\parallel}$.

In the revised manuscript, we added a subsection "Effects of $B_{\parallel}$ on a superconducting state" in the Methods section that discusses the impacts of $B_{\parallel}$ in detail. Based on this, we added sentences in Discussions and outlook: "Considering the alteration of adjacent states under a high in-plane field, we hypothesize that change in the normal states by $B_{\parallel}$ may result in the enhancement of SC2 and induction of SC4 (Methods)."

The data for SC2 shows that the resistance of this state does not go to zero, but in some cases only decreases by about half (e.g., from ~30 to ~15 Ohm in Fig. 2 c and h). Moreover, if one compares SC2 to SC1 and SC3, all three states have comparable critical currents and transition temperatures, but the resistance approaches zero only for SC1 and SC3, but not for SC2. Fig 2h also shows that the resistance in SC2 has some strong carrier-density dependence. All these anomalies suggest a possibility that SC2 might not be a superconducting state, but some distinct symmetry-broken metal, as was discussed above.

Response 40

We appreciate the referee for pointing out this concern. Firstly, we would like to note that the carrier-density dependence of R_{xx} in a graphene superconductor is fairly common. For examples, Figure R35a and R35b present SC1 in R4G and R5G, respectively. Both of them show R_{xx} varying with n. One possible origin of such carrier-density dependence is that the pairing strength depends on (n, D). Secondly, the saturation of R_{xx} depended on a measured sample. Figure R35c shows the superconducting transition of SC2 in Device R5G-2. The resistance suddenly drops from ~90 to ~5 Ω across the transition temperature, exhibiting the decrease by ~95%.

The phenomenon in which R_{xx} gets saturated at some temperatures is not fully understood. Such examples can be observed in several publications (eg. Fig. 4c-e of [H. Zhou et al. *Nature* **598**, 434-438 (2021)], Fig. 1e of [L. Holleis et al. *Nat. Phys.* **21**, 444-450 (2025)], Fig. 4b of [Y. Zhang et al. *Nature* **641**, 625-631 (2025)], Fig. 3b&4b [Y. Guo et al. arXiv:2511.17423v1 (2025)]). One of the possible reasons is the disequilibrium of the electron system with the lattice [L. Holleis et al. *Nat. Phys.* **21**, 444-450 (2025)], where the electrons cannot be cooled down well by insufficient noise filtering and finite contact resistance. In particular, we hypothesize the contact plays an important role in the quality of SC data. For example, the resistance R_{xx} and nonlinear dV_{xx}/dI at the base temperature for Device R4G/WSe_2 (Fig. R30) show notable quantitative differences in the two tested measurement configurations. Although the origin of saturated R_{xx} is not clear yet, we believe the quasi-periodic modulations of SC2 by $B_{\perp}$ in Figure R24d-f provide additional evidence for its superconducting nature.

Having said all these, to be fully transparent with the readers on this nonzero residual resistance issue, we added a sentence in the revised manuscript as 'The residual resistance relative to the normal-state resistance in some of the superconducting states, such as SC2 and SC4 in R5G, is relatively large

compared to the others.' in the subsection 'Fraunhofer-like interference patterns and nonzero residual resistance' in the Methods Section. Figure R35c is also added to Extended Data Figure 3i.

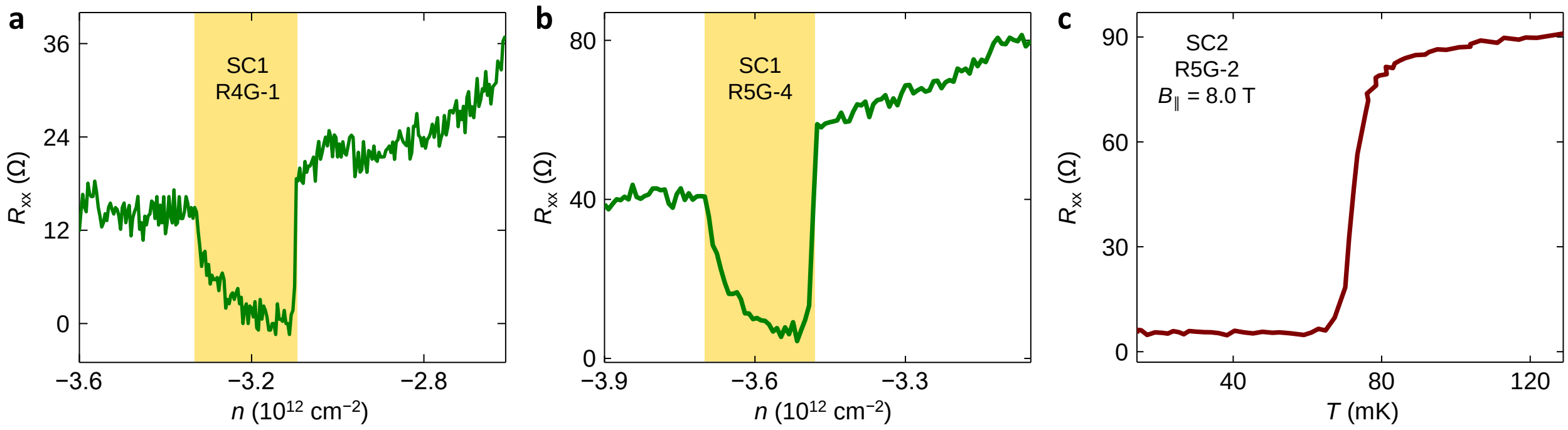

Figure R35. Carrier-density dependence of SC. a,b, *R_{xx} of SC1 in Device R4G-1 (D/ε_0 = 0.42 V/nm) (**a**) and R5G-4 (D/ε_0 = 0.225 V/nm) (**b**) as a function of n. The resistance of SC1 at heavier hole doping is higher within the same SC phase.* ***c,*** *Temperature-dependent R_{xx} of SC2 in Device R5G-2 ($n = -1.055 \times 10^{12}$ cm^{-2}, D/ε_0 = 93.17 mV/nm) at $B_\parallel$ = 8.0 T. The resistance in this device shows a very sharp drop across the SC2 transition and approaches nearly zero resistance. The measurements were performed at high $B_\parallel$ to strengthen SC2.*

Fig.3b shows the Fourier transform of the resistance as a function of 1/B. However, in contrast to other similar plots in the paper, the authors do not provide the corresponding original data R(B,n). It is also not specified at what displacement field this data is taken.

Response 41

We followed the suggestion and attached the corresponding original data in Figure R36. This data was taken at D/ε_0 = 54 mV/nm. In the revised manuscript, we added Figure R36 with the value of displacement field in Extended Data Fig. 9d.

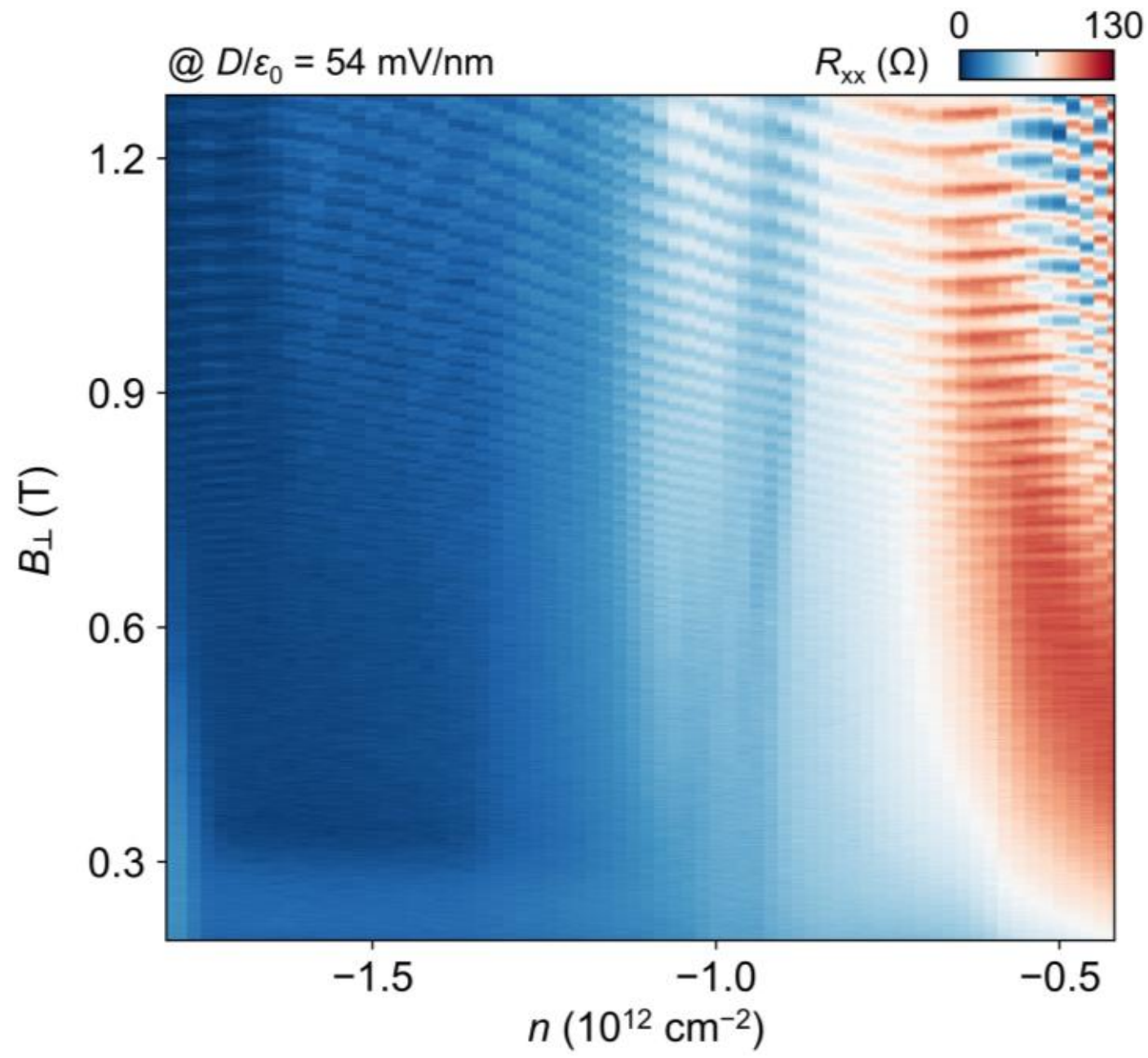

Figure R36. The original data of Fig. 3b in the original manuscript before the Fourier transform.

In the second-to-last paragraph of the main text, the authors discuss violation of the Pauli limit and field-

enhanced superconductivity, citing some references on these observations in non-graphene systems. From reading this paragraph, one might get the impression that similar observations have never been made in graphene systems before. However, this is not the case. The Pauli limit violation has been reported in twisted trilayer graphene (Cao et al. Nature 595 (2021)) and in rhombohedral trilayer graphene (Zhou et al., Nature 598 (2022)). The superconductivity induced by in-plane field was previously reported in Bernal bilayer graphene (Zhou et al., Science 375 (2022)), and the authors cite this earlier in the manuscript.

Response 42

We appreciate the referee for this comment. The original purpose of this paragraph in "Discussions and outlook" was to compare our results with different materials in a broad range instead of graphene-based systems only. However, we agree that this may give readers an impression that unconventional SCs have not been studied in other graphene-based systems.

In the revised manuscript, we added discussions about twisted trilayer graphene [Y. Cao et al. *Nature* **595**, 526-531 (2021)] and SC2 in R3G [H. Zhou et al. *Nature* **598**, 434-438 (2021)] as 'Secondly, notable PVR has been reported in twisted trilayer graphene[21] and R3G[36] without strong SOC. However, their demonstrated PVRs are smaller than the lower bound of SC3 in R5G (>35) that is not even enhanced by $B_{\parallel}$.' in the sub-section "Discussions and outlook." SC in R2G without proximitized SOC effect [H. Zhou et al. *Science* **375**, 774-778 (2022)] is also discussed as 'However, bare graphene lacks magnetic elements and the aforementioned mechanisms cannot be directly applied. Considering the alteration of adjacent states under a high in-plane field, we hypothesize that change in the normal states by $B_{\parallel}$ may result in the enhancement of SC2 and induction of SC4 (Methods). This might also be the case for SC in R2G[32] that appears only under finite $B_{\parallel}$' in the same subsection.

Responses to the referees

We sincerely thank the referees for taking the time to assess our manuscript and raising suggestions to improve it. We believe that this letter and the revised manuscript properly addressed their questions and comments. Most importantly, we have refined the theoretical model that can explain the enhancement of SC3 in R5G by an out-of-plane magnetic field. The document describing the theory model and calculations *before* the current round of revision is also attached after the end of this response letter.

Referee #1 (Remarks to the Authors):

The authors have answered my questions satisfactorily. They also present data on different R4G and R5G devices, with the conclusions broadly consistent with the original claims, assuaging concerns of reproducibility. The authors also present data at a lower electronic temperature for R4G/WSe2, which allowed them to study more fragile superconducting states (such as SC5) that were not convincingly demonstrated in the first version of the manuscript. Finally, the addition of magnetic field response of the various R4G/WSe2 superconductors reveals very different phenomenology (no field enhancement) from the hBN devices, an important piece of information to constrain theoretical models.

To recap the main criticisms (from all the referees) in the first round of review: (1) The presence of various superconducting states in this system is not really unexpected, given the vast family of known rhombohedral graphene superconductors. (2) The manuscript leaves central questions of interpretation outstanding (for example, the reason behind the reported out-of-plane enhancement of superconductivity). The authors provide more context for these observations in the revised manuscript by ruling out some natural explanations, but they do not flesh out a reasonable candidate scenario – leaving it to future theory work – which feels unsatisfying.

With these caveats in mind, I believe the work uncovers sufficiently new and unconventional superconducting phenomenology in hole-doped R4G and R5G to capture the attention of the community. The experimental results are very extensive and will drive the field forward in search of a unifying theoretical explanation for the mysterious observed phenomena. For this reason I am inclined to recommend publication of the work in Nature.

Response 1

We thank the referee for appreciating the merits of our work and the recommendation for publication in *Nature*. We understand the lack of a reasonable candidate scenario previously was a shortfall. To address this, we have proposed a possible mechanism for the $B_\perp$-induced enhancement of SC3. We have also improved it based on the referee's comments, as discussed in the Section "Referee #1 (Comments on theoretical modelling)".

I have two detailed comments below:

(1) When the authors discuss the effect of Hund's coupling in the discussion, I would emphasize (to contrast with WSe2 devices): "since the SOC of graphene is much weaker than Hund's coupling" -> "since the intrinsic SOC of graphene is much weaker than Hund's coupling"

Response 2

We appreciate the referee for the suggestion. We changed the corresponding sentence as the referee suggested to 'Since the intrinsic SOC of graphene is much weaker than Hund's coupling favoring the spin polarization of half-metals, this suggests SC2-4 are spin-polarized differently from Ising SC.'

(2) In response to my previous comments, the authors carried out additional measurements and emphasize that "No enhancement of SC by B⊥ was observed in any of the SCs in R4G/WSe2." This seems like a key piece of information to constrain future theoretical explanations of the B⊥ enhancement reported in the hBN devices. It should be pointed out more clearly in the main text (apologies if I missed it) and referred to the appropriate data in the SM Figures.

Response 3

We thank the referee for the comment. We note that the anomalous phenomenology of SCs under magnetic fields is found in *SC3 in R5G* rather than R4G, and the effect of proximitized spin-orbit coupling (SOC) on SCs was examined in the Device R4G/WSe_2 in the previous manuscript. We did not observe any superconducting state similar to the SC3 of R5G in our bare R4G devices.

In order to investigate how SC3 in R5G changes when it is proximitized by a transition-metal dichalcogenide, we measured a new Device R5G/WS_2 (Fig. R1a) during this round of revision. Both WSe_2 and WS_2 give the same form of proximitized SOC in the Hamiltonian of graphene [Li, Y. & Koshino, M. *Phys. Rev. B* **99**, 075438 (2019)]. As shown in Fig. R1b and R1c, we can observe SC2 at the WS_2-proximal side while SC3 appears to be very weak. The origin of the change in the strength of SC2 and SC3 requires further systematic studies, which is beyond the scope of the current response.

We further study whether the SC3 in this R5G/WS_2 device shows similar $B_\perp$ enhancement as observed in the R5G device. As shown in Fig. R1d, the critical current of this weak SC3 state is suppressed monotonically by $B_\perp$. Therefore, the enhancement by $B_\perp$ is absent with the proximitized SOC effect. This is consistent with our theory model, which will be discussed in **Response 10**.

In the revised manuscript, we included the additional data from the Device R5G/WS_2 (Fig. R1) in Extended Data Fig. 9. Following the suggestion by the referee, we explicitly mentioned in the main text that "This suggests the enhancement of SC3 be suppressed when strong SOC is proximitized to R5G. This prediction is also consistent with the absence of the increase in I_c by $B_\perp$ for SC3 in R5G/WS_2 (Extended Data Fig. 9o)." in the subsection "SC3 enhanced by $B_\perp$". The out-of-plane field responses of SCs from the Device R4G/WSe_2 are included in the manuscript (Fig. 4b-g, Extended Data Fig. 10p-s).

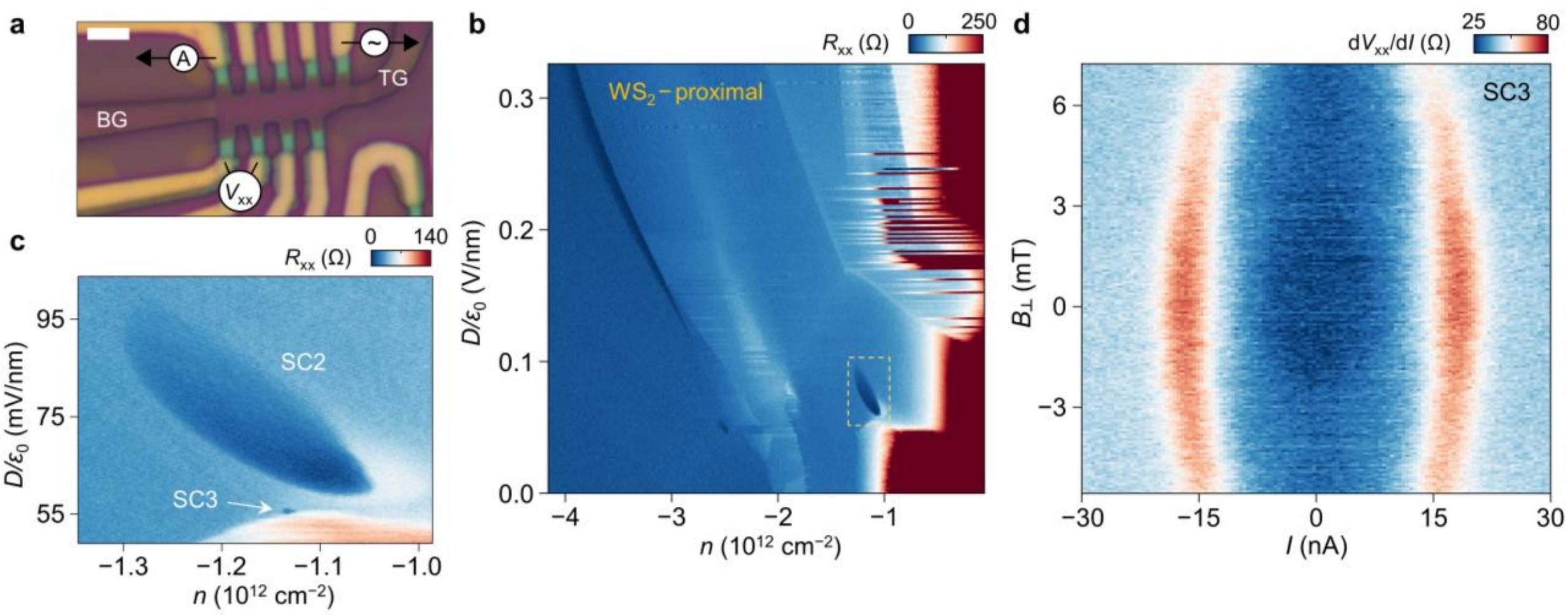

Figure R1. SCs in SOC-proximitized R5G. a, Optical image of the Device R5G/WS_2 and the representative

measurement configuration. Scale bar: 3 μm. **b,** R_{xx} map of the device. **c,** R_{xx} map in the yellow dashed box in **b**. A weak SC3 is observed from this device. **d,** Differential resistance as a function of I and $B_\perp$ for SC3 ($n = -1.133 \times 10^{12}$ cm^{-2}, $D/\varepsilon_0 = 55.7$ mV/nm). No enhancement by $B_\perp$ was observed from SC3 in this device.

Similarly, the in-plane field response of R4G/WSe2 could make it into an additional SM Figure (combining some representative data from the reply figures R8-12)

Response 4

We present the in-plane field responses of Device R4G/WSe_2 in Fig. R2. We have combined it with the representative data from the Figures R8-12 in the previous round of revision and included it as Extended Data Fig. 11 in the revised manuscript, as suggested by the referee.

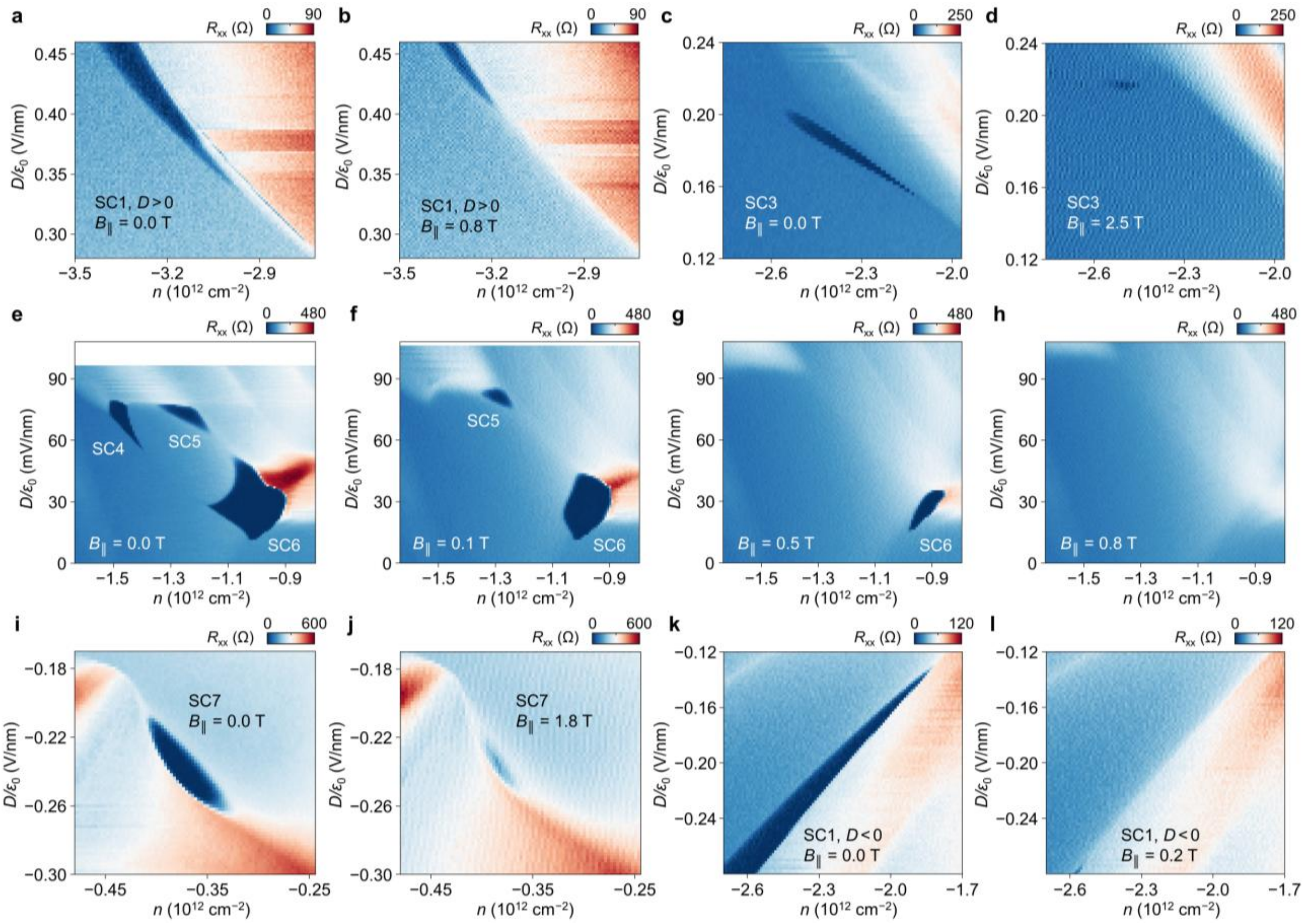

Figure R2. $B_\parallel$ responses of SCs in R4G/WSe_2. a,b, SC1 at the WSe_2-proximal side for $B_\parallel$ = 0.0 (**a**) and 0.8 (**b**) T. **c,d,** SC3 for $B_\parallel$ = 0.0 (**c**) and 2.5 (**d**) T. **e-h,** SC4-6 for $B_\parallel$ = 0.0 (**e**), 0.1 (**f**), 0.5 (**g**) and 0.8 (**h**) T. **i,j,** SC7 for $B_\parallel$ = 0.0 (**i**) and 1.8 (**j**) T. **k,l,** SC1 at the WSe_2-distant side for $B_\parallel$ = 0.0 (**k**) and 0.2 (**l**) T. All the observed SCs are monotonically suppressed by applying $B_\parallel$.

Referee #1 (Comments on theoretical modelling):

The authors propose a mechanism to explain the enhancement of pairing by out-of-plane magnetic fields in their experiment. The main assumptions are that (i) the dominant superconducting pairing channel is intra-valley, (ii) the competition between intrinsic Kane-Mele spin-orbit coupling and Hund's interaction

makes the underlying half-metallic ground state spin canted, and (iii) the band structure is such that a substantial variation of the density of states occurs when the chemical potential \mu in a given valley is slightly modified.

Overall I think the modeling is reasonable and provides a valuable addition to the manuscript. It helps contextualize the observations and makes connections to the recent literature on the interplay between spin-orbit coupling, interactions and superconductivity in graphene multilayers. It can also reproduce the detailed behavior under the combined weak B_perp and B_parallel fields that the authors also report.

Response 5

We sincerely appreciate the referee for supporting the validity and value of our theory model. We revised the proposed model based on the referee's comments as explained in the following responses.

I have a few questions and suggestions:

(1) The authors assume that the magnitude of spin polarization in each valley remains identical. This assumption is likely justified for the analytical scaling derived at small applied magnetic fields, but is nevertheless inconsistent with the symmetry of the problem in the presence of both B_perp and Ising SOC. When the authors present the numerical solution of F, do they allow for a fully general solution for the n_{+/-}, or do they also enforce identical magnitude n_0 in both valleys?

Response 6

This is a great point. In the original theory model before this revision, we made an approximation as $n_+ = n_-$ to capture the analytical scaling derived at small fields, as pointed out by the referee. Although the previous calculations resulted in the valley imbalance by the nonzero spin polarization s_z and the valley-contrasting energy $\pm\lambda s_z/2$, we agree that starting with this assumption is inconsistent with the symmetry of the problem, as pointed out by the referee. To address this issue, we allowed for a fully general solution (i.e., $n_+ \neq n_-$ where $n_+ + n_- = n$) in the calculations introduced in **Response 8**. In short, we incorporated the possibility for $n_+ \neq n_-$ when writing down the spin polarization vectors $\vec{n}_\pm$ as $\vec{n}_+ = (n_0 + \delta)(\sin\theta_+, 0, \cos\theta_+)$ and $\vec{n}_+ = (n_0 - \delta)(\sin\theta_-, 0, \cos\theta_-)$. As shown in **Response 8**, the new solution does not change the prediction of the model qualitatively.

(2) In a similar vein, I am wondering whether the neglect of the orbital coupling to B_perp is justified. As the authors explain, the valley Zeeman coupling is expected to be stronger than the spin Zeeman coupling used in the analysis.

Response 7

We thank the referee's comment. The physical reason we neglected the orbital coupling to $B_\perp$ was because the energy correction λs_z (~50 μeV) from the SOC by the $B_\perp$-induced spin polarization s_z is larger than the valley Zeeman energy $h_V = g_V \mu_B B_\perp$ (~1 μeV at $B_\perp = 2$ mT) by an order of magnitude. In the revised model (**Response 8**), we included the valley Zeeman coupling in the free energy to test the model in a complete way. We found that the valley Zeeman effect barely changes the optimal $B_\perp$ value corresponding to the strongest SC3, but it slightly affects the valley imbalance at $B_\perp$ values higher than this threshold.

(3) To resolve the above two points, I suggest that the authors compute the valley imbalance in their model as a function of B_perp. In my view this quantity is most directly linked to their proposed explanation in terms of valley-resolved modifications to the DOS.

As the authors explain, valley imbalance is correlated with out-of-plane spin polarization in systems with Ising SOC, but not always in a one-to-one correspondence. For example, in the fully spin-polarized phase shown for large B_perp in Fig. 1b: even though s_z is maximized (due to spins in both valleys pointing along z), there should always be a remnant valley imbalance originating from the interplay with Ising SOC. (While spins all point along +z, their valley populations are a bit different due to energy gain or penalty from ising SOC).

To be clear: I believe the salient point the authors are making is ultimately correct. I expect there will be a non-monotonous evolution of the valley imbalance as a function of B_perp, with a maximum likely around the critical field scale the authors obtain in Fig. 1b. But it would be helpful to present the valley imbalance from a general numerical solution of the free energy, similar to the SM of Kumar et al., https://arxiv.org/abs/2511.16578 (there for a different scenario where inter-valley pairing channel was assumed to dominate, valley polarization was supposed to be spontaneous, and only B_parallel considered). Addition of the orbital coupling to B_perp would also be valuable.

Response 8

We sincerely appreciate the referee's valuable comment. The referee's suggestions are (1) not to assume $n_+ = n_-$, (2) to include the valley Zeeman effect, and (3) to calculate the valley imbalance in addition to s_z. Reflecting these suggestions, we start from the free energy density F:

$$F = \frac{\kappa}{2}(n_+^2 + n_-^2) - J_H \vec{n}_+ \cdot \vec{n}_- - \frac{\lambda}{2}(n_+^z - n_-^z) - h_\perp(n_+^z + n_-^z) - h_\parallel(n_+^x + n_-^x) - h_V(n_+ - n_-).$$

Please note that the last term accounts for the valley Zeeman effect, while the other terms follow the previous model. Regarding the spin polarization vectors $\vec{n}_\pm$, we do not assume $n_+ = n_-$ and express them as $\vec{n}_+ = (n_0 + \delta)(\sin\theta_+, 0, \cos\theta_+)$ and $\vec{n}_+ = (n_0 - \delta)(\sin\theta_-, 0, \cos\theta_-)$. Here, $\theta_\pm$ describes the spin canting, and δ indicates the valley imbalance of hole occupations. Plugging these into F, we obtain F as a function of δ and $\theta_\pm$, and minimize it to obtain a solution for (δ, θ_+, θ_-).

$$F(\delta, \theta_\pm) = \kappa(n_0^2 + \delta^2) - J_H(n_0^2 - \delta^2)\cos(\theta_+ - \theta_-) - \frac{\lambda}{2}[n_0(\cos\theta_+ - \cos\theta_-) + \delta(\cos\theta_+ + \cos\theta_-)] - h_\perp[n_0(\cos\theta_+ + \cos\theta_-) + \delta(\cos\theta_+ - \cos\theta_-)] - h_\parallel[n_0(\sin\theta_+ + \sin\theta_-) + \delta(\sin\theta_+ - \sin\theta_-)] - 2h_V\delta.$$

We describe the parameters used in the following calculations. The Hund's coupling and SOC energy are the same to before: $J_H = 2$ meV/(10^{12} cm^{-2}) [Patterson, C. L. et al. *Nature* **641**, 632-638 (2025)] and $\lambda = 50$ μeV [Arp, T. et al. *Nat. Phys.* **20**, 1413-1420 (2024)]. The κ-term featuring the largest energy scale [Kumar, M. et al. arXiv:2511.16578 (2025)] and the valley g-factor g_V are affected by the band structure. To extract κ and g_V, hence, we used a 10-band Slonczewski-Weiss-McClure type tight-binding model and calculated the Fermi energy E_F, average energy per hole $\langle E_h \rangle$, and orbital magnetic moment averaged on the Fermi surface. We assumed a half-metal state with $n = -1.1 \times 10^{12}$ cm^{-2}, $D/\varepsilon_0 = 50$ mV/nm, which are the parameters where SC3 was observed. Table R1 shows the results from the calculations with two different sets of tight-binding parameters used in the literature [Zhou, H. et al. *Nature* **598**, 429-433 (2021); Han, T. et al. *Nature* **643**, 654-661 (2025)]. From these, we took $g_V = 6$ for the valley Zeeman term. The energy from the band structure could be used in estimating κ as $\frac{\kappa}{2}(n_0^2 + n_0^2) = \frac{\kappa n^2}{4} = \langle E_h \rangle |n|$ and thus $\kappa = 4\langle E_h \rangle / |n|$. This gave $\kappa \approx 5$ meV/(10^{12} cm^{-2}).

Ref.	γ_0 (eV)	γ_1 (eV)	γ_2 (eV)	γ_3 (eV)	γ_4 (eV)	E_F (meV)	$\langle E_h \rangle$ (meV)	κ	g_V
[1]	3.25	0.40	−0.0166	−0.293	−0.144	2.96	1.28	4.65	6.38
[2]	3.10	0.38	−0.0150	−0.290	−0.141	3.19	1.52	5.53	6.34

Table R1. Parameters used in the tight-binding model calculations to extract κ (in the unit of meV/(10^{12} cm^{-2})) and g_V. The isospin degeneracy of 2 is assumed. References: [1] Han, T. et al. *Nature* **643**, 654-661 (2025); [2] Zhou, H. et al. *Nature* **598**, 429-433 (2021). The two sets of the parameters give consistent results.

We first show s_z and δ by removing the assumption of $n_+ = n_-$ and including the valley Zeeman effect, as plotted in Fig. R3. Firstly, the value of $B_\perp$ resulting in the strongest SC3 decreases by directly incorporating the valley imbalance in the calculation. Nevertheless, the optimal $B_\perp$ value of ~1.1 mT is still fairly close to the experimental value showing the most enhanced SC3 (1.4 to 1.8 mT). Secondly, the optimal $B_\perp$ is barely affected by the valley Zeeman effect. This verifies the procedure to neglect the valley Zeeman term when previously calculating the optimal field (**Response 7**). However, we found that the valley imbalance is affected by the valley Zeeman effect. Figure R3b delineates the valley imbalance normalized by n_0 for $g_V = 0$ and 6. When the valley Zeeman term is turned on, the valley imbalance keeps increasing slowly above the optimal field, although s_z is saturated to the unity. This confirms the referee's comment that s_z and δ are not always in one-to-one correspondence. Nevertheless, such increase in δ is much slower than that driven by the spin canting, and the orbital depairing effect will probably become comparable to or more important than the valley Zeeman effect in experiments.

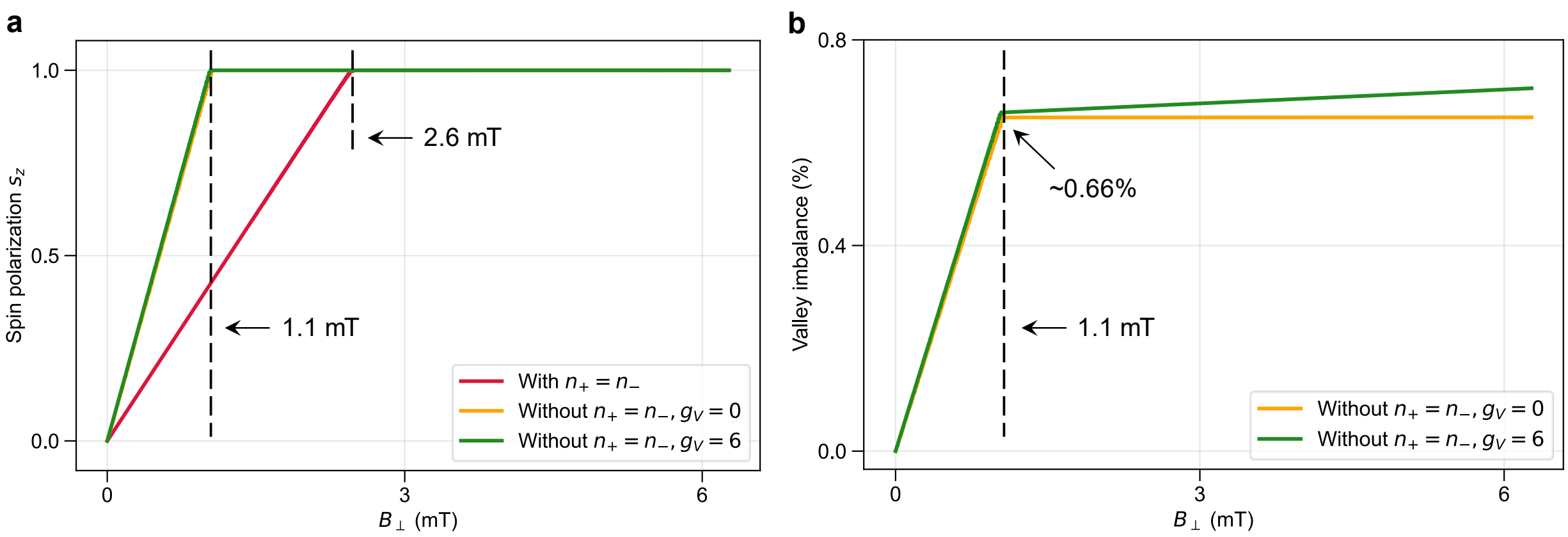

Figure R3. Effect of nonzero δ and valley Zeeman effect on the spin polarization and valley imbalance. a,b, Spin polarization s_z (**a**) and valley imbalance normalized by n_0 (**b**) as functions of $B_\perp$ for bare R5G when imposing the valley balance condition in the calculations (red), allowing the valley imbalance but without the valley Zeeman effect (orange) and with including the valley Zeeman effect (green). The optimal value of $B_\perp$, calculated to be 1.1 mT with allowing nonzero δ, is mostly independent of g_V. However, the valley imbalance for nonzero g_V keeps changing above the optimal field with a rate much lower than that driven by the increase in s_z.

Establishing that including the valley imbalance and valley Zeeman effect gives a reasonable value for optimal $B_\perp$, we now include the effect of $B_\parallel$ as shown in Fig. R4. Similar to the previous calculations, the rate at which the valley imbalance (and also the spin polarization) increases with $B_\perp$ is reduced by applying a small $B_\parallel$. This result is consistent with our experimental observations that the rise in the critical current by $B_\perp$ gets slower as increasing $B_\parallel$ from zero to a few mT (Fig. 3i in the revised manuscript).

In the revised manuscript, we first sketched the theory we proposed in the subsection "SC3

enhanced by $B_\perp$" as "We sketch one possible scenario that consistently explains all of our observations (see Methods for details). Under zero magnetic fields, the spin-polarized and valley-unpolarized normal state possesses the spin vectors canted with the angle $\varphi \approx 90°$ (Fig. 3h)[37], since Hund's coupling E_H (~2 meV) is much stronger than the intrinsic SOC λ of graphene (~50 μeV)[45-47] … This hypothesis is confirmed in Fig. 3i, where the maximum of I_c at $B_\perp$ ~1.8 mT is suppressed by $B_\parallel$ of merely few mT." The details of the model are explained in the subsection "Proposed mechanism for the enhancement of SC3 by $B_\perp$ based on SOC" in Methods and Extended Data Fig. 9.

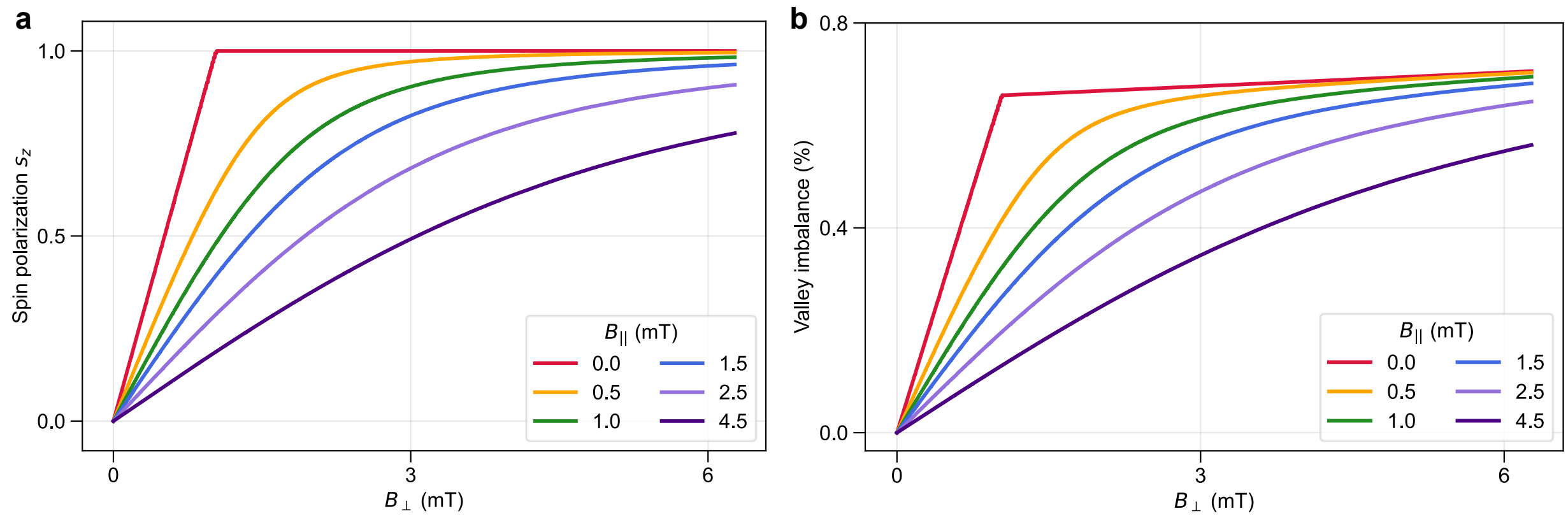

Figure R4. Effect of $B_\parallel$ on the valley imbalance. a,b, Spin polarization s_z (**a**) and valley imbalance normalized by n_0 (**b**) as functions of $B_\perp$ when is $B_\parallel$ applied. The increase in the valley imbalance by $B_\perp$ gets slower by applying $B_\parallel$, which is consistent with the experimental observations.

(4) Minor comment: the sentence: "Under $B_\parallel$, the electron spins are fixed to the in-plane direction. Hence, the SOC does not result in energy change: $\langle \rightarrow_x | \sigma_z | \rightarrow_x \rangle = 0$" is too strong. Ising SOC is a non-interacting term that lowers the symmetry of the fully in-plane polarized state otherwise obtained under B_parallel. So Ising will always cant the spins out of plane a little bit. This process is known to lower the energy quadratically in \lambda compared to a fully aligned state.

Response 9

We thank the referee for pointing this out. In the revised manuscript, we have deleted the original sentence when discussing the model and calculation results.

(5) Finally, I am hoping the authors can comment more explicitly on how their analysis changes when proximity-induced Ising SOC is strong (of order 1 meV). My sense is that this could tie the various measurements in the paper together (most notably the absence of B_perp enhancement in the WSe_2 proximitized devices).

Response 10

We appreciate the referee's comment. We computed the valley imbalance at two different values of SOC, λ = 0.05 meV to mimic bare R5G, and 1.0 meV to mimic SOC-proximitized R5G. Here we took 1.0 meV as a representative value from the reference [Zhang, Y. et al. *Nature* **641**, 625-631 (2025)]. As shown in Fig. R5b, the valley imbalance at the optimal field decreases by more than an order of magnitude by the SOC effect (0.66% to 0.05%). Furthermore, the sharp cusp developed at the optimal $B_\perp$ in the small-SOC

case disappears for λ = 1.0 meV. This result is consistent with the observed absence of $B_{\perp}$-induced enhancement of SC3 in the Device R5G/WS_2 (**Response 3**). The result in the valley imbalance can be more intuitively understood based on the spin polarization shown in Fig. R5a. The spin vectors are pinned more robustly when stronger SOC is imposed, since the Ising SOC acts as a valley-contrasting effective magnetic field. Therefore, a small $B_{\perp}$ of few mT can hardly induce the spin polarization and the valley imbalance arising from the valley-contrasting energy $\pm\frac{\lambda s_z}{2}$ is suppressed. Please note that the impact on the valley imbalance is less significant than that on s_z, due to the additional factor of λ in the valley-contrasting energy.

In the revised manuscript, we discussed the impact of proximitized SOC effect on the enhancement of SC3 in the subsection "Proposed mechanism for the enhancement of SC3 by $B_{\perp}$ based on SOC" in the Methods Section as "Another check of the theory can be performed by increasing the SOC strength through the proximation to a TMD. This is because the spin vectors are pinned more robustly when stronger SOC is imposed, as Ising SOC serves as a valley-contrasting effective magnetic field. … This is demonstrated in Extended Data Fig. 9e where the valley imbalance at optimal $B_{\perp}$ is reduced by an order of magnitude (0.66% to 0.05%)."

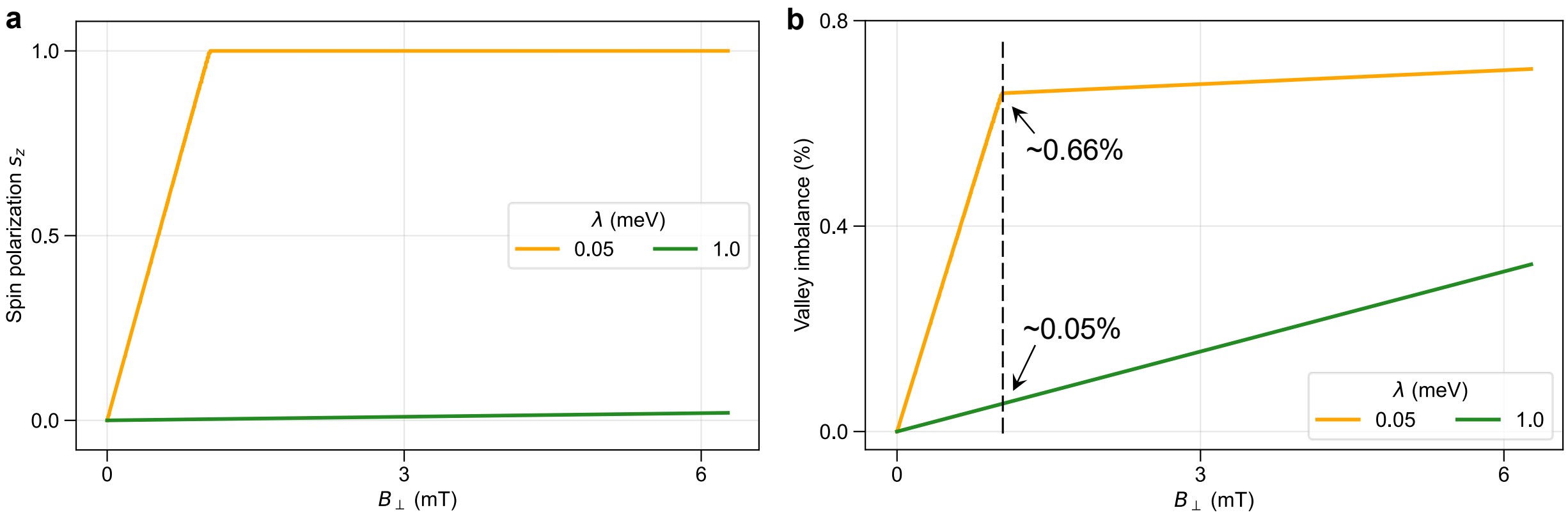

Figure R5. Effect of the proximitized SOC. a,b, Spin polarization s_z (**a**) and valley imbalance normalized by n_0 (**b**) as functions of $B_{\perp}$ for two different values of SOC strength. When the SOC is strong, the valley imbalance at the optimal field is reduced by an order of magnitude. This is in line with the observation that the enhancement of SC3 does not exist in SOC-proximitized R5G.

Referee #2 (Remarks to the Authors):

I appreciate the authors' efforts in revising the manuscript and responding to the referees' comments. However, after careful evaluation of the revised version and comparison with existing literatures, I remain unconvinced that the findings presented here constitute a sufficiently significant advance over prior work. Therefore, I cannot recommend publication in Nature, which requires a particularly high standard of novelty and conceptual impact. My assessment is based on the following points:

Response 11

We sincerely appreciate the referee's time to evaluate the manuscript and make the comments.

1. The enhancement of superconductivity under in-plane magnetic fields (SC2 and SC4) has already been extensively reported in multiple graphene-based systems, including bilayer graphene (Science 375, 774–778 (2022)), rhombohedral graphene (Nature 598, 434–438 (2021); Nature 643, 654–661 (2025), from the same group), and twisted trilayer graphene (Nature 595, 526–531 (2021)). While the authors may argue that their devices exhibit higher PVRs, this represents only a marginal quantitative improvement rather than a qualitatively new phenomenon. This does not, in my view, meet the threshold of significance required for publication in Nature.

Response 12

We respectfully disagree with the referee's comment. Firstly, none of the superconductors from rhombohedral graphene cited by the referee claim enhancements by an in-plane magnetic field. Figure R6 and R7 are the data taken from [Zhou, H. et al. *Nature* **598**, 434-438 (2021)] and [Han, T. et al. *Nature* **643**, 654-661 (2025)], respectively, showing the responses of SCs to $B_\parallel$. They do not show enhancements by $B_\parallel$. The authors of these two papers have not made any claims on the enhancements $B_\parallel$ either.

Secondly, the area in the *n*-*D* space occupied by the superconductivity in twisted trilayer graphene monotonically decreases by $B_\parallel$, as shown in Fig. R8a-c. This is in stark contrast to our observations in which the area occupied by field-enhanced SCs in the *n*-*D* space is increased by $B_\parallel$. At the same time, the PVR value of this SC (2 to 3) is substantially lower than the values reported in our work. We note that this is *not* only relevant to a quantitative improvement, since it has been proposed that the PVR in twisted trilayer graphene can be understood by its strongly coupled nature [Park, J. M. et al. *Nature* **590**, 249-255 (2021)] without considering spin-triplet pairing [Talantsev, E. F. *Materials* **16**, 256 (2023)].

Based on these, 3 out of the 4 publications cited by this referee did not show similar enhancements of superconductivity under in-plane magnetic field as we observed. Therefore, the comment "The enhancement of superconductivity under in-plane magnetic fields has already been extensively reported in multiple graphene-based systems" is false.

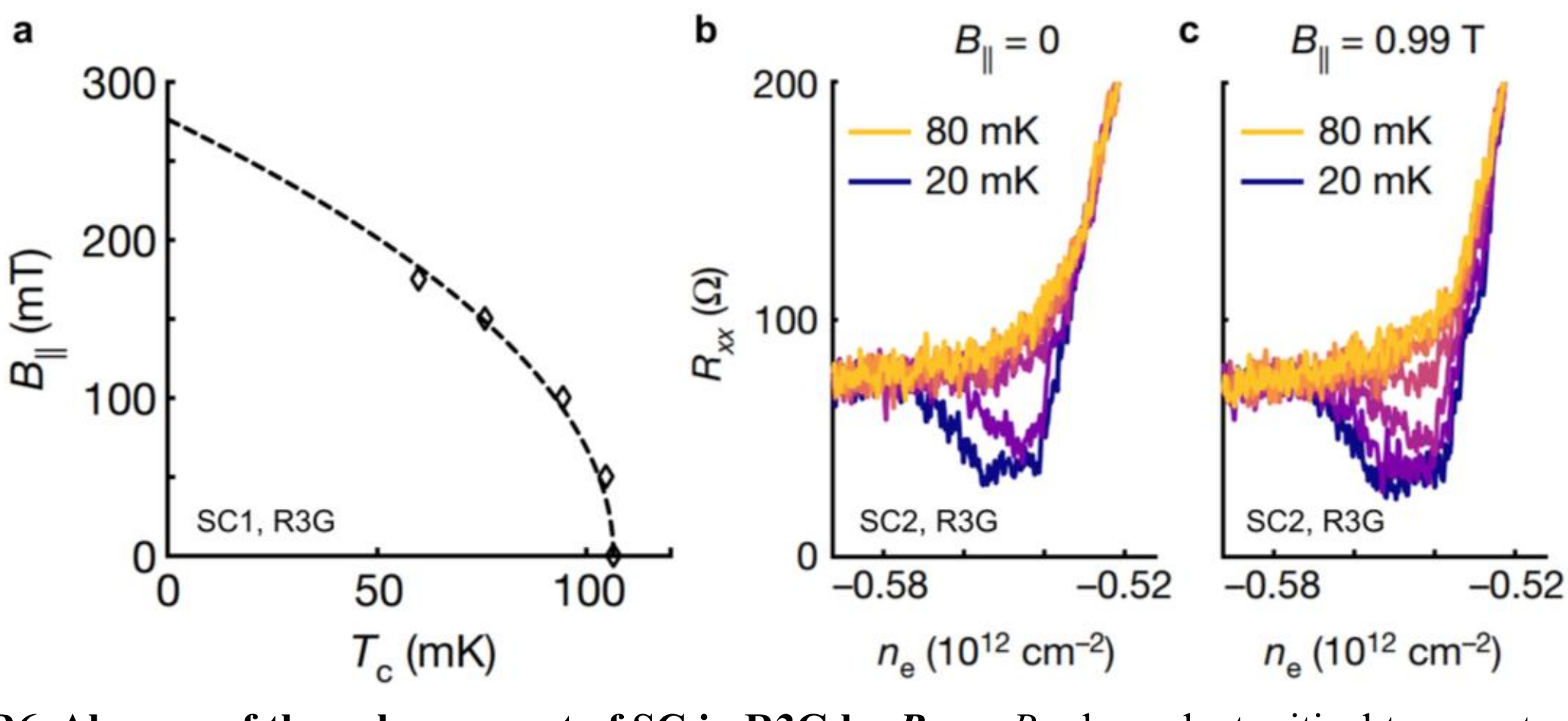

Figure R6. Absence of the enhancement of SC in R3G by $B_\parallel$. a, $B_\parallel$-dependent critical temperature of SC1 in R3G. T_c decreases monotonically by $B_\parallel$. **b,c,** Temperature-dependent R_{xx} of SC2 in R3G at $B_\parallel$ = 0 (**b**) and 0.99 T (**c**). There is no reported signature of enhancement by $B_\parallel$. The data are taken from [Zhou, H. et al. *Nature* **598**, 434-438 (2021)].

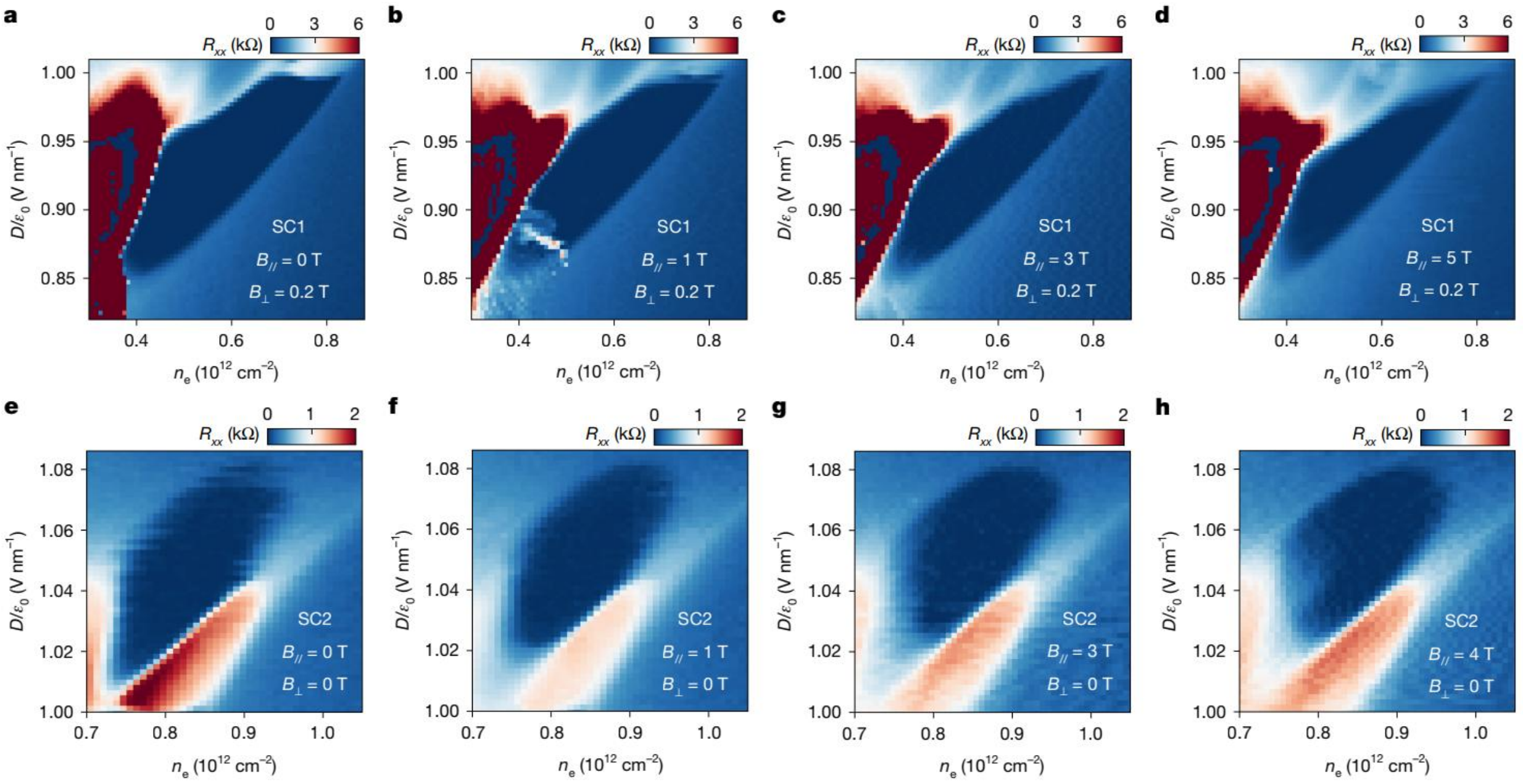

Figure R7. Absence of the enhancement of chiral SCs by $B_\parallel$. a-d, n_e-D maps of R_{xx} in chiral SC1 at $B_\parallel = 0$ (**a**), 1 (**b**), 3 (**c**) and 5 T (**d**) under $B_\perp = 0.2$ T to prevent the random fluctuations of R_{xx}. The area occupied by chiral SC1 decreases by $B_\parallel$. **e-h,** n_e-D maps of R_{xx} in chiral SC2 at $B_\parallel = 0$ (**e**), 1 (**f**), 3 (**g**) and 4 T (**h**). The area occupied by chiral SC2 decreases by $B_\parallel$. Overall, there is no signature of enhancement by $B_\parallel$. The data are taken from [Han, T. et al. *Nature* **643**, 654-661 (2025)].

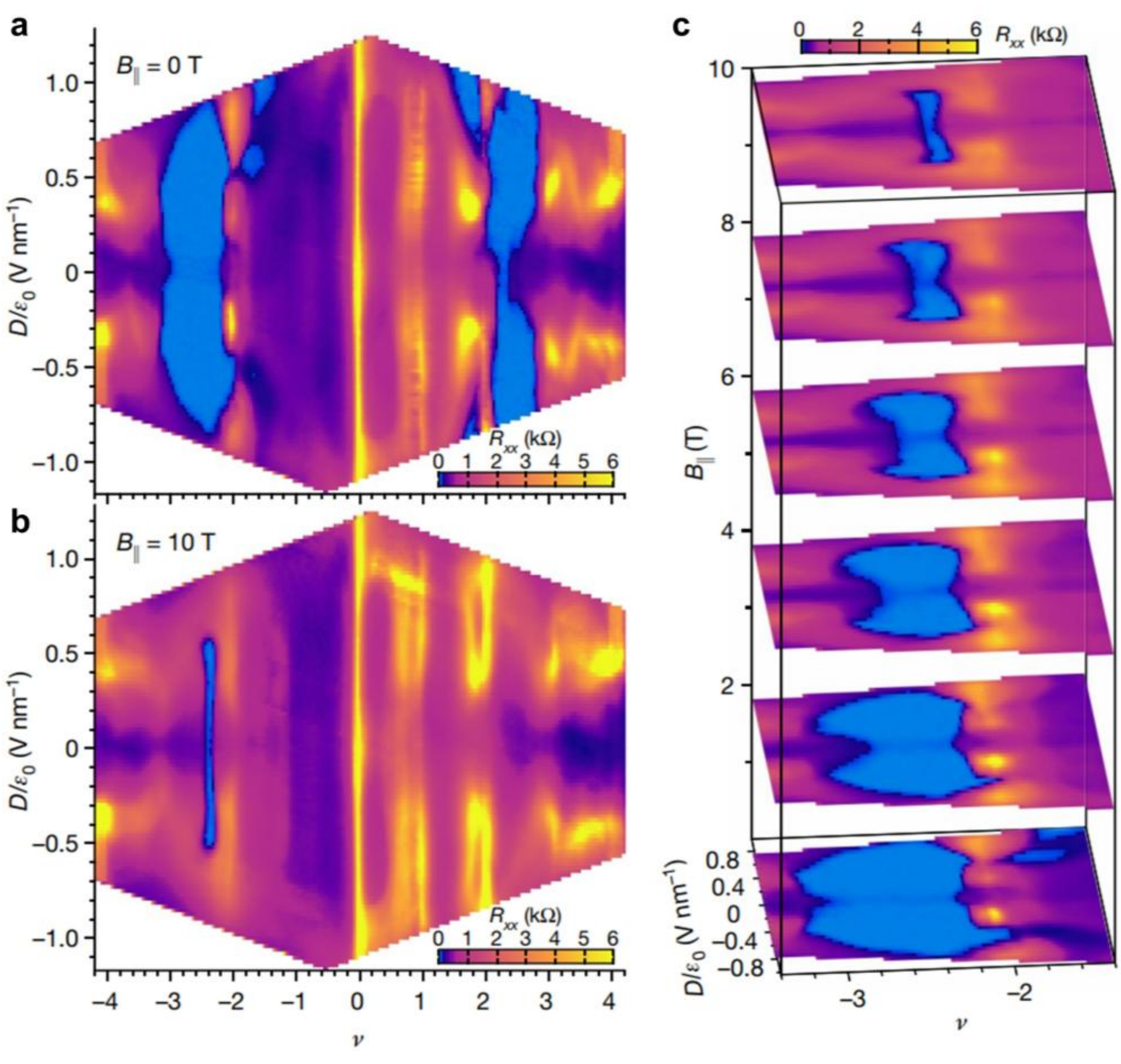

Figure R8. SC in twisted trilayer graphene under $B_\parallel$. a,b, R_{xx} at $B_\parallel = 0$ (a) and 10 T at $T = 300$ mK. **c,** Evolution of the SC on the hole-doped side as a function of $B_\parallel$. The area occupied by the SC decreases by $B_\parallel$.

The data are taken from [Cao, Y. et al. *Nature* **595**, 526-531 (2021)].

2. The SC3 state, emphasized by the authors and observed under perpendicular magnetic field in R5G, also lacks clear novelty. Similar behavior has been reported in other systems, such as $MoTe_2$ junctions (see Fig. 4 in Science Advances, eadq5712 (2025)), which the authors have not adequately acknowledged or discussed.

Response 13

We appreciate the referee for bringing up this $MoTe_2$ junction work, as it should have been compared to the SC3 we observed. However, we think the underlying mechanism of the $B_\perp$-induced enhancements of superconductivity is fundamentally different between that work and our work. In the reference [Jia, Y. et al. *Sci. Adv.* eadq5712 (2025)], the enhanced critical current is **only observed in twisted $MoTe_2$ ($tMoTe_2$) nanojunction (i.e., Pd_7MoTe_2-$tMoTe_2$-Pd_7MoTe_2), not in the superconducting material Pd_7MoTe_2 itself.** The absence of enhanced SC is shown in Fig. R9a&b for Pd_7MoTe_2 grown from natural bilayer and twisted bilayer $MoTe_2$, respectively. Instead, the authors of that paper have attributed their observation to the interference effect from the 0-π junction formed across the nanojunction. This junction mechanism has already been discussed extensively in literature [Weides, M. et al. *Phys. Rev. Lett.* **97**, 247001 (2006); Frolov, S. M. et al. *Phys. Rev. B* **74**, 020503(R) (2006); Yankowitz, M. et al. *Nature* **363**, 1059-1064 (2019); Ai, L. et al. *Nat. Commun.* **12**, 6580 (2021); Kang. K. et al. *Nano Lett.* **22**, 5510-5515 (2022)].

In contrast, the phenomenology of SC3 comes from the **superconductor itself** without any junction. We have already ruled out the 0-π junction scenario in the manuscript, assuming a situation that natural Josephson junctions were unintentionally formed in our devices due to sample inhomogeneity. All of our discussions in the manuscript suggest the observed phenomenology is intrinsic to SC3, which is distinct from the well-known 0-π junction mechanism. Instead, we have proposed a mechanism explaining the phenomenology of SC3 based on the intrinsic properties of R5G, such as Hund's coupling and intrinsic spin-orbit coupling (SOC).

To be constructive, we have followed the referee's suggestion to expand the discussion about 0-π junction and the specific work [Jia, Y. et al. *Sci. Adv.* eadq5712 (2025)] in our revised manuscript, by adding "The anomalous SC phenomenology under $B_\perp$ observed in some twisted bilayer graphene devices[19,65] and twisted $MoTe_2$ ($tMoTe_2$) nanojunctions[71] has also been understood as coming from a 0-π junction. For the $tMoTe_2$ nanojunctions, it was proposed that both spin-singlet and spin-triplet condensates exist across the Pd_7MoTe_2-$tMoTe_2$-Pd_7MoTe_2 junction (where Pd_7MoTe_2 is a superconductor without anomalous behavior), since $tMoTe_2$ breaks the inversion symmetry. Provided these two condensates had the phase difference by π, a 0-π junction is formed across the nanojunction and the SC transport phenomena may get enhanced by applying $B_\perp$." in the subsection "Ruling out other mechanisms for the enhancement of SC3 by $B_\perp$" in the Methods Section. We have also noted, however, the device setting and mechanism governing the anomalous enhanced superconductivity in our work is distinct from [Jia, Y. et al. *Sci. Adv.* eadq5712 (2025)], as "Regarding SC3 in R5G, if there are 0-π junctions in the devices due to sample inhomogeneity, the optimal $B_\perp$ value resulting in the strongest enhancement will notably depend on (n, D). This is because the normal-state area connecting two superconducting regions in the junction will expand as the SC gets weaker. However, the value of $B_\perp$ showing the strongest SC3 is independent of (n, D) in our data (Extended Data Fig. 7)."

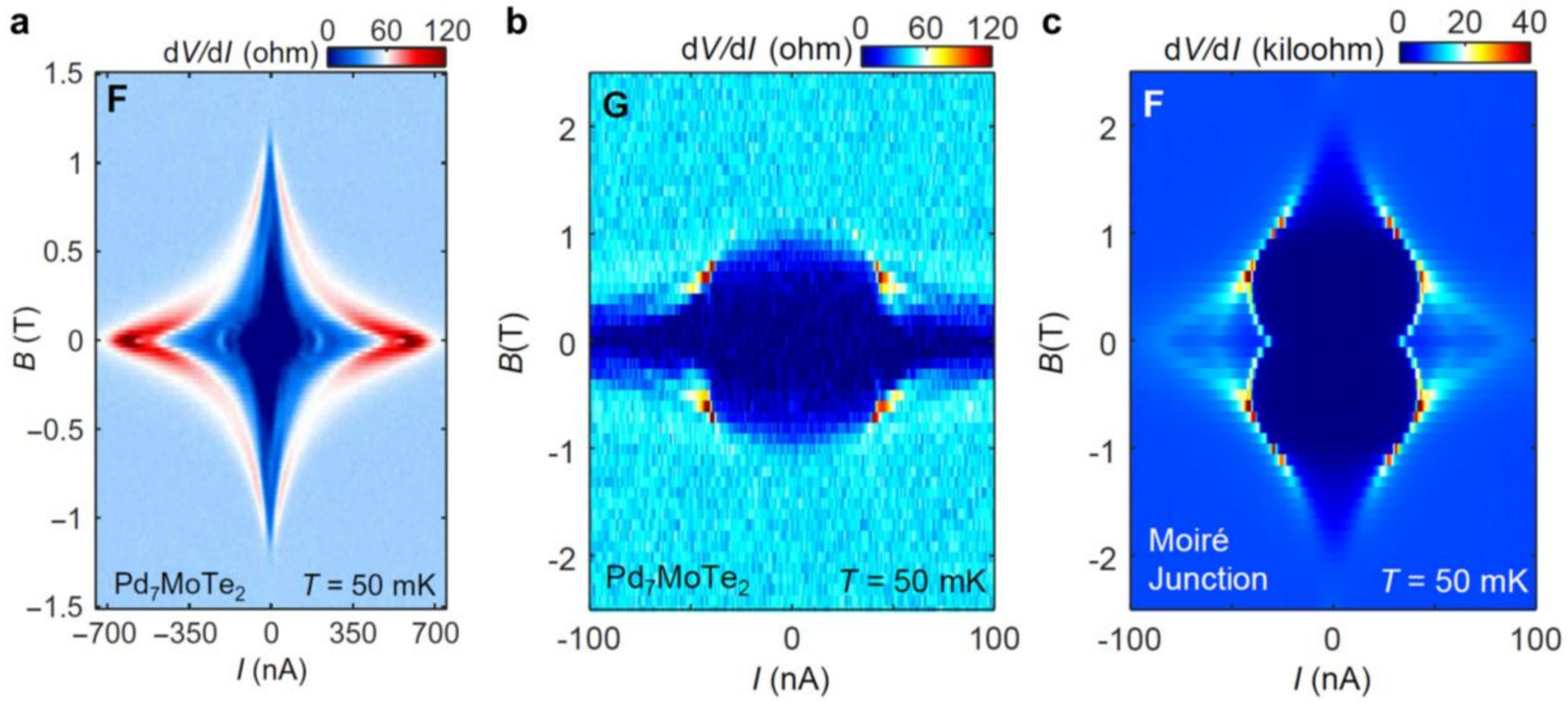

Figure R9. Phenomenology of Pd_7MoTe_2 and Pd_7MoTe_2-t$MoTe_2$-Pd_7MoTe_2 nanojunctions. a, Differential resistance of Pd_7MoTe_2 grown from natural bilayer $MoTe_2$. **b,** Differential resistance of Pd_7MoTe_2 grown from t$MoTe_2$ with the twist angle of 3.7°. **c,** Differential resistance across Pd_7MoTe_2-t$MoTe_2$-Pd_7MoTe_2 junction. The increase in the threshold current by $B_\perp$ only happens for the junction resistance, which was thus explained by the 0-π junction scenario. The data are taken from [Jia, Y. et al. *Sci. Adv.* **11**, eadq5712 (2025)].

3. A major concern from the first round was the absence of a convincing physical interpretation. This issue persists. Neither their experimental data nor existing theoretical frameworks sufficiently clarify the origin of SC3. As noted previously, the manuscript does not articulate a new physical principle or conceptual advance; rather, it remains largely a phenomenological report.

Response 14

We understand the referee's concern on the absence of a convincing physical interpretation, but this concern should be eliminated now, as we have proposed a possible mechanism for the enhancement of SC3 by $B_\perp$ in a separate document. We believe the theory we proposed gives conceptual advances in the graphene SC community, because the intrinsic SOC of graphene has not played a central role in the physics of graphene SCs so far.

In the revised manuscript, we sketched the theory we proposed in the subsection "SC3 enhanced by $B_\perp$" as "We sketch one possible scenario that consistently explains all of our observations (see Methods for details). Under zero magnetic fields, the spin-polarized and valley-unpolarized normal state possesses the spin vectors canted with the angle $\varphi \approx 90°$ (Fig. 3h)[37], since Hund's coupling E_H (~2 meV) is much stronger than the intrinsic SOC λ of graphene (~50 μeV)[45-47] … This prediction is also consistent with the absence of the increase in I_c by $B_\perp$ for SC3 in R5G/WS_2 (Extended Data Fig. 9o)." The details of the model are explained in the subsection "Proposed mechanism for the enhancement of SC3 by $B_\perp$ based on SOC" in Methods and Extended Data Fig. 9.

4. Schwinger-like nonlinear I–V characteristics have been reported in multiple prior works (Science 375, 430–433 (2022); Nature 614, 440–444 (2023); Nature Physics 19, 830–835 (2023); arXiv:2501.06460 (2025)). Such effects are expected in flat-band systems due to their extremely low Fermi velocity. The revised manuscript still does not convincingly exclude these non-superconducting mechanisms, leaving ambiguity in the interpretation of the observed states.

Response 15

We appreciate the referee for proposing another possible mechanism for nonlinear I-V_{xx} characteristics, which is brought up for the first time during the review and rebuttal process of this manuscript. Hence, we did not get a chance to discuss and rule out the Schwinger effect, but we are glad to address it in this round.

The Schwinger effect can indeed result in peaks in dV_{xx}/dI data. However, in contrast to peaks in dV_{xx}/dI from a superconductor, the peaks driven by the Schwinger effect do not disappear upon the application of a small $B_\perp$ of a few mT, since this field is too small to affect the displaced Fermi surface. This logic has indeed been applied to distinguish the two types of nonlinear V_{xx}-I characteristics (i.e., superconductivity and Schwinger effect) in the papers cited by this referee [Tian, H. et al. *Nature* **614**, 440-444 (2023); Liu, L. et al. arXiv:2501.06460 (2025)]. For instance, the authors of [Liu, L. et al. arXiv:2501.06460 (2025)] mentioned "*The lower boundary j_s vanishes with increasing perpendicular magnetic field, indicating its association with the superconducting critical current. In contrast, the upper boundary j_n remains evident even at higher magnetic fields. This behavior suggests that j_n originates from the critical behavior of a correlated gap at half filling, arising from interband transitions via the Zener-Klein tunneling[43-46] at high current densities.*" Considering the nonlinear V_{xx}-I characteristics in our work are suppressed by a small $B_\perp$, we conclude that the Schwinger effect cannot account for our observations.

To address the referee's concern and improve our manuscript, we have added a new subsection "Ruling out other mechanisms for nonlinear dV_{xx}/dI" in Methods and included the discussion above.

5. Finally, the identification of superconductivity based on nonzero resistance still raises concerns. This issue is especially critical for a journal like Nature. The claim should be made more cautiously, given that the normal-state resistance is already very low (on the order of tens of ohms), comparable to the residual resistance in the purported superconducting state. As noted by another referee, there are precedents where initial claims of superconductivity were not substantiated by subsequent studies.

Response 16

We understand the referee's concern about nonzero remaining resistance. To be fully transparent with the referees and readers, we stated "The residual resistance relative to the normal-state resistance in some of the superconducting states, such as SC2 and SC4 in R5G, is relatively large compared to the others." in the subsection "Fraunhofer-like interference patterns and nonzero residual resistance" in Methods. However, we have several points to make as follows, which support the identification of superconductivity and guarantee that our standard of claiming superconductivity is comparable or higher than other groups' crystalline graphene superconductivity works published in *Nature*. Moreover, Referee #3, who had similar concerns on our initial claims of superconductivity and the precedented claims of superconductivity, are now fully convinced by our new data from original and new devices.

First of all, our data under $B_\perp$ shows **evidence for phase-coherent transport in SC1-4 in R5G, supporting the claims of SCs**. Figure R10 shows the evolution of SC1-4 under $B_\perp$. They show clear modulations of dV_{xx}/dI and R_{xx} curves by $B_\perp$. These are Fraunhofer-like interference patterns arising from weak inhomogeneity in the device. This evidence for phase-coherent transport, in addition to the resistance measurement, strengthens our claims of SCs.

Secondly, the residual resistance of SC1-4 shown in Fig. R11 is **not comparable to their normal-state resistance**. The resistance of SC1 and SC3 already approached zero within the noise level, and the suppression of R_{xx} for SC2 and SC4 is appreciable: from ~90 to ~6 Ω for SC2, with a residual resistance ratio to normal state of ~0.07; from ~120 to ~20 Ω for SC4, with a residual resistance ratio of ~0.17. This

is in contrast to the referee's statement 'the normal-state is comparable to the residual resistance in the purported superconducting state.' Additionally, we note that we are using comparable or higher standards to identify superconductivity, compared to several previous publications in *Nature*. As shown in Fig. R12, several previous reports on crystalline graphene superconductivities, including rhombohedral trilayer, bilayer, bilayer on WSe_2, show considerable residual resistance. These include SC2 in [Zhou, H. et al. *Nature* **598**, 434-438 (2021)], which was regarded by this referee as a superconductor (**Response 12**). Figure R12e summarizes the ratio of the residual resistance to the normal-state resistance of those superconductors, showing the values ranging from ~0.1 to ~0.5. These are similar to or far higher than the values for SC2 (~0.07) and SC4 (~0.17) we report.

Thirdly, we would like to note that our results are fully reproducible thanks to the crystalline nature of rhombohedral graphene. This was a major concern from Referee #3 in the first round, but Referee #3 is now fully convinced, given that we have reproduced the SC phenomenology from multiple devices, including two R4G and five R5G devices. **The reproducibility presented in our work highly surpasses the standard that can be found in the previous publications.** In addition, the SC phenomenology has been reproduced in two different fridges from two different labs, even across the continent. This signifies the physics we have discovered is extremely robust. These facts are ultimately due to the highly uniform crystalline structure of rhombohedral graphene, which should be distinguished from moiré superlattices. The latter indeed shows sample-to-sample fluctuations and significant spatial inhomogeneities within the sample owing to twist-angle disorder [Uri, A. et al. *Nature* **581**, 47-52 (2020); Lau, C. N. et al. *Nature* **602**, 41-50 (2022)]. The high uniformity and reproducibility are an important message of our work to the superconductivity community.

Again, we understand and agree with the referee's thoughts on the caveat in identifying superconductivity just based on resistance measurement. To improve our manuscript, we have included data in Fig. R10 as Extended Data Fig. 3 and updated the data for SC2 and SC4 in R5G in Fig. 2 of the main text, based on the most recent data set collected after improving the filtering and ground loops in the measurement setup.

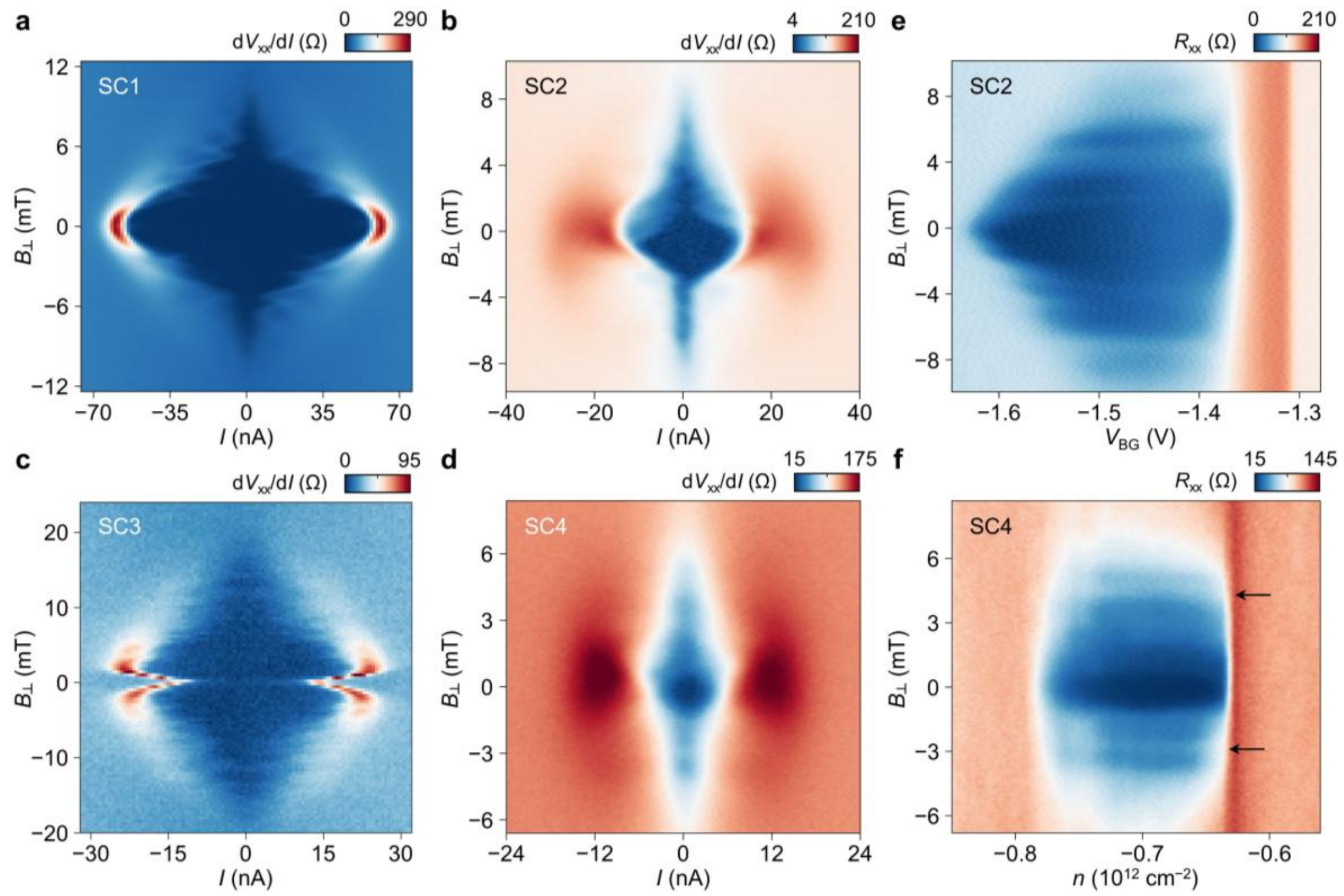

Figure R10. Fraunhofer-like interference patterns in R5G. a-d, Differential resistance as a function of *I* and

$B_\perp$ for SC1 ($n = -3.38 \times 10^{12}$ cm^{-2}, $D/\varepsilon_0 = 0.214$ V/nm, $B_\parallel = 0$ T, **a**), SC2 ($V_{BG} = -1.5285$ V, $V_{TG} = -0.2$ V, $B_\parallel = 6.5$ T, **b**), SC3 ($n = -1.135 \times 10^{12}$ cm^{-2}, $D/\varepsilon_0 = 57$ mV/nm, $B_\parallel = 0$ T, **c**) and SC4 ($n = -0.689 \times 10^{12}$ cm^{-2}, $D/\varepsilon_0 = 140.1$ mV/nm, $B_\parallel = 8.835$ T, **d**). **e,f,** R_{xx} as a function of $B_\perp$ for SC2 ($V_{TG} = -0.2$ V, $B_\parallel = 6.5$ T, **e**) and SC4 ($D/\varepsilon_0 = 140.1$ mV/nm, $B_\parallel = 8.835$ T, **f**). SC1-4 exhibit clear modulations by $B_\perp$, showing the Fraunhofer-like interference patterns.

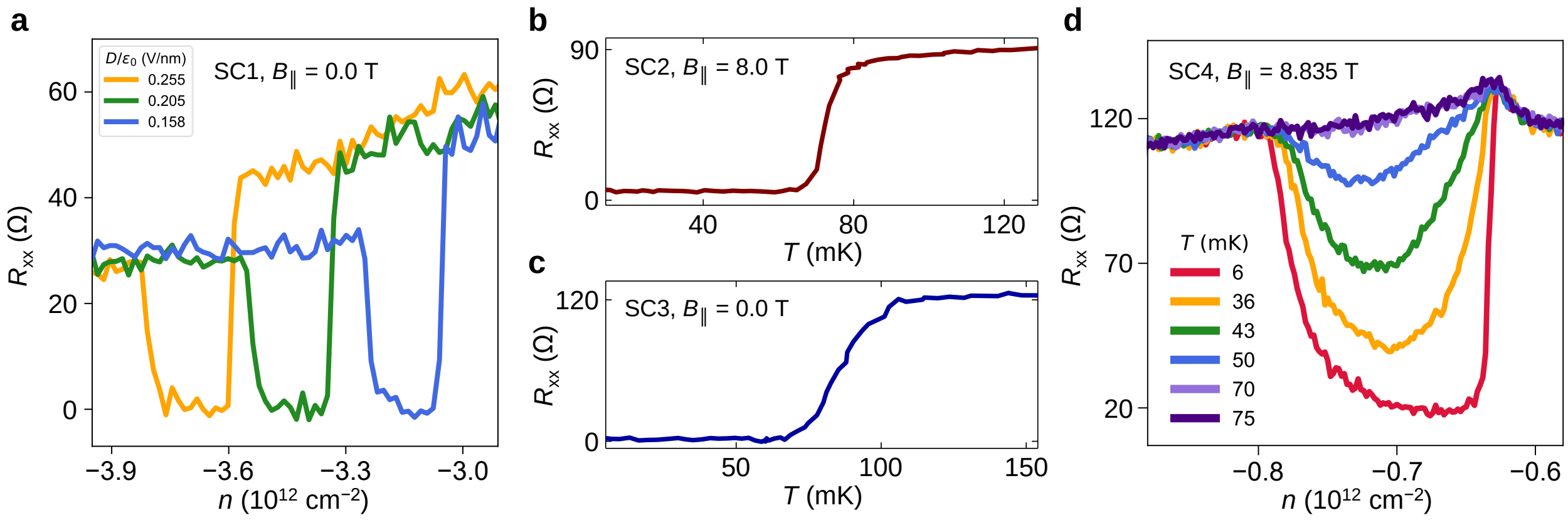

Figure R11. Residual resistance of SC1-4 in R5G. a, R_{xx} as a function of n at several values of D/ε_0 and the base temperature for SC1. **b,c,** Temperature-dependent R_{xx} of SC2 ($n = -1.055 \times 10^{12}$ cm^{-2}, $D/\varepsilon_0 = 93.17$ mV/nm, **b**) and SC3 ($n = -1.100 \times 10^{12}$ cm^{-2}, $D/\varepsilon_0 = 49.61$ mV/nm, **c**). **d,** R_{xx} as a function of n at varied temperatures for SC4 ($D/\varepsilon_0 = 140.1$ mV/nm). The resistance of SC1 and SC3 approaches 0 Ω. The residual resistance of SC2 and SC4 is smaller than the normal-state R_{xx} by an order of magnitude. The data were taken from Device R5G-2.

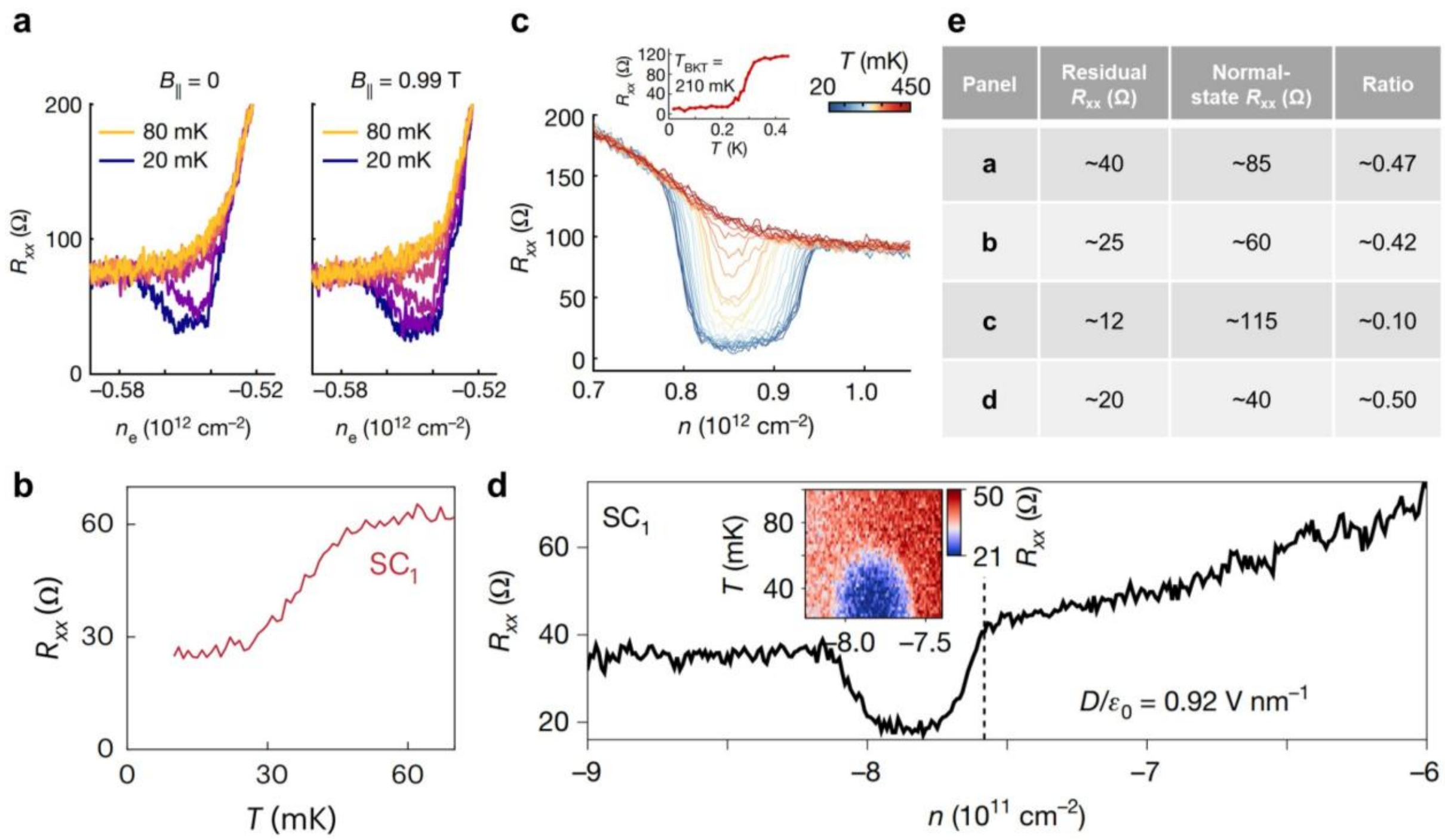

Panel	Residual R_{xx} (Ω)	Normal-state R_{xx} (Ω)	Ratio
a	~40	~85	~0.47
b	~25	~60	~0.42
c	~12	~115	~0.10
d	~20	~40	~0.50

Figure R12. Nonzero residual resistance of some graphene SCs in literature. a, Temperature-dependent R_{xx} of SC2 in R3G at $B_\parallel = 0$ (left) and 0.99 T (right) [Zhou, H. et al. *Nature* **598**, 434-438 (2021)]. **b,** Temperature-dependent R_{xx} of SC1 in R2G/WSe_2 [Holleis, L. et al. *Nat. Phys.* **21**, 444-450 (2025)]. **c,** Temperature-dependent R_{xx} of SC in electron-doped R2G/WSe_2 [Li, C. et al. *Nature* **631**, 300-306 (2024)]. **d,** R_{xx} of SC1 in R2G/WSe_2 with the optimized proximitized SOC effect [Zhang, Y. et al. *Nature* **641**, 625-631 (2025)]. **e,** Summary of the residual resistance, normal-state resistance, and the ratio of the two for the references in **a-d**.

Referee #2 (Comments on theoretical modelling):

I appreciate the authors' efforts. However, I still find the main conclusions to be premature.

More importantly, superconductivity in rhombohedral graphene has already been extensively reported in recent years. In this context, the present results appear largely complementary to prior studies rather than representing a substantial advance. In addition, the reported superconducting states seem less robust compared to those previously demonstrated.

Response 17

We respectfully disagree that our result is only complementary to prior studies. As we have explained in **Response 12**, the main point of our results (i.e., anomalous phenomenology under magnetic fields) has not been extensively reported in previous studies, although superconductivities in rhombohedral graphene have been reported in the past. The referee cited three references about crystalline graphene SCs in the question for **Response 12,** but two of them do not exhibit the enhancement by $B_{\parallel}$, including one from our own group that we ourselves know very well.

Furthermore, we disagree with the comment 'the reported superconducting states seem less robust compared to those previously demonstrated.' As explained in **Response 16**, (1) we have demonstrated Fraunhofer-like modulations by $B_{\perp}$ for all the four SCs in R5G. Most of the references in rhombohedral graphene SCs did not report these Fraunhofer-like patterns, which are more direct evidence for global phase coherence than vanishing resistance. (2) The ratio of the residual resistance to the normal-state resistance in these four SCs is comparable to or lower than the values from the literature (Fig. R12). We think the referee harbors wrong impressions on previous literatures, which we have clarified by direct comparison between the previous works and the current manuscript.

The out-of-plane magnetic-field-enhanced SC3 is also not observed here for the first time (see Sci. Adv. Eadq5712 (2025)). Therefore, its novelty should be more carefully justified.

Response 18

As we noted in **Response 13**, the enhancement in the reference cited by the referee was explained in the context of a 0-π junction. The 0-π junction has been studied extensively for more than 20 years. The enhancement of SC3 in our work is the **intrinsic** property of a superconductor itself, whose underlying physics is very different from a Josephson junction. We think the observation of a **superconductor** enhanced by $B_{\perp}$ is novel, and we have justified distinction between SC3 and [*Sci. Adv.* Eadq5712 (2025)] in the revised manuscript. We appreciate this referee's comment that allowed us to clarify this point.

Furthermore, the discussion regarding the mechanism of the SC3 phase remains speculative. In particular, it is unclear whether the intrinsically weak spin-orbit coupling (SOC) in graphene is sufficient to account for the observed phenomena. If SOC indeed plays a dominant role, one would expect a clear enhancement of SC3 in graphene/WSe_2 heterostructures due to proximity-induced SOC. However, this does not seem to be supported by the data presented in Fig. 4.

Response 19

We agree that the proposed mechanism can be discussed and even tested more in the future. However, at this moment, we would like to point out that the weak intrinsic SOC is not contradictory but consistent

with the observed phenomena. It is exactly the weak strength of intrinsic SOC that leads to the observed $B_\perp$ enhancement of SC3 at merely mT field values. A small $B_\perp$ could possibly impact the ground state significantly as we observed, only when SOC is weak; if the SOC is too strong as in TMD-proximitized graphene, the ground state will not be changed by a weak $B_\perp$ but is already fixed by the SOC and other larger energy scales.

The aforementioned picture is directly supported by our theory as well: Stronger SOC weakens the enhancement of SC by $B_\perp$. To show this, we start from the free energy density F:

$$F = \frac{\kappa}{2}(n_+^2 + n_-^2) - J_H \vec{n}_+ \cdot \vec{n}_- - \frac{\lambda}{2}(n_+^z - n_-^z) - h_\perp(n_+^z + n_-^z) - h_\parallel(n_+^x + n_-^x) - h_V(n_+ - n_-).$$

Here, g_V is the valley g-factor and λ determines the SOC strength. The last term accounts for the valley Zeeman effect, while the other terms follow the previous model. The valley g-factor averaged on the Fermi surface for $n = -1.1 \times 10^{12}$ cm^{-2}, $D/\varepsilon_0 = 50$ mV/nm was estimated to be around 6 by solving a 10-band Slonczewski-Weiss-McClure type tight-binding model [Han, T. et al. *Nature* **623**, 41-47 (2025); Zhou, H. et al. *Nature* **598**, 429-433 (2021)]. In addition, we took $\lambda = 0.05$ meV for bare R5G and 1.0 meV for SOC-proximitized R5G, following the reference [Zhang, Y. et al. *Nature* **641**, 625-631 (2025)].

Figure R13a shows the spin polarization s_z as a function of $B_\perp$ for the weak-SOC and strong-SOC case. The strong SOC decreases the spin polarization significantly. This is because the spin vectors are pinned more robustly when stronger SOC is imposed, as the Ising SOC serves as a valley-contrasting effective magnetic field. As a result, a small $B_\perp$ of few mT can hardly induce the spin polarization. Along with s_z, the valley imbalance arising from from the valley-contrasting energy $\pm\frac{\lambda s_z}{2}$ is suppressed. This is demonstrated in Fig. R13b where the valley imbalance at the optimal $B_\perp$ value is reduced by an order of magnitude (0.66% to 0.05%) by the SOC. Please note the impact on the valley imbalance is less significant than that on s_z, due to the additional factor of λ in the valley-contrasting energy.

The calculations in Fig. R13 are fully consistent with our experiments on the Device R5G/WS_2 (**Response 3**). We found that the enhancement of SC3 by $B_\perp$ disappears when strong SOC is proximitized to R5G. This is naturally connected to our theoretical results that the valley imbalance is hugely suppressed by the proximitized SOC. We hope this additional evidence clears the referee's concern about our theory.

In the revised manuscript, we discussed the impact of proximitized SOC effect on the enhancement of SC3 in the subsection "Proposed mechanism for the enhancement of SC3 by $B_\perp$ based on SOC" in the Methods Section as "Another check of the theory can be performed by increasing the SOC strength through the proximation to a TMD. … This is demonstrated in Extended Data Fig. 9e where the valley imbalance at the optimal $B_\perp$ is reduced by an order of magnitude (0.66% to 0.05%)."

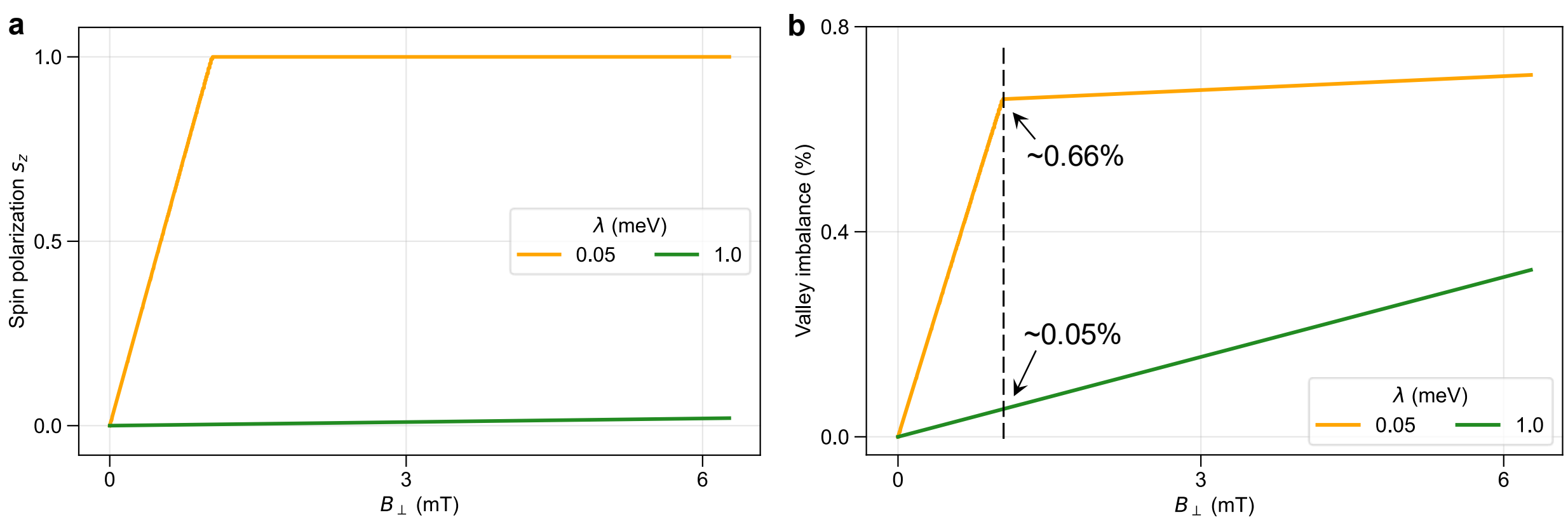

Figure R13. Effect of the proximitized SOC. a,b, Spin polarization s_z (**a**) and valley imbalance normalized by n_0

(**b**) as functions of $B_{\perp}$ for two different values of SOC strength. When the SOC is strong, the valley imbalance at the optimal field is reduced by an order of magnitude. This is in line with the observation that the enhancement of SC3 does not exist in the SOC-proximitized device.

Overall, additional evidence and a more rigorous analysis are required to substantiate the proposed interpretation.

Response 20

We hope our responses above (**Response 12-19**) have resolved the remaining issues.

Referee #3 (Remarks to the Authors):

I would like to thank the authors for providing detailed and thoughtful responses to my questions and for addressing the concerns I raised.

The new data, for both previously studied and newly fabricated devices of rhombohedral graphene multilayers, significantly strengthen the authors' claim of unconventional superconducting states in these systems and enhance the value of the results for the community. The additional measurement details further improve the credibility of the reported findings.

I recommend the revised version of the manuscript for publication.

Response 21

We thank the referee for supporting the publication of our work.

---- End of the response letter ----

A Possible Mechanism for $B_\perp$-induced Enhancement of SC3

1. Summary

To recap on the key experimental observations in the manuscript:

i. Superconductivity SC3 is enhanced by a small $B_\perp$~1.8 mT;

ii. A pure in-plane magnetic field $B_\parallel$ does not enhance SC3;

iii. The enhancement by $B_\perp$ is also absent when a large $B_\parallel$ is applied.

We provide a picture that is compatible with all three observations:

Our fermiology measurement determines that the parent state of SC3 is valley unpolarized half-metal. The spins from the two valleys are largely polarized in-plane with a small canting to the out-of-plane direction [1,2]. When a large enough $B_\perp$ is applied and the spins are polarized to out-of-plane, the spin-orbit coupling (SOC) $H_{SOC} = -\frac{\lambda}{2}\tau_z\sigma_z$ ($\tau_z = \pm 1$ is the valley, σ_z is the spin along the z-direction) induces a valley-contrasting change in the Fermi surface (FS). Assuming the pairing mechanism is intravalley and the valleys are decoupled or weakly coupled [3] due to the large momentum difference between the two valleys, the valley-contrasting change in the FS will result in the valley that has enhanced Density-of-States (DOS). This enhanced DOS can strengthen the superconductivity than that at zero magnetic field. The SOC-induced change in the FS disappears when the spin is aligned in-plane by $B_\parallel$. Hence, the enhancement by $B_\perp$ cannot be observed when $B_\parallel$ is applied.

In conclusion, the key points are:

i. $B_\perp$ aligns spins to the out-of-plane direction, and SOC induces a valley imbalance;

ii. The enhanced DOS in one valley enhances superconductivity, assuming intravalley pairing and at most weak coupling between the valleys;

iii. Such valley imbalance is absent when applying purely $B_\parallel$, or a large $B_\parallel$ plus a small $B_\perp$.

This proposed theory model has predicting power that can be directly tested by experiment: if the theory is correct, a small $B_\parallel$ of a few mT can slow down the enhancement of superconductivity by $B_\perp$, as the spins will be more robust in pointing to the in-plane direction and the valley-imbalance will require larger $B_\perp$ to realize. **This prediction was confirmed by the measurements *newly* added in this document (Section 3).**

2. Detailed explanations & Supporting calculations

To test the validity of the mechanism described above, we discuss below two key ingredients of the picture and support them with numerical calculations.

Firstly, we show that the SOC-induced valley-contrasting FS indeed dominates over other valley-contrasting energy scales at $B_\perp$ = 1.8 mT. According to the fermiology analysis of SC3, the Fermi energy cuts through the band residing on a single surface of rhombohedral graphene. As Referee 1 pointed out, the intrinsic spin-orbit coupling (SOC) in graphene acts as Ising SOC when projected to one layer. Thus, we take the SOC Hamiltonian as $H_{SOC} = -\frac{\lambda}{2}\tau_z\sigma_z$.

The SOC strength in bare graphene extracted from experiments is $\lambda \approx 50$ μeV [4-6]. This is much larger than other valley-contrasting energy scales at $B_\perp = 1.8$ mT, such as valley Zeeman energy $E_V = g_V \mu_B B_\perp \approx 2$ μeV ($g_V \approx 20$ is the valley orbital magnetic g-factor [7], μ_B is Bohr magneton) and spin Zeeman energy $E_S = g_S \mu_B B_\perp \approx 0.2$ μeV ($g_S \approx 2$ is the spin g-factor). Hence, if $\langle \sigma_z \rangle$ is substantial, the SOC provides the most dominant valley-contrasting energy scale. The SOC energy determines the maximal valley imbalance in FS and sets an upper bound of the enhancement of superconducting T_c and critical current I_c by at $B_\perp$.

Secondly, we show that $\langle \sigma_z \rangle$ is indeed almost saturated at $B_\perp$ = 1.8 mT, due to the interplay between the Hund's coupling and the SOC. This can be explicitly shown by a model calculation. We start from the phenomenological free energy from Ref. [1] and [2]:

$$F = \frac{\kappa}{2}(n_+^2 + n_-^2) - J_H \vec{n}_+ \cdot \vec{n}_- - \frac{\lambda}{2}(n_+^z - n_-^z) - h(n_+^z + n_-^z).$$

Here, $\vec{n}_{\tau_z}$ describes the total spin polarization for each valley with $\tau_z = \pm 1$. The first term describes the tendency to create the spin polarization in each valley separately. The second term is the Hund's coupling where $J_H \approx 2$ meV/(10^{12} cm^{-2}) [2]. The third term is the SOC, and the last term is the spin Zeeman energy with $h = g_S \mu_B B_\perp$. Since the valley-contrasting energy shift is much smaller than the Fermi energy (~meV), we assume $|\vec{n}_+| = |\vec{n}_-| \equiv n_0$ where $n = 2n_0$ is the total charge density. Taking the coordinate system in Fig. 1a, we can write the spin polarization vectors as $\vec{n}_\pm = n_0(\sin\theta_\pm, 0, \cos\theta_\pm)$.

We first sketch the situation for $h = 0$, which is described in Ref. [2]. In this case, $\theta_+ = \pi - \theta_- \equiv \varphi$. Plugging in $\vec{n}_\pm$, we obtain $F = \frac{\kappa}{4}n^2 - \frac{J_H n^2}{4}(1 - 2\cos^2\varphi) - \frac{\lambda n}{2}\cos\varphi$. The minimum of F is found for $\cos\varphi_0 = \frac{\lambda}{2J_H n}$.

Next, we include the spin Zeeman effect. Due to nonzero $B_\perp$, we cannot assume $\theta_+ = \pi - \theta_-$. Therefore, we use the bare form of the spin polarization vectors, and we get $F = \kappa n_0^2 - J_H n_0^2 \cos(\theta_+ - \theta_-) - \left(\frac{\lambda}{2} + h\right) n_0 \cos\theta_+ - \left(-\frac{\lambda}{2} + h\right) n_0 \cos\theta_-$. To minimize the free energy by setting $\partial F/\partial\theta_+ = \partial F/\partial\theta_- = 0$, we obtain $\left(\frac{\lambda}{2} + h\right)\sin\theta_+ = \left(\frac{\lambda}{2} - h\right)\sin\theta_-$. When re-writing the angles as $\theta_+ = \varphi_0 + \delta\theta_+$ and $\theta_- = \pi - \varphi_0 + \delta\theta_-$, we can assume $\delta\theta_\pm$ is small in the low-h limit. This leads to:

$$\left(\frac{\lambda}{2} + h\right)\sin\theta_+ \approx \left(\frac{\lambda}{2} + h\right)(\sin\varphi_0 + \delta\theta_+ \cos\varphi_0),$$
$$\left(\frac{\lambda}{2} - h\right)\sin\theta_- \approx \left(\frac{\lambda}{2} - h\right)(\sin\varphi_0 - \delta\theta_- \cos\varphi_0).$$
$$\rightarrow \delta\theta_+ + \delta\theta_- \approx -\frac{4h}{\lambda}\tan\varphi_0$$

The total spin polarization S_z is:

$$S_z = n_+^z + n_-^z = n_0(\cos\theta_+ + \cos\theta_-) = n_0[\cos(\varphi_0 + \delta\theta_+) - \cos(\varphi_0 - \delta\theta_-)]$$
$$\approx -n_0(\delta\theta_+ + \delta\theta_-)\sin\varphi_0 = \frac{2nh}{\lambda}\frac{1 - \cos^2\varphi_0}{\cos\varphi_0}$$

As a result, the spin polarization s_z per carrier is:

$$s_z = \frac{1}{n}S_z = \frac{g_S \mu_B B_\perp}{E_H}\left(\frac{4E_H^2}{\lambda^2} - 1\right)$$

Here, $E_H = J_H n \approx 2$ meV. Figure 1b shows both the linear approximation explained above and the numerical calculations that minimize the free energy F. The spin system reaches the saturation at $B_\perp \sim 2.6$ mT. **This value is fairly close to our experimental observed $B_\perp$ =**

1.8 mT, at which SC3 is maximally enhanced. The small difference in value could be due to the deviation of parameters in our system from those in Ref. [1] and [2].

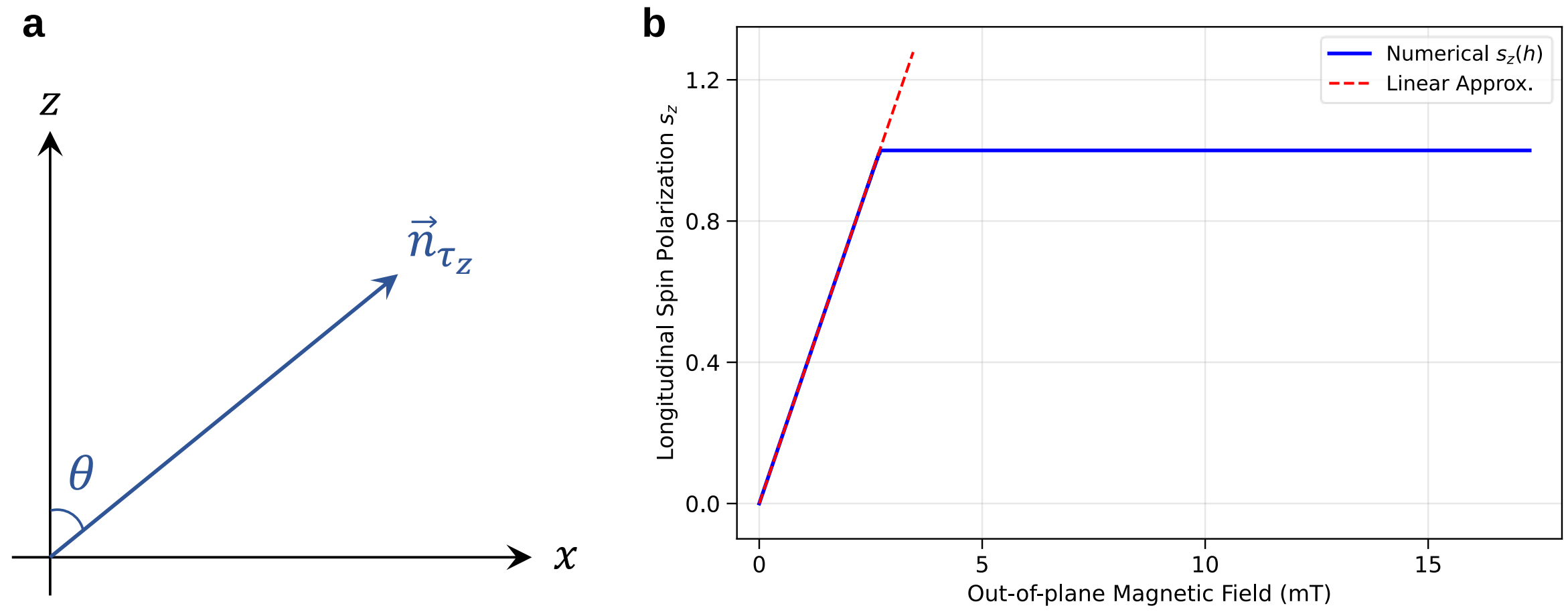

Figure 1. Interplay between the SOC and spin Zeeman effect. a, Spin polarization vector $\vec{n}_{\tau_z}$ drawn in the coordinate system. **b,** Calculated spin polarization per charge carrier s_z as a function of $B_\perp$. The numerically calculated values are consistent with those derived based on the linear approximation. The parameters used are: $E_H = 2$ meV, $g_S = 2$, $\lambda = 50$ μeV [2,6]. The spins are fully polarized out-of-plane with spin Zeeman energy lower than λ by two orders of magnitude, as the Hund's coupling prefers aligned spins.

Combining the two facts above, we further explain our picture. Since the parent state of SC3 is valley-unpolarized, both intravalley and intervalley pairings are possible [8]. If we assume in SC3 the intravalley pairing dominates the intervalley pairing, the pairing in each valley can be treated independently [3] because of large momentum difference between K and K'. Due to the opposite Fermi energy shift in the two valleys by SOC, the intravalley pairing strength will increase in one valley and decrease in the other valley. The density of states, which directly influences the robustness of intravalley pairing [9], will increase in one valley and decrease in the other valley. Since the global transport of a superconductor is dominated by the stronger pairing channel, superconductivity can be enhanced by applying small $B_\perp$. When s_z saturates to 1, the SOC effect no longer alters the FS and the enhancement accordingly stops. At higher $B_\perp$, SC3 will only be suppressed by the orbital depairing effect. These expectations agree with our experimental observations.

Our picture also explains why the enhancement of SC3 by $B_\perp$ disappears under a large $B_\parallel$. Under $B_\parallel$, the electron spins are fixed to the in-plane direction. Hence, the SOC does not result in energy change: $\langle \rightarrow_x | \sigma_z | \rightarrow_x \rangle = 0$. This leads to negligible change in the FS by $B_\perp$, and the enhancement phenomenon disappears. We also note that our scenario is consistent with the absence of the $B_\perp$-hysteresis of SC3 in its superconducting state, since the change of FS by the Ising SOC and $B_\perp$ is smooth rather than abrupt.

3. New evidence for the suggested theory/model

As pointed out in the last paragraph of Section 2, s_z plays a crucial role in our model, because it results in the valley-contrasting FS through the SOC effect. The magnitude of s_z induced by $B_\perp$ can be tuned by $B_\parallel$, since the latter competes with the former in determining the direction of electron spins. This is evident in the calculation shown in Fig. 2a, which is obtained by

minimizing the free energy including the spin Zeeman effect by both $B_\perp$ and $B_\parallel$:

$$F = \frac{\kappa}{2}(n_+^2 + n_-^2) - J_H \vec{n}_+ \cdot \vec{n}_- - \frac{\lambda}{2}(n_+^z - n_-^z) - h(n_+^z + n_-^z) - h_\parallel(n_+^x + n_-^x).$$

Here, $h_\parallel = g_S \mu_B B_\parallel$ accounts for the effect from the in-plane field. Not only is a larger $B_\perp$ required to reach $s_z = 1$, but also the slope $\mathrm{d}s_z/\mathrm{d}B_\perp$ at $B_\perp = 0$ decreases under larger $B_\parallel$. Thus, **<u>it is expected that (1) the optimal $B_\perp$ value at which SC3 becomes the strongest increases when a small $B_\parallel$ of few mT (i.e., comparable to 1.8 mT) is applied, and (2) the rate at which the critical current I_c increases by $B_\perp$ is reduced by applying $B_\parallel$</u>**.

Experimentally, we have tuned $B_\perp$ and $B_\parallel$ precisely at the same time to test these predictions by our theory model. Figure 2b-e show the evolution of critical current by $B_\perp$ under varied $B_\parallel$. There are two general trends that can be seen, and we highlight them by comparing the $B_\parallel = 0$ and 0.5 mT data in Fig. 2b&c. Firstly, the optimal value of $B_\perp$ giving the largest I_c, indicated by the horizontal yellow and green dotted lines for $B_\parallel = 0.0$ and 0.5 mT respectively, is increased by applying $B_\parallel$. Secondly, the rate of increase in I_c by $B_\perp$, denoted by the slope of the yellow and green slanted dashed lines for $B_\parallel = 0.0$ and 0.5 mT respectively, is decreased by $B_\parallel$. These two observations are fully consistent with the prediction by our theory model. Figure 2f shows a summary of the data points corresponding to maximal dV/dI from Fig. 2b-e, in which the two aforementioned trends can be seen clearly.

We note that $B_\perp$ can suppress the critical current of superconductivity by inducing orbital depairing effect, concurrent with the enhancement by $B_\perp$ indirectly through the valley-contrast FS by the SOC effect. Hence, the maximal critical current value shows a decreasing trend with increased $B_\parallel$, as the optimal $B_\perp$ gets pushed to higher values by $B_\parallel$, as can be seen in Fig. 2f.

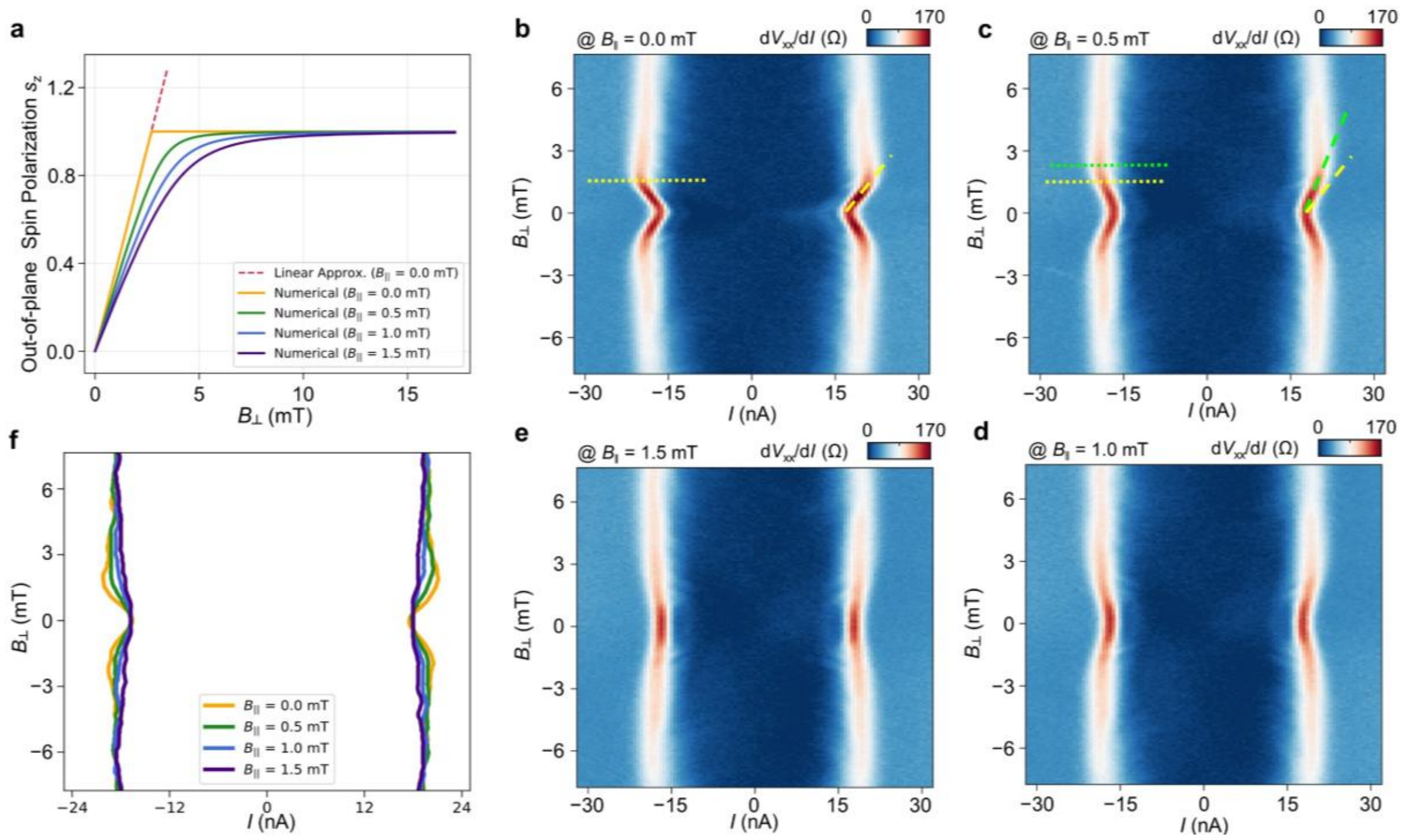

Figure 2. Effect of $B_\parallel$ on the out-of-plane spin polarization and enhancement of SC3. a, Calculated spin polarization per charge carrier s_z as a function of $B_\perp$ at varied $B_\parallel$. At increased $B_\parallel$, it takes larger $B_\perp$ to develop and saturate s_z. **b-e,** Differential resistance as a function of I and $B_\perp$ in SC3 from Device R5G-3 at $B_\parallel = 0.0$ (**b**), 0.5 (**c**), 1.0 (**d**) and 1.5 (**e**) mT, respectively. As the $B_\parallel$ increases, the $B_\perp$ corresponding to maximized dV/dI is increased, as can be seen from the comparison between the yellow and green dotted lines, labeling the maximal I_c at $B_\parallel = 0$ and 0.5 mT respectively. At the same time, the enhancement of

critical current with $B_\perp$ gets slower, as can be seen by the comparison of yellow and green dashed lines in **c**, tracing the slope of maximal d*V*/d*I* point on the *I*-$B_\perp$ map at $B_\parallel = 0$ and 0.5 mT respectively. These observations are due to the pinning of electron spins by $B_\parallel$ to the in-plane direction and the less efficient polarizing of spins by $B_\perp$. They are in full agreement with the prediction of our theory model and the numerical calculation in **a**. **f,** The current *I* corresponding to the maximal d*V*/d*I* at each $B_\perp$ and $B_\parallel$, extracted from **b-e**.

4. References

[1] Dong, Z., Lantagne-Hurtubise, É. & Alicea, J. *Phys. Rev. X* **16**, 011055 (2026).

[2] Patterson, C. L. et al. *Nature* **641**, 632-638 (2025).

[3] Wang, K. & Levin, K. arXiv:2510.06451v3 (2026).

[4] Sichua, J. et al. *Phys. Rev. Lett.* **122**, 046403 (2019).

[5] Banszerus, L. et al. *Phys. Rev. Lett.* **124**, 177701 (2020).

[6] Arp, T. et al. *Nat. Phys.* **20**, 1413-1420 (2024).

[7] Han, T. et al. *Nature* **623**, 41-47 (2023).

[8] Hsu, Y.-T. et al. *Nat. Commun.* **8**, 14985 (2017).

[9] Chou, Y.-Z., Zhu, J. & Sarma, S. D. *Phys. Rev. B* **111**, 174523 (2025).

Responses to the referees

We sincerely thank the referees for taking the time to assess our manuscript and raising suggestions to improve it. We believe that this letter and the revised manuscript properly addressed their questions and comments. Throughout the letter, we used blue color for the comments by the referees and black color for our responses. Changes made in the revised manuscript are highlighted in yellow.

Referee #1 (Remarks to the Authors):

The authors have answered my questions satisfactorily. First, the addition of data from a new spin-orbit proximitized R5G device, and the confirmation that no anomalous enhancement of superconductivity occurs under a perpendicular magnetic field, completes the experimental picture.

On the theory side, I am glad to see that (as expected on general symmetry grounds) the modeling holds up when relaxing the unphysical assumption that the two valleys must host equal spin polarization, as pointed out in my previous note. The addition of valley Zeeman coupling, and the direct computation of valley imbalance (as opposed to indirectly through spin imbalance) paints a clear story, with immediate observable consequences that could be tested in future experiments (e.g. using magnetometry). The additional discussion of the role of enhanced Ising SOC, and the corresponding suppression of induced valley imbalance by B_perp, nicely complements the new data on the spin-orbit proximitized R5G device mentioned above. Overall, I think the revised theory modeling is a valuable addition to the manuscript.

Response 1

We thank the referee for appreciating the new results added during the previous revision.

I would simply ask that the authors cite the manuscript Kumar et al. in the context of their theory modeling, since the fully unconstrained (n_+ != n_-) free energy minimization in a field-biased spin-canted state originates from there (hence the inspiration behind my question in the previous report).

Response 2

We appreciate the referee's comment. In the revised manuscript, we added the reference [Kumar, M. et al. arXiv:2511.16578v2 (2026)] as Ref. 70 and cited it as "We provide calculations supporting the aforementioned scenario. We consider the free energy density F (ref. [33,67,70]):" in the subsection "Proposed mechanism for the enhancement of SC3 by $B_\perp$ based on SOC" in Methods.

Overall I find the work to be very timely and interesting. It uncovers new and intriguing phenomenology within the large family of rhombohedral graphene superconductors, along with a reasonable theoretical proposal for the unconventional magnetic field response. The dataset is extensive, and now contains multiple R4G and R5G devices in both hBN and TMD-proximitized configurations, which allows for an interesting discussion of the role of induced SOC on superconducting phenomenology in rhombohedral systems. I am convinced the results will inspire many follow-up experiments and theory efforts aimed at resolving the remaining puzzles, and I therefore recommend its publication in Nature.

Response 3

We thank the referee for supporting the publication of our work.

Device	Observed SCs	$R_{SC,residual} / R_n$		Anomalous magnetic field responses
R4G−1	SC1−2	SC1	≈0	N.A.
		SC2	0.09	
R4G−2	SC1−2	SC1	0.07	N.A.
		SC2	0.13	
R5G−1	SC1−3<br>SC4 at high $B_\parallel$	SC1	≈0	SC2 enhanced by $B_\parallel$<br>SC3 enhanced by $B_\perp$<br>SC4 induced by $B_\parallel$
		SC2 (8.0 T)	0.33	
		SC3	0.09	
		SC4 (8.0 T)	0.55	
R5G−2	SC1−3<br>SC4 at high $B_\parallel$	SC1	≈0	SC2 enhanced by $B_\parallel$<br>SC3 enhanced by $B_\perp$<br>SC4 induced by $B_\parallel$
		SC2 (8.0 T)	0.07	
		SC3	≈0	
		SC4 (8.835 T)	0.17	
R5G−3	SC1−3<br>SC4 at high $B_\parallel$	Not tested above the base temperature		SC2 enhanced by $B_\parallel$<br>SC3 enhanced by $B_\perp$<br>SC4 induced by $B_\parallel$
R5G−4	SC1−3<br>Not tested at high $B_\parallel$	Not tested above the base temperature		SC3 enhanced by $B_\perp$
R5G−5	SC1−3<br>Not tested at high $B_\parallel$	Not tested above the base temperature		SC3 enhanced by $B_\perp$
R4G/WSe_2	SC1 & SC3−7	SC1	≈0	N.A.
		SC3	0.10	
		SC4	≈0	
		SC5	0.04	
		SC6	≈0	
		SC7	≈0	
R5G/WS_2	SC1−3<br>Not tested at high $B_\parallel$	Not tested above the base temperature		N.A.

Table R1. Summary of the studied devices. $R_{SC,residual}$ is the measured R_{xx} at the base temperature (i.e., below the SC transition temperature) of 7 mK. R_n is the normal-state resistance, defined as largely temperature-independent R_{xx} above the transition temperature (see Extended Data Fig. 3c for example). The values of $R_{SC,residual} / R_n$ are measured at zero magnetic fields, unless the in-plane magnetic field is specified. Since these values require temperature-dependent measurements, we did not put them for devices that were characterized at the base temperature only. For the devices measured at the base temperature only, the enhancement of SC3 by $B_\perp$ was concluded from the increase in I_c by applying small $B_\perp$. Here, the critical current could be extracted from the position of the DC current for peaks in dV_{xx}/dI.

Referee #2 (Remarks to the Authors):

I appreciate the authors' substantial efforts in addressing my previous concerns. The additional data have improved the manuscript and have made the experimental phenomenology clearer and more convincing.

Response 4

We thank the referee for appreciating the improvements made in our manuscript.

My remaining comment concerns the presentation of the experimental criteria. The definitions of residual resistance, normal-state resistance, and critical current should be stated more explicitly. I encourage the authors to include a concise device summary table, listing the measured devices, the observed superconducting states, base temperatures, residual-resistance ratios, magnetic-field responses, and the criteria used to extract Ic. Such a summary would make the reproducibility and robustness of the superconductivity claimed easier to assess.

Response 5

We appreciate the referee for the suggestions. Firstly, we described the criteria to extract I_c in the caption of Figure 3 as "Critical currents (extracted from peaks in dV_{xx}/dI from Extended Data Fig. 9f-k)," so that the readers can be aware of our methodology. Secondly, we added Table R1 as Table S1 in the Supplementary Information, summarizing the list of measured devices, observed superconducting states, residual-resistance ratio (i.e., $R_{SC,residual}/R_n$) and observed magnetic-field responses. We also provided the definition of residual resistance, normal-state resistance, and the criteria to determine I_c in the table caption.

Provided that this point is appropriately addressed in the revised manuscript, I would be pleased to support publication and would not require further review of the manuscript.

Response 6

We thank the referee for supporting the publication of our work.
